# Effects of carbohydrate-restricted diets and macronutrient replacements on cardiovascular health and body composition in adults: a meta-analysis of randomized trials

**DOI:** 10.1016/j.ajcnut.2025.09.012

**Published:** 2025-09-08

**Authors:** Shuo Feng, Renming Liu, Christopher Thompson, Brian Colwell, Sunghyun Chung, Adam Barry, Huishan Wang

**Affiliations:** 1 Department of Health Behavior, Texas A&M University, College Station, TX, United States; 2 Department of Educational Psychology, Baylor University, Waco, TX, United States; 3 Department of Educational Psychology, Texas A&M University, College Station, TX, United States

**Keywords:** carbohydrate-restricted diets, cardiovascular health, body composition, lipid profiles, inflammatory markers, meta-analysis, macronutrient replacement

## Abstract

**Background:**

Carbohydrate-restricted diets (CRDs) are widely promoted for improving cardiovascular and body composition outcomes, yet evidence remains mixed across dietary patterns, populations, and study designs.

**Objectives:**

This meta-analysis evaluated the effects of CRDs on cardiovascular and anthropometric outcomes and examined whether these effects varied by diet type, macronutrient replacement, participant characteristics, and study features.

**Methods:**

Following Preferred Reporting Items for Systematic Reviews and Meta-Analyses 2020 guidelines, 174 randomized trials (*n* = 11,481) from 27 countries were included. Eligible studies compared CRDs (≤45% of energy from carbohydrates) to higher-carbohydrate diets in adults and reported cardiovascular or anthropometric outcomes. Random-effects models estimated standardized mean differences (SMDs) and 95% confidence intervals (CIs). Subgroup analyses explored differences by CRD type (ketogenic, low-carb, and moderate-carb), replacement macronutrient (fat, protein, or combination), sex, weight status, diabetes status, intervention delivery, caloric intakes, and study design. Metaregressions assessed the effects of intervention duration.

**Results:**

CRDs significantly reduced triglycerides [SMD: –15.11 mg/dL; 95% CI: –18.76, –11.46], systolic (SMD: –2.05 mmHg; 95% CI: –3.13, –0.96) and diastolic blood pressure (SMD: –1.26 mmHg; 95% CI: –1.94, –0.57), various lipid profile ratios, and inflammatory markers (C-reactive protein, tumor necrosis factor-alpha), whereas increasing high-density lipoprotein (SMD: 2.92 mg/dL, 95% CI: 2.10, 3.74). Low-density lipoprotein (LDL) and total cholesterol increased modestly (SMD: 4.81, 95% CI: 2.58, 7.05; and SMD: 4.32 mg/dL, 95% CI: 1.66, 6.97, respectively). All measured body composition markers showed significant reductions. Moderate-carbohydrate diets offered balanced benefits, whereas ketogenic diets produced greater weight loss but greater increases in LDL and total cholesterol. Combined fat and protein replacements yielded the most comprehensive improvements. Benefits were most pronounced in females and individuals with overweight or obesity. Longer interventions amplified effects on lipid ratios and inflammatory markers.

**Conclusions:**

CRDs improved cardiovascular health and body composition, especially in diets with combined macronutrient replacement. Potential adverse effects, including LDL elevation and lean mass loss, warrant clinical monitoring.

**Trial registration number**: for body composition, PROSPERO: CRD420251043066 (https://www.crd.york.ac.uk/PROSPERO/view/CRD420251043066); for cardiovascular health: CRD420251011748 (https://www.crd.york.ac.uk/PROSPERO/view/CRD420251011748).

## Introduction

Cardiovascular diseases (CVDs), as the leading cause of death worldwide, account for 32% of all deaths [1]. The major contributor, overweight/obesity [2], defined as excessive and abnormal fat accumulation, is affecting ∼ 2.5 billion adults aged 18 y and older (BMI >25 kg/m^2^), including over 890 million living with obesity (BMI >30 kg/m^2^) [3]. The global economic burden is projected to rise to $3 trillion annually by 2030 and over $18 trillion by 2060 [4]. Promoting cardiovascular health and a healthy body composition remains a global public health priority.

Carbohydrate-restricted diets (CRDs), originally developed as a therapeutic intervention for epilepsy [5], have demonstrated potential clinical benefits across a broad spectrum of health conditions, including neurodegenerative diseases [6] and cancer [7]. Moreover, their roles in cardiovascular health and weight management have been recognized. Meta-analyses have shown that higher-carbohydrate intake is associated with increased risk of CVDs [8,9], including coronary artery disease [10,11] and stroke [10,12]. Comparatively, CRDs have demonstrated favorable effects on cardiovascular biomarkers, such as reduced triglycerides (TG) and blood pressure, along with increased HDL levels [13,14]. Parallel evidence supports the benefits of CRDs on anthropometric outcomes, including reductions in body weight (BW), BMI, body fat percentage (BFP) [[15], [16], [17], [18]], as well as fat mass (FM), fat-free mass (FFM), waist circumference (WC), and visceral adipose tissue (VAT) [18].

Despite these findings, the effects of CRDs on cardiovascular health and body composition remain inconsistent. Some studies report that CRD increased LDL and total cholesterol (TC) [13,14], markers traditionally considered adverse for cardiovascular disease risk. Others have found no significant improvements in blood pressure, lipid profiles [19], or body composition metrics such as FM [15], WC [16], BW, BFP, or lean mass (LM) [20,21]. Further complicating the evidence base is a proposed U-shaped relationship between carbohydrate intake and mortality, suggesting that both very low and very high carbohydrate consumption may elevate health risks [22]. Additionally, the precise role of diet duration remains unclear [18].

To address these knowledge gaps and clarify the mixed findings, this meta-analysis aims to systematically evaluate the effects of CRDs on a broad range of cardiovascular and anthropometric outcomes. Subgroup analyses and metaregressions examined differences by CRD type [ketogenic diet (KD): ≤10% of kcal, low-carb diet (LCD): 10%–26% of kcal, and moderate-carb diet (MCD): 26%–45% of kcal] [23] and replacement type (fat/protein/both), intervention length, participant characteristics (sex, diabetic status, and weight status), study design (consultation-only and food-provided; randomized crossover and parallel), and energy intakes [isocaloric (±5%) [24] and nonisocaloric].

## Methods

### Search strategy

This systematic review and meta-analysis followed the PRISMA 2020 guidelines [25] and was registered with PROSPERO (registration number and link are blinded for peer-review purposes). To ensure a comprehensive assessment, minor deviations from the registered protocol were implemented during data extraction. These included an expanded evaluation of cardiovascular outcomes, such as soluble intercellular adhesion molecule-1 (sICAM-1) and soluble vascular cell adhesion molecule-1 (sVCAM-1), and additional subgroup analyses (e.g., energy intake). A comprehensive search was conducted across 5 electronic databases—PubMed, Medline, Embase, Education Resources Information Center and Web of Science—to identify relevant randomized trials, including randomized crossover trials and randomized parallel-group trials. No restrictions were placed on language or publication date during the initial screening phase. Search terms were developed based on previous literature in the field [18,[26], [27], [28]], with the full strategy provided in the Supplementary Material. The final database search was conducted on March 14, 2025, covering studies from database inception to that date. All references were imported into Covidence systematic review software, which was used for managing title/abstract screening, full–text review, and duplicate removal. The screening process lasted ∼2 mo until mid-May.

### Eligibility criteria

Following the population, intervention, comparator, outcomes, and study design framework, studies were selected based on the following eligibility criteria: *1*) population: studies conducted on adults aged 18 y and older; *2*) intervention: randomized trials assessing the effects of macronutrient intake (e.g., variation in carbohydrate, fat, or protein intake) on anthropometric measures and body composition; *3*) comparator: studies employing either a crossover design with adequate washout periods or parallel-group designs with appropriate control or alternative diet comparison groups; *4*) outcomes: studies reporting ≥1 of the following measures, for blood pressure (mmHg): systolic (SBP) and diastolic (DBP), for lipid and apolipoprotein profiles: TG (mg/dL), TC (mg/dL), LDL (mg/dL), HDL (mg/dL), non-HDL (mg/dL), VLDL (mg/dL), LDL–HDL ratio, TC-HDL ratio, TG-HDL ratio, apolipoprotein A-I (ApoA1, g/L), apolipoprotein B-100 (ApoB, g/L), ApoB/ApoA1 ratio, for endothelial function: flow-mediated dilation (%), E-selectin (ng/mL), sICAM-1 (ng/mL), sVCAM-1 (ng/mL), for inflammatory biomarkers: C-reactive protein (CRP, mg/L), TNF-alpha (pg/mL), and IL-6 (pg/mL), and predicted 10-y CVD risk (%). Body composition outcomes include BW (kg), BMI (kg/m^2^), FM (kg), BFP (%), LM (kg), FFM (kg), WC (cm), hip circumference (HC, cm), waist-to-hip ratio (WHR), and VAT (kg); *5*) study design: original peer-reviewed studies with randomized trials, either a single-arm crossover design or multiple-arm parallel designs.

In addition, studies must report sufficient data to calculate delta mean and pooled SDs for pre- and postintervention comparisons (detailed in the Supplementary Material). Only studies published in English were included. Two reviewers (SF and RL) independently screened the titles, abstracts, and full texts of the identified studies. Disagreements were resolved through discussion and consensus.

### Data extraction

From each eligible study, the following data were extracted: study title, first author’s last name, and key intervention characteristics (e.g., country, study design, intervention type, duration, calorie intakes, and macronutrient distribution). Participant characteristics included sex distribution, mean age, baseline BMI, diabetes status, and weight classification. Additional details collected included the number of participants per group and the type of reported outcomes. For each outcome, the extracted data encompassed delta means (post and pre mean differences), pooled SDs, SE, 95% confidence interval (CI), median and IQR, as well as pre- and postintervention means, SDs, SEs, and 95% CIs. Data extraction was conducted by one reviewer (SF) and independently cross-checked by 2 additional reviewers (SC and HW). Any discrepancies were resolved through discussion and mutual consensus. There is no acknowledged missing data during the data extraction. The extracted data were converted to delta means and their associated SDs for analysis (detailed in the Supplementary Material).

### Risk of bias assessment

The Cochrane Risk of Bias 2 (RoB 2) tool [29] was used for evaluating the risk of bias in 5 domains: *1*) selection bias arising from the randomization process; *2*) performance bias due to deviations from intended interventions; *3*) attrition bias; *4*) detection/outcome bias in measurement; and *5*) reporting bias. Each domain was rated as having low risk, some concerns, or high risk of bias. Two reviewers (SF and RL) evaluated the included studies independently. Discrepancies were discussed and resolved with consensus. A traffic-light plot was used to visualize the risk of bias judgments across domains for each study.

### Statistical analysis

We performed and reported a given meta-analysis only when the number of independent data points reached ≥4 (*k* ≥ 4) [30]. Given the anticipated heterogeneity among studies, a random-effects model was employed to estimate standardized mean differences (SMDs) with corresponding 95% CIs. The restricted maximum likelihood (REML) was used for conducting the analyses. REML provides less biased estimates of between-study variance (τ^2^) compared with DerSimonian-Laird or other methods, and it is a preferred estimation method for random-effects meta-analysis and metaregression [[31], [32], [33]]. For data harmonization and consistency, due to variability in reported units across studies, all biomarker outcomes were converted to a common unit (e.g., mg/dL for lipid profiles) using standardized conversion factors. Detailed formulas are provided in the Supplementary Material. Heterogeneity was assessed by Cochran’s *Q* test and the *I*^2^ statistic. Forest plots and funnel plots were used to represent the meta-analysis results and associated publication bias.

After conducting the main meta-analysis for each outcome, subgroup analyses were conducted with grouping by macronutrient proportions (KD: ≤10% of total calorie or 20–50 g/d, LCD: 10%–26% of total calories or 50–130 g/d, and MCD: 26%–45% of total calorie or 130–230 g/d) [23], carbohydrate replacements (fat compared with protein compared with a combination of both) (see Supplementary Material for macronutrient calculation), dietary intervention length, sex (females compared with males), diabetic status [nondiabetic compared with type 2 diabetes (T2DM)], weight status (nonoverweight compared with overweight/obese). Finally, given concerns about consultation-only interventions in prior meta-analyses [34], this study also investigates differences between consultation-only and food-provided interventions, as well as between randomized crossover and parallel study designs, and between isocaloric (small energy margin with ∼±5%) [24] and nonisocaloric interventions (including personalized calorie intake calculated by the Harris-Benedict equation, ad libitum, etc.).

For each subgroup (e.g., males and females), we performed separate random-effects meta-analyses. Between-group heterogeneity was assessed using Cochran’s *Q*-between statistic (Qb) to determine whether the subgroup moderators could explain the observed variability in effect sizes. Lastly, the effects of diet length (in weeks) were assessed via metaregression analyses. Stata SE version 17.0 was used for analyses (Stata Corp LLC).

### Multiple testing correction

To control the family-wise error rate for multiple comparisons, a hybrid version of the Bonferroni correction was used. The corrected significance threshold (*α*∗) was calculated as *α*∗=*α*/*m*, where α∗ is the corrected alpha (i.e., type I error rate), α is original significance level (here, set as 0.05), and *m* is the total number of independent tests, computed as the sum of the number of omnibus tests (equal to the number of moderators, 8) and the number of multiple comparisons within each moderator (a total of 12). The corrected significance threshold was 0.0025 (i.e., 0.05/20). A test result was considered statistically significant if its *P* value ≤0.0025, ensuring a conservative approach to type I error. This decision balances specificity (reducing false positives) whereas acknowledging the exploratory nature of subgroup analyses.

### Sensitivity analysis

The robustness and reliability of the meta-analytic findings were tested by sensitivity analyses, which are repeated analyses excluding data from studies rated as having a high risk of bias, based on the RoB 2 tool, and outliers. After comparing the results of sensitivity analyses with the original meta-analyses, notable deviations in effect size or statistical significance are reported and interpreted in the context of potential bias and heterogeneity.

## Results

### Included study characteristics

**FIGURE 1:**
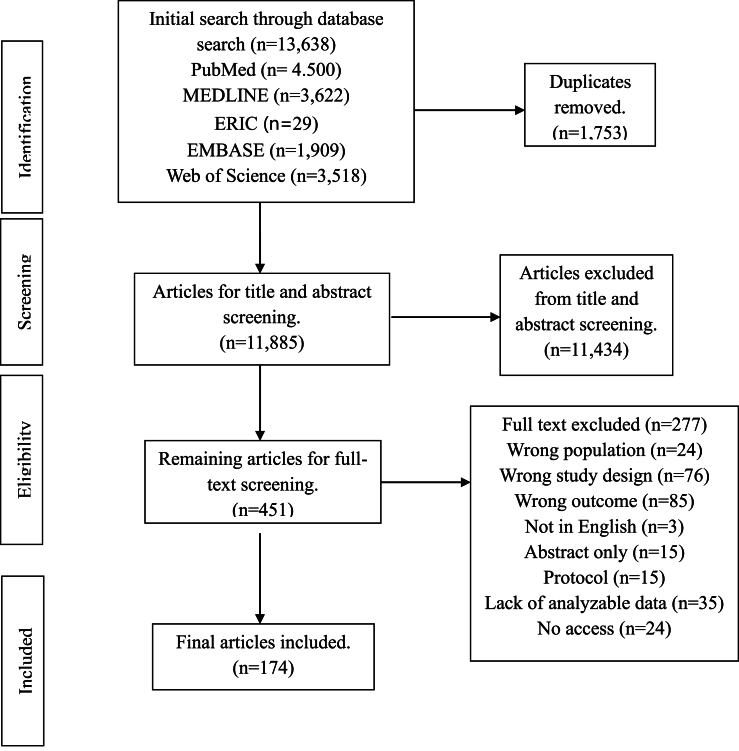
PRISMA diagram. ERIC stands for Education Resources Information Center.

A total of 13,638 publications were identified through the initial database search. After removing 1753 duplicates, 11,885 records remained for title and abstract screening. Of these, 11,434 were excluded based on relevant criteria. The remaining 451 full–text articles were assessed for eligibility, resulting in 174 studies being included in the final meta-analysis [[35], [36], [37], [38], [39], [40], [41], [42], [43], [44], [45], [46], [47], [48], [49], [50], [51], [52], [53], [54], [55], [56], [57], [58], [59], [60], [61], [62], [63], [64], [65], [66], [67], [68], [69], [70], [71], [72], [73], [74], [75], [76], [77], [78], [79], [80], [81], [82], [83], [84], [85], [86], [87], [88], [89], [90], [91], [92], [93], [94], [95], [96], [97], [98], [99], [100], [101], [102], [103], [104], [105], [106], [107], [108], [109], [110], [111], [112], [113], [114], [115], [116], [117], [118], [119], [120], [121], [122], [123], [124], [125], [126], [127], [128], [129], [130], [131], [132], [133], [134], [135], [136], [137], [138], [139], [140], [141], [142], [143], [144], [145], [146], [147], [148], [149], [150], [151], [152], [153], [154], [155], [156], [157], [158], [159], [160], [161], [162], [163], [164], [165], [166], [167], [168], [169], [170], [171], [172], [173], [174], [175], [176], [177], [178], [179], [180], [181], [182], [183], [184], [185], [186], [187], [188], [189], [190], [191], [192], [193], [194], [195], [196], [197], [198], [199], [200], [201], [202], [203], [204], [205], [206], [207], [208]]. The PRISMA flow diagram (Figure 1) illustrates the detailed study selection process. The included studies were published between 1992 and 2025, representing a sample of 11,481 participants from 27 countries. Comprehensive study characteristics, including participant demographics, study design, intervention types, and reported outcomes, are summarized in Supplemental Table 1.

### Risk of bias

On the basis of the Cochrane RoB 2 tool, 59% (*k* = 102) had some concerns, 33% (*k* = 57) had low risk, and 8% (*k* = 15) had high risk of bias. A visualization of the risk of bias assessment is presented in a traffic-light plot (Supplemental Figure 1).

### Meta-analysis results: overall effect sizes (SMDs)

The summary SMDs and corresponding 95% CIs for all outcomes are presented in Table 1. Detailed forest plots illustrating individual and pooled effects are provided in Supplemental Figure 2, whereas funnel plots assessing potential publication bias are included in Supplemental Figure 3. For cardiovascular health, compared with higher-carbohydrate diets, CRDs significantly reduced blood pressure: SBP (–2.05 mmHg) and DBP (–1.26 mmHg); lipid profiles: TG (–15.11 mg/dL), LDL–HDL ratio (–0.18), TG-HDL ratio (–0.32), VLDL (–3.34 mg/dL), and ApoB-ApoA1 ratio (–0.03); and other endothelial and inflammatory markers: E-selectin (–2.23 ng/mL), CRP (–0.37 mg/L), and TNF-α (–0.27 pg/mL). Conversely, increases were observed in TC (+4.32 mg/dL), LDL (+4.81 mg/dL), HDL (+2.92 mg/dL), and ApoA1 (+0.04 g/L). The overall CVD risk was decreased by 1.05% (95% CI: –1.89, –0.21).

**TABLE 1:** The pooled effect sizes (SMD) of outcomes.

	*N* of obs.	SMD (95% CI)	*P*	*I*^2^ (%)
Cardiovascular health				
Blood pressure				
SBP (mmHg)	88	–2.05 (–3.13, –0.96)	<0.001	99.91
DBP (mmHg)	89	–1.26 (–1.94, –0.57)	<0.001	99.64
Lipid profile				
TG (mg/dL)	143	–15.11 (–18.76, –11.48)	<0.001	99.24
TC (mg/dL)	128	4.32 (1.66, 6.97)	<0.001	99.70
LDL (mg/dL)	141	4.81 (2.58, 7.05)	<0.001	99.66
HDL (mg/dL)	142	2.92 (2.10, 3.74)	<0.001	99.79
Non-HDL (mg/dL)	12	1.91 (–4.66, 8.48)	0.57	99.91
LDL–HDL ratio	8	–0.18 (–0.31, –0.05)	<0.05	92.69
TC-HDL ratio	32	–0.13 (–0.26, 0.01)	0.06	99.47
TG-HDL ratio	16	–0.32 (–0.44, –0.20)	<0.001	90.83
VLDL (mg/dL)	10	–3.34 (–6.03, –0.66)	<0.05	96.39
ApoA1 (g/L)	16	0.04 (0.00, 0.07)	<0.05	94.90
ApoB (g/L)	20	0.02 (–0.01, 0.06)	0.18	99.72
ApoB-ApoA1 ratio	6	–0.03 (–0.04, –0.02)	<0.001	71.76
Endothelial functions				
FMD (%)	7	0.11 (–0.41, 0.63)	0.68	92.68
E-selectin (ng/mL)	8	–2.23 (–4.09, –0.36)	<0.05	96.11
sICAM-1 (ng/mL)	7	–12.60 (–28.93, 3.72)	0.13	96.52
sVCAM-1 (ng/mL)	6	–1.42 (–20.84, 18.00)	0.89	95.97
Inflammatory markers				
CRP (mg/L)	54	–0.37 (–0.69, –0.05)	<0.05	99.12
TNF-α (pg/mL)	10	–0.27 (–0.42, –0.11)	<0.001	83.56
IL-6 (pg/mL)	10	–0.21 (–0.67, 0.24)	0.36	91.60
CVD risk (%)	4	–1.05 (–1.89, –0.21)	<0.05	99.75
Body composition				
BW (kg)	135	–1.75 (–2.20, –1.31)	<0.001	99.56
BMI	93	–0.72 (–0.93, –0.51)	<0.001	99.01
FM (kg)	66	–0.84 (–1.19, –0.49)	<0.001	95.59
BFP	48	–0.78 (–1.09, –0.47)	<0.001	98.36
LM (kg)	31	–0.30 (–0.59, –0.01)	<0.05	98.33
FFM (kg)	27	–0.41 (–0.57, –0.25)	<0.001	70.72
WC (cm)	84	–1.86 (–2.48, –1.24)	<0.001	99.66
HC (cm)	18	–1.65 (–2.50, –0.79)	<0.001	95.97
WHR	23	–0.01 (–0.02, –0.00)	<0.05	93.71
VAT (kg)	6	–0.28 (–0.50, –0.05)	<0.05	98.48

Restricted maximum likelihood (REML) was used for conducting the analyses.

*N* of Obs. refers to the number of observations included for the associated outcomes.

Abbreviations: ApoA1, Apolipoprotein A-I; ApoB, Apolipoprotein B, BFP, body fat percentage; BW, body weight (kg); CRP, C-reactive protein; CVD risk%, cardiovascular disease risk (percentage); DBP, diastolic blood pressure; FFM, fat-free mass (kg); FM, fat mass (kg); FMD%, flow-mediated dilation (percentage); HC, hip circumference (cm); LM, lean mass (kg); SBP, systolic blood pressure; sICAM-1, soluble intercellular adhesion molecule-1; SMD (95% CI), standardized mean difference and the corresponding 95% confidence interval; sVCAM-1, soluble vascular cell adhesion molecule-1; TC, total cholesterol; TG, triglycerides; VAT, visceral adipose tissue; WC, waist circumference (cm); WHR, waist–hip ratio.

For body composition, compared with individuals with higher-carbohydrate intakes, those with CRDs had lowered levels of all measured anthropometric metrics (Table 1 for detailed information on reduced anthropometric metrics and their associated statistical indices). Lastly, excluding high-risk studies and outliers confirmed the robustness of results, except for VLDL and LM, which became nonsignificant (Supplemental Table 2).

### Subgroup analyses: macronutrient composition

Subgroup analyses were conducted to evaluate the differential effects of various CRD types and macronutrient replacements. Results are summarized in Table 2.

**TABLE 2:** Subgroup analysis of diet characteristics (types and replacements).

	KD		LCD		MCD	
	SMD (95% CI)	*P*	SMD (95% CI)	*P*	SMD (95% CI)	*P*
Blood pressure						
SBP (16 vs. 23 vs. 36)^1^	–1.10 (–3.63, 1.43)	0.39	–2.73 (–4.46, –1.00)	<0.001^2^	–2.18 (–4.14, –0.22)	<0.05
DBP (16 vs. 25 vs. 36)^1^	1.14 (–0.74, 3.02)	0.23	–1.23 (–2.36, –0.10)	<0.05	–2.39 (–3.41, –1.36)	<0.001^2^
Lipid profile						
TG (30 vs. 29 vs. 63)^1^	–15.73 (–24.59, –6.88)	<0.001^2^	–15.48 (–22.28, –8.67)	<0.001^2^	–15.54 (–21.41, –9.67)	<0.001^2^
TC (27 vs. 27 vs. 55)^1^	13.80 (6.67, 20.93)	<0.001^2^	7.46 (0.70, 14.23)	<0.05	–0.20 (–2.98, 2.58)	0.89
LDL (29 vs. 33 vs. 59)^1^	13.34 (7.68, 19.01)	<0.001^2^	7.30 (1.75, 12.84)	<0.05	0.29 (–2.28, 2.86)	0.82
HDL (30 vs. 33 vs. 59)^1^	7.30 (3.53, 11.07)	<0.001^2^	2.92 (1.04, 4.81)	<0.001^2^	1.86 (1.00, 2.72)	<0.001^2^
Non-HDL (2 vs. 3 vs. 4)^1^	19.93^3^ (–0.90, 40.76)	0.06	2.98^3^ (–6.23, 12.19)	0.53	–5.82 (–14.72, 3.08)	0.20
LDL–HDL (0 vs. 2 vs. 5)^1^	—		–0.40^3^ (–0.42, –0.38)	<0.001	–0.14 (–0.26, –0.02)	<0.05
TC-HDL (8 vs. 7 vs. 12)^1^	0.03 (–0.20, 0.26)	0.79	–0.07 (–0.30, 0.15)	0.53	–0.32 (–0.62, –0.01)	<0.05
TG-HDL (7 vs. 3 vs. 3)^1^	–0.30 (–0.45, –0.16)	<0.001	–0.14^3^ (–0.25, –0.02)	<0.05	–0.55^3^ (–0.95, –0.15)	<0.05
VLDL (3 vs. 0 vs. 5)^1^	–1.50^3^ (–5.99, 2.98)	0.51	—		–7.58 (–9.60, –5.57)	<0.001^2^
ApoA-1 (1 vs. 2 vs. 8)^1^	0.20^3^ (0.15, 0.25)	<0.001	0.02^3^ (–0.01, 0.05)	0.29	0.01 (–0.03, 0.05)	0.48
ApoB (5 vs. 3 vs. 9)^1^	0.11 (0.02, 0.20)	<0.05	0.03^3^ (–0.07, 0.13)	0.52	–0.02 (–0.04, 0.00)	0.10
ApoB-ApoA1 (0 vs. 1 vs. 3)^1^	—		–0.05^3^ (–0.05, –0.05)	<0.001	–0.02^3^ (–0.04, –0.01)	<0.001
Endothelial functions						
FMD, % (1 vs. 5 vs. 1)^1^	–1.00^3^ (–3.91, 1.91)	0.50	–0.04 (–0.54, 0.46)	0.87	0.90^3^ (0.30, 1.50)	<0.001
E-selectin (2 vs. 1 vs. 4)^1^	–0.56^3^ (–2.67, 1.55)	0.60	–7.70^3^ (–13.93, –1.47)	<0.05	–2.62 (–5.02, –0.22)	<0.05
sICAM-1 (2 vs. 1 vs. 4)^1^	–12.50^3^ (–16.14, –8.86)	<0.001	–22.70^3^ (–70.11, 24.71)	0.35	–8.68 (–34.86, 17.50)	0.52
sVCAM-1 (1 vs. 1 vs. 4)^1^	–31.20^3^ (–48.91, –13.49)	<0.001	–25^3^ (–210.4, 160.4)	0.79	5.26 (–4.28,14.80)	0.28
Inflammatory markers						
CRP (12 vs. 14 vs. 20)^1^	–0.47 (–1.19, 0.26)	0.21	–0.13 (–0.63, 0.30)	0.60	–0.63 (–1.46, 0.21)	0.14
TNF-α (1 vs. 4 vs. 5)^1^	–0.10^3^ (–0.27, 0.07)	0.25	–0.11 (–0.19, –0.04)	<0.001^2^	–0.41 (–0.64, –0.17)	<0.001^2^
IL-6 (2 vs. 4 vs. 3)^1^	–0.41^3^ (–1.00, 0.19)	0.18	0.19 (–0.35, 0.73)	0.50	–0.58^3^ (–1.69, 0.53)	0.31
Body composition						
BW (28 vs. 31 vs. 56)^1^	–3.36 (–4.80, –1.92)	<0.001^2^	–1.97 (–2.75, –1.15)	<0.001^2^	–0.77 (–1.26, –0.29)	<0.001^2^
BMI (23 vs. 22 vs. 37)^1^	–1.24 (–1.82, –0.65)	<0.001^2^	–0.87 (–1.37, –0.37)	<0.001^2^	–0.36 (–0.52, –0.20)	<0.001^2^
FM (15 vs. 13 vs. 29)^1^	–0.93 (–1.72, –0.14)	<0.05	–0.92 (–1.73, –0.12)	<0.05	–0.71 (–1.25, –0.16)	<0.05
BFP (9 vs. 13 vs. 23)^1^	–1.15 (–2.31, 0.00)	0.05	–0.47 (–1.07, 0.13)	0.13	–0.90 (–1.28, –0.51)	<0.001^2^
LM (8 vs. 6 vs. 15)^1^	–0.72 (–1.39, –0.06)	<0.001^2^	–0.56 (–1.56, 0.43)	0.27	–0.08 (–0.35, 0.20)	0.59
FFM (6 vs. 8 vs. 9)^1^	–0.71 (–1.22, –0.21)	<0.05	–0.29 (–0.50, –0.09)	<0.001^2^	–0.37 (–0.79, 0.04)	0.08
WC (14 vs. 23 vs. 38)^1^	–4.88 (–7.35, –2.41)	<0.001^2^	–2.08 (–2.87, –1.28)	<0.001^2^	–0.89 (–1.53, –0.26)	<0.05
HC (3 vs. 5 vs. 8)^1^	–0.91^3^ (–2.37, 0.55)	0.22	–2.96 (–4.86, –1.07)	<0.001^2^	–0.95 (–2.02, 0.13)	0.08
WHR (4 vs. 5 vs. 12)^1^	–0.02 (–0.03, –0.02)	<0.001^2^	–0.00 (–0.02, 0.01)	0.61	–0.01 (–0.02, 0.00)	0.14

	Fat	Protein	Both			
	SMD	*P*	SMD	*P*	SMD	*P*

Blood pressure						
SBP (21 vs. 9 vs. 58)^4^	–1.01 (–3.08, 1.07)	0.34	–1.29 (–3.28, 0.70)	0.20	–2.52 (–3.95, –1.09)	<0.001^2^
DBP (19 vs. 11 vs. 59)^4^	–0.19 (–1.59, 1.22)	0.80	–0.49 (–1.39, 0.40)	0.28	–1.70 (–2.60, –0.80)	<0.001^2^
Lipid profile						
TG (46 vs. 18 vs. 79)^4^	–12.24 (–19.24, –5.24)	<0.001^2^	–12.92 (–23.72, –2.11)	<0.05	–17.11 (–21.7, –12.54)	<0.001^2^
TC (41 vs. 17 vs. 70)^4^	5.68 (1.12, 10.24)	<0.05	–4.29 (–7.28, –1.30)	<0.001^2^	5.78 (1.92, 9.64)	<0.001^2^
LDL (43 vs. 17 vs. 81)^4^	6.39 (2.95, 9.84)	<0.001^2^	–6.55 (–13.07, –0.02)	0.05	6.49 (3.59, 9.39)	<0.001^2^
HDL (46 vs. 17 vs. 79)^4^	3.56 (2.11, 5.02)	<0.001^2^	0.47 (–1.27, 2.21)	0.60	3.14 (1.99, 4.29)	<0.001^2^
Non-HDL (3 vs. 2 vs. 7)^4^	1.56^3^ (–4.43, 7.55)	0.61	–5.20^3^ (–7.36, –3.04)	<0.001	4.16 (–7.22. 15.54)	0.47
LDL–HDL (4 vs. 0 vs. 4)^4^	0.00 (–0.06, 0.06)	0.97	—		–0.29 (–0.40, –0.18)	<0.001^2^
TC-HDL (12 vs. 1 vs. 19)^4^	–0.10 (–0.19, –0.01)	<0.05	0.10^3^ (0.00, 0.20)	0.05	–0.13 (–0.34, 0.08)	0.22
TG-HDL (2 vs. 0 vs. 14)^4^	–0.33^3^ (–0.43, –0.24)	<0.001	—		–0.32 (–0.47, –0.17)	<0.001^2^
VLDL (6 vs. 1 vs. 3)^4^	–3.66 (–7.54, 0.22)	0.06	–7.40^3^ (–9.57, –5.23)	<0.001	–1.50^3^ (–5.99, 2.98)	0.51
ApoA1 (7 vs. 0 vs. 7)^4^	0.05 (0.01, 0.08)	<0.05	—		0.03 (–0.03, 0.09)	0.32
ApoB (8 vs. 0 vs. 12)^4^	0.02 (–0.02, 0.07)	0.25	—		0.02 (–0.03, 0.07)	0.42
ApoB-ApoA1(2 vs. 0 vs. 4)^4^	–0.02^3^ (–0.03, –0.01)	<0.05	—		–0.04 (–0.06, –0.02)	<0.001^2^
Endothelial functions						
FMD, % (1 vs. 0 vs. 6)^4^	–0.56^3^ (–0.77, –0.35)	<0.001	—		0.31 (–0.14, 0.76)	0.18
e-Selectin (3 vs. 1 vs. 4)^4^	0.00^3^ (–0.41, 0.41)	0.99	–4.90^3^ (–5.40, –4.40)	<0.001	–2.70 (–5.04, –0.36)	<0.05
sICAM-1 (2 vs. 1 vs. 4)^4^	–6.92^3^ (–28.51, 14.67)	0.53	15.60^3^ (–1.86, 33.06)	0.08	–23.24 (–40.46, –6.03)	<0.01
sVCAM-1 (1 vs. 0 vs. 5)^4^	73.64^3^ (–8.68, 155.96)	0.08	—		–4.95 (–23.69, 13.78)	0.60
Inflammatory markers						
CRP (13 vs. 7 vs. 34)^4^	0.13 (–0.33, 0.58)	0.59	–2.17 (–5.32, 0.96)	0.18	–0.38 (–0.72, –0.04)	<0.05
TNF-α (2 vs. 1 vs. 7)^4^	–0.11^3^ (–0.18, –0.04)	<0.001	–0.90^3^ (–3.07, 1.27)	0.42	–0.34 (–0.54, –0.15)	<0.001^2^
IL-6 (4 vs. 1 vs. 5)^4^	–0.10 (–0.33, 0.13)	0.41	–0.90^3^ (–3.55, 1.75)	0.51	–0.06 (–0.88, 0.76)	0.89
CVD risk, % (0 vs. 0 vs. 4)^4^	—		—		–1.05 (–1.89, –0.21)	<0.05
Body composition						
BW (45 vs. 12 vs. 78)^4^	–1.14 (–2.10, –0.19)	<0.05	–1.64 (–3.48, 0.20)	0.08	–2.01 (–2.46, –1.56)	<0.001^2^
BMI (29 vs. 7 vs. 57)^4^	–0.71 (–1.18, –0.23)	<0.001^2^	–1.11 (–2.04, –0.18)	<0.05	–0.65 (–0.85, –0.45)	<0.001^2^
FM (25 vs. 10 vs. 31)^4^	–0.78 (–1.29, –0.26)	<0.001^2^	–1.29 (–2.41, –0.17)	<0.05	–0.73 (–1.23, –0.23)	<0.001^2^
BFP (17 vs. 3 vs. 28)^4^	–0.39 (–0.77, –0.01)	<0.05	–1.74^3^ (–3.43, –0.05)	<0.05	–0.90 (–1.32, –0.68)	<0.001^2^
LM (14 vs. 6 vs. 11)^4^	–0.49 (–1.12, 0.15)	0.13	–0.30 (–0.58, –0.01)	0.10	–0.15 (–0.52, 0.22)	0.43
FFM (10 vs. 1 vs. 16)^4^	–0.37 (–0.59, –0.15)	<0.001^2^	1.10^3^ (–0.50, 2.70)	0.18	–0.48 (–0.74, –0.23)	<0.001^2^
WC (27 vs. 6 vs. 51)^4^	–1.87 (–3.30, –0.45)	<0.05	–2.52 (–5.46, 0.43)	0.09	–1.73 (–2.34, –1.12)	<0.001^2^
HC (5 vs. 0 vs. 13)^4^	–0.62 (–1.66, 0.42)	0.24	—		–2.00 (–3.02, –0.98)	<0.001^2^
WHR (7 vs. 0 vs. 16)^4^	–0.01 (–0.02, –0.01)	<0.001^2^	—		–0.01 (–0.02, 0.00)	0.30
VAT (2 vs. 0 vs. 4)^4^	–0.22^3^ (–0.56, 0.11)	0.19	—		–0.30 (–0.62, 0.02)	0.06

Restricted maximum likelihood (REML) was used for conducting the analyses.

Abbreviations: 95% CI, 95% confidence interval; ApoA1, Apolipoprotein A-I; ApoB, Apolipoprotein B; BFP, body fat percentage; CRP, C-reactive protein; CVD risk%, cardiovascular disease risk (percentage). BW, body weight (kg); DBP, diastolic blood pressure; FFM, fat-free mass (kg); FM, fat mass (kg); FMD%, flow-mediated dilation (percentage); HC, hip circumference (cm); KD, ketogenic diet: ≤10% of total calories or 20–50 g/d; LCD, low-carb diet: 10%–26% of total calories or 50–130 g/d; LM, lean mass (kg); MCD, moderate-carb diet: 26%–45% of total calories or 130–230 g/d; SBP, systolic blood pressure; sICAM-1, soluble intercellular adhesion molecule-1; SMD, standardized mean difference; sVCAM-1, soluble vascular cell adhesion molecule-1; TC, total cholesterol; TG, triglycerides; VAT, visceral adipose tissue; WC, waist circumference (cm); WHR, waist–hip ratio.

1 (*k* vs. *k* vs. *k*) stands for the number of observations included for each associated group: KD vs. LCD vs. MCD.

2 Represents statistical significance after adjusted for multiple testing correction (*P* < 0.00125) among analyses included >4 studies (*k* ≥ 4).

3 Represents the number analyzed by fewer than 4 observations for the particular group.

4 (*k* vs. *k* vs. *k*) stands for the number of observations included for each associated group: fat vs. protein vs. combination.

#### Effects of CRD types on cardiovascular and anthropometric outcomes

Among analyses with >4 studies and adjustment for multiple testing (corrected alpha: *P* < 0.0025), all 3 CRDs demonstrated significant cardiovascular benefits, including reduced TG and increased HDL cholesterol. Additionally, both LCD and MCD were associated with improvements in blood pressure and reductions in TNF-α. In contrast, KD increased LDL and TC but reduced the TG:HDL ratio by –0.27 (95% CI: –0.45, –0.16). Regarding body composition, all 3 CRDs led to reductions in BW and BMI, with varying effects on BFP, FFM, HC, WC, and WHR (Table 2 for details).

#### Effects of carbohydrate replacement strategies

When comparing carbohydrate replacements (fat, protein, and combination), analyses with >4 studies (corrected alpha: *P* < 0.0025) indicated that combined replacement (fat and protein) exerted the broadest cardiovascular benefits. This approach significantly influenced blood pressure, lipid profiles, and TNF-α levels. In addition, nearly all anthropometric measures—except LM, WHR, and VAT were improved (Table 2 for details).

#### Sensitivity analysis results

Sensitivity analyses revealed minor changes in statistical significance, including attenuated reductions in systolic and diastolic blood pressure for MCD. The majority of outcomes remained robust, supporting the stability and reliability of the primary findings (Supplemental Table 3).

### Metaregression on intervention length

As presented in Table 3, longer durations were associated with greater reductions in LDL–HDL ratio (*β* = –0.03, *P* < 0.001), ApoB-ApoA1 (*β* = –0.001, *P* < 0.001), and IL-6 (*β* = –0.03, *P* < 0.001). The sensitivity analysis confirmed the robustness of the original findings (Supplemental Table 4).

**TABLE 3:** Metaregression of intervention length (in weeks).

	*b*-coefficient	SE	*P*
Blood pressure			
SBP	0.02	0.02	0.30
DBP	–0.01	0.01	0.28
Lipid profile			
TG	–0.04	0.08	0.67
TC	–0.09	0.06	0.11
LDL	–0.08	0.05	0.08
HDL	–0.01	0.02	0.61
Non-HDL	0.08	0.21	0.69
LDL–HDL	–0.02	0.003	<0.001^1^
TC-HDL	0.003	0.004	0.34
TG-HDL	–0.002	0.003	0.55
VLDL	–0.34	0.39	0.39
ApoA-1	–0.001	0.001	0.45
ApoB	–0.001	0.001	0.47
ApoB-ApoA1	–0.001	0.0003	<0.001^1^
Endothelial functions			
FMD, %	–0.004	0.008	0.68
E-selectin	–0.02	0.05	0.65
sICAM-1	–0.03	0.45	0.94
sVCAM-1	–0.55	0.63	0.38
Inflammatory markers			
CRP	0.01	0.01	0.25
TNF-α	–0.01	0.003	0.06
IL-6	–0.03	0.004	<0.001^1^
CVD risk, %	0.02	0.02	0.31
Body composition			
BW	–0.002	0.01	0.87
BMI	0.002	0.004	0.61
FM	0.01	0.008	0.13
BFP	–0.002	0.006	0.72
LM	–0.01	0.007	0.13
FFM	0.002	0.003	0.37
WC	0.02	0.01	0.15
HC	–0.02	0.02	0.40
WHR	0.0001	0.0002	0.65
VAT	0.003	0.003	0.43

Restricted maximum likelihood (REML) was used for conducting the analyses.

Abbreviations: ApoA1, Apolipoprotein A-I; ApoB, Apolipoprotein B; BFP, body fat percentage; BW, body weight (kg); CRP, C-reactive protein; CVD risk%, cardiovascular disease risk (percentage); DBP, diastolic blood pressure; FFM, fat-free mass (kg); FM, fat mass (kg); FMD%, flow-mediated dilation (percentage); HC, hip circumference (cm); LM, lean mass (kg); SBP, systolic blood pressure; sICAM-1, soluble intercellular adhesion molecule-1; sVCAM-1, soluble vascular cell adhesion molecule-1; TC, total cholesterol; TG, triglycerides; VAT, visceral adipose tissue; WC, waist circumference (cm); WHR, waist–hip ratio.

1 Represents statistical significance after adjusted for multiple testing correction (*P* < 0.00125) among analyses included >4 studies (*k* ≥ 4).

### Subgroup analyses of participants’ characteristics

Subgroup analyses based on participant characteristics were conducted to explore differential effects (Table 4).

**TABLE 4:** Subgroup analysis of population characteristics (sex, weight, and diabetic status).

	Male		Female		Diff.	
	SMD (95% CI)	*P*	SMD (95% CI)	*P*	Qb(1)	*P*
Blood pressure						
SBP (3 vs. 17)^1^	–5.34^2^ (–12.28, 1.60)	0.13	–2.10 (–5.66, 1.45)	0.25	0.66	0.42
DBP (4 vs. 20)^1^	–0.28 (–5.07, 4.50)	0.91	–1.41 (–2.77, –0.04)	<0.05	0.20	0.66
Lipid profile						
TG (11 vs. 29)^1^	–29.20 (–48.55, –11.88)	<0.001^3^	–14.47 (–23.34, –5.81)	<0.001^3^	2.20	0.14
TC (12 vs. 27)^1^	9.30 (–2.98, 21.58)	0.14	2.56 (–2.81, 7.93)	0.35	0.97	0.32
LDL (12 vs. 27)^1^	6.76 (–7.50, 21.02)	0.35	3.99 (–0.05, 8.02)	0.05	0.13	0.71
HDL (11 vs. 27)^1^	1.77 (–0.97, 4.50)	0.21	3.04 (1.27, 4.81)	<0.001^3^	0.59	0.44
TC-HDL (6 vs. 8)^1^	0.02 (–0.17, 0.21)	0.82	–0.17 (–0.30, –0.04)	<0.05	2.73	0.10
Inflammatory markers						
CRP (2 vs. 10)^1^	–2.23^2^ (–5.09, 0.63)	0.13	–1.76 (–3.80, 0.28)	0.09	0.07	0.79
Body composition						
BW (7 vs. 26)^1^	–1.08 (–3.16, 1.01)	0.31	–1.91 (–3.30, –0.51)	<0.05	0.42	0.52
BMI (9 vs. 22)^1^	–0.53 (–0.96, –0.10)	<0.05	–0.90 (–1.40, –0.40)	<0.001^3^	1.23	0.27
FM (6 vs. 14)^1^	–1.44 (–3.12, 0.23)	0.09	–1.40 (–2.11, –0.70)	<0.001^3^	0.00	0.96
BFP (6 vs. 11)^1^	–1.09 (–2.45, 0.27)	0.12	–1.17 (–1.86, –0.48)	<.001^3^	0.01	0.92
LM (3 vs. 11)^1^	0.11^2^ (–0.32, 0.53)	0.62	–0.17 (–0.50, 0.16)	0.31	1.03	0.31
FFM (4 vs. 6)^1^	–0.47 (–1.17, 0.23)	0.19	–0.60 (–0.77, –0.43)	<0.001^3^	0.11	0.74
WC (6 vs. 16)^1^	–1.77 (–5.04, 1.49)	0.29	–3.13 (–5.11, –1.16)	<0.001^3^	0.49	0.49
HC (0 vs. 6)^1^	—		–1.31 (–3.07, 0.45)	0.14	—	
WHR (0 vs. 10)^1^	—		–0.01 (–0.02, –0.01)	<0.001^3^	—	

	Nonoverweight		Overweight/obese		Diff.	
	SMD (95% CI)	*P*	SMD (95% CI)	*P*	Qb(1)	*P*

Blood pressure						
SBP (2 vs. 71)^4^	–2.86^2^ (–8.94, 3.21)	0.36	–1.74 (–3.01, –0.56)	<0.001^3^	0.13	0.72
DBP (2 vs. 70)^4^	4.06^2^ (–3.58, 11.71)	0.30	–1.15 (–1.89, –0.42)	<0.001^3^	10.77	0.18
Lipid profile						
TG (5 vs. 114)^4^	–8.01 (–28.16, 12.13)	0.44	–13.70 (–17.72, –9.67)	<0.001^3^	0.29	0.59
TC (5 vs. 100)^4^	10.66 (–6.47, 27.80)	0.22	4.21 (1.21, 7.29)	<0.05	0.53	0.47
LDL (5 vs. 111)^4^	9.44 (–3.10, 21.98)	0.14	5.06 (2.56, 7.71)	<0.001^3^	0.45	0.50
HDL (5 vs. 112)^4^	3.70 (–0.20, 7.60)	0.06	2.72 (1.81, 3.54)	<0.001^3^	0.23	0.63
Non-HDL (2 vs. 7)^4^	18.17^2^ (–6.07, 42.41)	0.14	2.57 (–2.61, 7.74)	0.33	1.52	0.22
LDL–HDL (1 vs.5)^4^	0.01^2^ (–0.05, 0.07)	0.76	–0.27 (–0.39, –0.16)	<0.001^3^	17.33	<0.001
TC-HDL (1 vs. 24)^4^	–0.05^2^ (–0.06, –0.04)	<0.001	–0.11 (–0.27, 0.08)	0.19	0.56	0.46
TG-HDL (0 vs. 14)^4^	—		–0.34 (–0.46, –0.21)	<0.001^3^	—	
ApoA1 (1 vs. 9)^4^	0.20^2^ (0.15, 0.25)	<0.001	0.02 (0.00, 0.05)	<0.05	38.12	<0.001
ApoB (2 vs. 13)^4^	0.11^2^ (–0.05, 0.28)	0.18	0.01 (–0.02, 0.04)	0.54	1.40	0.24
ApoB-ApoA1 (0 vs. 5)^4^	—		–0.03 (–0.04, –0.01)	<0.001^3^	—	
Endothelial functions						
FMD, % (0 vs. 5)^4^	—		0.26 (–0.22, 0.75)	0.29	—	
E-selectin (0 vs. 7)^4^	—		–1.89 (–3.74, –0.04)	0.05	—	
sICAM-1 (0 vs. 6)^4^	—		–11.76 (–29.56, 6.04)	0.20	—	
sVCAM-1 (0 vs. 5)^4^	—		–1.11 (–20.89, 18.67)	0.91	—	
Inflammatory markers						
CRP (1 vs. 45)^4^	–0.80^2^ (–1.37, –0.23)	<0.05	–0.31 (–0.54, –0.02)	<0.05	2.32	0.13
TNF-α (1 vs. 7)^4^	–0.11^2^ (–0.19, –0.03)	<0.05	–0.29 (–0.49, –0.10)	<0.001^3^	2.98	0.08
IL-6 (1 vs. 7)^4^	–0.34^2^ (–1.54, 0.86)	0.58	–0.31 (–0.93, 0.30)	0.32	0.00	0.97
Body composition						
BW (3 vs. 104)^4^	0.19 (–0.44, 0.82)	0.56	–1.90 (–2.45, –1.40)	<0.001^3^	24.81	<0.001^3^
BMI (4 vs. 74)^4^	–0.30 (–0.84, 0.23)	0.27	–0.78 (–0.98, –0.52)	<0.001^3^	31.45	<0.001^3^
FM (2 vs. 56)^4^	0.19^2^ (0.00, 0.97)	0.64	–0.75 (–1.11, –0.38)	<0.001^3^	4.51	<0.05
BFP (1 vs. 40)^4^	–0.30^2^ (–0.82, 0.22)	0.25	–0.70 (–1.03, –0.37)	<0.001^3^	1.65	0.20
LM (0 vs. 24)^4^	—		–0.24 (–0.53, 0.05)	0.11	—	
FFM (1 vs. 24)^4^	0^2^	1.00	–0.43 (–0.60, –0.25)	<0.001^3^	1.60	0.21
WC (2 vs. 68)^4^	–0.77^2^ (–3.90, 2.37)	0.63	–1.96 (–2.74, –1.31)	<0.001^3^	0.53	0.47
HC (0 vs. 13)^4^	—		–1.70 (–2.78, –0.62)	<0.001^3^	—	
WHR (0 vs. 20)^4^	—		–0.01 (–0.02, 0.00)	<0.05	—	
VAT (0 vs. 4)^4^	—		–0.19 (–0.31, –0.07)	<0.001^3^	—	

	Nondiabetic		T2D		Diff.	
	SMD (95% CI)	*P*	SMD (95% CI)	*P*	Qb(1)	*P*

Blood pressure						
SBP (41 vs. 23)^5^	–2.02 (–3.03, –1.02)	<0.001^3^	–1.56 (–3.79, 0.68)	0.17	0.08	0.77
DBP (44 vs. 20)^5^	–1.05 (–2.02, –0.10)	<0.05	–1.41 (–3.00, 0.19)	0.08	0.14	0.71
Lipid profile						
TG (73 vs. 35)^5^	–14.17 (–19.29, –9.07)	<0.001^3^	–14.15 (–22.17, –6.14)	<0.001^3^	0.00	1.00
TC (67 vs. 27)^5^	4.41 (0.62, 8.33)	<0.05	0.09 (–3.19, 3.38)	0.96	2.84	0.09
LDL (72 vs. 32)^5^	5.44 (2.34, 8.77)	<0.001^3^	1.27 (–1.77, 4.32)	0.41	3.45	0.06
HDL (72 vs. 33)^5^	2.79 (1.77, 3.66)	<0.001^3^	4.03 (1.28, 6.79)	<0.001^3^	0.70	0.40
Non-HDL (7 vs. 4)^5^	6.83 (–2.04, 15.69)	0.13	–5.87 (–14.17, 2.43)	0.17	4.20	<0.05
LDL–HDL (5 vs. 1)^5^	–0.15 (–0.41, 0.11)	0.26	–0.09^2^ (–0.28, 0.10)	0.36	0.13	0.72
TC-HDL (20 vs. 5)^5^	–0.14 (–0.33, 0.09)	0.17	–0.20 (–0.40, 0.00)	0.05	0.18	0.67
TG-HDL (8 vs. 1)^5^	–0.30 (–0.53, –0.08)	<0.05	–0.60^2^ (–0.65, –0.55)	<0.001	6.18	<0.05
VLDL (4 vs. 4)^5^	–4.56 (–8.58, –0.55)	<0.05	–5.44 (–10.61, –0.26)	<0.05	0.07	0.79
ApoA1 (8 vs. 6)^5^	0.06 (0.02, 0.11)	<0.05	0.00 (–0.04, 0.05)	0.95	3.44	0.06
ApoB (13 vs. 7)^5^	0.04 (0.00, 0.09)	0.06	–0.01 (–0.06, 0.04)	0.70	2.51	0.11
Endothelial functions						
E-selectin (6 vs. 1)^5^	–1.85 (–3.70, 0.01)	0.05	–11.00^2^ (–39.26, 17.26)	0.45	0.40	0.53
sICAM-1 (5 vs. 1)^5^	–9.87 (–28.97, 9.22)	0.31	–35.00^2^ (–90.02, 20.02)	0.21	0.71	0.40
sVCAM-1 (4 vs. 1)^5^	0.93 (–30.01, 31.88)	0.95	–0.30^2^ (–0.75, 0.15)	0.19	0.01	0.94
Inflammatory markers						
CRP (32 vs. 12)^5^	–0.73 (–1.19, –0.20)	<0.001^3^	0.10 (–0.44, 0.64)	0.72	5.07	<0.05
TNF-α (9 vs. 0)^5^	–0.27 (–0.43, –0.11)	<0.001^3^	—		—	
IL-6 (8 vs. 0)^5^	–0.24 (–0.74, 0.26)	0.36	—		—	
Body composition						
BW (63 vs. 34)^5^	–1.41 (–1.93, –0.88)	<0.001^3^	–2.03 (–3.01, –1.05)	<0.001^3^	1.20	0.27
BMI (41 vs. 25)^5^	–0.61 (–0.80, –0.43)	<0.001^3^	–0.99 (–1.49, –0.48)	<0.001^3^	1.66	0.17
FM (38 vs. 9)^5^	–0.93 (–1.48, –0.40)	<0.001^3^	–0.57 (–1.18, 0.05)	0.07	0.75	0.38
BFP (28 vs. 8)^5^	–0.79 (–1.23, –0.35)	<0.001^3^	–0.65 (–1.27, –0.03)	<0.05	0.13	0.72
LM (21 vs. 2)^5^	–0.33 (–0.67, 0.00)	0.05	–1.32^2^ (–3.82, 1.18)	0.30	0.59	0.44
FFM (10 vs. 5)^5^	–0.52 (–0.78, –0.27)	<0.001^3^	–0.18 (–0.28, –0.08)	<0.001^3^	6.14	<0.05
WC (40 vs. 22)^5^	–1.54 (–2.23, –0.86)	<0.001^3^	–2.34 (–3.59, –1.10)	<0.001^3^	1.21	0.27
HC (10 vs. 5)^5^	–1.50 (–2.56, –0.43)	<0.05	–2.60 (–4.35, –0.86)	<0.001^3^	1.12	0.29
WHR (16 vs. 1)^5^	–0.01 (–0.01, 0.00)	0.16	–0.01^2^ (–0.04, 0.02)	0.48	0.07	0.80
VAT (4 vs. 1)^5^	–0.37 (–0.67, –0.06)	<0.05	–0.14^2^ (–0.18, –0.10)	<0.001	2.12	0.15

Restricted maximum likelihood (REML) was used for conducting the analyses.

Abbreviations: 95% CI, 95% confidence interval; ApoA1, Apolipoprotein A-I; ApoB, Apolipoprotein B; BFP, body fat percentage; BW, body weight (kg); CRP, C-reactive protein; CVD risk%, cardiovascular disease risk (percentage); DBP, diastolic blood pressure; Diff., group difference; FFM, fat-free mass (kg); FM, fat mass (kg); FMD%, flow-mediated dilation (percentage); HC, hip circumference (cm); LM, lean mass (kg); Q(b), Cochran’s Q-between statistics; SBP, systolic blood pressure; sICAM-1, soluble intercellular adhesion molecule-1; SMD, standardized mean difference; sVCAM-1, soluble vascular cell adhesion molecule-1; T2D, type 2 diabetes; TC, total cholesterol; TG, triglycerides; VAT, visceral adipose tissue; WC, waist circumference (cm); WHR, waist–hip ratio.

1 (*k* vs. *k*) stands for the number of observations included for each associated group: male vs. female.

2 Represents the number analyzed by fewer than 4 observations for the particular group.

3 Represents statistical significance after adjusted for multiple testing correction (*P* < 0.00125) among analyses included >4 studies (*k* ≥ 4).

4 (*k* vs. *k*) stands for the number of observations included for each associated group: nonoverweight vs. overweight/obesity.

5 (*k* vs. *k*) stands for the number of observations included for each associated group: nondiabetic vs. T2D.

#### The effects of sex

After adjustment for multiple testing (corrected alpha: *P* < 0.0025), both sexes exhibited significant reductions in TG. However, females demonstrated more pronounced improvements in cardiovascular and anthropometric outcomes, including increased HDL, as well as reductions in BMI, DBP, BFP, WC, and WHR. Despite these trends, formal group comparisons revealed no statistically significant differences between sexes (Table 4).

#### The effects of weight status

Among individuals with overweight/obesity, significant improvements were observed in blood pressure, various lipid profile markers, and all anthropometric measures, except for LM and WHR. In contrast, nonoverweight individuals showed minimal changes. Group comparisons confirmed significantly greater reductions in BW and BMI in the overweight/obesity group (Table 4).

#### The effects of diabetic status

Both nondiabetic and T2DM groups exhibited significant decreases in TG, BW, BMI, FFM, and WC, alongside increased HDL. Although each group displayed unique significant outcomes, none reached statistical significance in direct group comparisons (Table 4).

#### Sensitivity analysis results

Sensitivity analyses revealed additional significant reductions in SBP, CRP, and BW among females, as well as decreases in FM and BFP in individuals with T2DM. Overall, most outcomes remained consistent with the primary analyses, supporting the robustness of the findings (Supplemental Table 5).

### Effects of study characteristics

Analyses with >4 studies (corrected alpha: *P* < 0.0025) revealed differential effects across study designs and intervention characteristics (Table 5). For study designs, although no statistically significant differences emerged between groups, randomized parallel trials demonstrated significant improvements in blood pressure, multiple lipid parameters, and anthropometric measures. In contrast, randomized crossover trials showed effects limited to TG, BMI, BFP, and WC. As for intervention delivery methods, consultation-only interventions produced more pronounced improvements in blood pressure and anthropometric outcomes compared with food-provided interventions. Lastly, when considering energy balance, nonisocaloric and isocaloric interventions showed similar effects on lipid profiles and body composition. However, differential blood pressure effects were observed: isocaloric studies demonstrated significant reductions in systolic blood pressure (SBP), whereas nonisocaloric studies showed decreases in DBP (Table 5). Overall, the sensitivity analyses showed minor variations in statistical significance, but the overall pattern of results remained consistent with the primary analyses (Supplemental Table 6).

**TABLE 5:** Subgroup analysis of study characteristics.

	Crossover		Parallel		Diff.	
	SMD (95% CI)	*P*	SMD (95% CI)	*P*	Qb(1)	*P*
Blood pressure						
SBP (16 vs. 73)^1^	–1.43 (–3.24, 0.37)	0.12	–2.04 (–3.35, –0.85)	<0.001^2^	0.30	0.58
DBP (16 vs. 74)^1^	–1.17 (–2.04, –0.31)	<0.05	–1.24 (–2.05, –0.45)	<0.001^2^	0.01	0.91
Lipid profile						
TG (35 vs. 109)^1^	–18.76 (–26.68, –10.84)	<0.001^2^	–14.01 (–18.76, –11.46)	<0.001^2^	1.10	0.29
TC (34 vs. 95)^1^	4.19 (–2.09, 10.47)	0.19	4.28 (1.42, 7.21)	<0.001^2^	0.00	0.98
LDL (35 vs. 107)^1^	5.32 (1.23, 9.42)	<0.05	4.62 (2.04, 7.33)	<0.001^2^	0.08	0.78
HDL (32 vs. 111)^1^	2.73 (0.75, 4.71)	<0.05	3.00 (2.07, 3.86)	<0.001^2^	0.06	0.81
Non-HDL (5 vs. 7)^1^	–2.48 (–11.97, 7.00)	0.61	5.18 (–3.68, 14.05)	0.25	1.34	0.25
LDL–HDL (4 vs. 4)^1^	–0.09 (–0.26, 0.08)	0.29	–0.21 (–0.38, –0.05)	<0.05	1.01	0.31
TC-HDL (14 vs. 19)^1^	–0.15 (–0.29, –0.01)	<0.05	–0.12 (–0.31, 0.11)	0.22	0.04	0.85
TG-HDL (4 vs. 12)^1^	–0.19 (–0.43, 0.05)	0.11	–0.35 (–0.49, –0.22)	<0.001^2^	1.32	0.25
VLDL (7 vs. 3)^1^	–1.91 (–5.14, 1.32)	0.25	–5.05^3^ (–8.16, –1.95)	<0.001	1.90	0.17
ApoA1 (7 vs. 7)^1^	0.04 (0.00, 0.07)	<0.05	0.04 (–0.02, 0.10)	0.20	0.00	0.96
ApoB (6 vs. 14)^1^	0.01 (–0.05, 0.06)	0.80	0.03 (–0.01, 0.07)	0.19	0.35	0.56
Endothelial functions						
FMD, % (1 vs. 6)^1^	1.00^3^ (–0.64, 2.64)	0.23	0.04 (–0.51, 0.59)	0.89	1.19	0.28
E-selectin (4 vs. 4)^1^	–0.05 (–0.45, 0.36)	0.82	–3.31 (–5.69, –0.94)	<0.05	7.05	<0.05
sICAM-1(3 vs. 4)^1^	–3.30^3^ (–11.54, 4.94)	0.43	–14.57 (–38.70, 9.57)	0.24	0.75	0.39
Inflammatory markers						
CRP (8 vs. 47)^1^	–.45 (–1.01, 0.10)	0.11	–0.42 (–0.76, 0.00)	<0.05	0.01	0.91
TNF-α (2 vs. 8)^1^	–0.11^3^ (–0.19, –0.03)	<0.05	–0.30 (–0.48, –0.12)	<0.001^2^	3.79	0.05
IL-6 (4 vs. 6)^1^	–0.04 (–0.12, 0.04)	0.34	–0.24 (–0.94, 0.47)	0.51	0.30	0.59
Body composition						
BW (29 vs. 106)^1^	–0.78 (–1.76, 0.20)	0.12	–1.94 (–2.43, –1.45)	<0.001^2^	4.35	<0.05
BMI (17 vs. 76)^1^	–0.48 (–0.80, –0.16)	<0.001^2^	–0.74 (–0.97, –0.51)	<0.001^2^	1.61	0.20
FM (10 vs. 56)^1^	–1.00 (–1.70, –0.29)	<0.05	–0.83 (–1.22, –0.44)	<0.001^2^	0.17	0.68
BFP (11 vs. 37)^1^	–0.90 (–1.48, –0.32)	<0.001^2^	–0.76 (–1.10, –0.39)	<0.001^2^	0.20	0.66
LM (6 vs. 25)^1^	–0.45 (–1.91, 1.01)	0.55	–0.29 (–0.57, –0.02)	<0.05	0.04	0.84
FFM (3 vs. 24)^1^	–1.12^3^ (–3.50, 1.26)	0.36	–0.42 (–0.58, –0.26)	<0.001^2^	0.33	0.56
WC (15 vs. 66)^1^	–2.20 (–3.39, –1.00)	<0.001	–1.80 (–2.52, –1.08)	<0.001^2^	0.32	0.57
HC (3 vs. 15)^1^	–.40^3^ (–1.83, 1.03)	0.58	–1.91 (–2.85, –0.97)	<0.001^2^	2.99	0.08
WHR (1 vs. 22)^1^	–0.01^3^ (–0.04, 0.02)	0.48	–0.01 (–0.02, 0.00)	<0.05	0.01	0.91
VAT (0 vs. 6)^1^	—		–0.28 (–0.50, –0.05)	<0.05		—

	Consultation		Food		Diff.	
	SMD (95% CI)	*P*	SMD (95% CI)	*P*	Qb(1)	*P*

Blood pressure						
SBP (68 vs. 20)^4^	–2.29 (–3.59, –0.98)	<0.001^2^	–1.30 (–3.11, 0.51)	0.16	0.67	0.41
DBP (73 vs. 17)^4^	–1.32 (–2.11, –0.55)	<0.001^2^	–0.98 (–2.44, 0.48)	0.19	0.16	0.69
Lipid profile						
TG (103 vs. 41)^4^	–13.69 (–17.97, –9.41)	<0.001^2^	–18.65 (–25.56, –11.74)	<0.001^2^	1.44	0.23
TC (93 vs. 36)^4^	6.52 (3.20, 9.99)	<0.001^2^	–1.19 (–4.45, 2.08)	0.48	10.42	<0.001^2^
LDL (102 vs. 40)^4^	7.06 (4.61, 9.70)	<0.001^2^	–1.07 (–5.05, 2.90)	0.60	11.46	<0.001^2^
HDL (104 vs. 39)^4^	3.38 (2.27, 4.41)	<0.001^2^	2.00 (0.81, 3.18)	<0.001^2^	2.89	0.09
Non-HDL (5 vs. 7)^4^	7.42 (–4.16, 18.99)	0.21	–1.91 (–9.20, 5.38)	0.61	1.78	0.18
LDL–HDL (3 vs. 5)^4^	–0.33^3^ (–0.47, –0.19)	<0.001	–0.09 (–0.22, 0.04)	0.19	5.99	<0.05
TC-HDL (22 vs. 11)^4^	–0.09 (–0.26, 0.11)	0.30	–0.18 (–0.27, –0.08)	<0.001^2^	0.71	0.40
TG-HDL (14 vs. 2)^4^	–0.33 (–0.45, –0.21)	<0.001	–0.25^3^ (–0.74, 0.24)	0.31	0.09	0.76
VLDL (5 vs. 5)^4^	–2.22 (–5.50, 1.06)	0.19	–5.30 (–10.17, –0.42)	<0.05	1.06	0.30
ApoA1 (6 vs. 8)^4^	0.06 (0.00, 0.13)	0.06	0.02 (–0.01, 0.06)	0.20	1.21	0.27
ApoB (11 vs. 9)^4^	0.06 (0.01, 0.12)	<0.05	–0.01 (–0.03, 0.01)	0.39	5.81	<0.05
ApoB-ApoA1 (1 vs. 5)^4^	–0.05^3^ (–0.05, –0.05)	<0.001	–0.02 (–0.03, –0.01)	<0.001^2^	25.47	<0.001
Endothelial functions						
FMD, % (6 vs. 1)^4^	0.15 (–0.39, 0.68)	0.59	–1.50^3^ (–4.92, 1.92)	0.39	0.87	0.35
E-selectin (6 vs. 2)^4^	–2.01 (–4.11, 0.09)	0.06	–2.49^3^ (–7.30, 2.31)	0.31	0.03	0.86
sICAM-1(7 vs. 0)^4^	–12.60 (–28.93, 3.72)	0.13	—		—	
sVCAM-1(5 vs. 1)^4^	–0.02 (–29.59, 29.56)	1.00	–0.30^3^ (–0.75, 0.15)	0.19	0.00	0.99
Inflammatory markers						
CRP (46 vs. 9)^4^	–0.33 (–0.57, –0.03)	<0.001^2^	–1.41 (–3.83, 1.01)	0.25	0.75	0.39
TNF-α (6 vs. 4)^4^	–0.40 (–0.60, –0.20)	<0.001^2^	–0.11 (–0.17, –0.04)	<0.001^2^	7.38	<0.05
IL-6 (5 vs. 5)^4^	–0.23 (–1.01, 0.55)	0.57	–0.07 (–0.24, 0.10)	0.42	0.15	0.70
CVD risk, % (4 vs. 0)^4^	–1.05 (–1.89, –0.21)	<0.05	—		—	
Body composition						
BW (99 vs. 36)^4^	–1.99 (–2.56, –1.42)	<0.001^2^	–1.14 (–1.73, –0.56)	<0.001^2^	4.10	<0.05
BMI (72 vs. 21)^4^	–0.82 (–1.08, –0.56)	<0.001^2^	–0.42 (–0.66, –0.18)	<0.001^2^	5.02	<0.05
FM (49 vs. 17)^4^	–0.78 (–1.19, –0.38)	<0.001^2^	–0.98 (–1.69, –0.26)	<0.05	0.22	0.64
BFP (34 vs. 14)^4^	–0.80 (–1.17, –0.43)	<0.001^2^	–0.73 (––1.30, –0.18)	<0.05	0.04	0.84
LM (23 vs. 8)^4^	–0.20 (–.54, 0.14)	0.25	–0.58 (–1.14, –0.01)	<0.05	1.27	0.26
FFM (20 vs. 7)^4^	–0.44 (–0.61, –0.27)	<0.001^2^	–0.26 (–0.76, 0.24)	0.31	0.44	0.51
WC (67 vs. 17)^4^	–1.93 (–2.65, –1.20)	<0.001^2^	–1.59 (–2.73, –0.45)	<0.05	0.23	0.63
HC (13 vs. 5)^4^	–1.97 (–2.83, –1.10)	<0.001^2^	–1.04 (–3.04, 0.96)	0.31	0.69	0.40
WHR (20 vs. 3)^4^	–0.01 (–0.02, 0.00)	0.09	–0.02^3^ (–0.04, 0.01)	0.15	0.72	0.40
VAT (5 vs. 1)^4^	–0.25 (–0.52, 0.01)	0.06	–0.40^3^ (–0.55, –0.25)	<0.001	0.87	0.35

	Nonisocaloric		Isocaloric		Diff.	
	SMD (95% CI)	*P*	SMD (95% CI)	*P*	Qb(1)	*P*

Blood pressure						
SBP (38 vs. 37)^5^	–1.66 (–3.51, 0.18)	0.08	–1.96 (–3.28, –0.65)	<0.001^2^	0.07	0.79
DBP (41 vs. 74)^5^	–1.20 (–1.97, –0.43)	<0.001^2^	–0.84 (–2.02, 0.34)	0.16	0.26	0.61
Lipid profile						
TG (60 vs. 60)^5^	–18.79 (–24.95, –12.64)	<0.001^2^	–12.60 (–17.45, –7.75)	<0.001^2^	2.40	0.12
TC (54 vs. 54)^5^	4.97 (0.57, 9.38)	<0.05	3.34 (–0.30, 6.97)	0.07	0.32	0.57
LDL (62 vs. 58)^5^	4.79 (0.99, 8.59)	<0.05	4.54 (1.52, 7.55)	<0.001^2^	0.01	0.92
HDL (63 vs. 60)^5^	2.97 (1.37, 4.57)	<0.001^2^	2.56 (1.70, 3.42)	<0.001^2^	0.20	0.66
Non-HDL (3 vs. 5)^5^	–0.58^3^ (–5.80, 4.63)	0.83	0.32 (–5.74, 6.39)	0.92	0.05	0.82
TC-HDL (13 vs. 13)^5^	–0.07 (–0.22, 0.08)	0.34	–0.17 (–0.45, 0.12)	0.25	0.35	0.56
TG-HDL (6 vs. 6)^5^	–0.28 (–0.45, –0.11)	<0.001^2^	–0.48 (–0.75, –0.20)	<0.001^2^	1.39	0.24
VLDL (4 vs. 5)^5^	–3.96 (–8.47, 0.55)	0.08	–0.50 (–1.21, 0.22)	0.17	2.21	0.14
ApoA1 (8 vs. 6)^5^	0.02 (0.00, 0.04)	0.11	0.06 (–0.01, 0.13)	0.09	1.19	0.28
ApoB (8 vs. 11)^5^	–0.00 (–0.05, 0.04)	0.83	0.05 (0.00, 0.11)	<0.05	2.87	0.09
Inflammatory markers						
CRP (26 vs. 23)^5^	–0.52 (–1.05, 0.00)	0.05	–0.14 (–0.50, 0.22)	0.44	1.38	0.24
TNF-α (3 vs. 4)^5^	–0.23^3^ (–0.45, 0.00)	0.05	–0.17 (–0.32, –0.02)	<0.05	0.16	0.69
Body composition						
BW (50 vs. 50)^5^	–1.86 (–2.72, –1.01)	<0.001^2^	–1.27 (–1.83, –0.70)	<0.001^2^	1.30	0.25
BMI (36 vs. 38)^5^	–.92 (–1.28, –0.55)	<0.001^2^	–0.55 (–0.84, –0.25)	<0.001^2^	2.31	0.13
FM (22 vs. 27)^5^	–1.00 (–1.60, –0.41)	<0.001^2^	–0.52 (–1.06, 0.05)	0.07	1.35	0.25
BFP (17 vs. 18)^5^	–0.62 (–1.13, –0.11)	<0.05	–0.83 (–1.37, –0.28)	<0.001^2^	0.30	0.58
LM (6 vs. 25)^5^	–0.71 (–1.17, –0.24)	<0.001^2^	–0.11 (–0.74, 0.52)	0.73	2.22	0.14
FFM (9 vs. 11)^5^	–0.21 (–0.44, 0.02)	0.07	–0.54 (–0.82, –0.26)	<0.001^2^	3.08	0.08
WC (30 vs. 66)^5^	–2.82 (–3.98, –1.67)	<0.001^2^	–0.90 (–1.67, –0.14)	<0.05	7.35	<0.05
HC (5 vs. 8)^5^	–1.59 (–2.14, –1.04)	<0.001^2^	–1.77 (–3.39, –0.14)	<0.05	0.04	0.84
WHR (8 vs. 10)^5^	–0.01 (–0.02, 0.00)	<0.05	0.00 (–0.02, 0.01)	0.52	0.83	0.36

Restricted maximum likelihood (REML) was used for conducting the analyses.

Abbreviations: 95% CI, 95% confidence interval; ApoA1, Apolipoprotein A-I; ApoB, Apolipoprotein B; BFP, body fat percentage; BW, body weight (kg); Consultation, studies with consultation-only interventions; Crossover, crossover designs; CRP, C-reactive protein; CVD risk%, cardiovascular disease risk (percentage); DBP, diastolic blood pressure; Diff., group difference; FFM, fat-free mass (kg); Food, interventions with food provided; FM, fat mass (kg); FMD%, flow-mediated dilation (percentage); HC, hip circumference (cm); LM, lean mass (kg); Q(b), Cochran’s Q-between statistics; Parallel, parallel designs; SBP, systolic blood pressure; sICAM-1, soluble intercellular adhesion molecule-1; SMD, standardized mean difference; sVCAM-1, soluble vascular cell adhesion molecule-1; TC, total cholesterol; TG, triglycerides; VAT, visceral adipose tissue; WC, waist circumference (cm); WHR, waist–hip ratio.

1 (*k* vs. *k*) stands for the number of observations included for each associated group: crossover trials vs. parallel trials.

2 Represents statistical significance after adjusted for multiple testing correction (*P* < 0.00125) among analyses included >4 studies (*k*≥4).

3 Represents the number analyzed by fewer than 4 observations for the particular group.

4 (*k* vs. *k*) stands for the number of observations included for each associated group: consultation-only interventions vs. food-provided intervention.

5 (*k* vs. *k*) stands for the number of observations included for each associated group: nonisocaloric diets vs. isocaloric diets.

## Discussion

This meta-analysis of 174 randomized trials (*n =* 11,481; 27 countries) examined the effects of CRDs on cardiovascular and anthropometric outcomes. Consistent with previous meta-analyses [13,14], CRDs were associated with reduced TG, blood pressure, and increased HDL, LDL, and TC. The lipid energy model (LEM) offers a potential mechanistic explanation for these changes. Among lean individuals, with depletion of glucose and glycogen stores during CRDs, the body shifts toward utilizing fat (lipid) metabolism, leading to VLDL conversion to LDL and enhanced ApoA1 production, which supports HDL formation [209].

Given these mixed lipid effects, a critical question is whether CRDs are ultimately beneficial for cardiovascular health. This meta-analysis further evaluated stronger predictors of cardiovascular disease risk—lipid ratios (LDL–HDL, TG-HDL, and ApoB-ApoA1) [[210], [211], [212]]. Our findings demonstrated favorable effects on all these lipid ratios. Additionally, CRDs reduced inflammation and endothelial dysfunction markers (CRP, IL-6, and E-selectin). Overall, like previous studies [[8], [9], [10], [11], [12]], CRDs were associated with a significant reduction in overall CVD risk.

Regarding body composition, consistent with previous meta-analyses, CRDs led to significant reductions in BW [[15], [16], [17], [18],^213^], BMI [15,16,18], BFP [15,16,18], FFM, WC, FM, and VAT [18]. A significant reduction was also found in HC. The carbohydrate-insulin model provides a framework for these results: high insulin levels from carbohydrate intake promote lipogenesis and inhibit fat oxidation, favoring storage. CRDs lower insulin, which facilitates fat oxidation and weight loss. The observed loss of FFM and LM may be explained by the gluconeogenic demand for glucose by certain tissues, leading to protein catabolism.

### A closer look at diets with various macronutrient proportions

The effects varied by carbohydrate restriction level. Blood pressure improved with LCD and MCD but not KD. Increases in LDL and TC were more pronounced with greater carbohydrate restriction (i.e., KD), likely due to higher dietary fat intake, consistent with the LEM [209]. However, the improvement in the TG:HDL ratio suggests this rise may not be atherogenic. Due to limited observations (*k* < 4), valid significant effects were minimal, indicating a need for research.

Certain anthropometric improvements followed a dose–response pattern, with greater weight and BMI loss seen in stricter diets. However, these also resulted in greater losses of LM and FFM. This trade-off suggests that MCDs may offer a more balanced option. KDs and LCDs may be preferred for aggressive weight loss, provided LM and lipids are monitored.

As for macronutrient replacement strategies, previous meta-analyses have reported that higher protein intake [9,214] and higher fat intake [9] are associated with reduced CVD risk. This study further indicates that replacing carbohydrates with fat improved the lipid profile (increased HDL, reduced TG). Substitution with protein lowered TC while preserving HDL. Most notably, a combination replacement (using both fat and protein) yielded the most comprehensive cardiometabolic benefits, including reductions in blood pressure, inflammation, and endothelial dysfunction. For body composition, fat replacement and combination replacement showed broad benefits across multiple anthropometric markers. The narrower benefits from protein replacement may be due to a smaller number of available studies. Overall, combination replacement appears optimal for improving both cardiovascular and anthropometric outcomes.

### Differences in participants’ characteristics

CRDs exert sex-specific effects, with females exhibiting stronger cardiovascular improvements than males. Among participants with T2DM, CRDs led to greater enhancements in TG and HDL, without the elevations in LDL or TC. CRDs also conferred more pronounced anthropometric benefits (e.g., weight loss, WC reduction) in the T2DM subgroup.

For weight differences, a prior meta-analysis reports an inverse association between baseline BMI and LDL changes under LCDs: individuals with a healthy BMI (<25 kg/m^2^) experienced LDL increases, whereas those with overweight (BMI: 25–35 kg/m^2^) or obesity (BMI >35 kg/m^2^) showed neutral or reduced levels [215]. Contrary to the previous report, LDL increased even among individuals with overweight/obesity in this study. The "lipid overflow hypothesis" may explain: in obesity, saturated fat from a high-fat CRD could exacerbate the dysregulation of hepatic lipoprotein metabolism in individuals with dysfunctional adipose tissue [216]. Under CRDs, increased dietary fat intake may exacerbate this pathway, driving LDL rises. Further research is needed to clarify the interplay between macronutrient composition, adipose dysfunction, and lipid dynamics across various populations.

### Differences in study designs

Sensitivity analyses found broadly consistent results across study designs. Parallel trials yielded more significant results than crossover designs. Consultation-only interventions produced stronger effects than those providing food, potentially because higher attrition in consultation studies left a more adherent and motivated completer population. Similar results between isocaloric and nonisocaloric trials might suggest that the macronutrient composition independently influences outcomes beyond caloric intake.

### Potential negative effects of CRDs

Potential drawbacks include the observed reductions in LM and increases in LDL and TC. Other reported concerns include the risk of osteoporosis [217], nutritional deficiencies and gut microbiota dysbiosis [218], hair loss, muscle cramps [213], gastrointestinal complications [218], and hyperuricemia [219]. The evidence for renal impairment remains inclusive. Despite reported negative effects [220], research also suggested that nutritional ketosis, a metabolic state induced by KD, may confer renal benefits among people with T2DM [221]. Additionally, the utility of CRDs may be limited for high-intensity anaerobic athletes due to the reliance on glucose. Clinical supervision is advised for implementing very low-carbohydrate diets.

### Limitations

Substantial heterogeneity was present due to diverse populations and interventions. Significant funnel plot asymmetry for outcomes including SBP, blood lipids (non-HDL, LDL:HDL, ApoB:ApoA1), CVD (%), sICAM-1, WC, HC, WHR, and VAT indicates potential publication bias, which may inflate effect estimates and necessitates cautious interpretation of these results. The conservative adjusted alpha for multiple testing correction might increase type II error risk, potentially leading to the false dismissal of true effects for certain outcomes. Use of SMDs may have inflated variance [222]. High attrition in some trials may also have introduced bias. To mitigate these issues, sensitivity analyses were conducted and confirmed the robustness of the findings. Another important limitation of this meta-analysis is the inadvertent omission of at least one eligible study [223], due to the search strategy (e.g., “RCT” not fully captured by our predefined search terms). While our results remain consistent with the findings of the omitted trial, we acknowledge that incomplete study inclusion has the potential to introduce bias. Future reviews should consider broader search strategies to minimize this risk. Other limitations include insufficient studies (*k* < 4) for certain subgroup analyses, an inability to assess food quality, and a geographic skew toward North America and Europe, with underrepresentation from Asia, Africa, and South America.

In conclusion, this meta-analysis supports the cardiovascular (including blood pressure, lipid profile, and inflammation markers) and anthropometric benefits of CRDs. MCDs provided a balanced benefit–risk profile, whereas stricter diets (LCD, KD) were more effective for weight loss. Replacing carbohydrates with a combination of fat and protein yielded the most comprehensive benefits. Benefits were most pronounced among females and individuals with overweight/obesity. Despite potential drawbacks, CRDs represent a viable dietary strategy for cardiovascular health when implemented with appropriate professional guidance, particularly for more restrictive regimens.

## Author contributions

The authors’ responsibilities were as follows – SF, BC, AB: designed research; SF, RL, SC, HW: conducted research; SF, CT: analyzed data; RL: conducted visualization; SF, CT, BC: wrote the paper; SF, RL, CT, BC, AB: reviewed and revised the paper; SF: has primary responsibility for the final content; and all authors: read and approved the final manuscript.

## Data availability

Data described in the manuscript, code book, and analytic code will be made publicly and freely available without restriction at https://figshare.com/s/7dd68520ec63bf964d77

## Funding

The authors reported no funding received for this study.

## Conflict of interest

The authors report no conflicts of interest.
