# Supplementary material for: Effects of carbohydrate-restricted diets and macronutrient replacements on cardiovascular health and body composition in adults: a meta-analysis of randomized trials
Source: Am J Clin Nutr. 2025 Sep 8;122(5):1461–78. doi: 10.1016/j.ajcnut.2025.09.012 (PMC12799388; doi:10.1016/j.ajcnut.2025.09.012)
Supplement: Multimedia component 2 [file mmc2.docx]

Supplementary Materials

Effects of carbohydrate-restricted diets and macronutrient replacements on cardiovascular health and body composition in adults: A meta-analysis of randomized trials; Feng, Shuo

Search Term----------------------------------------------------------------------------------------------------2

Calculation Formulas-----------------------------------------------------------------------------------------3

Units Conversion----------------------------------------------------------------------------------------------5

Macronutrient Percentage Calculation---------------------------------------------------------------------6

Supplementary Tables (Sensitivity analysis)--------------------------------------------------------------7

Search Terms

“Carbohydrate Diet’ OR ‘Low Carbohydrate Diet’ OR ‘Carbohydrate-Restricted Diet’ OR ‘Carbohydrate Restricted Diet’ OR ‘Ketogenic Diet’ OR keto OR ‘Paleolithic Diet’ OR ‘Paleo Diet’ OR ‘Caveman Diet’ OR ‘Hunter-Gatherer Diet’ OR ‘atkins diet’ OR ‘modified atkins diet’ OR ‘high-protein low-carbohydrate diet’ OR ‘very low carbohydrate diet’ OR VLC OR VLCK

AND

"coronary heart disease" OR "ischemic heart disease" OR "myocardial ischemia" OR "myocardial infarction" OR "cardiovascular disease" OR "cardiovascular events" OR "cardiovascular mortality" OR "angina" OR "CVD" OR coronary heart disease "CHD" OR "Stroke" OR "cardiovascular health" OR "cardiovascular function" OR cardiovascular risk factors OR blood sugar OR insulin resistance OR hyperlipidemia OR lipid metabolism OR hyperuricemia OR metabolic syndrome OR metabolism OR metabolic health OR glucose OR type 2 diabetes OR diabetes mellitus OR T2DM OR non-insulin dependent diabetes mellitus or NIDDM OR late onset diabetes OR adult onset diabetes OR sugar diabetes OR pre-diabetes OR impaired fasting glucose OR IFG or impaired glucose tolerance OR IGT OR impaired fasting glycaemia OR HOMA-IR OR HDL OR triglycerides OR obesity OR overweight OR abdominal obesity OR "body composition" OR "fat mass" OR "fat percentage" OR "body fat" OR "lean mass" OR "body lean" OR "body mass" OR weight OR "body mass index" OR BMI OR "Visceral adipose tissue" OR "adipose tissue" OR "Perinephric fat" OR "muscle mass"

AND

Adaptive Clinical Trial or Clinical Trial or Clinical Trial or Clinical Trial Protocol or Randomized Controlled Trial OR parallel trial OR crossover trial OR controlled trial

Calculation formulas

1. For studies reporting changes in means and their associated SDs between pre- and post-intervention, the change in mean and its associated SD for the control group (groups with higher intake of carbohydrate) were treated as the mean and SD for the control group in the meta-analyses. The change in mean and its associated SD for the treatment group (groups with lower intake of carbohydrate) were treated as the mean and SD for the treatment group in the meta-analyses.
2. For studies reporting changes in means and their associated SEs between pre- and post-intervention, we first calculated the associated SDs by the formula:
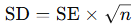


Then, the calculated SDs were used for the meta-analysis as shown in the first scenario.

1. For studies reporting changes in means and their associated 95% CI, we calculated SDs by using the formula:


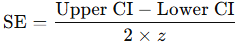
Z=1.96

Then, the calculated SDs were used for the meta-analysis as shown in the first scenario.

1. For studies reporting pre- and post-means and their associated SDs for each group in the study. Following the instructions in the Cochrane Handbook for Systematic Reviews of Interventions (Higgins et al., 2019), the change in mean is calculated by


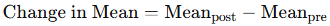


The change in SD is calculated by


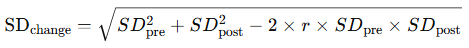


The correlation r is calculated by the average of correlation r calculated from studies reporting data of both SD_change_ and SD_pre_ and SD_post_ by using the formula:


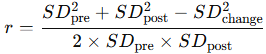


The average correlation r was used in studies to calculate the SD_change_. Then the calculated change in mean and SD_change_ were used in the meta-analysis.

1. For studies reporting pre- and post-means and their associated SEs for each group in the study, the change in mean is calculated by


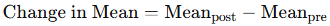


The SDs were calculated by:
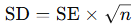
. Then, the calculated pre and post SDs were used to calculate SD_change_, following the steps in scenario 4.

1. For studies reporting pre- and post-means and their associated 95% CI for each group in the study, the change in mean is calculated by


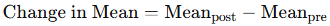


The SDs were calculated by:
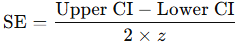
Z=1.96

. Then, the calculated pre and post SDs were used to calculate SD_change_, following the steps in scenario 4.

1. For studies reporting pre and post-medians and their associated interquartile ranges for each group, following the suggested formulas in Wan et al. (2014) and Luo et al. (2018), medians can be treated as means, and SDs were calculated by
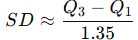


Following the steps in scenario 4, changes in means and SDs for both treatment groups and control groups were used for analysis.

References

Wan, X., Wang, W., Liu, J., & Tong, T. (2014). *Estimating the sample mean and standard deviation from the sample size, median, range and/or interquartile range*. BMC Medical Research Methodology, 14, 135. <https://doi.org/10.1186/1471-2288-14-135>

Luo, D., Wan, X., Liu, J., & Tong, T. (2018). *Optimally estimating the sample mean from the sample size, median, mid-range, and/or mid-quartile range*. Statistical Methods in Medical Research, 27(6), 1785–1805. <https://doi.org/10.1177/0962280216669183>

Higgins JP, Li T, Deeks JJ. 2019. Choosing effect measures and computing estimates of effect. In: Higgins JPT, T homas J, Chandler J, Cumpston M, Li T, Page MJ, Welch VA, editors. Cochrane handbook for systematic reviews of interventions. Wiley; p. 143–176.

Units Conversion

For cardiovascular health markers

Blood pressure: SBP and DBP: mmHg

Lipid profile:

Triglyceride: mg/dl; conversion formula: 1mg/dL=mmol/L x 88.57

Total cholesterol, LDL, HDL, VLDL: mg/dL; conversion formula: 1mg/dL=mmol/L x 38.67

ApoA-1 and ApoB: g/L. conversion formula: 1 g/L=100mg/dL=0.0357 x mmol/L

Endothelial function markers

e-selectin: ng/mL

s-ICAM-1: ng/mL

s-VCAM-1: ng/mL

Inflammatory marker

CRP: mg/L; conversion:1mg/L=1ug/ml=9.5239nmol/L=0.1mg/dL

TNF-a

IL-6: pg/mL; conversion: 1pg/ml=0.001ng/mL

Body composition

Body weight: kg

Fat mass: kg

Fat-free mass: kg

Lean mass: kg

Waist circumference: cm

Hip circumference: cm

Visceral adipose tissue: kg

Macronutrient percentage calculation

For studies that reported macronutrient percentage of total calorie intake, we used the original report.

For studies reported macronutrient in grams and total calories, we used protein(g)*4=total calories from protein. Carbohydrate(g)*4=total calories from carbohydrate. Fat(g)*9=total calories from fat. And the percentage was calculated by dividing the calories from each macronutrient by the total calorie intake.

Supplementary Tables 2-6 for Sensitivity Analysis

Effects of carbohydrate-restricted diets and macronutrient replacements on cardiovascular health and body composition in adults: A meta-analysis of randomized trials; Feng, Shuo

Supplementary Table 2. The pooled effect sizes (SMD) of outcomes

|  | N of Obs. | SMD (95% CI) | p | I^2^ |
| --- | --- | --- | --- | --- |
| Cardiovascular health |  |  |  |  |
| Blood pressure |  |  |  |  |
| SBP, mmHg | 67 | -1.86 (-2.61, -1.11) | <.001 | 99.65% |
| DBP, mmHg | 70 | -1.06 (-1.53, -.59) | <.001 | 99.03% |
| Lipid profile |  |  |  |  |
| TG, mg/dL | 91 | -12.46 (-14.80, -10.11) | <.001 | 97.08% |
| TC, mg/dL | 92 | 2.26 (.62, 3.90) | <.05 | 99.03% |
| LDL, mg/dL | 110 | 3.47 (2.01, 4.93) | <.001 | 99.03% |
| HDL, mg/dL | 115 | 2.30 (1.81, 2.79) | <.001 | 99.33% |
| Non-HDL, mg/dL | 5 | -1.40 (-3.83, 1.03) | .26 | 79.92% |
| LDL-HDL ratio | 6 | -.14 (-.26, -.03) | <.05 | 79.00% |
| TC-HDL ratio | 25 | -.11 (-.20, -.03) | <.05 | 98.43% |
| VLDL, mg/dL | 6 | -1.48^1^ (-3.99, 1.02) | .25 | 96.40% |
| ApoA1, g/L | 12 | .03 (.01, .05) | <.05 | 78.38% |
| Endothelial functions |  |  |  |  |
| sICAM-1, ng/mL | 4 | -19.35 (-41.56, 2.87) | .09 | 91.66% |
| Inflammatory markers |  |  |  |  |
| CRP, mg/L | 40 | -.27 (-.53, -.02) | <.05 | 98.49% |
| IL-6, pg/mL | 9 | -.26 (-.72, .19) | .25 | 92.11% |
| Body composition |  |  |  |  |
| BW, kg | 111 | -1.49 (-1.82, -1.16) | <.001 | 99.09% |
| BMI | 71 | -.52 (-.65, -.39) | <.001 | 96.45% |
| FM, kg | 48 | -.68 (-.94, -.43) | <.001 | 86.56% |
| BFP | 35 | -.67 (-.92, -.43) | <.001 | 97.11% |
| LM, kg | 26 | -.08^1^ (-.27, .12) | .43 | 96.05% |
| FFM, kg | 19 | -.44 (-.63, -.26) | <.001 | 68.56% |
| WC, cm | 65 | -1.77 (-2.21, -1.33) | <.001 | 99.21% |
| HC, cm | 11 | -.98 (-1.63, -.32) | <.001 | 81.72% |
| WHR | 19 | -.01 (-.02, -.01) | <.001 | 84.22% |
| VAT, kg | 4 | -.19 (-.32, -.06) | <.001 | 91.00% |

Note. ^1^.refers to deviations in the statistical significance from the original analyses

Abbreviations: N of Obs. refers to the number of observations included for the associated outcomes. SMD (95% CI) stands for standardized mean difference and the corresponding 95% confidence interval. SBP: Systolic Blood Pressure; DBP: Diastolic Blood Pressure; TG: Triglycerides; TC: Total Cholesterol; LDL: Low-Density Lipoprotein; HDL: High-Density Lipoprotein; non-HDL: Non–High-Density Lipoprotein Cholesterol; ApoA1: Apolipoprotein A-I; sICAM-1: Soluble Intercellular Adhesion Molecule-1; CRP: C-Reactive Protein; IL-6: Interleukin-6; BW stands for body weight (kg); BMI stands for body weight index; FM stands for fat mass (kg); BFP stands for body fat percentage; LM stands for lean mass (kg); FFM stands for fat-free mass (kg); WC stands for waist circumference (cm); HC stands for hip circumference (cm); WHR stands for waist-hip ratio; VAT stands for visceral adipose tissue.

Supplementary Table 3. Subgroup analysis of diet characteristics (types and replacements)

|  | KD | | LCD | | MCD | |
| --- | --- | --- | --- | --- | --- | --- |
|  | SMD | p | SMD | p | SMD | p |
| Blood pressure |  |  |  |  |  |  |
| SBP (13 vs.17 vs.26) | -1.77 | .10 | -2.04^4^ | <.05 | -1.88^4^ | <.001 |
| DBP (10 vs.17 vs.31) | -.79 | .17 | -.90 | .08 | -1.40 | <.001 |
| Lipid profile |  |  |  |  |  |  |
| TG (19 vs.20 vs.38) | -13.81 | <.001 | -14.44 | <.001 | -10.80 | <.001 |
| TC (18 vs.16 vs.43) | 5.03 | .06 | 4.05 | .05 | .22 | .80 |
| LDL (19 vs.23 vs.50) | 5.92^4^ | <.05 | 5.57^4^ | <.001 | 1.69 | .08 |
| HDL (20 vs.24 vs.53) | 4.52 | <.001 | 2.10 | <.001 | 1.84 | <.001 |
| LDL-HDL (0 vs.1 vs.5) | n/a |  | -.23 | .32 | -.14 | <.05 |
| TC-HDL (6 vs.5 vs.9) | -.05 | .69 | -.06 | .31 | -.19 | <.05 |
| Inflammatory markers |  |  |  |  |  |  |
| CRP (9 vs.8 vs.17) | -.84 | <.05 | -.15 | .25 | -.06^4^ | .80 |
| Body composition |  |  |  |  |  |  |
| BW (21 vs.26 vs.48) | -2.03 | <.001 | -1.72 | <.001 | -.94 | p<.001 |
| BMI (15 vs.15 vs.32) | -.57 | <.001 | -.57 | <.001 | -.42 | p<.001 |
| FM (11 vs.8 vs.23) | -.27^4^ | .31 | -.84^4^ | <.001 | -.79^4^ | p<.001 |
| BFP (5 vs.8 vs.20) | -.39 | .51 | -.54 | <.05 | -.77 | p<.001 |
| LM (5 vs.5 vs.14) | -.26 | <.001 | -.15 | .69 | .01 | p=.95 |
| FFM (3 vs.6 vs.7) | -.55^4^ | .07 | -.35^4^ | <.05 | -.44^4^ | P<.05 |
| WC (8 vs.18 vs.33) | -2.74 | <.001 | -2.29 | <.001 | -1.24^4^ | p<.001 |
| HC (3 vs.2 vs.5) | -.91^4^ | .22 | -1.06^4^ | .36 | -1.00 | <.05 |
| WHR (4 vs.5 vs.9) | -.02 | <.001 | -.00 | .61 | -.01^4^ | P<.001 |
|  | Fat | | Protein | | Combination | |
| Blood pressure |  |  |  |  |  |  |
| SBP (16 vs.8 vs.43) | -.78 | .23 | -.31 | .63 | -2.53 | <.001 |
| DBP (14 vs.11 vs.45) | -.62 | .10 | -.49 | .28 | -1.37 | <.001 |
| Lipid profile |  |  |  |  |  |  |
| TG (29 vs.14 vs.48) | -12.07 | <.001 | -12.31^4^ | <.001 | -12.87 | <.001 |
| TC (32 vs.14 vs.46) | 2.85^4^ | .06 | -2.71^4^ | <.05 | 3.49 | <.001 |
| LDL (36 vs.14 vs.60) | 5.74 | <.001 | -1.43 | <.05 | 3.28 | <.001 |
| HDL (39 vs.16 vs.60) | 2.80 | <.001 | 1.08 | .16 | 2.28 | <.001 |
| Non-HDL (2 vs.0 vs.3) | -1.95 | .36 | n/a |  | .04 | .77 |
| TC-HDL (11 vs.1 vs.13) | -.09 | .05 | .10 | .05 | -.15 | <.05 |
| Inflammatory markers |  |  |  |  |  |  |
| CRP (11 vs.5 vs.24) | -.02 | .92 | -.25 | .39 | -.43 | <.05 |
| IL-6 (4 vs.1 vs.4) | -.10 | .41 | -.990 | .51 | -.21 | .59 |
| Body composition |  |  |  |  |  |  |
| BW (38 vs.11 vs.62) | -.95^4^ | <.001 | -.82 | .06 | -1.90 | <.001 |
| BMI (24 vs.6 vs.41) | -.28 | <.001 | -.72^4^ | <.001 | -.62 | <.001 |
| FM (22 vs.9 vs.17) | -.39^4^ | <.05 | -.91 | <.05 | -.84 | <.001 |
| BFP (16 vs.2 vs.17) | -.40 | <.05 | -.72^4^ | <.001 | -.85 | <.001 |
| LM (11 vs.6 vs.9) | -.06 | .70 | -.30 | .10 | .01 | .95 |
| FFM (10 vs.0 vs 9) | -.37 | <.001 |  |  | -.53 | <.001 |
| WC (20 vs.5 vs.40) | -.93 | <.05 | -1.29 | .22 | -2.23 | <.001 |
| HC (4 vs.0 vs.7) | -.83 | .13 | n/a |  | -1.07^4^ | <.05 |
| WHR (7 vs.0 vs.12) | -.01 | <.001 | n/a |  | -.01^4^ | <.001 |

Note.

^1^(k vs. k vs. k) stands for the number of observations included for each associated group: KD vs. LCD vs. MCD;

^2^(k vs. k vs. k) stands for the number of observations included for each associated group: Fat vs. Protein vs. Combination.

^3^represents the number analyzed by fewer than 4 observations for the particular group.

^4^.refers to deviations in the statistical significance from the original analyses

Abbreviations:

KD stands for ketogenic diet: ≤10% of total calories or 20-50 g/day; LCD stands for low-carb diet: 10-26% of total calories or 50–130 g/day; MCD stands for moderate-carb diet: 26-45% of total calories or 130-230 g/day.

SMD for standardized mean difference.

SBP: Systolic Blood Pressure; DBP: Diastolic Blood Pressure; TG: Triglycerides; TC: Total Cholesterol; LDL: Low-Density Lipoprotein; HDL: High-Density Lipoprotein; non-HDL: Non–High-Density Lipoprotein Cholesterol; CRP: C-Reactive Protein; IL-6: Interleukin-6; BW stands for body weight (kg); BMI stands for body weight index; FM stands for fat mass (kg); BFP stands for body fat percentage; LM stands for lean mass (kg); FFM stands for fat-free mass (kg); WC stands for waist circumference (cm); HC stands for hip circumference (cm); WHR stands for waist-hip ratio.

Supplementary Table 4. Meta-regression of intervention length (in weeks)

|  | b-coefficient | Std.err | p |
| --- | --- | --- | --- |
| Blood pressure |  |  |  |
| SBP | .02 | .02 | .19 |
| DBP | .02 | .01 | .10 |
| Lipid profile |  |  |  |
| TG | -.03 | .06 | .65 |
| TC | -.001 | .04 | .97 |
| LDL | -.04 | .03 | .22 |
| HDL | .01 | .01 | .57 |
| Non-HDL | .06 | .11 | .56 |
| LDL-HDL ratio | -.03 | .01 | <.001 |
| TC-HDL ratio | -.004 | .002 | .07 |
| Inflammatory markers |  |  |  |
| C-reactive protein (CRP) | .01 | .01 | .33 |
| Interleukin-6 (IL-6) | -.03 | .004 | <.001 |
| Body composition |  |  |  |
| BW | -.005 | .01 | .56 |
| BMI | -.001 | .003 | .84 |
| FM | -.004 | .01 | .74 |
| BFP | -.01^1^ | .005 | <.01 |
| LM | -.02^1^ | .01 | <.05 |
| FFM | .00 | .01 | .99 |
| WC | .01 | .01 | .37 |
| HC | -.02 | .01 | .13 |
| WHR | .0001 | .0002 | .86 |

Note. ^1^.refers to deviations in the statistical significance from the original analyses

Abbreviations:

Std. err. stands for standard error.

SBP: Systolic Blood Pressure; DBP: Diastolic Blood Pressure; TG: Triglycerides; TC: Total Cholesterol; LDL: Low-Density Lipoprotein; HDL: High-Density Lipoprotein; non-HDL: Non–High-Density Lipoprotein Cholesterol; CRP: C-Reactive Protein; TNF-α: Tumor Necrosis Factor-alpha; IL-6: Interleukin-6; BW stands for body weight (kg); BMI stands for body weight index; FM stands for fat mass (kg); BFP stands for body fat percentage; LM stands for lean mass (kg); FFM stands for fat-free mass (kg); WC stands for waist circumference (cm); HC stands for hip circumference (cm); WHR stands for waist-hip ratio.

Supplementary Table 5. Subgroup analysis of population characteristics (sex, weight, and diabetic status)

|  | Male | | Female | | Diff. | |
| --- | --- | --- | --- | --- | --- | --- |
|  | SMD | p | SMD | p | Qb(1) | p |
| Blood pressure |  |  |  |  |  |  |
| SBP (2 vs.12) | -3.09 | .48 | -3.40^5^ | <.001 | .00 | .95 |
| DBP (3 vs.15) | -2.23 | .22 | -1.29 | <05 | .24 | .62 |
| Lipid profile |  |  |  |  |  |  |
| TG (6 vs.18) | -6.40^5^ | .26 | -13.99 | <.001 | 1.48 | .22 |
| TC (7 vs.17) | 3.28 | .26 | .30 | .85 | .82 | .36 |
| LDL (8 vs.22) | 8.59^5^ | <.05 | 2.79 | .11 | 2.29 | .13 |
| HDL (10 vs.23) | 2.76^5^ | <.05 | 2.44 | <.001 | .07 | .79 |
| TC-HDL (6 vs.8) | .02 | .82 | -.17 | <.05 | 2.73 | .10 |
| Inflammatory markers |  |  |  |  |  |  |
| CRP (1 vs.8) | -.76 | .06 | -.50^5^ | <.001 | .39 | .53 |
| Body composition |  |  |  |  |  |  |
| BW (5 vs. 19) | -1.46 | <.05 | -1.63^5^ | <.001 | .06 | .80 |
| BMI (8 vs. 17) | -.48 | <.05 | -.56 | <.001 | .09 | .76 |
| FM (5 vs. 10) | -.74 | .20 | -.80^5^ | <.05 | .01 | .92 |
| BFP (4 vs. 6) | -.24 | .56 | -.73^5^ | .06 | .72 | .40 |
| LM (3 vs. 10) | .11^5^ | .62 | -.07 | .64 | .45 | .50 |
| FFM (3 vs. 5) | -.44 | .22 | -.59 | <.001 | .16 | .69 |
| WC (3 vs. 12) | -2.63 | .11 | -1.81 | <.001 | .23 | .63 |
| WHR (0 vs. 10) | n/a |  | -.01 | <.001 | n/a |  |
|  | Non-overweight | | Overweight/Obese | | Diff. | |
| Blood pressure |  |  |  |  |  |  |
| SBP (2 vs.51) | -2.86 | .36 | -1.84 | <.001 | .11 | .74 |
| DBP (1 vs.53) | .20^5^ | <.001 | -.94 | <.001 | 18.10 | <.001 |
| Lipid profile |  |  |  |  |  |  |
| TG (3 vs.71) | -3.55 | .11 | -12.50 | <.001 | 11.65 | <.001 |
| TC (4 vs.67) | 2.42 | .49 | 2.63 | <.05 | .00 | .95 |
| LDL (4 vs.83) | 3.18 | .24 | 3.75 | <.001 | .04 | .84 |
| HDL (4 vs.89) | 1.90 | <.05 | 2.26 | <.001 | .14 | .71 |
| TC-HDL (1 vs.17) | -.05 | <.001 | -.09 | .10 | .80 | .44 |
| Inflammatory markers |  |  |  |  |  |  |
| CRP (1 vs.33) | -.80 | <.05 | -.26^1^ | .08 | 2.74 | .10 |
| Body composition |  |  |  |  |  |  |
| BW (3 vs. 83) | .19 | .56 | -1.62 | <.001 | 24.03 | <.001 |
| BMI (2 vs. 54) | .12 | .23 | -.55 | <.001 | 29.68 | <.001 |
| FM (2 vs. 39) | .19 | .64 | -.66 | <.001 | 4.01 | .05 |
| BFP (1 vs. 28) | -.30 | .25 | -.64 | <.001 | 1.30 | .25 |
| LM (0 vs. 20) | n/a |  | -.07 | .54 | n/a |  |
| FFM (1 vs. 17) | 0^5^ | 1.00 | -.45 | <.001 | 1.72 | .19 |
| WC (2 vs. 51) | -.77^5^ | .63 | -1.92 | <.001 | .51 | .48 |
| HC (0 vs. 8) | n/a |  | -.69 | <.05 | n/a |  |
| WHR (0 vs. 17) | n/a |  | -.01 | <.001 | n/a |  |
|  | Non-diabetic | | T2DM | | Diff. | |
| Blood pressure |  |  |  |  |  |  |
| SBP (31 vs.9) | -1.33 | <.05 | -1.30 | .10 | .00 | .97 |
| DBP (35 vs.13) | -.76^5^ | <.001 | -.57 | .32 | .10 | .76 |
| Lipid profile |  |  |  |  |  |  |
| TG (45 vs.19) | -11.40 | <.001 | -15.10 | <.001 | 2.12 | .15 |
| TC (45 vs.24) | 2.48 | .05 | 1.13 | .51 | .41 | .52 |
| LDL (55 vs.26) | 3.86 | <.001 | 1.93 | .20 | 1.07 | .30 |
| HDL (56 vs.27) | 2.59 | <.001 | 2.33 | <.001 | .20 | .66 |
| TC-HDL (14 vs.3) | -.14 | <.05 | -.14 | .19 | .00 | .97 |
| Inflammatory markers |  |  |  |  |  |  |
| CRP (25 vs.6) | -.56 | <.001 | .12 | .75 | 2.78^5^ | .10 |
| Body composition |  |  |  |  |  |  |
| BW (52 vs. 28) | -1.51 | <.001 | -1.26 | <.001 | .32 | .57 |
| BMI (31 vs. 19) | -.54 | <.001 | -.59 | <.001 | .07 | .79 |
| FM (26 vs. 6) | -.46 | <.05 | -.98^5^ | <.001 | 2.68 | .10 |
| BFP (19 vs. 7) | -.61 | <.001 | -.98^5^ | <.001 | 2.38 | .12 |
| LM (17 vs. 1) | -.12 | .37 | -.05 | .85 | .06 | .81 |
| FFM (9 vs. 2) | -.50 | <.001 | -.30 | .25 | 7.39 | <.05 |
| WC (32 vs. 17) | -2.07 | <.001 | -1.45 | <.001 | 1.30 | .25 |
| HC (7 vs. 2) | -.63^5^ | .08 | -2.39 | <.001 | 7.81 | <.05 |
| WHR (15 vs. 1) | -.01^5^ | <.05 | -.01^5^ | .48 | .07 | .80 |

Note. ^1^(k vs. k) stands for the number of observations included for each associated group: Male vs. Female

^2^(k vs. k) stands for the number of observations included for each associated group: Non-overweight vs. Overweight/obesity

^3^(k vs. k) stands for the number of observations included for each associated group: Non-diabetic vs. T2D

^4^represents the number analyzed by fewer than 4 observations for the particular group.

^5^.refers to deviations in the statistical significance from the original analyses

Abbreviations:

Diff. stands for group difference.

n/a refers to not applicable due to insufficient data for analysis.

SMD for standardized mean difference.

T2D stands for type-2 diabetes

SBP: Systolic Blood Pressure; DBP: Diastolic Blood Pressure; TG: Triglycerides; TC: Total Cholesterol; LDL: Low-Density Lipoprotein; HDL: High-Density Lipoprotein; non-HDL: Non–High-Density Lipoprotein Cholesterol; CRP: C-Reactive Protein; BW stands for body weight (kg); BMI stands for body weight index; FM stands for fat mass (kg); BFP stands for body fat percentage; LM stands for lean mass (kg); FFM stands for fat-free mass (kg); WC stands for waist circumference (cm); HC stands for hip circumference (cm); WHR stands for waist-hip ratio; VAT stands for visceral adipose tissue.

Supplementary Table 6. Subgroup analysis of study characteristics

|  | Crossover | | Parallel | | Diff. | |
| --- | --- | --- | --- | --- | --- | --- |
|  | SMD | p | SMD | p | Qb(1) | p |
| Blood pressure |  |  |  |  |  |  |
| SBP (15 vs.52)^1^ | -.64 | .38 | -1.86 | <.001 | 3.05 | .08 |
| DBP (15 vs.55)^1^ | -1.26^5^ | <.001 | -.99 | <.001 | .27 | .60 |
| Lipid profile |  |  |  |  |  |  |
| TG (21 vs.70)^1^ | -12.80 | <.001 | -12.35 | <.001 | .02 | .88 |
| TC (27 vs.65)^1^ | 2.16 | .17 | 2.27^5^ | <.05 | .00 | .95 |
| LDL (33 vs.77)^1^ | 2.73 | .05 | 3.68 | <.001 | .32 | .57 |
| HDL (29 vs.86)^1^ | 2.08^5^ | <.001 | 2.35 | <.001 | .21 | .65 |
| Non-HDL (1 vs.4)^1^ | 1.70^4^ | .62 | -1.76 | .20 | .86 | .26 |
| TC-HDL (13 vs.12)^1^ | -.13 | .06 | -.10 | .06 | .12 | .73 |
| Inflammatory markers |  |  |  |  |  |  |
| CRP (7 vs.33)^1^ | -.49 | .08 | -.23 | .11 | .67 | .41 |
| IL-6 (4 vs.5)^1^ | -.04 | .34 | -.36 | .31 | .81 | .37 |
| Body composition |  |  |  |  |  |  |
| BW (25 vs. 86)^1^ | -1.08^5^ | <.001 | -1.58 | <.001 | 1.48 | .22 |
| BMI (15 vs. 56)^1^ | -.43 | <.001 | -.54 | <.001 | .40 | .52 |
| FM (9 vs. 39)^1^ | -.99 | <.05 | -.61 | <.001 | .91 | .34 |
| BFP (8 vs. 27)^1^ | -.75 | <.05 | -.66 | <.001 | .08 | .77 |
| LM (4 vs. 22)^1^ | .38 | .05 | -.14 | .19 | 5.46 | <.05 |
| FFM (1 vs. 18)^1^ | .00 | 1.00 | -.47 | <.001 | 1.89 | .17 |
| WC (12 vs. 50)^1^ | -1.85 | <.001 | -1.81 | <.001 | .00 | .94 |
| HC (2 vs. 9)^1^ | -.73^4^ | .35 | -1.06 | <.05 | .14 | .71 |
| WHR (1 vs. 18)^1^ | -.01^4^ | .48 | -.01^5^ | <.001 | .01 | .92 |
| VAT (0 vs. 4)^1^ | n/a |  | -.19^5^ | <.001 |  | n/a |
|  | Consultation | | Food | | Diff. | |
| Blood pressure |  |  |  |  |  |  |
| SBP (49 vs.18)^2^ | -2.06 | <.001 | -1.17 | .05 | 1.41 | .23 |
| DBP (56 vs.14)^2^ | -1.01 | <.001 | -1.22 | <.001 | .20 | .66 |
| Lipid profile |  |  |  |  |  |  |
| TG (66 vs.25)^2^ | -12.50 | <.001 | -12.27 | <.001 | .01 | .93 |
| TC (63 vs.29)^2^ | 3.14 | <.001 | .53 | .72 | 2.15^5^ | .14 |
| LDL (77 vs.33)^2^ | 3.98 | <.001 | 2.26 | .10 | 1.13^5^ | .29 |
| HDL (81 vs.34)^2^ | 2.26 | <.001 | 2.40 | <.001 | .06 | .81 |
| LDL-HDL (2 vs.4)^2^ | -.25^4^ | <.001 | -.11 | .12 | 3.15 | .08 |
| TC-HDL (16 vs.9)^2^ | -.08 | .13 | -.16 | <.001 | 1.09 | .30 |
| Inflammatory markers |  |  |  |  |  |  |
| CRP (34 vs.6)^2^ | -.39 | <.001 | .35 | .24 | 5.08 | <.05 |
| IL-6 (5 vs.4)^2^ | -.23 | .57 | -.11 | .38 | .08 | .78 |
| Body composition |  |  |  |  |  |  |
| BW (77 vs. 34)^2^ | -1.76 | <.001 | -.88 | <.001 | 7.86 | <.05 |
| BMI (51 vs. 20)^2^ | -.61 | <.001 | -.34 | <.001 | 4.71 | <.05 |
| FM (34 vs. 14)^2^ | -.77 | <.001 | -.53 | <.05 | .82 | .36 |
| BFP (23 vs. 12)^2^ | -.81 | <.001 | -.40 | <.05 | 2.78 | .10 |
| LM (19 vs. 7)^2^ | .01 | .92 | -.16 | <.001 | 1.69 | .19 |
| FFM (14 vs. 5)^2^ | -.48 | <.001 | -.33 | .22 | .24 | .62 |
| WC (49 vs. 16)^2^ | -1.96 | <.001 | -1.02 | <.05 | 4.29^1^ | <.05 |
| HC (8 vs. 3)^2^ | -1.32 | <.001 | -.36 | .52 | 1.97 | .16 |
| WHR (16 vs. 3)^2^ | -.01^5^ | <.001 | -.02 | .15 | .42 | .52 |
|  | Non-isocaloric | | Isocaloric | | Diff. | |
|  | SMD | p | SMD | p | Qb(1) | p |
| Blood pressure |  |  |  |  |  |  |
| SBP (24 vs.24)^3^ | -1.97 | <.001 | -1.93 | <.001 | .00 | .96 |
| DBP (35 vs.26)^3^ | -1.06^5^ | <.05 | -1.03^5^ | <.001 | .00 | .97 |
| Lipid profile |  |  |  |  |  |  |
| TG (38 vs.40)^3^ | -14.28 | <.001 | -10.51 | <.001 | 2.06 | .15 |
| TC (41 vs.39)^3^ | 2.29 | .08 | 2.01 | .09 | .02 | .88 |
| LDL (46 vs.50)^3^ | 4.38^5^ | <.001 | 2.70 | <.05^5^ | 1.15 | .28 |
| HDL (52 vs.49)^3^ | 1.84 | <.001 | 2.58 | <.001 | 2.01 | .16 |
| TC-HDL (11 vs.9)^3^ | -.09 | .16 | -.08 | .13 | .02 | .86 |
| Inflammatory markers |  |  |  |  |  |  |
| CRP (16 vs.19)^3^ | -.22 | .24 | -.25 | .22 | .01 | .92 |
| Body composition |  |  |  |  |  |  |
| BW (31 vs. 34)^3^ | -1.02 | <.001 | -.78 | <.001 | .52 | .47 |
| BMI (21 vs. 25)^3^ | -.57 | <.001 | -.21 | <.001 | 6.27 | <.05 |
| FM (13 vs. 18)^3^ | -.54 | <.001 | -.46 | <.05 | .08 | .76 |
| BFP (13 vs. 13)^3^ | -.33^5^ | <.001 | -.76 | <.001 | 3.29 | .07 |
| LM (4 vs. 8)^3^ | -.15 | .16 | -.01 | .90 | 1.06 | .30 |
| FFM (7 vs. 6)^3^ | -.08 | .55 | -.38^5^ | .08 | 1.33 | .25 |
| WC (10 vs. 23)^3^ | -.28^5^ | .51 | -.78^5^ | <.001 | 1.20 | .27 |
| WHR (4 vs. 7)^3^ | -.01^5^ | <.001 | -.00 | .03 | 3.39 | .07 |

Note. ^1^(k vs. k) stands for the number of observations included for each associated group: Crossover trials vs. Parallel trials

^2^(k vs. k) stands for the number of observations included for each associated group: Consultation-only interventions vs. Food-provided intervention

^3^(k vs. k) stands for the number of observations included for each associated group: Non-isocaloric Diets vs. Isocaloric Diets.

^4^represents the number analyzed by fewer than 4 observations for the particular group.

^5^refers to deviations in the statistical significance from the original analyses

Abbreviations:

Diff. stands for group difference. n/a refers to not applicable due to insufficient data for analysis.

SMD for standardized mean difference. Crossover refers to crossover designs. Parallel refers to parallel designs. Consultation represents studies with consultation-only interventions.

Food refers to interventions with food provided. SBP: Systolic Blood Pressure; DBP: Diastolic Blood Pressure; TG: Triglycerides; TC: Total Cholesterol; LDL: Low-Density Lipoprotein; HDL: High-Density Lipoprotein; non-HDL: Non–High-Density Lipoprotein Cholesterol; CRP: C-Reactive Protein; IL-6: Interleukin-6; BW stands for body weight (kg); BMI stands for body weight index; FM stands for fat mass (kg); BFP stands for body fat percentage; LM stands for lean mass (kg); FFM stands for fat-free mass (kg); WC stands for waist circumference (cm); HC stands for hip circumference (cm); WHR stands for waist-hip ratio.
