# Supplementary material for: Effects of carbohydrate-restricted diets and macronutrient replacements on cardiovascular health and body composition in adults: a meta-analysis of randomized trials
Source: Am J Clin Nutr. 2025 Sep 8;122(5):1461–78. doi: 10.1016/j.ajcnut.2025.09.012 (PMC12799388; doi:10.1016/j.ajcnut.2025.09.012)
Supplement: Multimedia component 3 [file mmc3.pdf]

Effects of carbohydrate-restricted diets and macronutrient replacements on cardiovascular health and body composition in adults: A meta-analysis of randomized trials; Feng, Shuo

Supplementary Figures

Supplementary Figure 1. Risk of Bias Results ---2

Supplementary Figure 2. Forest Plots --4

Supplementary Figure 3. Funnel Plots ---37

Effects of carbohydrate-restricted diets and macronutrient replacements on cardiovascular health and body composition in adults: A meta-analysis of randomized trials; Feng, Shuo

Supplementary Figure 1. Risk of Bias Results

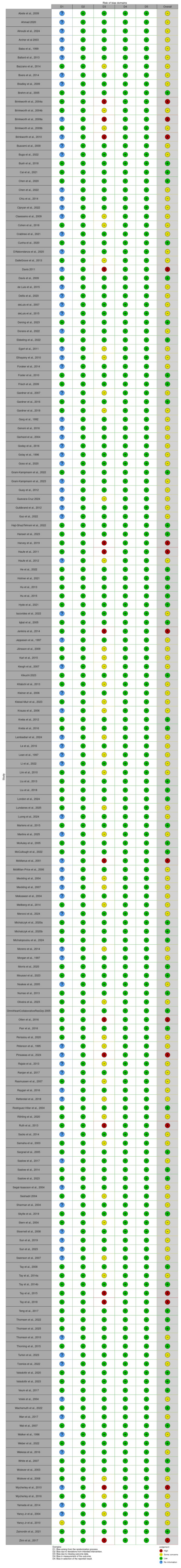
Risk of bias domains
D1
D2
D3
D4
D5
Overall
Study
Domains:
D1: Bias arising from the randomization process.
D2: Bias due to deviations from intended intervention.
D3: Bias due to missing outcome data.
D4: Bias in measurement of the outcome.
D5: Bias in selection of the reported result.
Judgement
High
Some concerns
Low
No information

Effects of carbohydrate-restricted diets and macronutrient replacements on cardiovascular health and body composition in adults: A meta-analysis of randomized trials; Feng, Shuo

Supplementary Figures - Forest plots of Effect Sizes of CRDs on Cardiovascular Health and Body Composition Outcomes

Study	Treatment N	Treatment Mean	Treatment SD	Control N	Control Mean	Control SD	Mean diff. with 95% CI	Weight (%)
Kleissl-Muir et al., 2023	8	-5.4	6.94	5	18	15.5	-23.40 [-35.54, -11.26]	0.50
Morris et al., 2020	21	-9.6	16.2	12	4.8	10.6	-14.40 [-24.66, -4.14]	0.61
Ruth et al., 2013	18	-8.8	14.1	15	2.5	12.4	-11.30 [-20.45, -2.15]	0.69
Chen et al., 2022	36	-8.7	3.3	35	1.9	.8	-10.60 [-11.72, -9.48]	1.33
Abete et al., 2009	19	-13.3	11.2	19	-3	.1	-10.30 [-15.34, -5.26]	1.05
McAuley et al., 2005	31	-12	.001	30	-2	1	-10.00 [-10.35, -9.65]	1.35
Chen et al., 2020	43	-8.26	2.39	42	1.62	1.91	-9.88 [-10.80, -8.96]	1.34
Jönsson et al., 2009	13	140	12	13	149	22	-9.00 [-22.62, 4.62]	0.43
McCullough et al., 2022	8	-7	1	8	2	2	-9.00 [-10.55, -7.45]	1.31
Brinkworth et al., 2004a	19	-4.6	.44	19	3.7	.87	-8.30 [-8.74, -7.86]	1.35
McAuley et al., 2005	30	-12	.001	31	-4	2	-8.00 [-8.72, -7.28]	1.34
Alnoubi et al., 2024	14	-8.8	2.5	14	-1.1	3.7	-7.70 [-10.04, -5.36]	1.27
Ahmad 2020	25	-1.4	11.5	25	6.3	1	-7.70 [-12.22, -3.18]	1.09
YancyWSJr et al., 2010	57	-5.94	1.46	65	1.5	1.21	-7.44 [-7.91, -6.97]	1.35
Pinsawas et al., 2024	26	-2.3	4.89	22	5.1	6.1	-7.40 [-10.51, -4.29]	1.22
Gardner et al., 2016	16	-8.8	10.1	15	-2.2	9	-6.60 [-13.35, 0.15]	0.89
Claessens et al., 2009	32	-4.94	4.61	16	1.63	7.28	-6.57 [-9.94, -3.20]	1.19
Brehm et al., 2005	20	-9	.45	20	-3	2.68	-6.00 [-7.19, -4.81]	1.33
Retterstøl et al., 2018	15	2	.8	15	8	2.5	-6.00 [-7.33, -4.67]	1.32
Gardner et al., 2007	77	-7.6	11	76	-1.9	7.7	-5.70 [-8.71, -2.69]	1.22
Mellberg et al., 2014	27	-3.7	18.19	22	1.7	10.32	-5.40 [-13.95, 3.15]	0.73
Skytte et al., 2019	14	-4.1	6.73	14	1.3	7.11	-5.40 [-10.53, -0.27]	1.04
Liu et al., 2013	25	-20.3	6.66	24	-15	2.45	-5.30 [-8.13, -2.47]	1.24
Meksawan et al., 2004	10	107.2	14.55	10	112.3	12.65	-5.10 [-17.05, 6.85]	0.51
Otten et al., 2016	25	1	13	16	6	6	-5.00 [-11.81, 1.81]	0.88
Wolever et al., 2008	53	0	14.97	55	5	22.25	-5.00 [-12.18, 2.18]	0.85
Lim et al., 2010	18	-10.6	44.97	17	-6	54.84	-4.60 [-37.74, 28.54]	0.10
Saslow et al., 2023	23	-9.77	.37	25	-5.18	.55	-4.59 [-4.86, -4.32]	1.35
Boers et al., 2014	18	-8.5	5	16	-4.2	5.6	-4.30 [-7.86, -0.74]	1.18
Frisch et al., 2009	85	-5	14	80	-1	15	-4.00 [-8.42, 0.42]	1.10
Kikuchi 2023	21	-8	11.85	21	-4	5.56	-4.00 [-9.60, 1.60]	0.99
Dorans et al., 2022	75	-4.9	1.56	75	-1.6	1.43	-3.30 [-3.78, -2.82]	1.35
Ebbeling et al., 2022	53	-1.5	1.51	46	1.8	1.61	-3.30 [-3.92, -2.68]	1.34
Genoni et al., 2016	22	-4	2.96	17	-.71	2.3	-3.29 [-5.00, -1.58]	1.31
Wekesa et al., 2016	24	127	14.7	24	130	9.8	-3.00 [-10.07, 4.07]	0.86
de Luis et al., 2015	168	-5.1	7.1	163	-2.5	2.1	-2.60 [-3.74, -1.46]	1.33
Foster et al., 2010	153	-7.74	.94	154	-5.2	.96	-2.54 [-2.75, -2.33]	1.35
Tay et al., 2014b	46	-11	10.6	47	-8.7	12.5	-2.30 [-7.02, 2.42]	1.08
Michalopoulou et al., 2024	28	-1.2	1.81	17	1.1	1.53	-2.30 [-3.33, -1.27]	1.33
Kleiner et al., 2006	9	1	1.94	7	3	5.29	-2.00 [-5.71, 1.71]	1.17
Zainordin et al., 2021	14	-13	8.1	16	-11	7.08	-2.00 [-7.43, 3.43]	1.01
Hansen et al., 2023	110	-5.5	2.07	55	-3.7	3.19	-1.80 [-2.61, -0.99]	1.34
Mousavi et al., 2023	35	-2.67	3.49	35	-1.07	3.49	-1.60 [-3.24, 0.04]	1.31
Tay et al., 2008	45	-12.3	2.9	43	-10.8	3.2	-1.50 [-2.77, -0.23]	1.33
Sacks et al., 2014	150	122.6	10.3	150	123.9	11.5	-1.30 [-3.77, 1.17]	1.26
OmniHeartCollaborativeResGrp 2005	164	-9.5	.69	164	-8.2	.71	-1.30 [-1.45, -1.15]	1.35
Brinkworth et al., 2004b	21	1.7	4.14	22	3	.94	-1.30 [-3.08, 0.48]	1.30
Tay et al., 2014a	58	-7.1	1.76	57	-5.8	1.84	-1.30 [-1.96, -0.64]	1.34
Tay et al., 2015	41	-7.1	1.76	37	-5.8	1.84	-1.30 [-2.10, -0.50]	1.34
OmniHeartCollaborativeResGrp 2005	164	-9.3	.66	164	-8.2	.71	-1.10 [-1.25, -0.95]	1.35
Jenkins et al., 2014	20	-5	.001	19	-4	1.02	-1.00 [-1.45, -0.55]	1.35
Röhling et al., 2020	65	-6	2.04	28	-5	3.06	-1.00 [-2.06, 0.06]	1.33
Stern et al., 2004	44	1	4	43	2	2	-1.00 [-2.33, 0.33]	1.32
Ranjan et al., 2017	10	124.9	13.7	10	125.6	5.5	-0.70 [-9.85, 8.45]	0.69
Holmer et al., 2021	22	-6.4	2.17	24	-5.8	3.7	-0.60 [-2.37, 1.17]	1.30
Gardner et al., 2018	304	-3.72	.58	305	-3.18	.59	-0.54 [-0.63, -0.45]	1.35
Perissiou et al., 2020	33	-3.7	7	31	-3.3	10	-0.40 [-4.61, 3.81]	1.12
Sacks et al., 2014	151	123.4	10.1	151	123.8	10.5	-0.40 [-2.72, 1.92]	1.27
DalleGrave et al., 2013	43	-10.3	21.5	45	-10.1	19.1	-0.20 [-8.69, 8.29]	0.74
Bradley et al., 2009	12	-8	2	12	-8	4	0.00 [-2.53, 2.53]	1.26
He et al., 2022	44	1	13.27	44	1	13.27	0.00 [-5.55, 5.55]	1.00
Meckling et al., 2007	10	-9	6	8	-9	2	0.00 [-4.36, 4.36]	1.11
Turton et al., 2023	16	126.7	17.3	16	126.7	12.1	0.00 [-10.34, 10.34]	0.61
Teng et al., 2017	18	109.5	1.58	18	109.4	1.53	0.10 [-0.92, 1.12]	1.33
Wan et al., 2017	101	-2.4	.56	101	-2.6	.51	0.20 [0.05, 0.35]	1.35
Lim et al., 2010	18	-5.4	56.43	17	-6	54.84	0.60 [-36.30, 37.50]	0.08
Saslow et al., 2014	15	5.3	.7	18	4.7	1.2	0.60 [-0.09, 1.29]	1.34
Brinkworth et al., 2009	33	-13.8	2.31	36	-14.6	4.8	0.80 [-1.00, 2.60]	1.30
Lean et al., 1997	36	-.3	2.98	38	-1.1	2.81	0.80 [-0.52, 2.12]	1.32
Buscemi et al., 2009	10	-7	3.16	10	-8	15.81	1.00 [-8.99, 10.99]	0.63
deLuis et al., 2015	49	-4.7	4.9	45	-5.7	1.9	1.00 [-0.53, 2.53]	1.32
Meckling et al., 2004	15	-10.2	3.43	16	-11.2	2.8	1.00 [-1.20, 3.20]	1.28
Veum et al., 2017	20	-14	1.4	18	-15	3.9	1.00 [-0.83, 2.83]	1.30
Wolever et al., 2008	53	0	13.5	48	-1	6.93	1.00 [-3.25, 5.25]	1.12
Bazzano et al., 2014	59	-.2	1.2	60	-1.3	1.17	1.10 [0.67, 1.53]	1.35
Krebs et al., 2012	152	-.2	2.5	158	-1.3	.2	1.10 [0.71, 1.49]	1.35
Tay et al., 2018	33	-2	1.96	28	-3.2	2.09	1.20 [0.18, 2.22]	1.33
Yamada et al., 2014	12	-1.9	1.1	12	-3.6	.9	1.70 [0.90, 2.50]	1.34
Guldbrand et al., 2012	30	-9	1	31	-11	.001	2.00 [1.65, 2.35]	1.35
Gardner et al., 2016	14	-4.4	8.1	16	-6.9	10.2	2.50 [-4.16, 9.16]	0.89
Gram-Kampmann et al., 2022	44	-3	4.32	20	-6	.001	3.00 [1.10, 4.90]	1.30
Hyde et al., 2021	12	-2	.01	12	-5	.001	3.00 [2.99, 3.01]	1.35
Davis et al., 2009	55	2	15.6	50	-1.8	22.6	3.80 [-3.57, 11.17]	0.83
Saslow et al., 2017	16	3.2	.05	18	-1.7	.15	4.90 [4.82, 4.98]	1.35
deLuis et al., 2015	61	-6.2	1.1	56	-11.2	.4	5.00 [4.69, 5.31]	1.35
Thomson et al., 2010	19	-.8	14.1	21	-8.6	16.3	7.80 [-1.69, 17.29]	0.66
Merovci et al., 2024	10	0	2.16	8	-11	5.66	11.00 [7.21, 14.79]	1.16
Foraker et al., 2014	38	-2	1.07	41	-22.2	.64	20.20 [19.81, 20.59]	1.35
**Overall**							-2.05 [-3.13, -0.96]	

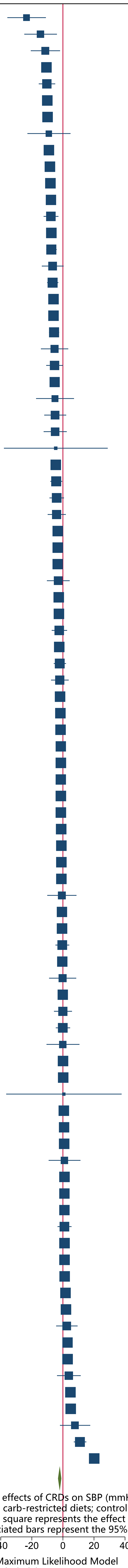

Heterogeneity: τ^2 = 22.63, I^2 = 99.91%, H^2 = 1084.90

Test of $\theta_i = \theta_j$: Q(87) = 44186.17, p = 0.00

Test of θ = 0: z = -3.70, p = 0.00

Forest plot depicting the effects of CRDs on SBP (mmHg)
Note. Treatment involves carb-restricted diets; control refers to higher-carbohydrate diets. For each study, the square represents the effect size (standardized mean difference) and the associated bars represent the 95% CI.

Random-effects REML model
Sorted by: _meta_es

Restricted Randomized Maximum Likelihood Model
The number of studies included: k=88

Study	Treatment N	Treatment Mean	Treatment SD	Control N	Control Mean	Control SD	Mean diff. with 95% CI	Weight (%)
Pinsawas et al., 2024	26	-6.3	3.41	22	3.1	4.06	-9.40 [-11.51, -7.29]	1.19
McCullough et al., 2022	8	-8	1.27	8	0	1.56	-8.00 [-9.39, -6.61]	1.27
Chen et al., 2020	43	-5	1.51	42	2.45	1.69	-7.45 [-8.13, -6.77]	1.32
Brinkworth et al., 2004a	19	-4.9	2.4	19	2.5	2.17	-7.40 [-8.85, -5.95]	1.27
Chen et al., 2022	36	-5	3.12	35	1.4	1.56	-6.40 [-7.55, -5.25]	1.29
Ruth et al., 2013	18	-5.2	8.2	15	.9	9.5	-6.10 [-12.14, -0.06]	0.66
McAuley et al., 2005	30	-7	1.41	31	-1	1.67	-6.00 [-6.78, -5.22]	1.32
Otten et al., 2016	25	-3	2.37	16	3	1.36	-6.00 [-7.28, -4.72]	1.28
Morris et al., 2020	21	-5.3	11	12	.5	8.8	-5.80 [-13.09, 1.49]	0.54
Claessens et al., 2009	32	-2.44	1.49	16	2.63	10.18	-5.07 [-8.63, -1.51]	0.99
DalleGrave et al., 2013	43	-6.4	11	45	-1.4	9.9	-5.00 [-9.37, -0.63]	0.87
Wekesa et al., 2016	24	79	9.8	24	84	9.8	-5.00 [-10.54, 0.54]	0.72
YancyWSJr et al., 2010	57	-4.53	1.04	65	.43	.91	-4.96 [-5.31, -4.61]	1.34
Brinkworth et al., 2004b	7	-6.2	10.12	12	-1.4	3.81	-4.80 [-11.09, 1.49]	0.63
Yamada et al., 2014	12	-6	3.38	12	-1.4	1.43	-4.60 [-6.68, -2.52]	1.20
Boers et al., 2014	18	-8	3.17	16	-3.5	4.28	-4.50 [-7.01, -1.99]	1.14
Krebs et al., 2016	5	-7.6	4.76	5	-3.1	3.36	-4.50 [-9.61, 0.61]	0.77
Ahmad 2020	25	-2.1	2.62	25	2	5.26	-4.10 [-6.40, -1.80]	1.17
Ballard et al., 2013	21	75	1.56	21	79	9	-4.00 [-7.91, -0.09]	0.94
Jönsson et al., 2009	13	79	6	13	83	9	-4.00 [-9.88, 1.88]	0.68
Gardner et al., 2007	77	-4.4	8.4	76	-.7	6	-3.70 [-6.02, -1.38]	1.16
Genoni et al., 2016	22	-1.9	2.32	17	1.7	1.35	-3.60 [-4.84, -2.36]	1.29
Perissiou et al., 2020	33	-4.4	7	31	-1	8	-3.40 [-7.08, 0.28]	0.97
Mellberg et al., 2014	27	-4.8	7.79	22	-1.5	8.44	-3.30 [-7.85, 1.25]	0.85
Skytte et al., 2019	14	-2.9	3.74	14	.2	4.12	-3.10 [-6.01, -0.19]	1.08
Liu et al., 2013	25	-10.8	1.72	24	-7.8	1.96	-3.00 [-4.03, -1.97]	1.30
Brehm et al., 2005	20	-5	2.46	20	-2	5.29	-3.00 [-5.56, -0.44]	1.13
He et al., 2022	44	-5	13.27	44	-2	6.63	-3.00 [-7.38, 1.38]	0.87
McAuley et al., 2005	31	-7	1.41	30	-4	2.29	-3.00 [-3.95, -2.05]	1.31
Gardner et al., 2016	16	-5.3	7.5	15	-2.4	6.8	-2.90 [-7.95, 2.15]	0.78
Dorans et al., 2022	75	-3.2	1.02	75	-.6	.88	-2.60 [-2.90, -2.30]	1.34
Hansen et al., 2023	110	-1.2	1.2	55	1.4	1.99	-2.60 [-3.09, -2.11]	1.33
Kleissl-Muir et al., 2023	8	-2.5	2.84	5	0	21.86	-2.50 [-17.44, 12.44]	0.18
Foster et al., 2010	153	-5.53	.6	154	-3.05	.63	-2.48 [-2.62, -2.34]	1.34
Hyde et al., 2021	12	-4	1.39	12	-2	1.39	-2.00 [-3.11, -0.89]	1.30
Keogh et al., 2007	13	-6	1.44	12	-4	3.6	-2.00 [-4.12, 0.12]	1.19
Kitabchi et al., 2013	12	-9	.9	12	-7	1.61	-2.00 [-3.04, -0.96]	1.30
deLuis et al., 2015	61	-2.2	3.18	56	-.3	1.63	-1.90 [-2.83, -0.97]	1.31
Tay et al., 2014b	46	-8.2	5.6	47	-6.4	7.8	-1.80 [-4.56, 0.96]	1.10
deLuis et al., 2015	49	-5.2	1.67	45	-3.7	4.16	-1.50 [-2.76, -0.24]	1.28
Lim et al., 2010	18	-9	9.3	17	-7.5	8.7	-1.50 [-7.48, 4.48]	0.67
OmniHeartCollaborativeResGrp 2005	164	-5.2	.43	164	-4.1	.43	-1.10 [-1.19, -1.01]	1.34
Meckling et al., 2004	15	-6.1	3.56	16	-5	3.27	-1.10 [-3.50, 1.30]	1.15
Frisch et al., 2009	85	-3	9	80	-2	8	-1.00 [-3.60, 1.60]	1.13
Kleiner et al., 2006	9	-3	3.34	7	-2	3.22	-1.00 [-4.25, 2.25]	1.03
Meckling et al., 2007	10	-7	6.15	8	-6	3.48	-1.00 [-5.79, 3.79]	0.81
Michalopoulou et al., 2024	28	-9.3	1.45	17	-8.3	1.66	-1.00 [-1.92, -0.08]	1.31
Valsdottir et al., 2023	14	-3	.71	15	-2	2.37	-1.00 [-2.29, 0.29]	1.28
Ranjan et al., 2017	10	75.1	9.4	10	76	4.8	-0.90 [-7.44, 5.64]	0.61
Gardner et al., 2018	304	-2.64	.36	305	-1.94	.36	-0.70 [-0.76, -0.64]	1.34
OmniHeartCollaborativeResGrp 2005	164	-4.8	.41	164	-4.1	.43	-0.70 [-0.79, -0.61]	1.34
Bazzano et al., 2014	59	-.5	.89	60	.2	.87	-0.70 [-1.02, -0.38]	1.34
Davis et al., 2009	55	-2.9	9.4	50	-2.2	11.6	-0.70 [-4.72, 3.32]	0.92
Zainordin et al., 2021	14	-1.5	4.81	16	-1	2.81	-0.50 [-3.28, 2.28]	1.10
Mousavi et al., 2023	35	-1.63	2.25	35	-1.16	2.25	-0.47 [-1.52, 0.58]	1.30
Teng et al., 2017	18	79.38	1.43	18	79.73	1.3	-0.35 [-1.24, 0.54]	1.31
Holmer et al., 2021	22	-4	1.86	24	-3.8	2.07	-0.20 [-1.34, 0.94]	1.29
Ebbeling et al., 2022	53	2.2	1.33	46	2.2	1.43	0.00 [-0.54, 0.54]	1.33
Jenkins et al., 2014	20	-1	.86	19	-1	.42	0.00 [-0.43, 0.43]	1.33
Wan et al., 2017	101	-1.1	.31	101	-1.3	.31	0.20 [0.11, 0.29]	1.34
Tay et al., 2014a	58	-6.2	1.05	57	-6.4	1.05	0.20 [-0.18, 0.58]	1.34
Tay et al., 2015	41	-6.2	1.05	37	-6.4	1.05	0.20 [-0.27, 0.67]	1.33
Krebs et al., 2012	152	-.1	1.66	158	-.5	1.56	0.40 [0.04, 0.76]	1.34
Lean et al., 1997	36	-2.3	1.94	38	-2.7	1.91	0.40 [-0.48, 1.28]	1.31
Abete et al., 2009	19	-5	1.25	19	-5.5	1.38	0.50 [-0.34, 1.34]	1.32
de Luis et al., 2015	168	-.9	5.4	163	-1.5	2.51	0.60 [-0.31, 1.51]	1.31
Thomson et al., 2010	19	1.6	11	21	.9	6.9	0.70 [-4.93, 6.33]	0.71
Tay et al., 2018	33	-1.2	1.22	28	-2	1.28	0.80 [0.17, 1.43]	1.33
Lim et al., 2010	18	-6.6	12.1	17	-7.5	8.7	0.90 [-6.12, 7.92]	0.56
Tay et al., 2008	45	-4.6	1.65	43	-5.5	1.73	0.90 [0.19, 1.61]	1.32
Bradley et al., 2009	12	-5	1.45	12	-6	2.29	1.00 [-0.53, 2.53]	1.26
Gram-Kampmann et al., 2022	44	-1	1.33	20	-2	1.79	1.00 [0.21, 1.79]	1.32
Guldbrand et al., 2012	30	-5	3.28	31	-6	2.45	1.00 [-0.45, 2.45]	1.27
Kikuchi 2023	21	-5	7.41	21	-6	8.52	1.00 [-3.83, 5.83]	0.81
Röhling et al., 2020	65	-3	1.02	28	-4	1.53	1.00 [0.47, 1.53]	1.33
Saslow et al., 2017	16	-1.5	.32	18	-2.7	.3	1.20 [0.99, 1.41]	1.34
Foraker et al., 2014	38	.5	1.77	41	-1.1	2.58	1.60 [0.62, 2.58]	1.31
Brinkworth et al., 2009	33	-6.3	2.46	36	-7.9	2.18	1.60 [0.51, 2.69]	1.30
Stern et al., 2004	44	3	3.48	43	1	1.67	2.00 [0.85, 3.15]	1.29
Gardner et al., 2016	14	-4.3	5.6	16	-6.4	7.1	2.10 [-2.52, 6.72]	0.84
Brinkworth et al., 2004b	15	2.4	1.86	31	.2	3.81	2.20 [0.16, 4.24]	1.20
Turton et al., 2023	16	74.1	6.3	16	71.8	6.9	2.30 [-2.28, 6.88]	0.84
Saslow et al., 2014	15	2.6	.97	18	.3	5.08	2.30 [-0.32, 4.92]	1.12
Buscemi et al., 2009	10	-1	1.9	10	-5	3.52	4.00 [1.52, 6.48]	1.14
Alnoubi et al., 2024	14	.3	3.92	14	-4.8	2.43	5.10 [2.68, 7.52]	1.15
Meksawan et al., 2004	10	72.2	9.8	10	66.7	5.06	5.50 [-1.34, 12.34]	0.58
Veum et al., 2017	20	-2.3	2.32	18	-8	1.75	5.70 [4.38, 7.02]	1.28
Merovci et al., 2024	10	-1	1.9	8	-7	1.7	6.00 [4.31, 7.69]	1.24
Retterstøl et al., 2018	15	2	1.26	15	-6	2.61	8.00 [6.53, 9.47]	1.26
**Overall**							-1.26 [-1.94, -0.57]	

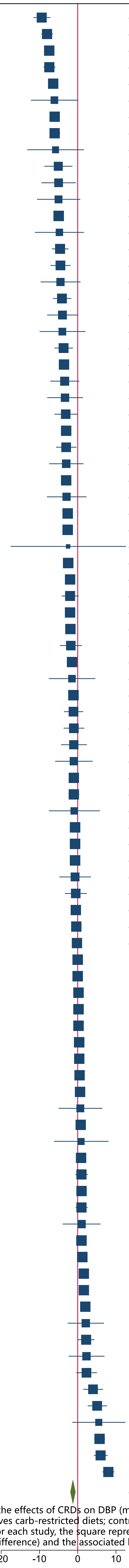

Heterogeneity: τ^2 = 9.20, I^2 = 99.64%, H^2 = 277.08

Test of $\theta_i = \theta_j$: Q(88) = 3991.85, p = 0.00

Test of θ = 0: z = -3.58, p = 0.00

Forest plot depicting the effects of CRDs on DBP (mmHg)
Note. Treatment involves carb-restricted diets; control refers to higher-carbohydrate diets. For each study, the square represents the effect size (standardized mean difference) and the associated bars represent the 95% CI.

Random-effects REML model
Sorted by: _meta_es

Restricted Randomized Maximum Likelihood Model
The number of studies included: k=89

Study	Treatment N	Treatment Mean	Treatment SD	Control N	Control Mean	Control SD	Mean diff. with 95% CI	Weight (%)
Boers et al., 2014	18	-79.713	71.7417	16	8.857	35.428	-88.57 [-127.39, -49.75]	0.45
Michalczyk et al., 2020b	46	-84.32	18.99	45	-4.13	14.86	-80.19 [-87.21, -73.17]	0.87
Michalczyk et al., 2020a	18	-77.01	9.25	20	1.93	7.18	-78.94 [-84.18, -73.70]	0.89
Garg et al., 1992	8	-129.3122	93.8842	8	-67.3132	100.9698	-62.00 [-157.54, 33.54]	0.13
Stern et al., 2004	44	-57.5705	157.6546	43	4.4285	85.0272	-62.00 [-115.40, -8.59]	0.31
Jeppesen et al., 1997	10	114.2553	89.4557	10	174.4829	178.9114	-60.23 [-184.20, 63.75]	0.08
Elhayany et al., 2010	61	-134.6264	38.0851	55	-77.9416	30.1138	-56.68 [-69.27, -44.10]	0.83
Wekesa et al., 2016	24	26.571	43.3993	24	81.4844	25.6853	-54.91 [-75.09, -34.74]	0.71
Ahmad 2020	25	-30.9995	54.0277	25	22.1425	9.7427	-53.14 [-74.66, -31.62]	0.69
Yamada et al., 2014	12	-58.2	34.46	12	-6.8	13.24	-51.40 [-72.29, -30.51]	0.70
Claessens et al., 2009	32	-2.6571	12.3998	16	48.7135	85.9129	-51.37 [-81.44, -21.30]	0.56
Morris et al., 2020	21	-43.3993	61.999	12	7.9713	53.142	-51.37 [-93.22, -9.52]	0.41
Bradley et al., 2009	12	-60.2276	20.3711	12	-10.6284	30.9995	-49.60 [-70.59, -28.61]	0.69
Numao et al., 2013	11	-38.0851	23.9139	11	11.5141	3.5428	-49.60 [-63.89, -35.31]	0.79
Skytte et al., 2019	14	-38.0851	39.8565	14	10.6284	28.3424	-48.71 [-74.33, -23.10]	0.62
Sharman et al., 2004	15	71.7417	21.2568	15	116.9124	45.1707	-45.17 [-70.43, -19.91]	0.63
Ruth et al., 2013	18	-33.2	37.3	15	11.3	35.3	-44.50 [-69.45, -19.55]	0.63
Jönsson et al., 2009	13	88.57	44.285	13	132.855	61.999	-44.28 [-85.70, -2.87]	0.42
Luong et al., 2024	11	79.713	17.714	11	123.998	61.999	-44.28 [-82.39, -6.18]	0.45
Thomsen et al., 2025	28	-28	4.34	28	14	7.14	-42.00 [-45.09, -38.91]	0.90
Guay et al., 2012	12	-16.8283	17.714	12	24.7996	18.5997	-41.63 [-56.16, -27.10]	0.79
Liu et al., 2013	25	-79.713	84.1415	24	-38.0851	7.9713	-41.63 [-75.45, -7.81]	0.51
Moreno et al., 2014	26	-48.3	17.55	27	-8.8	11.55	-39.50 [-47.47, -31.53]	0.86
Thomson et al., 2010	19	-31.1	36.6	21	7.2	67.1	-38.30 [-72.31, -4.29]	0.51
Lim et al., 2010	18	-26.571	300.2523	17	8.857	328.5947	-35.43 [-243.77, 172.92]	0.03
Perissiou et al., 2020	23	-35.428	61.999	31	0	44.285	-35.43 [-61.98, -8.88]	0.61
Tay et al., 2014b	46	-44.285	44.285	47	-8.857	44.285	-35.43 [-53.43, -17.43]	0.74
Baba et al., 1999	7	-110.1	82.27	6	-75.5	44.63	-34.60 [-108.53, 39.33]	0.19
Tay et al., 2015	61	-35.428	7.0856	54	-.8857	8.857	-34.54 [-38.09, -31.00]	0.89
Cipryan et al., 2022	25	-24.7996	10.6284	19	8.857	15.9426	-33.66 [-41.51, -25.80]	0.87
Keogh et al., 2007	13	-58.4562	122.2266	12	-24.7996	26.571	-33.66 [-104.41, 37.10]	0.21
McAuley et al., 2005	30	-61.1133	37.1994	31	-27.4567	8.857	-33.66 [-47.13, -20.19]	0.80
Brehm et al., 2005	20	-48.1	33.28	20	-14.98	31.02	-33.12 [-53.06, -13.18]	0.71
Stoernell et al., 2008	10	-25.6853	7.9713	10	7.0856	46.0564	-32.77 [-61.79, -3.75]	0.57
Brinkworth et al., 2009	33	-51.3706	13.2855	36	-19.4854	29.2281	-31.89 [-42.77, -21.01]	0.83
Samaha et al., 2003	64	-38	25.04	68	-7	19.08	-31.00 [-38.57, -23.43]	0.87
Wycherley et al., 2010	26	-50.4849	54.0277	23	-21.2568	63.7704	-29.23 [-62.21, 3.76]	0.52
Wachsmuth et al., 2022	18	-1	4.06	18	28	8.33	-29.00 [-33.28, -24.72]	0.89
Turton et al., 2023	16	70.856	17.714	16	97.427	35.428	-26.57 [-45.98, -7.16]	0.72
Kleissl-Muir et al., 2023	8	-35.428	29.2281	5	-8.857	29.2281	-26.57 [-59.23, 6.09]	0.52
Lim et al., 2010	18	-17.714	263.0529	17	8.857	328.5947	-26.57 [-223.20, 170.06]	0.03
Alnoubi et al., 2024	14	-21.5	23.25	14	4.5	11.02	-26.00 [-39.48, -12.52]	0.80
Archer et al 2003	32	-24.7996	8.857	31	.8857	12.3998	-25.69 [-30.99, -20.38]	0.88
Tay et al., 2008	45	-56.6848	30.9995	43	-30.9995	18.5997	-25.69 [-36.43, -14.94]	0.84
Jenkins et al., 2014	20	-64.6561	14.1712	19	-39.8565	8.857	-24.80 [-32.26, -17.34]	0.87
Saslow et al., 2017	16	-9.9	1.69	18	14.5	3.53	-24.40 [-26.30, -22.50]	0.90
Gardner et al., 2016	14	-32.2	41.4	16	-9.6	27.9	-22.60 [-47.58, 2.38]	0.63
Foster et al., 2010	153	-40.08	2.63	154	-17.99	3.39	-22.09 [-22.77, -21.41]	0.90
Segal-Isaacson et al., 2004	4	53	11	4	75	20	-22.00 [-44.37, 0.37]	0.67
Sacks et al., 2014	151	86.4	48.1	151	107.4	59.2	-21.00 [-33.17, -8.83]	0.82
Lambadiari et al., 2024	16	106.9	29.24	16	127.82	47.95	-20.92 [-48.44, 6.60]	0.60
Mousavi et al., 2023	35	-26.8	14.08	35	-7.19	14.08	-19.61 [-26.21, -13.01]	0.88
Mellberg et al., 2014	27	-20.3711	31.8852	22	-.8857	24.7996	-19.49 [-35.77, -3.20]	0.76
Sacks et al., 2014	150	91.4	48.1	150	110.8	65.7	-19.40 [-32.43, -6.37]	0.81
He et al., 2022	44	-45.1707	1180.6381	44	-26.571	798.9014	-18.60 [-439.81, 402.61]	0.01
Thorning et al., 2015	14	-14.1712	7.9713	14	4.4285	14.1712	-18.60 [-27.12, -10.08]	0.86
Saslow et al., 2014	15	-22.3	22.51	18	-3.9	17.85	-18.40 [-32.17, -4.63]	0.80
Goss et al., 2020	19	-39.3	25.43	15	-20.9	12.21	-18.40 [-32.42, -4.38]	0.80
Gardner et al., 2018	304	-28.2	3.81	305	-9.95	3.83	-18.25 [-18.86, -17.64]	0.90
Goday et al., 2016	45	-35.9	8.37	40	-17.8	32.76	-18.10 [-28.01, -8.19]	0.85
Gerhard et al., 2004	11	-45	66.6	11	-27	71.22	-18.00 [-75.62, 39.62]	0.28
Kitabchi et al., 2013	12	-26	25.42	12	-8	6.97	-18.00 [-32.91, -3.09]	0.78
Otten et al., 2016	25	-26.571	5.3142	16	-8.857	18.5997	-17.71 [-25.41, -10.02]	0.87
Parr et al., 2016	29	-44.285	115.141	32	-26.571	44.285	-17.71 [-60.71, 25.28]	0.40
Thomsen et al., 2022	34	-44.285	46.0564	32	-26.571	38.9708	-17.71 [-38.36, 2.93]	0.70
OmniHeartCollaborativeResGrp 2005	164	-16.4	4.64	164	.1	4.44	-16.50 [-17.48, -15.52]	0.90
Hansen et al., 2023	110	-32.7709	12.3998	55	-17.714	14.1712	-15.06 [-19.27, -10.84]	0.89
Li et al., 2022	24	-28.3424	29.2281	29	-13.2855	29.2281	-15.06 [-30.87, 0.75]	0.77
Gardner et al., 2007	77	-29.3	59	76	-14.9	46.2	-14.40 [-31.21, 2.41]	0.76
Wolever et al., 2008	53	12.3998	52.2563	55	26.571	21.2568	-14.17 [-29.12, 0.78]	0.78
DalleGrave et al., 2013	43	-45.6	57.2	45	-31.5	75.3	-14.10 [-42.13, 13.93]	0.59
Thomsen et al., 2025	34	-27	5.61	33	-13	5.87	-14.00 [-16.75, -11.25]	0.90
Chen et al., 2020	43	-31.4	12.32	42	-17.81	15.17	-13.59 [-19.46, -7.72]	0.88
Meksawan et al., 2004	10	72	34.79	10	85	31.62	-13.00 [-42.14, 16.14]	0.57
Davis et al., 2009	55	-13.2855	77.9416	50	-.8857	76.1702	-12.40 [-41.93, 17.13]	0.57
Harvey et al., 2019	14	-15.9426	6.1999	15	-3.5428	7.9713	-12.40 [-17.85, -6.95]	0.89
McMillan-Price et al., 2006	28	-16.8283	32.7709	30	-4.4285	33.6566	-12.40 [-29.52, 4.72]	0.75
Kleiner et al., 2006	9	7.3	42.36	7	19.6	12.68	-12.30 [-44.97, 20.37]	0.52
Chen et al., 2022	36	-28.3	14.84	35	-16.1	33.45	-12.20 [-24.18, -0.22]	0.82
Krebs et al., 2012	152	-9.7427	11.5141	158	1.7714	11.5141	-11.51 [-14.08, -8.95]	0.90
Zinn et al., 2017	14	-32.7709	15.9426	12	-21.2568	10.6284	-11.51 [-22.13, -0.90]	0.84
Genoni et al., 2016	22	-15.9426	18.5997	17	-5.3142	2.6571	-10.63 [-19.57, -1.69]	0.86
Veum et al., 2017	20	-46.9421	7.0856	18	-36.3137	8.857	-10.63 [-15.70, -5.55]	0.89
Cai et al., 2021	22	-30.9995	54.0277	22	-20.3711	41.6279	-10.63 [-39.13, 17.87]	0.58
Volek et al., 2004	13	69	31	13	79	36	-10.00 [-35.83, 15.83]	0.62
Seshadri 2004	43	-14.1712	3.5428	35	-4.4285	3.5428	-9.74 [-11.32, -8.16]	0.90
OmniHeartCollaborativeResGrp 2005	164	-9.3	4.16	164	.1	4.44	-9.40 [-10.33, -8.47]	0.90
Ranjan et al., 2017	10	61.999	17.714	10	70.856	8.857	-8.86 [-21.13, 3.42]	0.82
Guldbrand et al., 2012	30	-17.714	54.9134	31	-8.857	14.1712	-8.86 [-28.84, 11.12]	0.71
Haufe et al., 2012	41	-17.714	10.6284	49	-8.857	11.5141	-8.86 [-13.47, -4.24]	0.89
Weber et al., 2022	28	-35.428	13.2855	31	-26.571	13.2855	-8.86 [-15.65, -2.07]	0.88
Martins et al., 2025	23	2.3	25.09	21	11	12.98	-8.70 [-20.68, 3.28]	0.82
de Luis et al., 2015	168	-14	38.53	163	-6.4	5.68	-7.60 [-13.58, -1.62]	0.88
Raygan et al., 2016	28	6.7	6.5	28	14.2	40.97	-7.50 [-22.87, 7.87]	0.78
YancyWSJr et al., 2010	57	-28.83	9.82	65	-21.4	9.3	-7.43 [-10.83, -4.03]	0.89
Le et al., 2016	81	-20	19.51	82	-13	20.17	-7.00 [-13.09, -0.91]	0.88
Valsdottir et al., 2020	14	-5.3142	3.5428	15	.8857	13.2855	-6.20 [-13.39, 0.99]	0.87
Wan et al., 2017	101	5.3142	1.7714	101	11.5141	2.6571	-6.20 [-6.82, -5.58]	0.90
Buscemi et al., 2009	10	-14	28.71	10	-8	14.09	-6.00 [-25.82, 13.82]	0.71
Meckling et al., 2004	15	-40	22.07	16	-34	56.68	-6.00 [-36.68, 24.68]	0.55
Frisch et al., 2009	85	-8.857	41.6279	80	-3.5428	44.285	-5.31 [-18.42, 7.79]	0.81
McAuley et al., 2005	31	-61.1133	37.1994	30	-55.7991	21.2568	-5.31 [-20.59, 9.96]	0.78
Cunha et al., 2020	20	-19.09	11.06	19	-14.03	17.96	-5.06 [-14.37, 4.25]	0.85
Haufe et al., 2011	52	-16.8283	38.0851	50	-12.3998	50.4849	-4.43 [-21.74, 12.88]	0.75
deLuis et al., 2015	61	-6.4	20.6	56	-2.4	13.3	-4.00 [-10.35, 2.35]	0.88
McMillan-Price et al., 2006	31	-15.9426	34.5423	27	-12.3998	31.8852	-3.54 [-20.74, 13.66]	0.75
Gram-Kampmann et al., 2022	44	-43.3993	37.1994	20	-40.7422	40.7422	-2.66 [-22.91, 17.60]	0.71
Sargrad et al., 2005	6	-176.2543	433.993	6	-174.4829	193.0826	-1.77 [-381.85, 378.31]	0.01
Abete et al., 2009	19	-4.5	7.07	19	-3	22.15	-1.50 [-11.95, 8.95]	0.84
deLuis et al., 2015	49	-7	34.22	45	-5.6	8.23	-1.40 [-11.66, 8.86]	0.84
Gardner et al., 2016	16	-35.1	67.4	15	-34.1	30.1	-1.00 [-38.19, 36.19]	0.47
Chiu et al., 2014	32	-10.6284	50.4849	31	-9.7427	49.5992	-0.89 [-25.61, 23.84]	0.64
Egert et al., 2011	18	2.6571	4.4285	19	3.5428	8.857	-0.89 [-5.44, 3.67]	0.89
Krebs et al., 2016	5	0	9.7427	5	0	8.857	0.00 [-11.54, 11.54]	0.83
Retterstøl et al., 2018	15	0	12.3998	15	0	15.9426	0.00 [-10.22, 10.22]	0.84
Hyde et al., 2021	12	1	5.43	12	.6	3.7	0.40 [-3.32, 4.12]	0.89
Lean et al., 1997	34	-22.1425	8.857	39	-23.9139	7.9713	1.77 [-2.09, 5.63]	0.89
Teng et al., 2017	18	118.6838	9.7427	18	116.9124	7.9713	1.77 [-4.04, 7.59]	0.88
Rodriguez-Villar et al., 2004	22	187.7684	86.7986	22	185.1113	139.9406	2.66 [-66.15, 71.47]	0.21
Röhling et al., 2020	65	-15	9.18	28	-18	13.27	3.00 [-1.68, 7.68]	0.89
Buga et al., 2022	7	2.6	2.32	7	-1.7	5.14	4.30 [0.12, 8.48]	0.89
Zainordin et al., 2021	14	-17.714	49.5992	16	-22.1425	90.3414	4.43 [-48.84, 57.69]	0.31
Bush et al., 2018	12	-2	6.47	17	-7	9.33	5.00 [-1.12, 11.12]	0.88
Kikuchi 2023	21	-98	72.22	21	-103	63.7	5.00 [-36.19, 46.19]	0.42
Pinsawas et al., 2024	26	-40.5	49.13	22	-45.5	50.95	5.00 [-23.37, 33.37]	0.58
Krauss et al., 2006	47	.8857	1.7714	47	-6.1999	1.7714	7.09 [6.38, 7.79]	0.90
Noakes et al., 2005	52	26.571	32.7709	48	18.5997	10.6284	7.97 [-1.74, 17.68]	0.85
Brinkworth et al., 2004b	21	17.714	41.6279	22	8.857	42.5136	8.86 [-16.31, 34.02]	0.63
Golay et al., 1996	22	-26.571	6.1999	21	-35.428	11.5141	8.86 [3.37, 14.35]	0.88
Guevara-Cruz 2024	11	10	52.5	11	1	12.95	9.00 [-22.95, 40.95]	0.53
Meckling et al., 2007	10	1	7.35	8	-8	16.36	9.00 [-2.29, 20.29]	0.83
Sun et al., 2019	15	7.9713	57.5705	15	-2.6571	21.2568	10.63 [-20.43, 41.69]	0.55
Liu et al., 2018	30	6.1999	6.1999	30	-7.0856	6.1999	13.29 [10.15, 16.42]	0.90
Brinkworth et al., 2004a	19	5.3142	59.3419	19	-11.5141	13.2855	16.83 [-10.52, 44.17]	0.60
Holmer et al., 2021	22	-17.714	11.5141	24	-35.428	11.5141	17.71 [11.05, 24.38]	0.88
Wolever et al., 2008	53	12.3998	52.2563	48	-6.1999	49.5992	18.60 [-1.32, 38.52]	0.71
Peterson et al., 1995	13	25	14.18	13	1	30.17	24.00 [5.88, 42.12]	0.74
Morgan et al., 1997	24	177.14	115.141	24	150.569	79.713	26.57 [-29.46, 82.60]	0.29
Merovci et al., 2024	10	13	19.73	8	-15	29.8	28.00 [5.09, 50.91]	0.67
Guo et al., 2022	27	-24.7996	61.999	27	-56.6848	32.7709	31.89 [4.15, 59.62]	0.59
Dellis et al., 2020	35	-15.0569	7.9713	35	-51.3706	46.9421	36.31 [20.54, 52.09]	0.77
Peterson et al., 1995	12	-3	5.12	12	-41	2.81	38.00 [34.70, 41.30]	0.89
Davis 2011	27	96	98.73	24	47	49	49.00 [5.36, 92.64]	0.39
**Overall**							-15.11 [-18.76, -11.46]	

Heterogeneity: τ^2 = 384.93, I^2 = 99.24%, H^2 = 132.23

Test of $\theta_i = \theta_j$: Q(142) = 8319.47, p = 0.00

Test of θ = 0: z = -8.11, p = 0.00

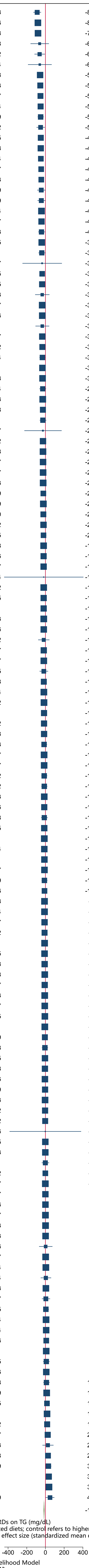

Forest plot depicting the effects of CRDs on TG (mg/dL)
Note. Treatment involves carb-restricted diets; control refers to higher-carbohydrate diets each study, the square represents the effect size (standardized mean difference) and the associated bars represent the 95% CI.

Random-effects REML model
Sorted by: _meta_es

Restricted Randomized Maximum Likelihood Model
The number of studies included: k=143

Study	Treatment N	Treatment Mean	Treatment SD	Control N	Control Mean	Control SD	Mean diff. with 95% CI	Weight (%)
Meckling et al., 2007	10	-57	12.12	8	41	159.76	-98.00 [-196.60, 0.60]	0.07
Peterson et al., 1995	13	-57	54.49	13	-7	4.43	-50.00 [-79.72, -20.28]	0.43
Skytte et al., 2019	14	-17.0148	14.3079	14	0	14.3079	-17.01 [-27.61, -6.42]	0.80
Baba et al., 1999	7	-49.1	6.94	6	-33.3	4.49	-15.80 [-22.29, -9.31]	0.87
Ahmad 2020	25	-21.6552	5.8005	25	-6.1872	7.734	-15.47 [-19.26, -11.68]	0.90
Brinkworth et al., 2004b	21	15.468	5.0271	22	30.936	19.335	-15.47 [-24.00, -6.93]	0.84
Otten et al., 2016	25	-11.601	5.8005	16	3.867	5.0271	-15.47 [-18.93, -12.01]	0.90
Golay et al., 1996	22	-46.404	19.335	21	-30.936	19.7217	-15.47 [-27.14, -3.79]	0.78
Jenkins et al., 2014	20	-25.5222	2.7069	19	-10.0542	1.9335	-15.47 [-16.95, -13.98]	0.91
Moreno et al., 2014	26	-14	9.22	27	-1.9	5.5	-12.10 [-16.17, -8.03]	0.90
Perissiou et al., 2020	33	-11.601	23.202	31	0	19.335	-11.60 [-22.10, -1.10]	0.80
Boers et al., 2014	18	-27.069	27.069	16	-15.468	19.335	-11.60 [-27.60, 4.40]	0.69
Lambadiari et al., 2024	16	174.95	36.24	16	185.74	36.04	-10.79 [-35.83, 14.25]	0.50
Mellberg et al., 2014	27	-7.734	20.1084	22	2.7069	20.1084	-10.44 [-21.76, 0.88]	0.79
Brinkworth et al., 2004a	19	3.0936	10.0542	19	13.5345	11.601	-10.44 [-17.34, -3.54]	0.86
Buscemi et al., 2009	10	-17	8	10	-7	7.21	-10.00 [-16.67, -3.33]	0.87
Röhling et al., 2020	65	-16	3.57	28	-6	5.36	-10.00 [-11.85, -8.15]	0.91
Garg et al., 1992	8	-48.3375	40.9902	8	-39.4434	37.8966	-8.89 [-47.58, 29.79]	0.31
Stoernell et al., 2008	10	-10.4409	13.1478	13	-1.5468	7.3473	-8.89 [-17.34, -0.45]	0.84
Goss et al., 2020	19	-10.6	5.87	15	-1.8	8.3	-8.80 [-13.56, -4.04]	0.89
Jönsson et al., 2009	13	166.281	46.404	13	174.015	46.404	-7.73 [-43.41, 27.94]	0.35
Morgan et al., 1997	24	232.02	34.803	24	239.754	27.069	-7.73 [-25.37, 9.91]	0.65
Wolever et al., 2008	53	-.7734	10.8276	48	6.9606	22.0419	-7.73 [-14.41, -1.06]	0.87
Martins et al., 2025	23	6	9.87	21	13.6	6.05	-7.60 [-12.49, -2.71]	0.89
OmniHeartCollaborativeResGrp 2005	164	-19.9	1.81	164	-12.4	1.68	-7.50 [-7.88, -7.12]	0.92
Li et al., 2022	24	-20.1084	10.4409	29	-12.7611	8.5074	-7.35 [-12.45, -2.25]	0.89
Kleiner et al., 2006	9	-17.6	4.69	7	-10.3	9.67	-7.30 [-14.47, -0.13]	0.86
Claessens et al., 2009	32	22.4286	4.6404	16	28.2291	5.8005	-5.80 [-8.83, -2.77]	0.91
Noakes et al., 2005	52	-18.5616	6.5739	48	-12.7611	6.1872	-5.80 [-8.31, -3.29]	0.91
Rodriguez-Villar et al., 2004	22	210.7515	42.9237	22	216.552	45.2439	-5.80 [-31.86, 20.26]	0.49
Genoni et al., 2016	22	-21.2685	5.0271	17	-15.8547	5.8005	-5.41 [-8.82, -2.01]	0.90
Chiu et al., 2014	32	-6.9606	17.4015	31	-1.5468	17.4015	-5.41 [-14.01, 3.18]	0.84
Goday et al., 2016	45	-12.6	11.81	40	-7.7	17.9	-4.90 [-11.28, 1.48]	0.87
Liu et al., 2018	30	-10.4409	3.867	30	-5.8005	3.867	-4.64 [-6.60, -2.68]	0.91
Michalczyk et al., 2020a	18	-1.17	3	20	3.28	5.53	-4.45 [-7.33, -1.57]	0.91
DalleGrave et al., 2013	43	-11.6	40.9	45	-7.2	33.2	-4.40 [-19.93, 11.13]	0.70
Frisch et al., 2009	85	1.1601	29.0025	80	5.0271	23.5887	-3.87 [-11.96, 4.23]	0.84
Lim et al., 2010	18	-15.468	131.0913	17	-11.601	127.611	-3.87 [-89.65, 81.92]	0.09
Thomsen et al., 2022	34	-23.202	23.202	32	-19.335	23.202	-3.87 [-15.07, 7.33]	0.79
Weber et al., 2022	28	-23.202	23.202	31	-19.335	23.202	-3.87 [-15.72, 7.99]	0.77
Ballard et al., 2013	21	-3.867	6.1872	21	0	.3867	-3.87 [-6.52, -1.22]	0.91
Wekesa et al., 2016	24	208.818	18.9483	24	212.685	19.335	-3.87 [-14.70, 6.96]	0.79
Brehm et al., 2005	20	-11.15	12.37	20	-7.36	9.25	-3.79 [-10.56, 2.98]	0.86
Jeppesen et al., 1997	10	203.4042	27.069	10	206.8845	32.8695	-3.48 [-29.87, 22.91]	0.48
Abete et al., 2009	19	-15.9	12.31	19	-12.6	6.43	-3.30 [-9.54, 2.94]	0.87
Teng et al., 2017	18	182.1357	4.6404	18	185.2293	.7734	-3.09 [-5.27, -0.92]	0.91
OmniHeartCollaborativeResGrp 2005	164	-15.4	1.86	164	-12.4	1.68	-3.00 [-3.38, -2.62]	0.92
deLuis et al., 2015	61	-9.8	18.68	56	-7	7.14	-2.80 [-8.01, 2.41]	0.88
Krebs et al., 2012	152	-3.867	5.4138	158	-1.5468	5.8005	-2.32 [-3.57, -1.07]	0.91
Gerhard et al., 2004	11	-19	8.68	11	-18	14.12	-1.00 [-10.79, 8.79]	0.81
Elhayany et al., 2010	61	-34.0296	6.9606	55	-33.2562	8.1207	-0.77 [-3.52, 1.97]	0.91
Thomson et al., 2010	19	-5.6	32.4	21	-5.2	31.5	-0.40 [-20.21, 19.41]	0.61
Chen et al., 2020	43	-5.23	5.88	42	-4.93	5.58	-0.30 [-2.74, 2.14]	0.91
Chen et al., 2022	36	-5.23	37.46	35	-4.93	35.1	-0.30 [-17.20, 16.60]	0.67
Ebbeling et al., 2022	53	16.1	2.34	46	16.2	2.52	-0.10 [-1.06, 0.86]	0.92
Krebs et al., 2016	5	-3.867	6.5739	5	-3.867	8.5074	0.00 [-9.42, 9.42]	0.82
Lim et al., 2010	18	-11.601	196.8303	17	-11.601	127.611	0.00 [-110.63, 110.63]	0.05
Luong et al., 2024	11	204.951	54.5247	11	204.951	57.2316	0.00 [-46.71, 46.71]	0.24
Numao et al., 2013	11	3.4803	5.0271	11	3.4803	8.5074	0.00 [-5.84, 5.84]	0.88
Parr et al., 2016	29	-15.468	23.202	32	-15.468	23.202	0.00 [-11.66, 11.66]	0.78
Sargrad et al., 2005	6	-31.3227	27.069	6	-31.3227	27.069	0.00 [-30.63, 30.63]	0.41
Tay et al., 2014b	46	-11.601	27.069	47	-11.601	34.803	0.00 [-12.69, 12.69]	0.76
Lean et al., 1997	37	-.12	.15	40	-.34	.11	0.22 [0.16, 0.28]	0.92
Cunha et al., 2020	20	-13.58	10.61	19	-13.88	4.43	0.30 [-4.85, 5.45]	0.89
Bazzano et al., 2014	59	1.9335	3.0936	60	1.1601	3.0936	0.77 [-0.34, 1.89]	0.91
Morris et al., 2020	21	1.9335	23.202	12	1.1601	15.468	0.77 [-13.97, 15.52]	0.71
de Luis et al., 2015	168	-5.6	10.62	163	-6.5	8.97	0.90 [-1.22, 3.02]	0.91
Wolever et al., 2008	53	-.7734	10.8276	55	-1.9335	11.2143	1.16 [-3.00, 5.32]	0.90
Wycherley et al., 2010	26	2.3202	22.4286	23	.3867	10.4409	1.93 [-8.08, 11.95]	0.81
Archer et al 2003	32	-27.4557	8.1207	31	-29.7759	6.9606	2.32 [-1.42, 6.06]	0.90
Foraker et al., 2014	38	-2	15.84	41	-4.5	18.86	2.50 [-5.21, 10.21]	0.85
Raygan et al., 2016	28	3	35	28	.5	28.8	2.50 [-14.29, 19.29]	0.67
Mousavi et al., 2023	35	-2.28	6.98	35	-5.08	6.98	2.80 [-0.47, 6.07]	0.90
Samaha et al., 2003	64	3	8.12	68	0	11.14	3.00 [-0.34, 6.34]	0.90
Valsdottir et al., 2020	14	-15.468	9.2808	15	-19.335	3.867	3.87 [-1.24, 8.98]	0.89
Tay et al., 2018	33	7.734	6.9606	28	3.867	6.9606	3.87 [0.36, 7.37]	0.90
Le et al., 2016	81	-9	5.09	82	-13	5.12	4.00 [2.43, 5.57]	0.91
He et al., 2022	44	5.4138	33.2562	44	1.1601	43.6971	4.25 [-11.97, 20.48]	0.68
Egert et al., 2011	18	-17.0148	4.6404	19	-21.2685	17.4015	4.25 [-4.06, 12.56]	0.84
McMillan-Price et al., 2006	28	-1.9335	20.4951	30	-6.9606	21.2685	5.03 [-5.74, 15.79]	0.80
YancyWSJr et al., 2010	57	-3.8	3.51	65	-8.86	3.29	5.06 [3.85, 6.27]	0.91
Seshadri 2004	43	3	9.31	35	-3	17.69	6.00 [-0.12, 12.12]	0.87
Keogh et al., 2007	13	-28.2291	17.4015	12	-35.1897	36.3498	6.96 [-15.09, 29.01]	0.56
McMillan-Price et al., 2006	31	9.2808	21.6552	27	1.9335	20.1084	7.35 [-3.46, 18.16]	0.80
Sharman et al., 2004	15	171.6948	36.7365	15	164.3475	29.0025	7.35 [-16.34, 31.03]	0.53
Ruth et al., 2013	18	-2.67	37.4	15	-10.2	24.2	7.53 [-14.48, 29.54]	0.56
Cipryan et al., 2022	25	-.3867	8.1207	19	-8.1207	8.1207	7.73 [2.89, 12.58]	0.89
Guldbrand et al., 2012	30	-3.867	6.5739	31	-11.601	6.5739	7.73 [4.43, 11.03]	0.90
Thorning et al., 2015	14	-10.4409	5.4138	14	-18.1749	5.0271	7.73 [3.86, 11.60]	0.90
deLuis et al., 2015	49	-10.4	12.08	45	-19.1	10.22	8.70 [4.16, 13.24]	0.89
Davis et al., 2009	55	3.867	29.3892	50	-5.0271	27.069	8.89 [-1.95, 19.74]	0.79
Alnoubi et al., 2024	14	-6.9	4.54	14	-16.2	3.6	9.30 [6.26, 12.34]	0.91
Wan et al., 2017	101	2.3202	1.5468	101	-7.734	1.5468	10.05 [9.63, 10.48]	0.92
Bradley et al., 2009	12	-23.202	10.4409	12	-34.0296	5.4138	10.83 [4.17, 17.48]	0.87
Hyde et al., 2021	12	-.5	4.72	12	-11.6	3.53	11.10 [7.77, 14.43]	0.90
Turton et al., 2023	16	185.616	46.404	16	174.015	27.069	11.60 [-14.72, 37.92]	0.48
London et al., 2024	11	-11.601	7.734	11	-23.202	6.5739	11.60 [5.60, 17.60]	0.88
Ranjan et al., 2017	10	170.148	34.803	10	158.547	38.67	11.60 [-20.64, 43.85]	0.39
Guay et al., 2012	12	3.0936	4.2537	12	-9.6675	6.5739	12.76 [8.33, 17.19]	0.89
Segal-Isaacson et al., 2004	4	143	30	4	130	7	13.00 [-17.19, 43.19]	0.42
Stern et al., 2004	44	6.1872	4.2537	43	-7.3473	6.5739	13.53 [11.21, 15.86]	0.91
Dellis et al., 2020	35	-8.1207	6.1872	35	-21.6552	6.1872	13.53 [10.64, 16.43]	0.91
Haufe et al., 2011	52	-3.0936	25.1355	50	-17.4015	30.1626	14.31 [3.55, 25.07]	0.80
Meksawan et al., 2004	10	189	31.62	10	174	31.62	15.00 [-12.72, 42.72]	0.46
Volek et al., 2004	13	185	26	13	170	30	15.00 [-6.58, 36.58]	0.57
McAuley et al., 2005	30	-7.734	9.6675	31	-23.202	5.0271	15.47 [11.62, 19.32]	0.90
Gram-Kampmann et al., 2022	44	3.867	3.4803	20	-11.601	5.0271	15.47 [13.34, 17.59]	0.91
Haufe et al., 2012	41	0	5.8005	49	-15.468	6.5739	15.47 [12.88, 18.05]	0.91
Guevara-Cruz 2024	11	-11	12.79	11	-27	4.36	16.00 [8.01, 23.99]	0.85
Merovci et al., 2024	10	-1	20.23	8	-17	9.45	16.00 [0.74, 31.26]	0.70
Cai et al., 2021	22	6.9606	6.9606	22	-10.0542	3.4803	17.01 [13.76, 20.27]	0.90
Krauss et al., 2006	47	7	3.1	49	-10.6	2.5	17.60 [16.48, 18.72]	0.91
Bush et al., 2018	12	-1	7.86	17	-20	5.09	19.00 [14.30, 23.70]	0.89
McAuley et al., 2005	31	-7.734	9.6675	30	-27.069	9.2808	19.34 [14.58, 24.09]	0.89
Tay et al., 2008	45	-.7734	11.601	43	-20.8818	5.0271	20.11 [16.34, 23.87]	0.90
Brinkworth et al., 2009	33	27.069	57.6183	36	3.867	23.202	23.20 [2.79, 43.61]	0.59
Harvey et al., 2019	14	26.2956	20.1084	12	3.0936	12.7611	23.20 [9.99, 36.42]	0.75
Pinsawas et al., 2024	26	23.4	15.47	22	-.5	15.72	23.90 [15.05, 32.75]	0.83
Liu et al., 2013	25	-.3867	10.0542	24	-24.7488	15.8547	24.36 [16.96, 31.76]	0.86
Sun et al., 2019	15	37.8966	13.1478	15	11.9877	5.8005	25.91 [18.64, 33.18]	0.86
Guo et al., 2022	27	-1.1601	6.1872	24	-27.8424	11.601	26.68 [21.66, 31.71]	0.89
Holmer et al., 2021	22	7.734	5.0271	24	-19.335	5.0271	27.07 [24.16, 29.98]	0.91
Peterson et al., 1995	12	16	25.5	12	-21	3.53	37.00 [22.43, 51.57]	0.72
Veum et al., 2017	20	5.0271	8.1207	18	-37.1232	5.4138	42.15 [37.71, 46.59]	0.89
Retterstøl et al., 2018	15	46.404	23.5887	15	0	3.867	46.40 [34.31, 58.50]	0.77
Wachsmuth et al., 2022	18	16	25.83	18	-31	8.21	47.00 [34.48, 59.52]	0.76
Buga et al., 2022	7	54.4	3.46	7	0	2.77	54.40 [51.12, 57.68]	0.90
Meckling et al., 2004	15	2	7.39	16	-62	17.34	64.00 [54.50, 73.50]	0.82
**Overall**							4.32 [1.66, 6.97]	

Heterogeneity: $\tau^2 = 200.25$, $I^2 = 99.70\%$, $H^2 = 338.31$

Test of $\theta_i = \theta_j$: Q(127) = 9351.54, p = 0.00

Test of $\theta = 0$: z = 3.19, p = 0.00

Random-effects REML model
Sorted by: _meta_es

Forest plot depicting the effects of CRDs on TC (mg/dL)
Note. Treatment involves carb-restricted diets; control refers to higher-carbohydrate diets. For each study, the square represents the effect size (standardized mean difference) and the associated bars represent the 95% CI.

-200 -100 0 100

Restricted Randomized Maximum Likelihood Model
The number of studies included: k=128

Study	Treatment N	Treatment Mean	Treatment SD	Control N	Control Mean	Control SD	Mean diff. with 95% CI	Weight (%)
Meckling et al., 2007	10	-54	87	8	44	149.09	-98.00 [-207.93, 11.93]	0.04
Baba et al., 1999	7	-21.3	4.42	6	21.5	4.24	-42.80 [-47.53, -38.07]	0.80
Michalczyk et al., 2020a	18	-32.45	2.36	20	-1.6	2.75	-30.85 [-32.49, -29.21]	0.83
Brinkworth et al., 2004b	21	7.734	5.0271	22	27.069	19.335	-19.34 [-27.87, -10.80]	0.74
Jenkins et al., 2014	20	-18.1749	1.1601	19	0	1.9335	-18.17 [-19.17, -17.18]	0.83
Brinkworth et al., 2004a	19	-7.3473	5.4138	19	10.4409	8.8941	-17.79 [-22.47, -13.11]	0.80
Goss et al., 2020	19	-10.2	3.9	15	1.9	3.97	-12.10 [-14.76, -9.44]	0.82
Wolever et al., 2008	53	-5.0271	14.3079	48	6.9606	13.9212	-11.99 [-17.50, -6.47]	0.79
Moreno et al., 2014	26	-13.6	2.2	27	-1.9	5.08	-11.70 [-13.82, -9.58]	0.83
Otten et al., 2016	25	-15.468	.3867	16	-3.867	6.1872	-11.60 [-14.02, -9.19]	0.82
Röhling et al., 2020	65	-13	3.06	28	-3	4.59	-10.00 [-11.59, -8.41]	0.83
Lambadiari et al., 2024	16	87.6	21.23	16	96.95	23.02	-9.35 [-24.69, 5.99]	0.60
Elhayany et al., 2010	61	-23.5887	3.4803	55	-14.3079	5.0271	-9.28 [-10.84, -7.72]	0.83
Li et al., 2022	24	-15.8547	7.734	29	-6.9606	5.4138	-8.89 [-12.44, -5.34]	0.81
Sacks et al., 2014	151	138	36.8	151	146.2	37.2	-8.20 [-16.55, 0.15]	0.75
Mellberg et al., 2014	27	-10.0542	16.2414	22	-2.7069	16.2414	-7.35 [-16.49, 1.80]	0.73
DalleGrave et al., 2013	43	-11.6	37.5	45	-4.6	30.7	-7.00 [-21.29, 7.29]	0.62
Buscemi et al., 2009	10	-7	10.37	10	-1	5.81	-6.00 [-13.37, 1.37]	0.76
Rodriguez-Villar et al., 2004	22	126.4509	24.7488	22	132.2514	34.4163	-5.80 [-23.51, 11.91]	0.55
Genoni et al., 2016	22	-15.468	3.867	17	-10.0542	5.0271	-5.41 [-8.20, -2.62]	0.82
Chiu et al., 2014	32	-4.2537	17.4015	31	.3867	17.4015	-4.64 [-13.24, 3.95]	0.74
Teng et al., 2017	18	111.7563	3.867	18	116.01	3.0936	-4.25 [-6.54, -1.97]	0.82
Martins et al., 2025	23	9	7	21	13	4.69	-4.00 [-7.56, -0.44]	0.81
Ahmad 2020	25	-10.0542	5.0271	25	-6.1872	4.2537	-3.87 [-6.45, -1.29]	0.82
Weber et al., 2022	28	-15.468	15.468	31	-11.601	15.468	-3.87 [-11.77, 4.04]	0.75
Parr et al., 2016	29	-7.734	19.335	32	-3.867	23.202	-3.87 [-14.65, 6.91]	0.70
Boers et al., 2014	18	-11.601	19.335	16	-7.734	19.335	-3.87 [-16.89, 9.15]	0.65
Liu et al., 2018	30	-5.8005	3.4803	30	-1.9335	3.4803	-3.87 [-5.63, -2.11]	0.83
Jönsson et al., 2009	13	104.409	38.67	13	108.276	42.537	-3.87 [-35.12, 27.38]	0.32
Wekesa et al., 2016	24	123.744	37.8966	24	127.611	18.9483	-3.87 [-20.82, 13.08]	0.56
Skytte et al., 2019	14	-6.9606	11.601	14	-3.4803	11.601	-3.48 [-12.07, 5.11]	0.74
Stoernell et al., 2008	10	-4.6404	6.5739	13	-1.5468	6.5739	-3.09 [-8.51, 2.33]	0.79
Yamada et al., 2014	12	-4.6	7.2	12	-1.7	3.22	-2.90 [-7.36, 1.56]	0.81
Noakes et al., 2005	52	-10.0542	6.1872	48	-7.3473	5.4138	-2.71 [-4.99, -0.42]	0.82
Claessens et al., 2009	32	13.9212	5.8005	16	16.6281	5.0271	-2.71 [-6.04, 0.63]	0.82
OmniHeartCollaborativeResGrp 2005	164	-14.2	1.68	164	-11.6	1.53	-2.60 [-2.95, -2.25]	0.83
Kleiner et al., 2006	9	-10.5	3.65	7	-8.6	12.37	-1.90 [-10.35, 6.55]	0.74
Frisch et al., 2009	85	.7734	25.1355	80	2.3202	22.8153	-1.55 [-8.89, 5.79]	0.76
OmniHeartCollaborativeResGrp 2005	164	-13.1	1.68	164	-11.6	1.53	-1.50 [-1.85, -1.15]	0.83
Wolever et al., 2008	53	-5.0271	14.3079	55	-3.867	23.202	-1.16 [-8.46, 6.14]	0.76
Bazzano et al., 2014	59	-3.0936	3.0936	60	-1.9335	3.0936	-1.16 [-2.27, -0.05]	0.83
Abete et al., 2009	19	-9.3	8.7	19	-8.3	6.45	-1.00 [-5.87, 3.87]	0.80
Dorans et al., 2022	75	3.2	1.96	75	4.2	2.45	-1.00 [-1.71, -0.29]	0.83
Sacks et al., 2014	150	138	36.1	150	138.6	36.6	-0.60 [-8.83, 7.63]	0.75
Garg et al., 1992	8	-25.9089	28.2291	8	-25.9089	22.8153	0.00 [-25.15, 25.15]	0.40
Guldbrand et al., 2012	30	-11.601	7.734	31	-11.601	3.867	0.00 [-3.05, 3.05]	0.82
Lim et al., 2010	18	-11.601	114.8499	17	-11.601	111.7563	0.00 [-75.14, 75.14]	0.08
Morgan et al., 1997	24	162.414	34.803	24	162.414	30.936	0.00 [-18.63, 18.63]	0.53
Perissiou et al., 2020	33	0	19.335	31	0	23.202	0.00 [-10.44, 10.44]	0.70
Tay et al., 2014b	46	-11.601	27.069	47	-11.601	27.069	0.00 [-11.00, 11.00]	0.69
Thomsen et al., 2022	34	-11.601	15.468	32	-11.601	15.468	0.00 [-7.47, 7.47]	0.76
Valsdottir et al., 2020	14	-11.601	.3867	15	-11.601	5.4138	0.00 [-2.85, 2.85]	0.82
de Luis et al., 2015	168	-4.3	10.9	163	-5	4.16	0.70 [-1.09, 2.49]	0.83
Krebs et al., 2012	152	-2.3202	1.1601	158	-3.0936	5.0271	0.77 [-0.05, 1.59]	0.83
Chen et al., 2022	36	-3.5	.4	35	-4.3	9.82	0.80 [-2.41, 4.01]	0.82
Goday et al., 2016	45	-1.8	4.8	40	-2.7	16.45	0.90 [-4.13, 5.93]	0.80
Samaha et al., 2003	64	4	4	68	3	4.43	1.00 [-0.44, 2.44]	0.83
Seshadri 2004	43	7	6	35	6	6.32	1.00 [-1.74, 3.74]	0.82
Cunha et al., 2020	20	-6.85	7.2	19	-7.91	3.87	1.06 [-2.60, 4.72]	0.81
Chen et al., 2020	43	-1.77	4.65	42	-3.03	4.02	1.26 [-0.59, 3.11]	0.83
Saslow et al., 2014	15	-2.1	7.8	18	-3.4	4.5	1.30 [-2.96, 5.56]	0.81
Sargrad et al., 2005	6	-12.3744	5.4138	6	-13.9212	39.8301	1.55 [-30.62, 33.71]	0.31
Foraker et al., 2014	38	2.1	11.09	41	.4	17.47	1.70 [-4.81, 8.21]	0.78
Ebbeling et al., 2022	53	10	1.89	46	8.2	2.04	1.80 [1.03, 2.57]	0.83
Raygan et al., 2016	28	.7	2.7	28	-1.2	10.65	1.90 [-2.17, 5.97]	0.81
Le et al., 2016	81	-5	10.02	82	-7	10.08	2.00 [-1.09, 5.09]	0.82
Jeppesen et al., 1997	10	108.6627	27.069	10	105.9558	36.7365	2.71 [-25.58, 30.99]	0.36
Bradley et al., 2009	12	-13.9212	11.601	12	-17.0148	6.5739	3.09 [-4.45, 10.64]	0.76
Egert et al., 2011	18	-12.3744	.7734	19	-15.468	3.0936	3.09 [1.62, 4.57]	0.83
Archer et al 2003	32	-21.6552	7.734	31	-25.1355	6.5739	3.48 [-0.07, 7.03]	0.81
Krebs et al., 2016	5	3.867	3.867	5	0	8.5074	3.87 [-4.32, 12.06]	0.75
Tay et al., 2015	41	-3.867	3.867	37	-7.734	4.2537	3.87 [2.06, 5.67]	0.83
Tay et al., 2018	33	7.734	6.9606	28	3.867	5.8005	3.87 [0.62, 7.12]	0.82
Turton et al., 2023	16	96.675	69.606	16	92.808	23.202	3.87 [-32.08, 39.82]	0.26
Brehm et al., 2005	20	-4.75	6.92	20	-8.68	10.49	3.93 [-1.58, 9.44]	0.79
Merovci et al., 2024	10	-6	32.25	8	-10	4.12	4.00 [-18.63, 26.63]	0.45
Gardner et al., 2007	77	.8	22.6	76	-3.8	19	4.60 [-2.02, 11.22]	0.78
McMillan-Price et al., 2006	28	-1.5468	18.5616	30	-6.5739	21.2685	5.03 [-5.28, 15.33]	0.71
Davis et al., 2009	55	-1.5468	24.3621	50	-6.9606	25.5222	5.41 [-4.13, 14.96]	0.72
Lean et al., 1997	34	-1.1601	5.4138	37	-6.5739	3.867	5.41 [3.24, 7.59]	0.83
Gardner et al., 2018	304	3.62	1.31	305	-2.12	1.31	5.74 [5.53, 5.95]	0.83
Thomson et al., 2010	19	-2	28	21	-7.8	26.8	5.80 [-11.19, 22.79]	0.56
Thorning et al., 2015	14	-.7734	3.867	14	-6.9606	4.6404	6.19 [3.02, 9.35]	0.82
Wan et al., 2017	101	-.3867	1.1601	101	-6.5739	1.1601	6.19 [5.87, 6.51]	0.83
YancyWSJr et al., 2010	57	-1.91	3.18	65	-8.29	2.94	6.38 [5.29, 7.47]	0.83
Ruth et al., 2013	18	-1.1	36.1	15	-7.6	19.2	6.50 [-13.84, 26.84]	0.49
He et al., 2022	44	11.601	33.2562	44	5.0271	35.9631	6.57 [-7.90, 21.05]	0.62
Hyde et al., 2021	12	3.6	3.93	12	-4	3.34	7.60 [4.68, 10.52]	0.82
Ballard et al., 2013	21	100.542	27.069	21	92.808	27.069	7.73 [-8.64, 24.11]	0.58
Lim et al., 2010	18	-3.867	180.5889	17	-11.601	110.9829	7.73 [-92.29, 107.76]	0.05
Meksawan et al., 2004	10	111	31.62	10	103	34.78	8.00 [-21.13, 37.13]	0.34
Keogh et al., 2007	13	-23.5887	28.2291	12	-31.7094	77.34	8.12 [-36.79, 53.03]	0.19
McMillan-Price et al., 2006	31	10.0542	21.6552	27	1.5468	20.1084	8.51 [-2.30, 19.32]	0.70
Guo et al., 2022	27	-1.9335	4.6404	24	-10.8276	5.4138	8.89 [6.13, 11.65]	0.82
Numao et al., 2013	11	10.8276	3.867	11	1.9335	10.8276	8.89 [2.10, 15.69]	0.77
Saslow et al., 2017	16	6.9	.52	18	-2	.94	8.90 [8.38, 9.42]	0.83
Haji-GhaziTehrani et al., 2022	24	-4.45	14.93	24	-13.7	20.57	9.25 [-0.92, 19.42]	0.71
Kikuchi 2023	21	8	29.62	21	-2	17.04	10.00 [-4.62, 24.62]	0.61
Segal-Isaacson et al., 2004	4	92	23	4	82	7	10.00 [-13.56, 33.56]	0.43
deLuis et al., 2015	61	7.8	1.6	56	-2.8	9.11	10.60 [8.28, 12.92]	0.82
Stern et al., 2004	44	8.1207	6.9606	43	-2.7069	4.2537	10.83 [8.40, 13.26]	0.82
deLuis et al., 2015	49	-10.8	18	45	-22	12.84	11.20 [4.83, 17.57]	0.78
Haufe et al., 2011	52	-1.5468	19.335	50	-12.7611	21.6552	11.21 [3.25, 19.17]	0.75
Gram-Kampmann et al., 2022	44	3.867	3.4803	20	-7.734	5.0271	11.60 [9.48, 13.72]	0.83
Haufe et al., 2012	41	0	3.867	49	-11.601	6.1872	11.60 [9.42, 13.78]	0.83
McAuley et al., 2005	30	0	3.867	31	-11.601	6.1872	11.60 [9.00, 14.20]	0.82
Ranjan et al., 2017	10	85.074	34.803	10	73.473	27.069	11.60 [-15.73, 38.93]	0.37
Gerhard et al., 2004	11	.8	1	11	-11	3.82	11.80 [9.47, 14.13]	0.82
Volek et al., 2004	13	119	29	13	107	27	12.00 [-9.54, 33.54]	0.47
Dellis et al., 2020	35	-.7734	1.9335	35	-13.5345	6.1872	12.76 [10.61, 14.91]	0.83
Krauss et al., 2006	47	4.3	2.7	49	-8.9	2.5	13.20 [12.16, 14.24]	0.83
Foster et al., 2010	153	7.2	2.46	154	-6.36	1.76	13.56 [13.08, 14.04]	0.83
Harvey et al., 2019	14	19.335	18.5616	12	5.4138	10.4409	13.92 [2.06, 25.78]	0.67
Sharman et al., 2004	15	117.9435	30.936	15	103.6356	25.9089	14.31 [-6.11, 34.73]	0.49
Hansen et al., 2023	110	8.8941	2.3202	55	-5.8005	3.0936	14.69 [13.85, 15.54]	0.83
Cipryan et al., 2022	25	2.3202	6.1872	19	-12.7611	5.0271	15.08 [11.67, 18.49]	0.82
McAuley et al., 2005	31	0	3.867	30	-15.468	5.8005	15.47 [13.00, 17.93]	0.82
Luong et al., 2024	11	143.079	42.537	11	127.611	27.069	15.47 [-14.33, 45.26]	0.34
Tay et al., 2008	45	-1.9335	.3867	43	-17.7882	4.2537	15.85 [14.61, 17.10]	0.83
Guay et al., 2012	12	6.9606	1.9335	12	-9.2808	3.4803	16.24 [13.99, 18.49]	0.82
Pinsawas et al., 2024	26	8.2	7.62	22	-8.6	7.63	16.80 [12.47, 21.13]	0.81
Zinn et al., 2017	14	10.0542	13.1478	12	-7.3473	5.8005	17.40 [9.35, 25.45]	0.75
Bush et al., 2018	12	3	7	17	-16	5.82	19.00 [14.32, 23.68]	0.80
Brinkworth et al., 2009	33	23.202	22.8153	36	3.867	23.202	19.34 [8.46, 30.21]	0.69
Liu et al., 2013	25	1.9335	14.6946	24	-17.4015	13.9212	19.34 [11.31, 27.36]	0.75
London et al., 2024	11	0	5.8005	11	-19.335	24.7488	19.34 [4.31, 34.36]	0.60
Guevara-Cruz 2024	11	4	11.2	11	-16	7.51	20.00 [12.03, 27.97]	0.75
Cai et al., 2021	22	11.2143	6.1872	22	-9.2808	3.4803	20.50 [17.53, 23.46]	0.82
Gardner et al., 2016	16	6.5	25.7	15	-14.4	23.8	20.90 [3.43, 38.37]	0.55
Sun et al., 2019	15	34.4163	7.734	15	11.9877	5.4138	22.43 [17.65, 27.21]	0.80
Wycherley et al., 2010	26	23.202	18.5616	23	.7734	4.6404	22.43 [14.63, 30.23]	0.76
Holmer et al., 2021	22	7.734	5.4138	24	-15.468	3.867	23.20 [20.50, 25.90]	0.82
Alnoubi et al., 2024	14	-1.1	4.9	14	-26.4	20.21	25.30 [14.41, 36.19]	0.69
Zainordin et al., 2021	14	21.2685	25.9089	16	-7.734	14.3079	29.00 [14.28, 43.73]	0.61
Wachsmuth et al., 2022	18	11	23	18	-20	11.69	31.00 [19.08, 42.92]	0.67
Retterstøl et al., 2018	15	34.803	15.468	15	0	4.2537	34.80 [26.68, 42.92]	0.75
Gardner et al., 2016	14	17.8	40.8	16	-20.8	26.1	38.60 [14.41, 62.79]	0.42
Buga et al., 2022	7	44.2	.9	7	4	2.05	40.20 [38.54, 41.86]	0.83
Veum et al., 2017	20	10.0542	6.5739	18	-30.1626	5.8005	40.22 [36.26, 44.18]	0.81
Mousavi et al., 2023	35	-29.0025	107.5026	35	-73.8597	107.5026	44.86 [-5.51, 95.22]	0.16
Meckling et al., 2004	15	1	6.93	16	-52	17.13	53.00 [43.68, 62.32]	0.73
**Overall**							4.81 [2.58, 7.05]	

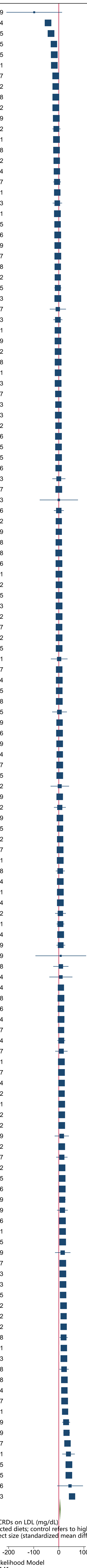

Heterogeneity: $\tau^2 = 156.23$, $I^2 = 99.66\%$, $H^2 = 290.86$

Test of $\theta_i = \theta_j$: Q(140) = 15505.10, p = 0.00

Test of $\theta = 0$: z = 4.22, p = 0.00

Random-effects REML model

Sorted by: _meta_es

Forest plot depicting the effects of CRDs on LDL (mg/dL)
Note. Treatment involves carb-restricted diets; control refers to higher-carbohydrate diets. For eac study, the square represents the effect size (standardized mean difference) and the associated bar represent the 95% CI.

Restricted Randomized Maximum Likelihood Model

The number of studies included: k=141

Study	N	Treatment Mean	Treatment SD	N	Control Mean	Control SD	Mean diff. with 95% CI	Weight (%)
Morgan et al., 1997	24	42.537	15.468	24	54.138	15.468	-11.60 [-20.35, -2.85]	0.42
Baba et al., 1999	7	-4.7	2.34	6	3.3	1.31	-8.00 [-10.12, -5.88]	0.77
Krebs et al., 2016	5	-7.734	.3867	5	0	3.867	-7.73 [-11.14, -4.33]	0.71
Lim et al., 2010	18	-3.867	32.8695	17	3.867	47.9508	-7.73 [-34.83, 19.37]	0.08
Buscemi et al., 2009	10	-8	6.56	10	-4	2.24	-4.00 [-8.30, 0.30]	0.66
Golay et al., 1996	22	-7.734	2.7069	21	-3.867	2.3202	-3.87 [-5.38, -2.36]	0.79
Otten et al., 2016	25	7.734	.3867	16	11.601	3.867	-3.87 [-5.38, -2.35]	0.79
Luong et al., 2024	11	50.271	15.468	11	54.138	15.468	-3.87 [-16.79, 9.06]	0.27
Abete et al., 2009	19	-5.6	.9	19	-3.7	3.2	-1.90 [-3.39, -0.41]	0.79
Foraker et al., 2014	38	-3.4	5.42	41	-1.8	6.19	-1.60 [-4.17, 0.97]	0.75
Keogh et al., 2007	13	-1.1601	7.3473	12	.3867	5.8005	-1.55 [-6.77, 3.67]	0.61
Rodriguez-Villar et al., 2004	22	49.4976	10.8276	22	51.0444	10.4409	-1.55 [-7.83, 4.74]	0.55
DalleGrave et al., 2013	43	4.4	7.3	45	5.9	16	-1.50 [-6.74, 3.74]	0.61
OmniHeartCollaborativeResGrp 2005	164	-2.6	.51	164	-1.4	.56	-1.20 [-1.32, -1.08]	0.81
Ahmad 2020	25	-5.4138	1.5468	25	-4.2537	1.5468	-1.16 [-2.02, -0.30]	0.80
McMillan-Price et al., 2006	31	1.9335	8.5074	27	3.0936	8.1207	-1.16 [-5.46, 3.14]	0.66
Sargrad et al., 2005	6	-3.0936	3.867	6	-1.9335	1.1601	-1.16 [-4.39, 2.07]	0.72
Kikuchi 2023	21	4	5.56	21	5	5.56	-1.00 [-4.36, 2.36]	0.71
Röhling et al., 2020	65	-1	1.28	28	0	1.53	-1.00 [-1.60, -0.40]	0.81
Cai et al., 2021	22	2.7069	1.9335	22	3.4803	2.3202	-0.77 [-2.04, 0.49]	0.80
Mellberg et al., 2014	27	6.1872	8.1207	22	6.9606	7.3473	-0.77 [-5.16, 3.61]	0.66
deLuis et al., 2015	61	-3.3	1.4	56	-2.8	.1	-0.50 [-0.87, -0.13]	0.81
Wekesa et al., 2016	24	64.1922	13.1478	24	64.5789	13.1478	-0.39 [-7.83, 7.05]	0.49
Chiu et al., 2014	32	-.3867	4.2537	31	0	4.2537	-0.39 [-2.49, 1.71]	0.77
deLuis et al., 2015	49	.1	.6	45	.4	.1	-0.30 [-0.48, -0.12]	0.81
Cunha et al., 2020	20	-2.54	2.24	19	-2.4	.07	-0.14 [-1.15, 0.87]	0.80
Ballard et al., 2013	21	0	.3867	21	0	.3867	0.00 [-0.23, 0.23]	0.81
Bush et al., 2018	12	-3	4	17	-3	2	0.00 [-2.20, 2.20]	0.77
Dellis et al., 2020	35	-2.7069	1.9335	35	-2.7069	3.0936	0.00 [-1.21, 1.21]	0.80
Haufe et al., 2012	41	-3.867	23.202	49	-3.867	11.601	0.00 [-7.40, 7.40]	0.49
Lim et al., 2010	18	3.867	49.1109	17	3.867	47.9508	0.00 [-32.18, 32.18]	0.06
Liu et al., 2018	30	-1.5468	2.3202	30	-1.5468	2.3202	0.00 [-1.17, 1.17]	0.80
Martins et al., 2025	23	-2.5	1.4	21	-2.5	.01	0.00 [-0.60, 0.60]	0.81
Parr et al., 2016	29	-3.867	7.734	32	-3.867	7.734	0.00 [-3.89, 3.89]	0.69
Perissiou et al., 2020	33	0	7.734	31	0	3.867	0.00 [-3.03, 3.03]	0.73
Seshadri 2004	43	-1	1	35	-1	1	0.00 [-0.45, 0.45]	0.81
Thomsen et al., 2022	34	-3.867	7.734	32	-3.867	3.867	0.00 [-2.98, 2.98]	0.73
Weber et al., 2022	28	-3.867	7.734	31	-3.867	3.867	0.00 [-3.07, 3.07]	0.73
Wolever et al., 2008	53	1.9335	5.8005	48	1.9335	5.4138	0.00 [-2.19, 2.19]	0.77
YancyWSJr et al., 2010	57	3.77	.98	65	3.43	.93	0.34 [0.00, 0.68]	0.81
Frisch et al., 2009	85	-.7734	8.1207	80	-1.1601	6.5739	0.39 [-1.88, 2.65]	0.76
Krebs et al., 2012	152	1.1601	.3867	158	.7734	.3867	0.39 [0.30, 0.47]	0.81
Brinkworth et al., 2004a	19	6.1872	2.3202	19	5.8005	1.5468	0.39 [-0.87, 1.64]	0.80
Haufe et al., 2011	52	-3.4803	27.8424	50	-3.867	18.9483	0.39 [-8.89, 9.67]	0.40
Stoernell et al., 2008	10	-1.1601	.3867	13	-1.5468	1.5468	0.39 [-0.60, 1.37]	0.80
Kleiner et al., 2006	9	-6.2	3.28	7	-6.6	6.02	0.40 [-4.21, 5.00]	0.65
Krauss et al., 2006	47	2.4	.8	49	1.9	.7	0.50 [0.20, 0.80]	0.81
Gardner et al., 2016	16	4.3	3.7	15	3.7	5.9	0.60 [-2.84, 4.04]	0.71
Noakes et al., 2005	52	-3.0936	3.4803	48	-3.867	1.5468	0.77 [-0.30, 1.84]	0.80
Sacks et al., 2014	151	58.2	157	151	57.3	17.2	0.90 [-24.29, 26.09]	0.09
Gerhard et al., 2004	11	-2	1	11	-3	.01	1.00 [0.41, 1.59]	0.81
Samaha et al., 2003	64	0	1	68	-1	1	1.00 [0.66, 1.34]	0.81
OmniHeartCollaborativeResGrp 2005	164	-.3	.51	164	-1.4	.56	1.10 [0.98, 1.22]	0.81
Egert et al., 2011	18	-5.4138	4.2537	19	-6.5739	1.1601	1.16 [-0.82, 3.15]	0.77
Teng et al., 2017	18	46.7907	1.5468	18	45.6306	1.1601	1.16 [0.27, 2.05]	0.80
Sun et al., 2019	15	1.9335	9.6675	15	.3867	6.5739	1.55 [-4.76, 7.08]	0.57
Skytte et al., 2019	14	0	4.2537	14	-1.5468	2.7069	1.55 [-1.09, 4.19]	0.75
McMillan-Price et al., 2006	28	2.7069	8.1207	30	1.1601	8.5074	1.55 [-2.74, 5.83]	0.66
Tay et al., 2015	41	3.867	1.1601	37	2.3202	1.1601	1.55 [1.03, 2.06]	0.81
Sharman et al., 2004	15	38.2833	7.734	15	36.7365	6.1872	1.55 [-3.47, 6.56]	0.62
Mousavi et al., 2023	35	1.89	1.48	35	.24	1.48	1.65 [0.96, 2.34]	0.81
Goday et al., 2016	45	-1	3.4	40	-2.7	1.7	1.70 [0.53, 2.87]	0.80
Dorans et al., 2022	75	5.2	.89	75	3.4	.87	1.80 [1.52, 2.08]	0.81
Saslow et al., 2017	16	4.9	.57	18	3.1	.44	1.80 [1.46, 2.14]	0.81
Brinkworth et al., 2004b	21	5.8005	1.1601	22	3.867	1.5468	1.93 [1.11, 2.75]	0.80
Holmer et al., 2021	22	3.867	1.5468	24	1.9335	1.1601	1.93 [1.15, 2.72]	0.81
Jenkins et al., 2014	20	1.5468	.3867	19	-.3867	.3867	1.93 [1.69, 2.18]	0.81
Genoni et al., 2016	22	-3.0936	1.9335	17	-5.0271	1.1601	1.93 [0.89, 2.97]	0.80
Le et al., 2016	81	-1	2.54	82	-3	2.56	2.00 [1.22, 2.78]	0.81
Meckling et al., 2007	10	-2	4	8	-4	1	2.00 [-0.86, 4.86]	0.74
Chen et al., 2020	43	4.94	1.1	42	2.94	1	2.00 [1.55, 2.45]	0.81
Chen et al., 2022	36	4.94	1.5	35	2.94	1.5	2.00 [1.30, 2.70]	0.81
Raygan et al., 2016	28	1	.5	28	-1.1	.7	2.10 [1.78, 2.42]	0.81
Thomson et al., 2010	19	2.5	8.1	21	.4	8	2.10 [-2.89, 7.09]	0.62
Sacks et al., 2014	150	59	17.5	150	56.9	15.1	2.10 [-1.60, 5.80]	0.70
Gardner et al., 2018	304	2.64	.39	305	.4	.4	2.24 [2.18, 2.30]	0.81
Cipryan et al., 2022	25	1.1601	1.5468	19	-1.1601	2.3202	2.32 [1.18, 3.46]	0.80
Guo et al., 2022	27	1.1601	2.3202	24	-1.1601	25.1355	2.32 [-7.19, 11.83]	0.39
Hansen et al., 2023	110	3.867	.7734	55	1.5468	1.1601	2.32 [2.02, 2.62]	0.81
McAuley et al., 2005	31	.3867	.3867	30	-1.9335	.3867	2.32 [2.13, 2.51]	0.81
Numao et al., 2013	11	1.5468	3.867	11	-.7734	1.1601	2.32 [-0.07, 4.71]	0.76
Lambadiari et al., 2024	16	43.96	7.05	16	41.55	7.97	2.41 [-2.80, 7.62]	0.61
He et al., 2022	44	3.4803	5.0271	44	.7734	7.734	2.71 [-0.02, 5.43]	0.74
Lean et al., 1997	34	1.9335	1.5468	37	-.7734	1.1601	2.71 [2.07, 3.34]	0.81
Foster et al., 2010	153	2.3	.64	154	-.47	.48	2.77 [2.64, 2.90]	0.81
Garg et al., 1992	8	.3867	1.5468	8	-2.7069	2.3202	3.09 [1.16, 5.03]	0.78
McAuley et al., 2005	30	.3867	.3867	31	-2.7069	1.5468	3.09 [2.52, 3.66]	0.81
Jönsson et al., 2009	13	51.8178	11.601	13	48.7242	8.8941	3.09 [-4.85, 11.04]	0.46
de Luis et al., 2015	168	1.8	1.3	163	-1.3	1.9	3.10 [2.75, 3.45]	0.81
Ebbeling et al., 2022	53	9.8	.89	46	6.7	.94	3.10 [2.74, 3.46]	0.81
Hyde et al., 2021	12	-4.8	3.56	12	-8.1	1.16	3.30 [1.18, 5.42]	0.77
London et al., 2024	11	-1.1601	.7734	11	-4.6404	.3867	3.48 [2.97, 3.99]	0.81
Pinsawas et al., 2024	26	7.6	1.23	22	4	1.25	3.60 [2.90, 4.30]	0.81
Gardner et al., 2016	14	4.4	7	16	.6	5.8	3.80 [-0.78, 8.38]	0.65
Thorning et al., 2015	14	-5.0271	1.5468	14	-8.8941	1.9335	3.87 [2.57, 5.16]	0.80
Archer et al 2003	32	-.7734	1.5468	31	-4.6404	.3867	3.87 [3.31, 4.43]	0.81
Morris et al., 2020	21	2.3202	7.734	12	-1.5468	7.734	3.87 [-1.62, 9.35]	0.60
Valsdottir et al., 2020	14	-7.734	.3867	15	-11.601	3.867	3.87 [1.83, 5.90]	0.77
Claessens et al., 2009	32	7.734	4.6404	16	3.867	1.1601	3.87 [1.55, 6.19]	0.76
Gram-Kampmann et al., 2022	44	3.867	1.5468	20	0	2.3202	3.87 [2.91, 4.83]	0.80
Wan et al., 2017	101	1.1601	.7734	101	-2.7069	.7734	3.87 [3.65, 4.08]	0.81
Wolever et al., 2008	53	1.9335	5.8005	55	-1.9335	1.1601	3.87 [2.30, 5.43]	0.79
Davis et al., 2009	55	6.1872	10.4409	50	2.3202	8.1207	3.87 [0.26, 7.47]	0.70
Li et al., 2022	24	5.0271	4.6404	29	1.1601	.3867	3.87 [2.17, 5.56]	0.78
Jeppesen et al., 1997	10	57.6183	10.8276	10	53.7513	10.8276	3.87 [-5.62, 13.36]	0.39
Turton et al., 2023	16	61.872	15.468	16	58.005	19.335	3.87 [-8.27, 16.00]	0.29
Stern et al., 2004	44	-.7734	1.1601	43	-5.0271	.7734	4.25 [3.84, 4.67]	0.81
Guldbrand et al., 2012	30	8.8941	4.2537	31	4.2537	1.1601	4.64 [3.09, 6.19]	0.79
Tay et al., 2018	33	.7734	1.5468	28	-3.867	1.1601	4.64 [3.94, 5.34]	0.81
Guay et al., 2012	12	-.7734	1.5468	12	-5.4138	3.4803	4.64 [2.49, 6.80]	0.77
Gardner et al., 2007	77	4.9	9.1	76	0	6.3	4.90 [2.42, 7.38]	0.76
Volek et al., 2004	13	53	12	13	48	10	5.00 [-3.49, 13.49]	0.43
Bradley et al., 2009	12	-4.6404	3.0936	12	-9.6675	3.4803	5.03 [2.39, 7.66]	0.75
Brehm et al., 2005	20	7.25	2.45	20	1.99	2.11	5.26 [3.84, 6.68]	0.79
Moreno et al., 2014	26	7.8	2.7	27	2.3	1.4	5.50 [4.35, 6.65]	0.80
Zainordin et al., 2021	14	1.9335	5.8005	16	-3.867	2.7069	5.80 [2.63, 8.97]	0.72
Veum et al., 2017	20	5.4138	1.5468	18	-.3867	9.6675	5.80 [1.51, 10.09]	0.66
Tay et al., 2008	45	9.6675	3.0936	43	3.0936	1.9335	6.57 [5.49, 7.66]	0.80
Goss et al., 2020	19	7.3	.1	15	.5	.4	6.80 [6.61, 6.99]	0.81
Bazzano et al., 2014	59	9.2808	1.5468	60	2.3202	1.5468	6.96 [6.40, 7.52]	0.81
Elhayany et al., 2010	61	5.0271	1.5468	55	-1.9335	1.1601	6.96 [6.60, 7.32]	0.81
Segal-Isaacson et al., 2004	4	41	12	4	34	6	7.00 [-6.15, 20.15]	0.26
Boers et al., 2014	18	0	3.867	16	-7.734	3.867	7.73 [5.13, 10.34]	0.75
Wycherley et al., 2010	26	10.8276	10.0542	23	3.0936	3.867	7.73 [3.36, 12.11]	0.66
Ranjan et al., 2017	10	81.207	19.335	10	73.473	23.202	7.73 [-10.99, 26.45]	0.16
Davis 2011	27	5.5	11.43	24	-2.3	9.8	7.80 [1.92, 13.68]	0.57
Alnoubi et al., 2024	14	-2.5	2.1	14	-10.8	10.3	8.30 [2.79, 13.81]	0.59
Brinkworth et al., 2009	33	11.601	9.2808	36	2.7069	1.9335	8.89 [5.79, 11.99]	0.73
Guevara-Cruz 2024	11	2.6	.6	11	-6.4	.85	9.00 [8.39, 9.61]	0.81
Meksawan et al., 2004	10	63	9.49	10	54	9.49	9.00 [0.68, 17.32]	0.44
Merovci et al., 2024	10	4	5.66	8	-5	2.4	9.00 [4.79, 13.21]	0.67
Michalczyk et al., 2020a	18	7.31	.74	20	-1.72	.71	9.03 [8.57, 9.49]	0.81
Yamada et al., 2014	12	5.4	4.9	12	-4.2	5.2	9.60 [5.56, 13.64]	0.68
Zinn et al., 2017	14	8.5074	5.4138	12	-1.1601	1.9335	9.67 [6.43, 12.90]	0.72
Ruth et al., 2013	18	5.1	7.3	15	-5	10.5	10.10 [4.01, 16.19]	0.56
Liu et al., 2013	25	5.8005	1.9335	24	-5.0271	2.7069	10.83 [9.51, 12.14]	0.79
Harvey et al., 2019	14	9.2808	3.4803	12	-1.9335	5.8005	11.21 [7.60, 14.83]	0.70
Retterstøl et al., 2018	15	11.601	3.867	15	0	3.867	11.60 [8.83, 14.37]	0.74
Meckling et al., 2004	15	6	4.1	16	-8	1.7	14.00 [11.82, 16.18]	0.77
Michalczyk et al., 2020b	16	16.28	3.35	45	-1.13	.04	17.41 [16.43, 18.39]	0.80
Wachsmuth et al., 2022	18	5	5	18	-19	.01	24.00 [21.69, 26.31]	0.76
Saslow et al., 2014	15	34.803	23.202	18	-34.803	11.601	69.61 [57.41, 81.80]	0.29
**Overall**							2.92 [2.10, 3.74]	

Heterogeneity: $\tau^2 = 21.55$, $I^2 = 99.79\%$, $H^2 = 485.96$

Test of $\theta_i = \theta_j$: $Q(141) = 14239.47$, $p = 0.00$

Test of $\theta = 0$: $z = 6.99$, $p = 0.00$

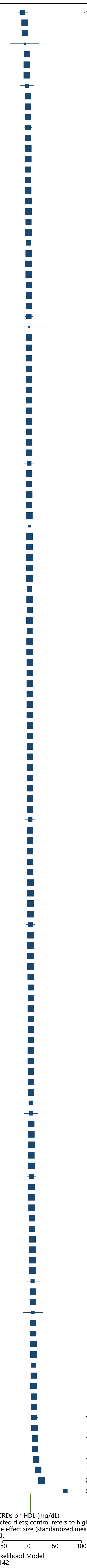

Forest plot depicting the effects of CRDs on HDL (mg/dL)
Note. Treatment involves carb-restricted diets; control refers to higher-carbohydrate diets. For each study, the square represents the effect size (standardized mean difference) and the associated bars represent the 95% CI.

Random-effects REML model
Sorted by: _meta_es

Restricted Randomized Maximum Likelihood Model
The number of studies included: k=142

Study	Treatment N	Treatment Mean	Treatment SD	Control N	Control Mean	Control SD	Mean diff. with 95% CI	Weight (%)
Skytte et al., 2019	14	-17.0148	14.3079	14	1.5468	13.1478	-18.56 [-28.74, -8.38]	7.40
OmniHeartCollaborativeResGrp 2005	164	-17.3	1.79	164	-11	1.63	-6.30 [-6.67, -5.93]	8.99
OmniHeartCollaborativeResGrp 2005	164	-15.1	1.76	164	-11	1.63	-4.10 [-4.47, -3.73]	8.99
Thomsen et al., 2022	34	-19.335	23.202	32	-15.468	23.202	-3.87 [-15.07, 7.33]	7.13
Weber et al., 2022	28	-19.335	23.202	31	-15.468	23.202	-3.87 [-15.72, 7.99]	6.96
Ebbeling et al., 2022	53	6.3	2.32	46	9.5	2.5	-3.20 [-4.15, -2.25]	8.98
Morris et al., 2020	21	.08	.5	12	.03	.2	0.05 [-0.25, 0.35]	8.99
Gardner et al., 2007	77	-5.1	22.5	76	-6.8	20.3	1.70 [-5.09, 8.49]	8.21
Wan et al., 2017	101	1.1601	1.5468	101	-5.0271	1.5468	6.19 [5.76, 6.61]	8.99
Brinkworth et al., 2009	33	9.2808	6.9606	36	-.3867	8.5074	9.67 [5.98, 13.36]	8.75
Haji-GhaziTehrani et al., 2022	24	-10.4	5.23	24	-22.8	5.44	12.40 [9.38, 15.42]	8.83
Retterstøl et al., 2018	15	34.803	15.8547	15	3.867	6.1872	30.94 [22.32, 39.55]	7.79
**Overall**							1.91 [-4.66, 8.48]	

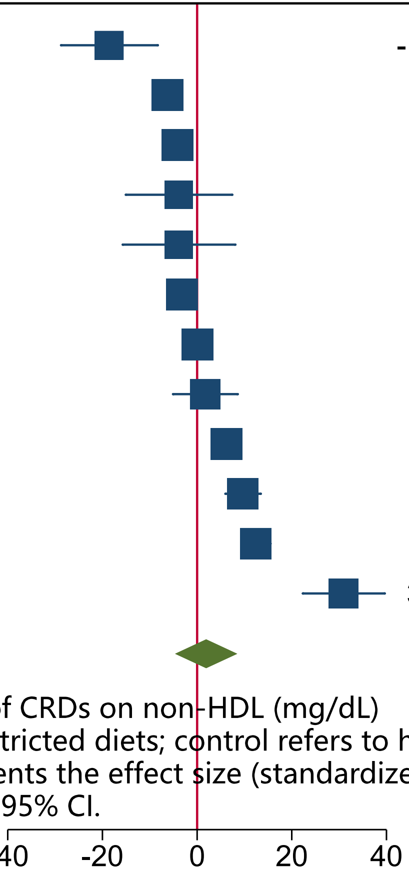

Heterogeneity: $\tau^2 = 124.74$, $I^2 = 99.91\%$, $H^2 = 1078.24$

Test of $\theta_i = \theta_j$: Q(11) = 2379.93, p = 0.00

Test of $\theta = 0$: z = 0.57, p = 0.57

Forest plot depicting the effects of CRDs on non-HDL (mg/dL)
Note. Treatment involves carb-restricted diets; control refers to higher-carbohydrate
For each study, the square represents the effect size (standardized mean difference)
the associated bars represent the 95% CI.

Random-effects REML model
Sorted by: _meta_es

Restricted Randomized Maximum Likelihood Model
The number of studies included: k=12

Study	Treatment N	Treatment Mean	Treatment SD	Control N	Control Mean	Control SD	Mean diff. with 95% CI	Weight (%)
Jenkins et al., 2014	20	-.41	.03	19	-.01	.04	-0.40 [-0.42, -0.38]	19.57
Claessens et al., 2009	32	-.08	.14	16	.17	.16	-0.25 [-0.34, -0.16]	18.08
Meksawan et al., 2004	10	1.43	.63	10	1.67	.89	-0.24 [-0.92, 0.44]	3.17
Haji-GhaziTehrani et al., 2022	24	-.37	1.13	24	-.14	.11	-0.23 [-0.68, 0.22]	5.88
Liu et al., 2013	25	-.3	.15	24	-.09	.11	-0.21 [-0.28, -0.14]	18.52
Gerhard et al., 2004	11	-.08	.1	11	.01	.31	-0.09 [-0.28, 0.10]	13.84
Egert et al., 2011	18	-.1	.07	19	-.11	.12	0.01 [-0.05, 0.07]	18.80
Morgan et al., 1997	24	4.1	1.5	24	3.5	1.5	0.60 [-0.25, 1.45]	2.14
**Overall**							-0.18 [-0.31, -0.05]	

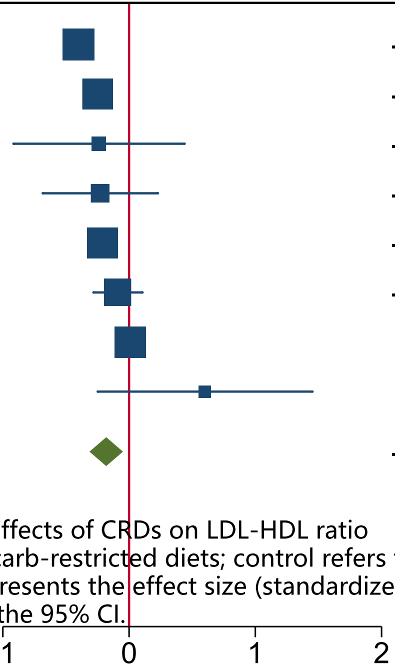

Heterogeneity: $\tau^2 = 0.02$, $I^2 = 92.69\%$, $H^2 = 13.69$

Test of $\theta_i = \theta_j$: Q(7) = 171.30, p = 0.00

Test of $\theta = 0$: z = -2.70, p = 0.01

Forest plot depicting the effects of CRDs on LDL-HDL ratio
Note. Treatment involves carb-restricted diets; control refers to higher-carbohydrate diets. each study, the square represents the effect size (standardized mean difference) and the associated bars represent the 95% CI.

Random-effects REML model
Sorted by: _meta_es

Restricted Randomized Maximum Likelihood Model
The number of studies included: k=8

Study	Treatment N	Treatment Mean	Treatment SD	Control N	Control Mean	Control SD	Mean diff. with 95% CI	Weight (%)
Boers et al., 2014	18	-.6	.28	16	1	.65	-1.60 [-1.93, -1.27]	3.23
Garg et al., 1992	8	-1.75	.68	8	-.67	1.19	-1.08 [-2.03, -0.13]	1.30
Bazzano et al., 2014	59	-.49	.1	60	-.05	.1	-0.44 [-0.48, -0.40]	4.03
Jenkins et al., 2014	20	-.68	.07	19	-.25	.11	-0.43 [-0.49, -0.37]	4.01
Jeppesen et al., 1997	10	3.65	.89	10	4.05	1.2	-0.40 [-1.33, 0.53]	1.35
Liu et al., 2013	25	-.5	3.33	24	-.11	1.19	-0.39 [-1.80, 1.02]	0.72
Haji-GhaziTehrani et al., 2022	24	-.69	.45	24	-.39	.78	-0.30 [-0.66, 0.06]	3.10
Brinkworth et al., 2004a	19	-.09	.3	19	.2	.89	-0.29 [-0.71, 0.13]	2.86
Morris et al., 2020	21	-.17	.7	12	.1	.3	-0.27 [-0.69, 0.15]	2.87
Gerhard et al., 2004	11	-.25	.13	11	.01	.32	-0.26 [-0.46, -0.06]	3.69
Archer et al 2003	32	-.57	.18	31	-.31	.25	-0.26 [-0.37, -0.15]	3.94
Genoni et al., 2016	22	-.23	.13	17	.02	.08	-0.25 [-0.32, -0.18]	4.00
Thomson et al., 2010	19	-.3	.5	21	-.1	.5	-0.20 [-0.51, 0.11]	3.30
Thorning et al., 2015	14	.2	.13	14	.36	.91	-0.16 [-0.64, 0.32]	2.63
Dorans et al., 2022	75	-.54	.07	75	-.41	.06	-0.13 [-0.15, -0.11]	4.04
Teng et al., 2017	18	4.01	.13	18	4.13	.11	-0.12 [-0.20, -0.04]	3.98
Guay et al., 2012	12	3.55	.97	12	3.63	1.17	-0.08 [-0.94, 0.78]	1.49
Sharman et al., 2004	15	4.53	.73	15	4.59	1.17	-0.06 [-0.76, 0.64]	1.89
Wan et al., 2017	101	-.01	.04	101	.04	.03	-0.05 [-0.06, -0.04]	4.04
Foster et al., 2010	153	-.31	.08	154	-.28	.06	-0.03 [-0.05, -0.01]	4.04
Volek et al., 2004	13	3.66	.92	13	3.68	.85	-0.02 [-0.70, 0.66]	1.94
Raygan et al., 2016	28	0	.24	28	0	.15	0.00 [-0.10, 0.10]	3.94
Valsdottir et al., 2020	14	.3	.24	15	.3	.16	0.00 [-0.15, 0.15]	3.85
YancyWSJr et al., 2010	57	-.44	.12	65	-.51	.12	0.07 [0.03, 0.11]	4.03
Meksawan et al., 2004	10	.7	.32	10	.6	.32	0.10 [-0.18, 0.38]	3.42
Abete et al., 2009	19	.2	.15	19	.1	.16	0.10 [0.00, 0.20]	3.95
Veum et al., 2017	20	-.61	.35	18	-.75	.26	0.14 [-0.06, 0.34]	3.70
Buga et al., 2022	7	.5	.22	7	.3	.22	0.20 [-0.03, 0.43]	3.60
Lim et al., 2010	18	-.02	1.7	17	-.3	.41	0.28 [-0.55, 1.11]	1.55
Pinsawas et al., 2024	26	0	.14	22	-.3	1.18	0.30 [-0.16, 0.76]	2.72
Krauss et al., 2006	47	-.03	.09	49	-.45	.08	0.42 [0.39, 0.45]	4.03
Lim et al., 2010	18	.2	.85	17	-.3	.41	0.50 [0.05, 0.95]	2.76
**Overall**							-0.13 [-0.26, 0.01]	

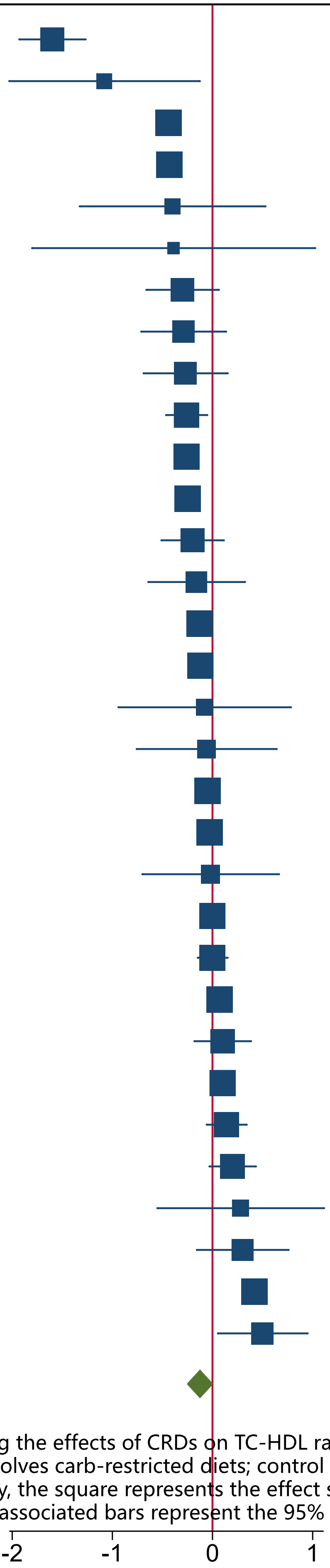

Heterogeneity: $\tau^2 = 0.11$, $I^2 = 99.47\%$, $H^2 = 188.07$

Test of $\theta_i = \theta_j$: Q(31) = 1619.82, p = 0.00

Test of $\theta = 0$: z = -1.86, p = 0.06

Forest plot depicting the effects of CRDs on TC-HDL ratio
Note. Treatment involves carb-restricted diets; control refers to higher-carbohydrate diets. For each study, the square represents the effect size (standardized mean difference) and the associated bars represent the 95% CI.

Restricted Randomized Maximum Likelihood Model
The number of studies included: k=32

Random-effects REML model
Sorted by: _meta_es

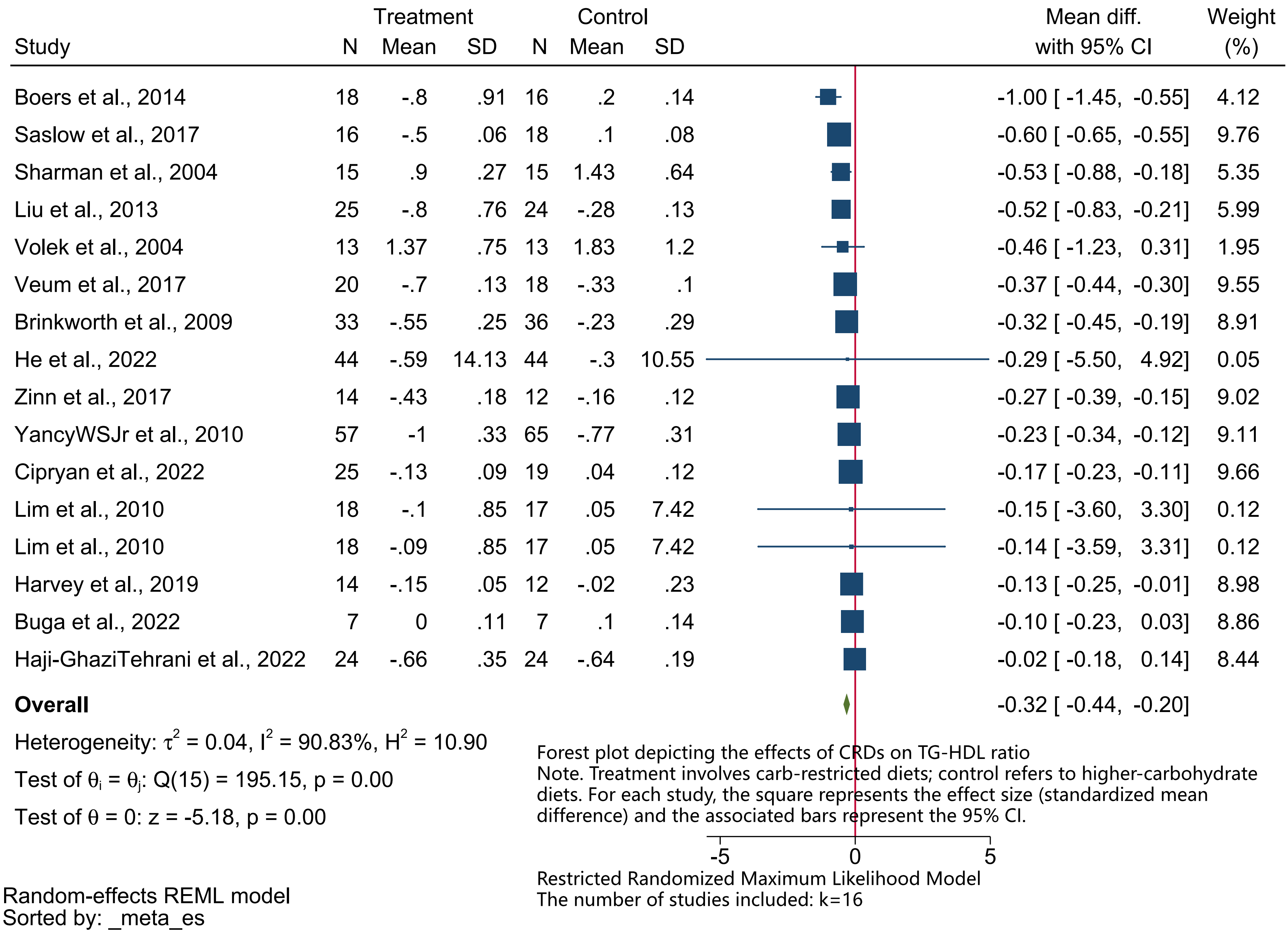

Forest plot depicting the effects of CRDs on TG-HDL ratio

Note. Treatment involves carb-restricted diets; control refers to higher-carbohydrate diets. For each study, the square represents the effect size (standardized mean difference) and the associated bars represent the 95% CI.

Restricted Randomized Maximum Likelihood Model

The number of studies included: k=16

Study	Treatment N	Treatment Mean	Treatment SD	Control N	Control Mean	Control SD	Mean diff. with 95% CI	Weight (%)
Garg et al., 1992	8	-22.4286	3.867	8	-10.4409	14.6946	-11.99 [-22.52, -1.46]	4.56
Jeppesen et al., 1997	10	27.4557	22.0419	10	38.67	35.5764	-11.21 [-37.15, 14.72]	1.00
Rodriguez-Villar et al., 2004	22	16.2414	10.8276	22	25.1355	17.4015	-8.89 [-17.46, -0.33]	5.98
Kleiner et al., 2006	9	-4.4	2.16	7	3	2.24	-7.40 [-9.57, -5.23]	13.86
Foster et al., 2010	153	-8.91	.72	154	-3.25	.91	-5.66 [-5.84, -5.48]	15.23
Gerhard et al., 2004	11	-9	11.35	11	-4	13.35	-5.00 [-15.36, 5.36]	4.67
Raygan et al., 2016	28	1.3	1.3	28	2.8	8.17	-1.50 [-4.56, 1.56]	12.72
Teng et al., 2017	18	29.0025	1.1601	18	29.3892	1.1601	-0.39 [-1.14, 0.37]	15.06
Ranjan et al., 2017	10	11.601	3.867	10	11.601	3.867	0.00 [-3.39, 3.39]	12.27
Buga et al., 2022	7	1.5	1.63	7	0	.91	1.50 [0.12, 2.88]	14.65
**Overall**							-3.34 [-6.03, -0.66]	

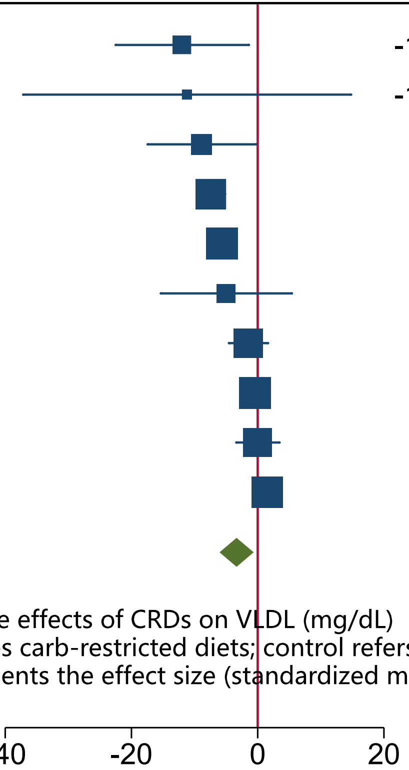

Heterogeneity: $\tau^2 = 12.33$, $I^2 = 96.39\%$, $H^2 = 27.73$

Test of $\theta_i = \theta_j$: Q(9) = 290.05, p = 0.00

Test of $\theta = 0$: z = -2.44, p = 0.01

Random-effects REML model
Sorted by: _meta_es

Forest plot depicting the effects of CRDs on VLDL (mg/dL)
Note. Treatment involves carb-restricted diets; control refers to higher-carbohydrate diets. For e
study, the square represents the effect size (standardized mean difference) and the associated b
represent the 95% CI.

Restricted Randomized Maximum Likelihood Model
The number of studies included: k=10

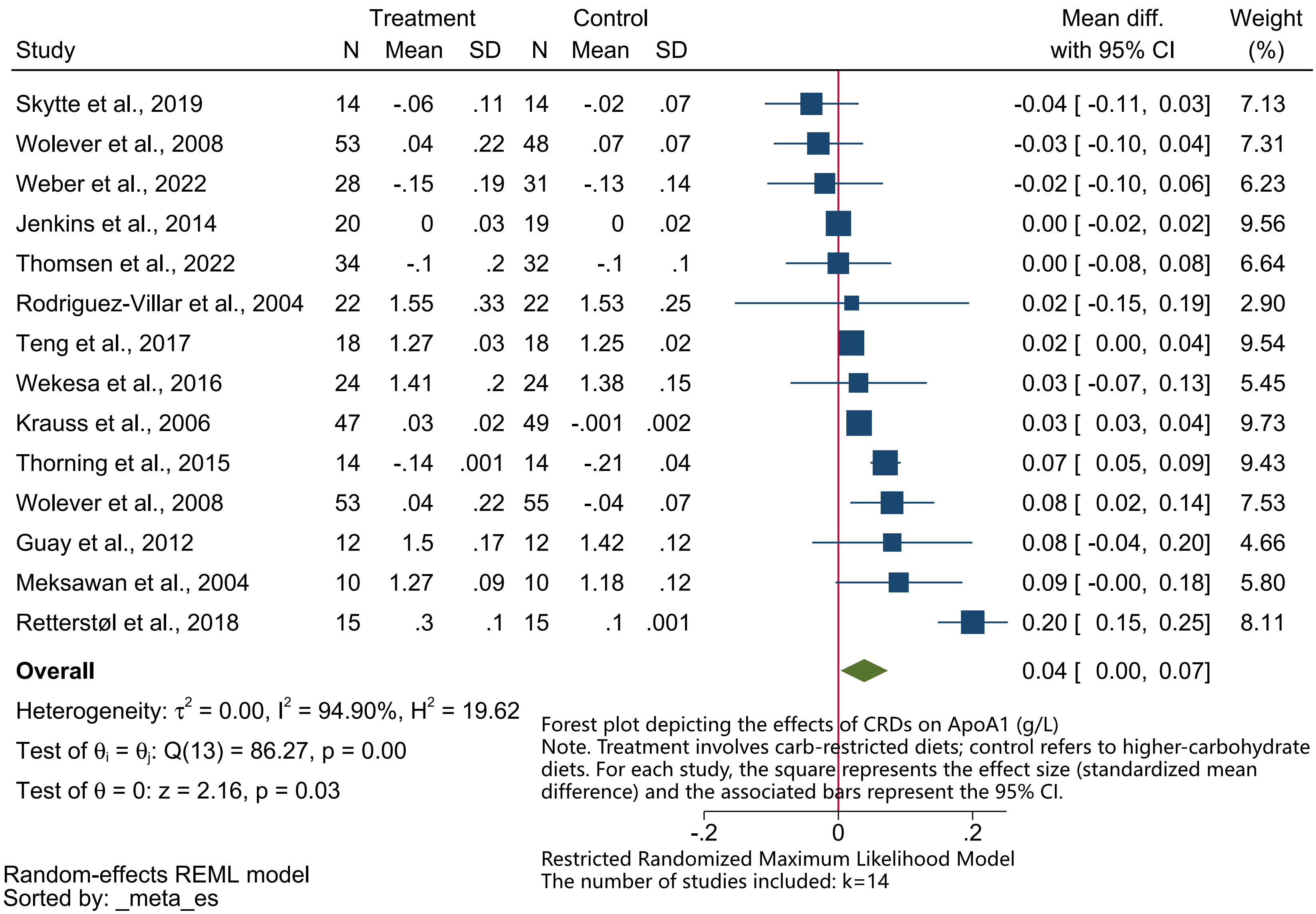

Forest plot depicting the effects of CRDs on ApoA1 (g/L)
Note. Treatment involves carb-restricted diets; control refers to higher-carbohydrate diets. For each study, the square represents the effect size (standardized mean difference) and the associated bars represent the 95% CI.

Restricted Randomized Maximum Likelihood Model
The number of studies included: k=14

Study	Treatment N	Treatment Mean	Treatment SD	Control N	Control Mean	Control SD	Mean diff. with 95% CI	Weight (%)
Skytte et al., 2019	14	-.09	.07	14	-.01	.11	-0.08 [-0.15, -0.01]	5.30
Jenkins et al., 2014	20	-.22	.01	19	-.15	.01	-0.07 [-0.08, -0.06]	6.68
Wolever et al., 2008	53	-.03	.07	48	.02	.14	-0.05 [-0.09, -0.01]	6.07
Weber et al., 2022	28	-.13	.13	31	-.1	.12	-0.03 [-0.09, 0.03]	5.45
Wolever et al., 2008	53	-.03	.07	55	0	.15	-0.03 [-0.07, 0.01]	6.02
Wekesa et al., 2016	24	1.1	.2	24	1.12	.15	-0.02 [-0.12, 0.08]	4.28
Chiu et al., 2014	32	-.03	.06	31	-.02	.06	-0.01 [-0.04, 0.02]	6.38
Lim et al., 2010	18	-.1	1.27	17	-.1	.82	0.00 [-0.71, 0.71]	0.23
Thomsen et al., 2022	34	-.1	.1	32	-.1	.1	0.00 [-0.05, 0.05]	5.92
Thorning et al., 2015	14	-.02	.001	14	-.03	.001	0.01 [0.01, 0.01]	6.69
Wan et al., 2017	101	0	.01	101	-.03	.01	0.03 [0.03, 0.03]	6.69
Tay et al., 2008	45	-.02	.18	43	-.05	.19	0.03 [-0.05, 0.11]	5.01
Rodriguez-Villar et al., 2004	22	1.31	.34	22	1.27	.34	0.04 [-0.16, 0.24]	2.05
Guay et al., 2012	12	.88	.2	12	.83	.22	0.05 [-0.12, 0.22]	2.58
Krauss et al., 2006	47	.02	.02	49	-.06	.02	0.08 [0.07, 0.09]	6.67
Brinkworth et al., 2009	33	.03	.001	36	-.06	.06	0.09 [0.07, 0.11]	6.54
Hansen et al., 2023	110	.04	.02	55	-.05	.02	0.09 [0.08, 0.10]	6.68
Meksawan et al., 2004	10	.7	.1	10	.6	.1	0.10 [0.01, 0.19]	4.68
Lim et al., 2010	18	.1	1.27	17	-.1	.82	0.20 [-0.51, 0.91]	0.23
Retterstøl et al., 2018	15	.2	.1	15	0	.001	0.20 [0.15, 0.25]	5.85
**Overall**							0.02 [-0.01, 0.06]	

Heterogeneity: $\tau^2 = 0.00$, $I^2 = 99.72\%$, $H^2 = 352.96$

Test of $\theta_i = \theta_j$: $Q(19) = 1817.94$, $p = 0.00$

Test of $\theta = 0$: $z = 1.33$, $p = 0.18$

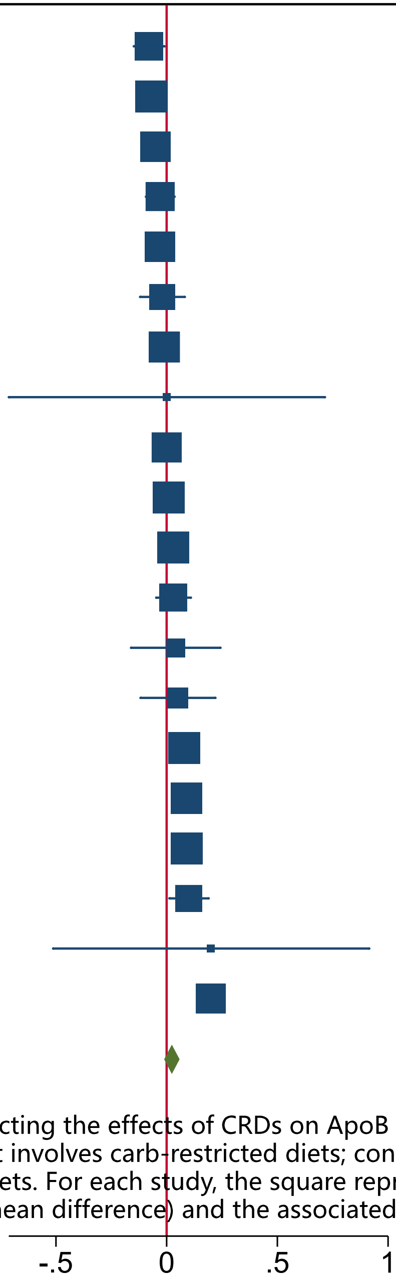

Forest plot depicting the effects of CRDs on ApoB (g/L)
Note. Treatment involves carb-restricted diets; control refers to higher-carbohydrate diets. For each study, the square represents the effect size (standardized mean difference) and the associated bars represent the 95% CI.

Restricted Randomized Maximum Likelihood Model
The number of studies included: k=20

Random-effects REML model
Sorted by: _meta_es

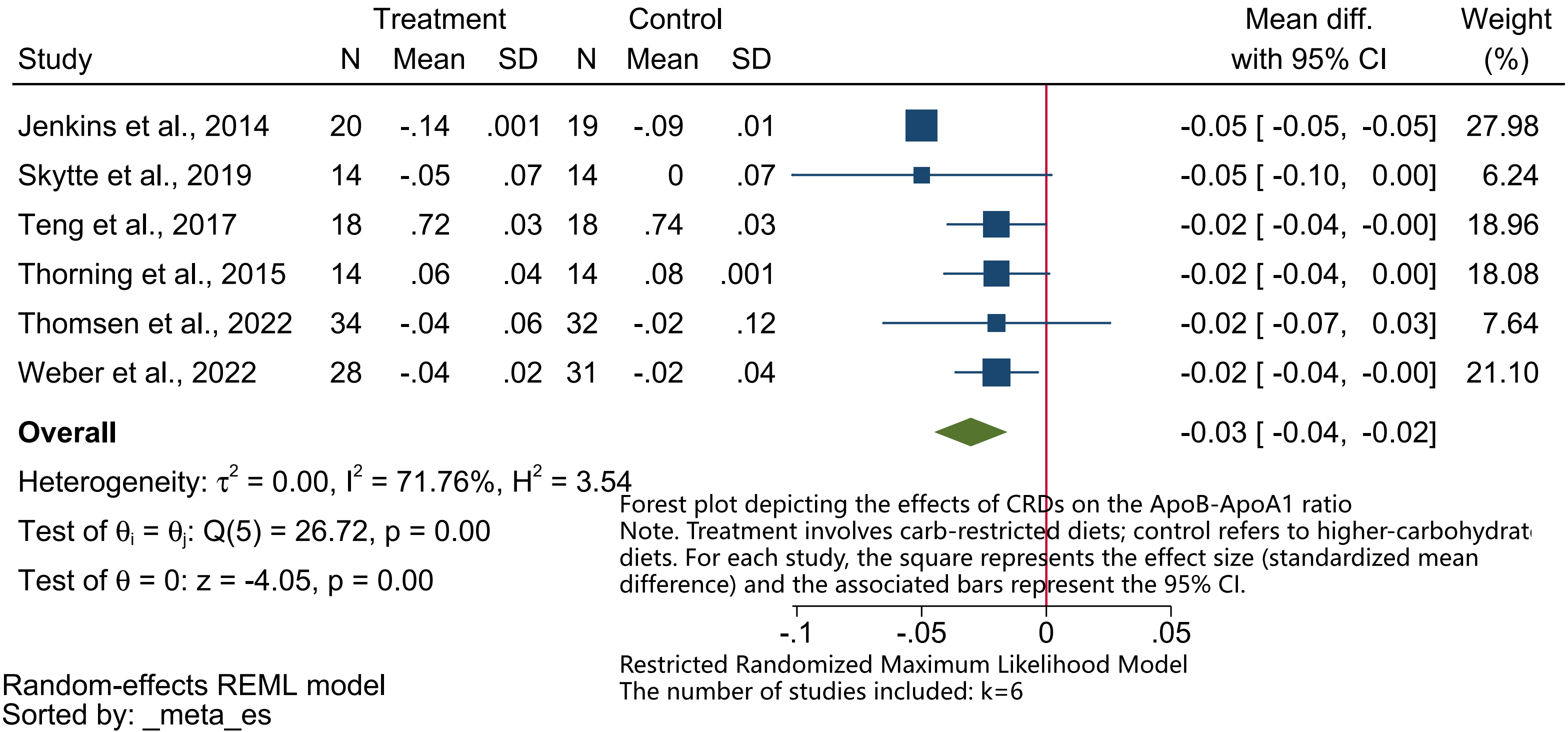
Treatment
Control
Study
N
Mean
SD
N
Mean
SD
Mean diff. with 95% CI
Weight (%)
Jenkins et al., 2014 20 -.14 .001 19 -.09 .01 -0.05 [-0.05, -0.05] 27.98
Skytte et al., 2019 14 -.05 .07 14 0 .07 -0.05 [-0.10, 0.00] 6.24
Teng et al., 2017 18 .72 .03 18 .74 .03 -0.02 [-0.04, -0.00] 18.96
Thorning et al., 2015 14 .06 .04 14 .08 .001 -0.02 [-0.04, 0.00] 18.08
Thomsen et al., 2022 34 -.04 .06 32 -.02 .12 -0.02 [-0.07, 0.03] 7.64
Weber et al., 2022 28 -.04 .02 31 -.02 .04 -0.02 [-0.04, -0.00] 21.10
Overall -0.03 [-0.04, -0.02]
Heterogeneity: τ^2 = 0.00, I^2 = 71.76%, H^2 = 3.54
Test of $\theta_i = \theta_j$: Q(5) = 26.72, p = 0.00
Test of θ = 0: z = -4.05, p = 0.00
Forest plot depicting the effects of CRDs on the ApoB-ApoA1 ratio
Note. Treatment involves carb-restricted diets; control refers to higher-carbohydrat
diets. For each study, the square represents the effect size (standardized mean
difference) and the associated bars represent the 95% CI.
-.1
-.05
0
.05
Restricted Randomized Maximum Likelihood Model
The number of studies included: k=6
Random-effects REML model
Sorted by: _meta_es

Study	Treatment N	Treatment Mean	Treatment SD	Control N	Control Mean	Control SD	Mean diff. with 95% CI	Weight (%)
Buscemi et al., 2009	10	-1.2	5.38	10	.3	1.26	-1.50 [-4.92, 1.92]	2.12
Ruth et al., 2013	18	-.53	4.22	15	.47	4.29	-1.00 [-3.91, 1.91]	2.84
Gram-Kampmann et al., 2023	44	-.19	.34	20	.37	.52	-0.56 [-0.77, -0.35]	23.11
Tay et al., 2018	33	-.5	.51	28	-.4	.54	-0.10 [-0.36, 0.16]	22.64
Wycherley et al., 2016	58	-.28	.23	57	-.64	.23	0.36 [0.28, 0.44]	23.88
Keogh et al., 2007	13	0	.36	12	-.9	1.04	0.90 [0.30, 1.50]	18.25
Ballard et al., 2013	21	5.5	2.9	21	4.5	2.5	1.00 [-0.64, 2.64]	7.15
**Overall**							0.11 [-0.41, 0.63]	

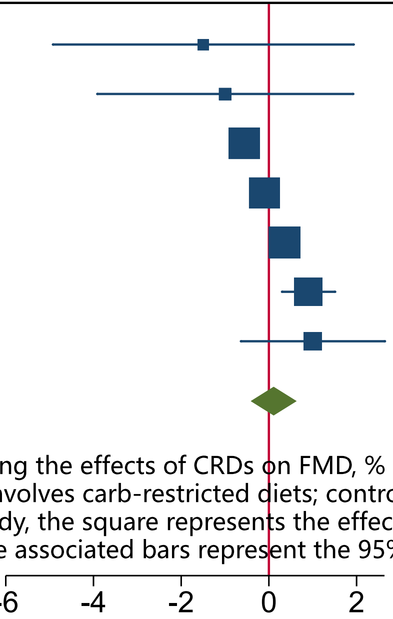

Heterogeneity: $\tau^2 = 0.30$, $I^2 = 92.68\%$, $H^2 = 13.66$

Test of $\theta_i = \theta_j$: $Q(6) = 75.06$, $p = 0.00$

Test of $\theta = 0$: $z = 0.41$, $p = 0.68$

Forest plot depicting the effects of CRDs on FMD, %

Note. Treatment involves carb-restricted diets; control refers to higher-carbohydrate diets. For each study, the square represents the effect size (standardized mean difference) and the associated bars represent the 95% CI.

Restricted Randomized Maximum Likelihood Model

The number of studies included: k=143

Random-effects REML model

Sorted by: _meta_es

Study	Treatment N	Treatment Mean	Treatment SD	Control N	Control Mean	Control SD	Mean diff. with 95% CI	Weight (%)
Davis 2011	27	82	51.96	27	93	53.96	-11.00 [-39.26, 17.26]	0.42
Ballard et al., 2013	21	22.5	9.3	21	30.2	11.2	-7.70 [-13.93, -1.47]	5.96
Kitabchi et al., 2013	12	-8.6	.67	12	-3.7	.57	-4.90 [-5.40, -4.40]	17.62
HU et al., 2015	75	.4	1.89	73	4.2	1.89	-3.80 [-4.41, -3.19]	17.51
Wekesa et al., 2016	24	11.9	6.86	24	13.1	6.86	-1.20 [-5.08, 2.68]	10.06
Wycherley et al., 2010	26	-3.8	5.1	23	-3.3	.93	-0.50 [-2.62, 1.62]	14.50
Rajaie et al., 2013	30	-6.36	.84	30	-6.36	.86	0.00 [-0.43, 0.43]	17.68
Teng et al., 2017	18	36.53	2.1	18	36.53	2.15	0.00 [-1.39, 1.39]	16.24
**Overall**							-2.23 [-4.09, -0.36]	

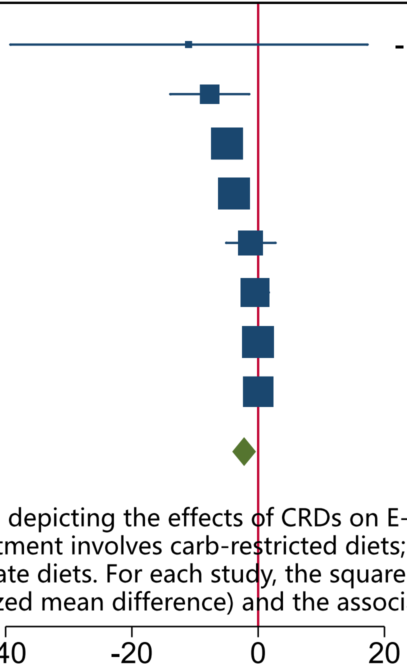

Heterogeneity: $\tau^2 = 5.07$, $I^2 = 96.11\%$, $H^2 = 25.69$

Test of $\theta_i = \theta_j$: Q(7) = 254.72, p = 0.00

Test of $\theta = 0$: z = -2.34, p = 0.02

Forest plot depicting the effects of CRDs on E-selectin (ng/mL)
Note. Treatment involves carb-restricted diets; control refers to higher-carbohydrate diets. For each study, the square represents the effect size (standardized mean difference) and the associated bars represent the 95% CI.

Restricted Randomized Maximum Likelihood Model
The number of studies included: k=8

Random-effects REML model
Sorted by: _meta_es

Study	Treatment N	Treatment Mean	Treatment SD	Control N	Control Mean	Control SD	Mean diff. with 95% CI	Weight (%)
Davis 2011	27	199	103.1	27	234	103.19	-35.00 [-90.02, 20.02]	6.31
Hu et al., 2015	75	-16.8	7.76	73	18.1	5.48	-34.90 [-37.07, -32.73]	22.19
Ballard et al., 2013	21	125.4	57.8	21	148.1	94.6	-22.70 [-70.11, 24.71]	7.74
Wekesa et al., 2016	24	294	181.26	24	307	127.37	-13.00 [-101.63, 75.63]	2.94
Wycherley et al., 2010	26	-17.5	7.91	23	-5.1	4.4	-12.40 [-16.05, -8.75]	22.03
Rajaie et al., 2013	30	23	18.33	30	25.6	14.68	-2.60 [-11.00, 5.80]	21.04
Brinkworth et al., 2004b	21	-150.4	27.2	22	-166	31	15.60 [-1.86, 33.06]	17.75
**Overall**							-12.60 [-28.93, 3.72]	

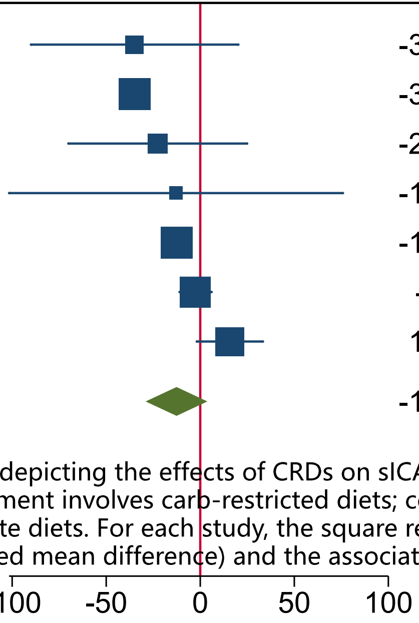

Heterogeneity: $\tau^2 = 311.40$, $I^2 = 96.52\%$, $H^2 = 28.74$

Test of $\theta_i = \theta_j$: $Q(6) = 167.70$, $p = 0.00$

Test of $\theta = 0$: $z = -1.51$, $p = 0.13$

Forest plot depicting the effects of CRDs on sICAM-1 (ng/mL)
Note. Treatment involves carb-restricted diets; control refers to higher-carbohydrate diets. For each study, the square represents the effect size (standardized mean difference) and the associated bars represent the 95%

Restricted Randomized Maximum Likelihood Model
The number of studies included: k=7

Random-effects REML model
Sorted by: _meta_es

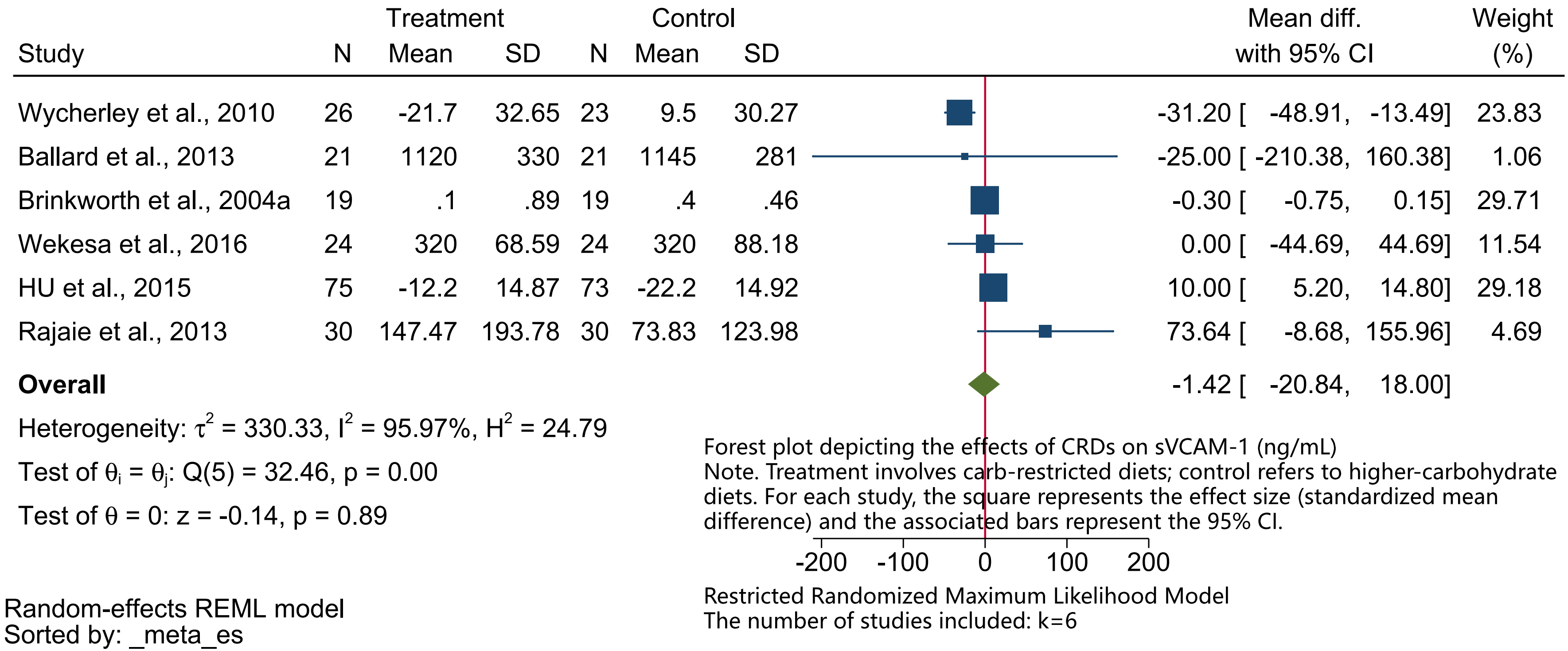

Forest plot depicting the effects of CRDs on sVCAM-1 (ng/mL)
Note. Treatment involves carb-restricted diets; control refers to higher-carbohydrate diets. For each study, the square represents the effect size (standardized mean difference) and the associated bars represent the 95% CI.

Restricted Randomized Maximum Likelihood Model
The number of studies included: k=6

Study	Treatment N	Treatment Mean	Treatment SD	Control N	Control Mean	Control SD	Mean diff. with 95% CI	Weight (%)
Kitabchi et al., 2013	12	-21	7.1	12	-8	1	-13.00 [-17.06, -8.94]	0.49
Saslow et al., 2014	15	-15	22.8	18	-6	5.6	-9.00 [-19.88, 1.88]	0.08
Le et al., 2016	81	-16.8	19.2	82	-10.9	9.7	-5.90 [-10.56, -1.24]	0.39
Michalczyk et al., 2020a	18	-3.52	1.39	20	.16	.63	-3.68 [-4.35, -3.01]	2.12
Stoernell et al., 2008	10	-2.2	2.56	13	.6	.25	-2.80 [-4.19, -1.41]	1.62
Jenkins et al., 2014	20	-4	1.1	19	-2	3.1	-2.00 [-3.45, -0.55]	1.58
Saslow et al., 2017	16	-12	3.3	18	-10	2.7	-2.00 [-4.02, 0.02]	1.21
Bazzano et al., 2014	59	-.7035	.51135	60	.903	.51135	-1.61 [-1.79, -1.42]	2.32
Guevara-Cruz 2024	11	-.27	.54	11	1.25	1.16	-1.52 [-2.28, -0.76]	2.07
Ruth et al., 2013	18	-1.68	2.78	15	-.19	1.1	-1.49 [-2.99, 0.01]	1.55
deLuis et al., 2007	47	-1.3	2.12	43	-.1	.95	-1.20 [-1.89, -0.51]	2.11
Noakes et al., 2005	52	-1.7	.98	48	-.8	.82	-0.90 [-1.26, -0.54]	2.27
Raygan et al., 2016	28	-.5643	.5198	28	.2861	.27175	-0.85 [-1.07, -0.63]	2.32
Tzenios et al., 2022	11	1.24	1.16	11	2.05	2.55	-0.81 [-2.47, 0.85]	1.44
Retterstøl et al., 2018	15	-.7	1.11	15	.1	.16	-0.80 [-1.37, -0.23]	2.18
Bush et al., 2018	12	-.6	.73	17	.16	1.24	-0.76 [-1.54, 0.02]	2.05
de Luis et al., 2015	168	-.5	.44	163	.2	1.37	-0.70 [-0.92, -0.48]	2.31
Perissiou et al., 2020	33	-.9	1.9	31	-.2	1.3	-0.70 [-1.50, 0.10]	2.04
Genoni et al., 2016	22	-.59	1.24	17	.07	.63	-0.66 [-1.31, -0.01]	2.14
McAuley et al., 2005	30	-.64	.25	31	0	.55	-0.64 [-0.86, -0.42]	2.32
Jönsson et al., 2009	13	2	1.6	13	2.6	2.3	-0.60 [-2.12, 0.92]	1.53
Brinkworth et al., 2004a	19	-1.2	1.03	19	-.6	.43	-0.60 [-1.10, -0.10]	2.21
Zainordin et al., 2021	14	-.31	2.51	16	.26	1.37	-0.57 [-1.99, 0.85]	1.60
Boers et al., 2014	18	.2	.55	16	.7	1.5	-0.50 [-1.24, 0.24]	2.08
Mellberg et al., 2014	27	-.45	1.2	22	-.05	1.36	-0.40 [-1.12, 0.32]	2.09
McAuley et al., 2005	31	-.64	.25	30	-.27	.34	-0.37 [-0.52, -0.22]	2.33
Wekesa et al., 2016	24	1.14	1.76	24	1.49	1.52	-0.35 [-1.28, 0.58]	1.95
Brinkworth et al., 2004b	21	-.3	.65	22	0	.4	-0.30 [-0.62, 0.02]	2.29
Teng et al., 2017	18	3.7	.56	18	3.84	.4	-0.14 [-0.46, 0.18]	2.29
Haufe et al., 2012	41	-.4	.41	49	-.3	.19	-0.10 [-0.23, 0.03]	2.33
Rajaie et al., 2013	30	-.32	.99	30	-.23	1.02	-0.09 [-0.60, 0.42]	2.21
YancyWSJr et al., 2010	57	-.18	.06	65	-.15	.06	-0.03 [-0.05, -0.01]	2.34
McMillan-Price et al., 2006	31	-.8	2.23	27	-.8	2.08	0.00 [-1.12, 1.12]	1.82
Tay et al., 2014b	46	-.6	1.7	47	-.6	1.7	0.00 [-0.69, 0.69]	2.11
Thomson et al., 2010	19	-.4	1.7	21	-.4	1.6	0.00 [-1.02, 1.02]	1.89
Tay et al., 2008	45	-1.2	.49	43	-1.27	.81	0.07 [-0.21, 0.35]	2.30
Ballard et al., 2013	21	1.2	1	21	1.1	1	0.10 [-0.50, 0.70]	2.16
Brinkworth et al., 2009	33	-1.3	1.15	36	-1.4	1.8	0.10 [-0.62, 0.82]	2.09
Kikuchi 2023	21	-.1	1.3	21	-.2	.6	0.10 [-0.51, 0.71]	2.16
Haufe et al., 2011	52	-.34	1.37	50	-.51	1.13	0.17 [-0.32, 0.66]	2.22
Tay et al., 2018	33	-.6	.27	28	-.8	.31	0.20 [0.05, 0.35]	2.33
McCullough et al., 2022	8	.14	.39	8	-.08	.14	0.22 [-0.07, 0.51]	2.30
Tay et al., 2015	41	-.9	.33	37	-1.2	.33	0.30 [0.15, 0.45]	2.33
Lim et al., 2010	18	-1.8	13.15	17	-2.1	10.31	0.30 [-7.56, 8.16]	0.15
Lim et al., 2010	18	-1.7	28.43	17	-2.1	10.31	0.40 [-13.94, 14.74]	0.05
Goss et al., 2020	19	2.1	2.64	15	1.6	1.72	0.50 [-1.05, 2.05]	1.51
deLuis et al., 2015	61	.3	1.31	56	-.4	1.11	0.70 [0.26, 1.14]	2.24
Wolever et al., 2008	53	.41	.09	48	-.59	.21	1.00 [0.94, 1.06]	2.34
McMillan-Price et al., 2006	28	-.01	2.12	30	-1.1	2.19	1.09 [-0.02, 2.20]	1.83
Wolever et al., 2008	53	.41	.09	55	-.69	.31	1.10 [1.01, 1.19]	2.34
Davis 2011	27	.5	1.41	24	-1	.53	1.50 [0.90, 2.10]	2.16
deLuis et al., 2015	49	.9	.44	45	-.7	.87	1.60 [1.32, 1.88]	2.30
Wachsmuth et al., 2022	18	-1	4.1	18	-4	10.01	3.00 [-2.00, 8.00]	0.35
Harvey et al., 2019	14	.14	.33	12	-3.9	4.08	4.04 [1.90, 6.18]	1.15
**Overall**							-0.37 [-0.69, -0.05]	

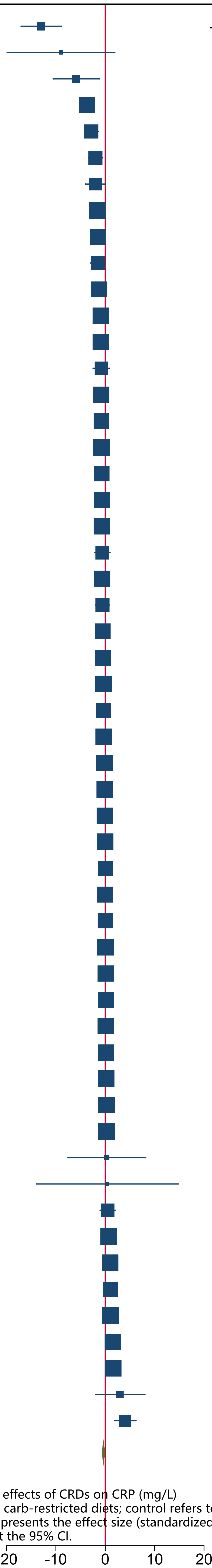

Heterogeneity: $\tau^2 = 1.14$, $I^2 = 99.12\%$, $H^2 = 113.75$

Test of $\theta_i = \theta_j$: $Q(53) = 2489.15$, $p = 0.00$

Test of $\theta = 0$: $z = -2.26$, $p = 0.02$

Forest plot depicting the effects of CRDs on CRP (mg/L)
Note. Treatment involves carb-restricted diets; control refers to higher-carbohydrate diets. each study, the square represents the effect size (standardized mean difference) and the associated bars represent the 95% CI.

Restricted Randomized Maximum Likelihood Model
The number of studies included: k=54

Random-effects REML model
Sorted by: _meta_es

Study	Treatment N	Treatment Mean	Treatment SD	Control N	Control Mean	Control SD	Mean diff. with 95% CI	Weight (%)
Kitabchi et al., 2013	12	-1.8	3.82	12	-.9	.35	-0.90 [-3.07, 1.27]	0.50
Pinsawas et al., 2024	26	-.2	.22	22	.5	.52	-0.70 [-0.92, -0.48]	13.25
McCullough et al., 2022	8	-.77	.51	8	-.35	.54	-0.42 [-0.93, 0.09]	6.09
Hu et al., 2015	75	.74	.2	73	1.08	.2	-0.34 [-0.40, -0.28]	17.41
deLuis et al., 2007	47	-.4	2.05	43	-.1	.45	-0.30 [-0.93, 0.33]	4.62
Ballard et al., 2013	21	7.3	4.6	21	7.5	4.8	-0.20 [-3.04, 2.64]	0.30
Boers et al., 2014	18	.1	.31	16	.3	.44	-0.20 [-0.45, 0.05]	12.19
Numao et al., 2013	11	-.06	.07	11	.05	.11	-0.11 [-0.19, -0.03]	17.19
Buscemi et al., 2009	10	.1	.09	10	.2	.32	-0.10 [-0.31, 0.11]	13.68
Goss et al., 2020	19	.1	.17	15	.2	.33	-0.10 [-0.27, 0.07]	14.77
**Overall**							-0.27 [-0.42, -0.11]	

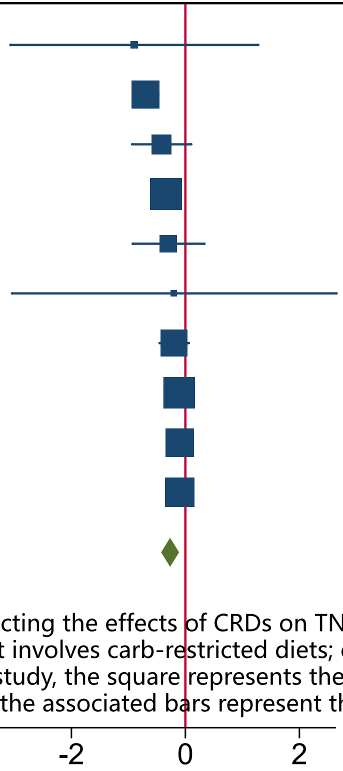

Heterogeneity: $\tau^2 = 0.04$, $I^2 = 83.56\%$, $H^2 = 6.08$

Test of $\theta_i = \theta_j$: $Q(9) = 42.20$, $p = 0.00$

Test of $\theta = 0$: $z = -3.36$, $p = 0.00$

Forest plot depicting the effects of CRDs on TNF-a (pg/mL)
Note. Treatment involves carb-restricted diets; control refers to higher-carbohydrat
diets. For each study, the square represents the effect size (standardized mean difference) and the associated bars represent the 95% CI.

Restricted Randomized Maximum Likelihood Model
The number of studies included: k=10

Random-effects REML model
Sorted by: _meta_es

Study	Treatment N	Treatment Mean	Treatment SD	Control N	Control Mean	Control SD	Mean diff. with 95% CI	Weight (%)
Lambadiari et al., 2024	16	6.33	12.47	16	8.17	12.85	-1.84 [-10.61, 6.93]	0.27
HU et al., 2015	75	.58	.46	73	1.8	.47	-1.22 [-1.37, -1.07]	18.00
Kitabchi et al., 2013	12	-1.3	4.68	12	-.4	.1	-0.90 [-3.55, 1.75]	2.56
Goss et al., 2020	19	-.1	1.16	15	.3	.24	-0.40 [-1.00, 0.20]	13.95
Numao et al., 2013	11	-.24	2.03	11	.1	.17	-0.34 [-1.54, 0.86]	8.06
Teng et al., 2017	18	1.52	.1	18	1.56	.15	-0.04 [-0.12, 0.04]	18.25
Ballard et al., 2013	21	2	1.9	21	1.9	1.5	0.10 [-0.94, 1.14]	9.44
deLuis et al., 2007	47	.5	1.42	43	.3	.35	0.20 [-0.24, 0.64]	15.72
McCullough et al., 2022	8	.24	1	8	-.07	.46	0.31 [-0.45, 1.07]	12.14
Buscemi et al., 2009	10	-.9	4.53	10	-3.8	3.22	2.90 [-0.54, 6.34]	1.60
**Overall**							-0.21 [-0.67, 0.24]	

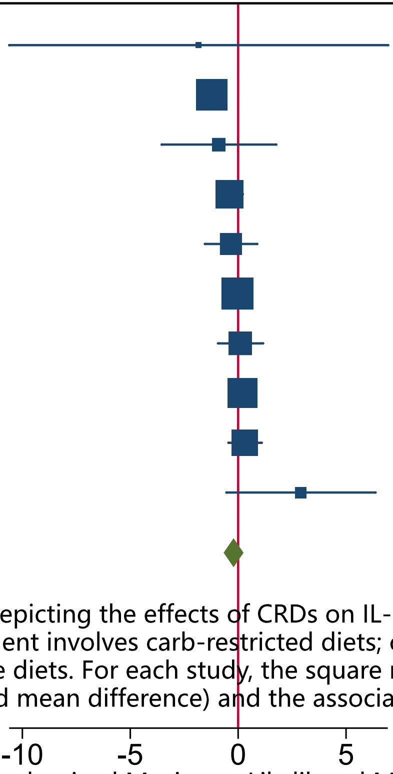

Heterogeneity: $\tau^2 = 0.30$, $I^2 = 91.60\%$, $H^2 = 11.90$

Test of $\theta_i = \theta_j$: Q(9) = 194.09, p = 0.00

Test of $\theta = 0$: z = -0.92, p = 0.36

Forest plot depicting the effects of CRDs on IL-6 (pg/mL)
Note. Treatment involves carb-restricted diets; control refers to higher-carbohydrate diets. For each study, the square represents the effect size (standardized mean difference) and the associated bars represent the 95% CI.

Restricted Randomized Maximum Likelihood Model
The number of studies included: k=10

Random-effects REML model
Sorted by: _meta_es

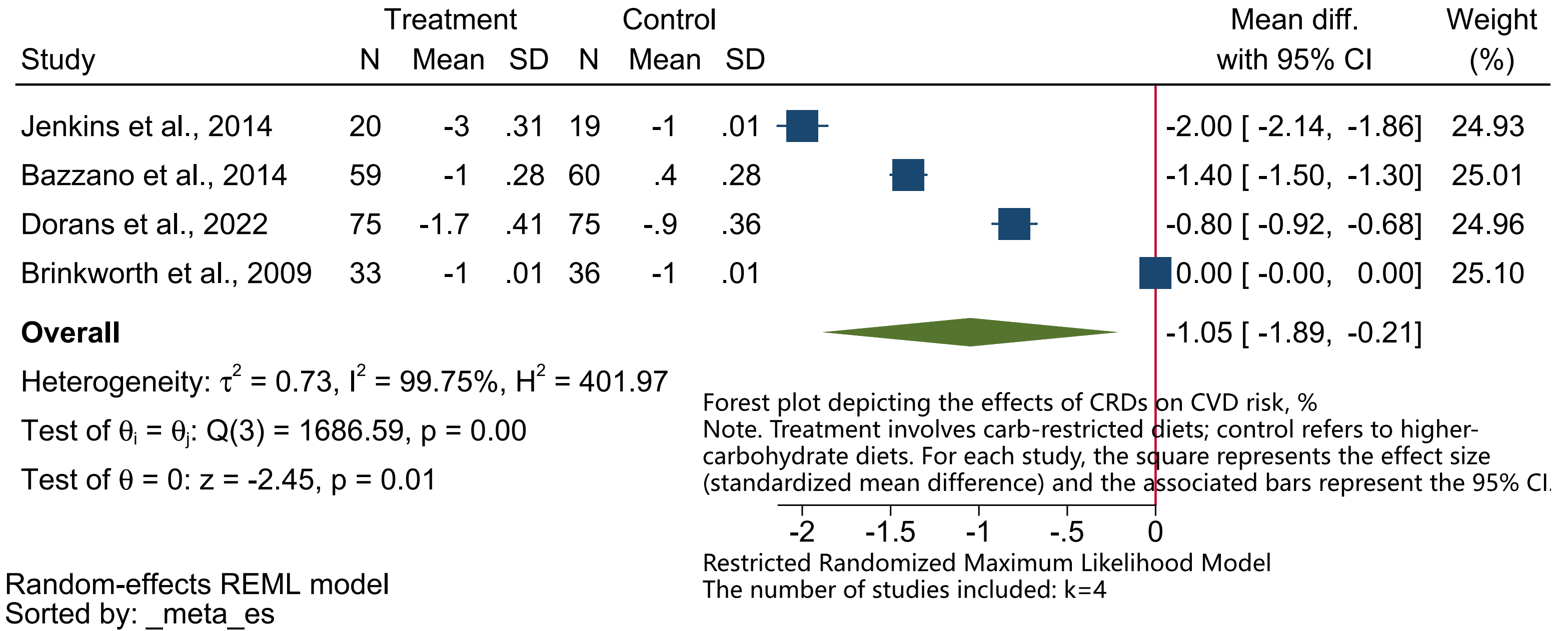
Treatment
Control
Mean diff. with 95% CI
Weight (%)
Study
N Mean SD N Mean SD
Jenkins et al., 2014 20 -3 .31 19 -1 .01 -2.00 [-2.14, -1.86] 24.93
Bazzano et al., 2014 59 -1 .28 60 .4 .28 -1.40 [-1.50, -1.30] 25.01
Dorans et al., 2022 75 -1.7 .41 75 -.9 .36 -0.80 [-0.92, -0.68] 24.96
Brinkworth et al., 2009 33 -1 .01 36 -1 .01 0.00 [-0.00, 0.00] 25.10
Overall -1.05 [-1.89, -0.21]
Heterogeneity: τ^2 = 0.73, I^2 = 99.75%, H^2 = 401.97
Test of $\theta_i = \theta_j$: Q(3) = 1686.59, p = 0.00
Test of θ = 0: z = -2.45, p = 0.01
Forest plot depicting the effects of CRDs on CVD risk, %
Note. Treatment involves carb-restricted diets; control refers to higher-carbohydrate diets. For each study, the square represents the effect size (standardized mean difference) and the associated bars represent the 95% CI.
-2 -1.5 -1 -.5 0
Restricted Randomized Maximum Likelihood Model
The number of studies included: k=4
Random-effects REML model
Sorted by: _meta_es

Study	Treatment N	Treatment Mean	Treatment SD	Control N	Control Mean	Control SD	Mean diff. with 95% CI	Weight (%)
D'Abbondanza et al., 2020	28	124	20	28	137	18	-13.00 [-22.97, -3.03]	0.16
Michalczyk et al., 2020b	46	-13.72	2.33	45	-.77	1.6	-12.95 [-13.77, -12.13]	0.90
Kleissl-Muir et al., 2023	8	-10.9	11.26	5	.4	2.91	-11.30 [-21.53, -1.07]	0.16
D'Abbondanza et al., 2020	42	109	20	42	119	20	-10.00 [-18.55, -1.45]	0.21
Goday et al., 2016	45	-14.7	2.71	40	-5.05	2.89	-9.65 [-10.84, -8.46]	0.87
Saslow et al., 2023	23	-19.14	8.3	25	-10.34	8.65	-8.80 [-13.60, -4.00]	0.44
Cunha et al., 2020	20	-9.7	3.88	19	-1.67	2.22	-8.03 [-10.03, -6.03]	0.78
Morris et al., 2020	21	-9.5	5.4	12	-2	2.5	-7.50 [-10.75, -4.25]	0.62
Li et al., 2022	24	-8.06	2.18	29	-.61	2.47	-7.45 [-8.72, -6.18]	0.86
Saslow et al., 2017	16	-7.9	.83	18	-1.7	.78	-6.20 [-6.74, -5.66]	0.91
Dorans et al., 2022	75	-6.4	.69	75	-.5	.36	-5.90 [-6.08, -5.72]	0.92
Iqbal et al., 2005	39	-8.88	3.58	32	-3.56	5.1	-5.32 [-7.34, -3.30]	0.78
Goss et al., 2020	19	-5.9	3.25	15	-.9	4.88	-5.00 [-7.74, -2.26]	0.68
Guevara-Cruz 2024	11	-5.2	2.54	11	-.3	1.55	-4.90 [-6.66, -3.14]	0.81
Gram-Kampmann et al., 2022	44	-5.1	3.72	20	-.2	2.78	-4.90 [-6.73, -3.07]	0.80
Krebs et al., 2016	5	-5.2	4.75	5	-.4	3.61	-4.80 [-10.03, 0.43]	0.41
Lundanes et al., 2025	30	-11.8	1.17	25	-7.5	1.3	-4.30 [-4.95, -3.65]	0.91
Zainordin et al., 2021	14	-4	2.89	16	.2	3.11	-4.20 [-6.36, -2.04]	0.76
Wycherley et al., 2014	23	-15.9	10.55	20	-12.1	7.16	-3.80 [-9.28, 1.68]	0.38
Wekesa et al., 2016	24	70.1	8.82	24	73.8	8.82	-3.70 [-8.69, 1.29]	0.43
Röhling et al., 2020	65	-5.6	.54	28	-1.9	.79	-3.70 [-3.98, -3.42]	0.92
Pinsawas et al., 2024	26	-4	2.77	22	-.4	3.01	-3.60 [-5.24, -1.96]	0.82
Dening et al., 2023	40	-4.36	3.66	47	-.77	3.52	-3.59 [-5.10, -2.08]	0.83
Bazzano et al., 2014	59	-5.3	.77	60	-1.8	.77	-3.50 [-3.78, -3.22]	0.92
Hu et al., 2013	75	-5.3	.77	73	-1.8	.77	-3.50 [-3.75, -3.25]	0.92
Zinn et al., 2017	14	-5.5	2.32	12	-2	1.74	-3.50 [-5.10, -1.90]	0.83
Dellis et al., 2020	35	-9.35	.51	35	-5.9	.54	-3.45 [-3.70, -3.20]	0.92
de Luis et al., 2015	168	-8.4	4.2	163	-5	4.1	-3.40 [-4.29, -2.51]	0.89
Wolever et al., 2008	53	-.4	17.49	55	2.8	17.07	-3.20 [-9.72, 3.32]	0.31
Lambadiari et al., 2024	16	98.17	17.46	16	101.21	17.95	-3.04 [-15.31, 9.23]	0.12
Jönsson et al., 2009	13	81	13	13	84	15	-3.00 [-13.79, 7.79]	0.14
Otten et al., 2016	25	-8	1.79	16	-5	2.34	-3.00 [-4.27, -1.73]	0.86
Sun et al., 2019	15	-2.85	1.5	15	.09	1.25	-2.94 [-3.93, -1.95]	0.88
Saslow et al., 2014	15	-5.5	4.68	18	-2.6	3.48	-2.90 [-5.69, -0.11]	0.68
Ahmad 2020	25	-2.1	2.5	25	.7	1.5	-2.80 [-3.94, -1.66]	0.87
Buscemi et al., 2009	10	-7.6	2.53	10	-4.9	1.9	-2.70 [-4.66, -0.74]	0.78
Alnoubi et al., 2024	14	-10.36	1.4	14	-7.71	1.09	-2.65 [-3.58, -1.72]	0.89
Perissiou et al., 2020	33	-4.4	4	31	-1.8	2.5	-2.60 [-4.25, -0.95]	0.82
Brinkworth et al., 2010	33	-14.1	2.8	35	-11.5	1.76	-2.60 [-3.70, -1.50]	0.87
Kikuchi 2023	21	-8	1.93	21	-5.4	3.34	-2.60 [-4.25, -0.95]	0.82
Iacovides et al., 2022	11	-2.9	.28	11	-.4	.28	-2.50 [-2.73, -2.27]	0.92
Meckling et al., 2007	10	-4.6	2.31	8	-2.1	1.58	-2.50 [-4.38, -0.62]	0.79
Mousavi et al., 2023	35	-4.82	1.42	35	-2.4	1.42	-2.42 [-3.09, -1.75]	0.91
Michalczyk et al., 2020a	18	-2.76	1.19	20	-.34	.76	-2.42 [-3.05, -1.79]	0.91
Turton et al., 2023	16	91.4	17.7	16	93.8	18.7	-2.40 [-15.02, 10.22]	0.11
Wal et al., 2007	42	-2.94	2.25	44	-.61	1.33	-2.33 [-3.11, -1.55]	0.90
Cai et al., 2021	22	-3.56	1.74	22	-1.24	1.69	-2.32 [-3.33, -1.31]	0.88
Baba et al., 1999	7	-8.3	1.85	6	-6	1.47	-2.30 [-4.14, -0.46]	0.80
McAuley et al., 2005	30	-6.6	1.57	31	-4.3	2.18	-2.30 [-3.26, -1.34]	0.89
Claessens et al., 2009	32	-1.09	3.34	16	1.19	2.24	-2.28 [-4.10, -0.46]	0.80
Luong et al., 2024	11	93.7	40.46	11	95.9	40.46	-2.20 [-36.01, 31.61]	0.02
DalleGrave et al., 2013	43	-18.1	14.3	45	-15.9	10.1	-2.20 [-7.35, 2.95]	0.41
Chen et al., 2020	43	-2.76	.96	42	-.71	.35	-2.05 [-2.36, -1.74]	0.92
Michalopoulou et al., 2024	28	2.3	.56	17	4.3	.99	-2.00 [-2.45, -1.55]	0.92
Genoni et al., 2016	22	-3.2	.31	17	-1.21	.38	-1.99 [-2.21, -1.77]	0.92
Crabtree et al., 2021	13	-8	1.43	12	-6.1	2.24	-1.90 [-3.36, -0.44]	0.84
Holmer et al., 2021	22	-9.3	1.22	24	-7.4	.69	-1.90 [-2.47, -1.33]	0.91
McManus et al., 2001	25	-4.8	5.6	10	-2.9	8.6	-1.90 [-6.71, 2.91]	0.44
Ranjan et al., 2017	10	72.9	10.3	10	74.8	9.6	-1.90 [-10.63, 6.83]	0.20
Jenkins et al., 2014	20	-6.8	.39	19	-5	.45	-1.80 [-2.06, -1.54]	0.92
Lim et al., 2010	18	-3.9	26.83	17	-2.1	19.39	-1.80 [-17.39, 13.79]	0.07
YancyWSJr et al., 2010	57	-11.37	1.77	65	-9.62	1.18	-1.75 [-2.28, -1.22]	0.91
Hyde et al., 2021	12	-2.2	2.01	12	-.5	1.77	-1.70 [-3.22, -0.18]	0.83
Brinkworth et al., 2004a	19	-3.8	2.67	19	-2.1	2.87	-1.70 [-3.46, 0.06]	0.81
He et al., 2022	44	-5	2.65	44	-3.4	2.65	-1.60 [-2.71, -0.49]	0.87
Chen et al., 2022	36	-2.4	2.49	35	-.8	1.72	-1.60 [-2.60, -0.60]	0.88
McMillan-Price et al., 2006	31	-5.3	2.78	27	-3.7	2.6	-1.60 [-2.99, -0.21]	0.85
Valsdottir et al., 2023	14	-6.5	1.1	15	-5.1	1.39	-1.40 [-2.32, -0.48]	0.89
Brinkworth et al., 2009	32	-8.1	2.96	28	-6.7	1.76	-1.40 [-2.66, -0.14]	0.86
Elhayany et al., 2010	61	-8.9	2.28	55	-7.6	1.97	-1.30 [-2.08, -0.52]	0.90
Lean et al., 1997	40	-6.8	.82	42	-5.6	.61	-1.20 [-1.51, -0.89]	0.92
Yamada et al., 2014	12	-2.6	2.72	12	-1.4	1.25	-1.20 [-2.89, 0.49]	0.81
McAuley et al., 2005	31	-6.6	1.57	30	-5.4	2.15	-1.20 [-2.14, -0.26]	0.89
Harvey et al., 2019	14	-4.12	.75	12	-2.97	1.05	-1.15 [-1.84, -0.46]	0.90
Li et al., 2024	22	-3.7	.48	23	-2.57	.46	-1.13 [-1.40, -0.86]	0.92
Foster et al., 2010	153	-9.49	.32	154	-8.37	.34	-1.12 [-1.19, -1.05]	0.92
Martins et al., 2025	26	-6	2.64	24	-4.9	2.67	-1.10 [-2.57, 0.37]	0.84
Boers et al., 2014	18	-2.7	2.62	16	-1.7	2.54	-1.00 [-2.74, 0.74]	0.81
Golay et al., 1996	22	-8	5.55	21	-7	5.1	-1.00 [-4.19, 2.19]	0.63
Haufe et al., 2011	52	-7.5	4.33	50	-6.5	4.95	-1.00 [-2.80, 0.80]	0.80
Haufe et al., 2012	41	-7	2.28	49	-6	2.54	-1.00 [-2.01, 0.01]	0.88
Bradley et al., 2009	12	-7.4	2.44	12	-6.5	1.58	-0.90 [-2.54, 0.74]	0.82
Kleiner et al., 2006	9	-4.9	11.93	7	-4	11.29	-0.90 [-12.42, 10.62]	0.13
deLuis et al., 2007	47	-4.5	2.16	43	-3.6	2.54	-0.90 [-1.87, 0.07]	0.88
Krebs et al., 2012	152	-3.2	3.26	158	-2.4	2.94	-0.80 [-1.49, -0.11]	0.90
deLuis et al., 2015	61	-6.8	2.75	56	-6	2.11	-0.80 [-1.69, 0.09]	0.89
Lim et al., 2010	18	-2.9	20.79	17	-2.1	19.39	-0.80 [-14.14, 12.54]	0.10
Gardner et al., 2018	304	-5.99	.33	305	-5.29	.33	-0.70 [-0.75, -0.65]	0.92
Heggen et al., 2016	64	-.2	3.3	57	.5	2.6	-0.70 [-1.77, 0.37]	0.88
Skytte et al., 2019	14	-1.4	.75	14	-.8	.75	-0.60 [-1.16, -0.04]	0.91
Keogh et al., 2007	13	-8.5	16.22	12	-7.9	3.81	-0.60 [-10.02, 8.82]	0.18
Karl et al., 2015	22	-5.8	.64	24	-5.3	.82	-0.50 [-0.93, -0.07]	0.92
Tay et al., 2014b	46	-12	6.3	47	-11.5	5.5	-0.50 [-2.90, 1.90]	0.73
Wolever et al., 2008	53	-.4	17.49	48	-.1	15.95	-0.30 [-6.85, 6.25]	0.31
OmniHeartCollaborativeResGrp 2005	164	86	18.2	164	86.3	18.2	-0.30 [-4.24, 3.64]	0.54
Rodriguez-Villar et al., 2004	22	77.8	13.9	22	78.1	14	-0.30 [-8.54, 7.94]	0.22
Meckling et al., 2004	15	-7	2.47	16	-6.8	1.7	-0.20 [-1.68, 1.28]	0.84
Tay et al., 2018	33	-6.8	1.05	28	-6.6	1.1	-0.20 [-0.74, 0.34]	0.91
Egert et al., 2011	18	-.2	1.91	19	0	1.43	-0.20 [-1.28, 0.88]	0.88
Walker et al., 1996	16	-1.3	.04	16	-1.1	.03	-0.20 [-0.22, -0.18]	0.92
Krauss et al., 2006	47	-5.4	.3	49	-5.3	.3	-0.10 [-0.22, 0.02]	0.92
Weber et al., 2022	28	-5.9	1.9	31	-5.8	2.3	-0.10 [-1.18, 0.98]	0.88
Chiu et al., 2014	32	-.7	2.26	31	-.6	2.78	-0.10 [-1.35, 1.15]	0.86
Numao et al., 2013	11	-.7	.84	11	-.6	.89	-0.10 [-0.82, 0.62]	0.90
deLuis et al., 2015	49	-5.1	6.04	45	-5	7.54	-0.10 [-2.85, 2.65]	0.68
Rajaie et al., 2013	30	-1.72	2.19	30	-1.7	1.97	-0.02 [-1.07, 1.03]	0.88
Davis et al., 2009	55	-3.1	4.8	50	-3.1	5.8	0.00 [-2.03, 2.03]	0.77
Haji-GhaziTehrani et al., 2022	24	-3.9	2.25	24	-3.9	3.28	0.00 [-1.59, 1.59]	0.83
Merovci et al., 2024	10	0	1.69	8	0	.87	0.00 [-1.29, 1.29]	0.86
Thomsen et al., 2022	34	-5.8	1.8	33	-5.8	2.3	0.00 [-0.99, 0.99]	0.88
White et al., 2007	9	-4	2.38	10	-4	2.82	0.00 [-2.36, 2.36]	0.73
Wachsmuth et al., 2022	18	-1.3	.9	18	-1.4	11.6	0.10 [-5.27, 5.47]	0.39
Archer et al 2003	32	-2.1	2.23	31	-2.2	2	0.10 [-0.95, 1.15]	0.88
Raygan et al., 2016	28	-2.1	1.7	28	-2.2	2.1	0.10 [-0.90, 1.10]	0.88
Garg et al., 1992	8	-1.2	1.85	8	-1.4	2.21	0.20 [-1.80, 2.20]	0.78
Rasmussen et al., 2007	12	1.2	3.9	15	1	4.2	0.20 [-2.89, 3.29]	0.64
Martens et al., 2015	15	0	1.29	17	-.2	1.43	0.20 [-0.75, 1.15]	0.89
Karl et al., 2015	21	-6.6	.71	24	-6.8	.79	0.20 [-0.24, 0.64]	0.92
Tay et al., 2014a	58	-9.8	.97	57	-10.1	.97	0.30 [-0.05, 0.65]	0.92
McMillan-Price et al., 2006	28	-4.4	2.65	30	-4.8	2.74	0.40 [-0.99, 1.79]	0.85
Thomson et al., 2010	19	-5.9	4.1	21	-6.3	5.6	0.40 [-2.67, 3.47]	0.64
Parr et al., 2016	29	-4.3	2.3	32	-4.7	2.4	0.40 [-0.78, 1.58]	0.87
OmniHeartCollaborativeResGrp 2005	164	86.7	18.4	164	86.3	18.2	0.40 [-3.56, 4.36]	0.53
Liu et al., 2013	25	-5.3	1.01	24	-5.8	1.28	0.50 [-0.14, 1.14]	0.91
Wycherley et al., 2016	58	-10.4	2.13	57	-10.9	2.53	0.50 [-0.35, 1.35]	0.89
Meksawan et al., 2004	10	64.9	13.6	10	64.3	13.28	0.60 [-11.18, 12.38]	0.12
Wolever et al., 2003	11	.27	1.49	11	-.49	.96	0.76 [-0.29, 1.81]	0.88
Retterstøl et al., 2018	15	-1.2	1.22	15	-2	1.13	0.80 [-0.04, 1.64]	0.89
Guldbrand et al., 2012	30	-2	4.17	31	-2.9	2.97	0.90 [-0.91, 2.71]	0.80
Gerhard et al., 2004	11	-.47	.93	11	-1.53	1.21	1.06 [0.16, 1.96]	0.89
Yancy Jr et al., 2004	45	.3	2.14	34	-1.1	2.89	1.40 [0.29, 2.51]	0.87
Le et al., 2016	81	-5.9	4.13	82	-7.7	1.84	1.80 [0.82, 2.78]	0.88
Bush et al., 2018	12	-.5	1.81	17	-4.1	1.58	3.60 [2.36, 4.84]	0.86
Peterson et al., 1995	13	-3.64	1.51	13	-9.09	8.85	5.45 [0.57, 10.33]	0.44
Peterson et al., 1995	12	1.36	3.01	12	-4.54	1.28	5.90 [4.05, 7.75]	0.80
**Overall**							-1.75 [-2.20, -1.31]	

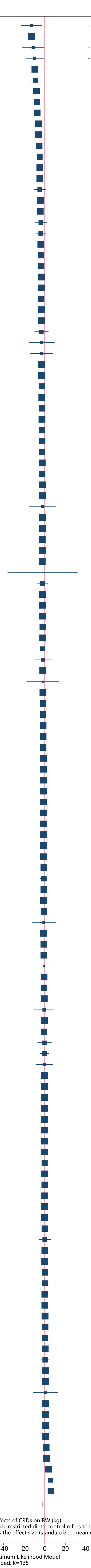

Heterogeneity: $\tau^2 = 5.57$, $I^2 = 99.56\%$, $H^2 = 227.88$

Test of $\theta_i = \theta_j$: Q(134) = 9883.85, p = 0.00

Test of $\theta = 0$: z = -7.72, p = 0.00

Random-effects REML model
Sorted by: _meta_es

Forest plot depicting the effects of CRDs on BW (kg)
Note. Treatment involves carb-restricted diets; control refers to higher-carbohydrate diets. For each study, the square represents the effect size (standardized mean difference) and the associated bars represent the 95% CI.

Restricted Randomized Maximum Likelihood Model
The number of studies included: k=135

Study	Treatment N	Treatment Mean	Treatment SD	Control N	Control Mean	Control SD	Mean diff. with 95% CI	Weight (%)
Michalczyk et al., 2020b	46	-5.05	1.02	45	-.29	.91	-4.76 [-5.16, -4.36]	1.21
D'Abbondanza et al., 2020	28	42	7	28	46	7	-4.00 [-7.67, -0.33]	0.26
D'Abbondanza et al., 2020	42	42	8	42	46	8	-4.00 [-7.42, -0.58]	0.29
Hansen et al., 2023	110	-5.5	.66	55	-1.7	.97	-3.80 [-4.05, -3.55]	1.24
Tay et al., 2014b	46	-4	2	47	-.4	1.8	-3.60 [-4.37, -2.83]	1.08
Goday et al., 2016	45	-5.4	.44	40	-1.9	.71	-3.50 [-3.75, -3.25]	1.24
Cunha et al., 2020	20	-3.56	1.34	19	-.71	.96	-2.85 [-3.59, -2.11]	1.09
Li et al., 2022	24	-2.83	1.16	29	-.33	1.21	-2.50 [-3.14, -1.86]	1.13
Guevara-Cruz 2024	11	-2	.91	11	-.1	.8	-1.90 [-2.62, -1.18]	1.10
McManus et al., 2001	25	-1.9	2.3	10	-.1	4.9	-1.80 [-4.16, 0.56]	0.48
Saslow et al., 2014	15	-1.9	1.75	18	-.1	1.28	-1.80 [-2.83, -0.77]	0.96
Saslow et al., 2017	16	-2.6	.34	18	-.9	.33	-1.70 [-1.93, -1.47]	1.25
Krebs et al., 2016	5	-1.7	1.24	5	-.1	1.23	-1.60 [-3.13, -0.07]	0.75
Gram-Kampmann et al., 2022	44	-1.8	1.19	20	-.3	4	-1.50 [-2.78, -0.22]	0.85
Zainordin et al., 2021	14	-1.5	.3	16	-.07	.39	-1.43 [-1.68, -1.18]	1.24
Wekesa et al., 2016	24	25.7	2.94	24	27.1	2.94	-1.40 [-3.06, 0.26]	0.70
Abete et al., 2009	19	-3	.38	19	-1.6	.68	-1.40 [-1.75, -1.05]	1.22
Otten et al., 2016	25	-3.1	.78	16	-1.8	.93	-1.30 [-1.83, -0.77]	1.17
Alnoubi et al., 2024	14	-4.07	.54	14	-2.8	.6	-1.27 [-1.69, -0.85]	1.20
Dening et al., 2023	40	-1.48	1.19	47	-.27	1.2	-1.21 [-1.71, -0.71]	1.18
Röhling et al., 2020	65	-1.9	.55	28	-.7	.28	-1.20 [-1.42, -0.98]	1.25
Sun et al., 2019	15	-1.09	.56	15	.02	.69	-1.11 [-1.56, -0.66]	1.19
Lambadiari et al., 2024	16	36.3	7.33	16	37.4	7.75	-1.10 [-6.33, 4.13]	0.14
Brinkworth et al., 2009a	33	-5.2	3.45	36	-4.1	2.4	-1.10 [-2.49, 0.29]	0.81
Brinkworth et al., 2010	33	-5.2	.9	35	-4.1	.77	-1.10 [-1.50, -0.70]	1.21
McAuley et al., 2005	30	-2.5	.78	31	-1.4	1.12	-1.10 [-1.59, -0.61]	1.18
Dellis et al., 2020	35	-3.14	.15	35	-2.09	.19	-1.05 [-1.13, -0.97]	1.26
Jönsson et al., 2009	13	28	5	13	29	6	-1.00 [-5.25, 3.25]	0.20
Michalczyk et al., 2020a	18	-1.4	.34	20	-.4	.28	-1.00 [-1.20, -0.80]	1.25
Mousavi et al., 2023	35	-1.88	.59	35	-.94	.59	-0.94 [-1.22, -0.66]	1.24
Valsdottir et al., 2023	14	-2.6	.75	15	-1.7	.52	-0.90 [-1.37, -0.43]	1.19
Ahmad 2020	25	-.7	.4	25	.2	.5	-0.90 [-1.15, -0.65]	1.24
Gardner et al., 2007	77	-1.65	2.54	76	-.77	2.14	-0.88 [-1.62, -0.14]	1.09
Boers et al., 2014	18	-.9	1.17	16	-.1	1.05	-0.80 [-1.55, -0.05]	1.09
Turton et al., 2023	16	31.1	5.6	16	31.9	5.9	-0.80 [-4.79, 3.19]	0.22
Cai et al., 2021	22	-1.17	.52	22	-.41	.61	-0.76 [-1.09, -0.43]	1.22
Chen et al., 2020	43	-1.2	.36	42	-.49	.19	-0.71 [-0.83, -0.59]	1.26
Baba et al., 1999	7	-2.7	.53	6	-2	.49	-0.70 [-1.26, -0.14]	1.16
Ruth et al., 2013	18	-2.5	1.5	15	-1.8	1.5	-0.70 [-1.73, 0.33]	0.97
Luong et al., 2024	11	31.6	4.5	11	32.3	4.3	-0.70 [-4.38, 2.98]	0.26
Brinkworth et al., 2009b	32	-2.9	.93	28	-2.3	.92	-0.60 [-1.07, -0.13]	1.19
Meckling et al., 2007	10	-1.7	.97	8	-1.1	.72	-0.60 [-1.41, 0.21]	1.06
Wal et al., 2007	42	-1.02	.84	44	-.47	1.9	-0.55 [-1.18, 0.08]	1.13
DalleGrave et al., 2013	43	-6.2	4.5	45	-5.7	3.3	-0.50 [-2.14, 1.14]	0.71
de Luis et al., 2015	168	-2.6	1.3	163	-2.1	1.2	-0.50 [-0.77, -0.23]	1.24
deLuis et al., 2007	47	-1.7	1.11	43	-1.2	1.17	-0.50 [-0.97, -0.03]	1.19
Elhayany et al., 2010	61	-3.3	.56	55	-2.8	.66	-0.50 [-0.72, -0.28]	1.25
Jenkins et al., 2014	20	-2.4	.15	19	-1.9	.13	-0.50 [-0.59, -0.41]	1.26
Kikuchi 2023	21	-2.6	.23	21	-2.1	.36	-0.50 [-0.68, -0.32]	1.25
Kitabchi et al., 2013	12	-4	1.33	12	-3.5	1.06	-0.50 [-1.46, 0.46]	0.99
Li et al., 2024	22	-1.34	.17	23	-.91	.16	-0.43 [-0.53, -0.33]	1.26
Ranjan et al., 2017	10	23.9	1.7	10	24.3	1.5	-0.40 [-1.81, 1.01]	0.80
He et al., 2022	44	-1.8	1.32	44	-1.4	1.99	-0.40 [-1.11, 0.31]	1.10
Chen et al., 2022	36	-1	1	35	-.6	.8	-0.40 [-0.82, 0.02]	1.20
Frisch et al., 2009	85	-1.9	2.1	80	-1.5	1.8	-0.40 [-1.00, 0.20]	1.14
Holmer et al., 2021	22	-2.8	.41	24	-2.4	.2	-0.40 [-0.58, -0.22]	1.25
Lean et al., 1997	40	-2.6	.33	42	-2.2	.28	-0.40 [-0.53, -0.27]	1.26
McAuley et al., 2005	31	-2.5	.78	30	-2.1	1.13	-0.40 [-0.89, 0.09]	1.18
Harvey et al., 2019	14	-1.39	.25	12	-1.03	.37	-0.36 [-0.60, -0.12]	1.24
Gardner et al., 2018	304	-2.07	.11	305	-1.75	.11	-0.32 [-0.34, -0.30]	1.26
Bradley et al., 2009	12	-2.6	.86	12	-2.3	.6	-0.30 [-0.89, 0.29]	1.15
Haufe et al., 2011	52	-2.7	1.44	50	-2.4	1.41	-0.30 [-0.85, 0.25]	1.16
Haufe et al., 2012	41	-2.6	.9	49	-2.3	.8	-0.30 [-0.65, 0.05]	1.22
Martins et al., 2025	26	-2	1.29	24	-1.7	1.23	-0.30 [-1.00, 0.40]	1.11
Yamada et al., 2014	12	-.9	1.11	12	-.6	.95	-0.30 [-1.13, 0.53]	1.05
Rajaie et al., 2013	30	-.96	.88	30	-.67	.77	-0.29 [-0.71, 0.13]	1.20
Archer et al 2003	32	-.9	1.05	31	-.7	.86	-0.20 [-0.67, 0.27]	1.19
Skytte et al., 2019	14	-.5	.37	14	-.3	.37	-0.20 [-0.47, 0.07]	1.24
Walker et al., 1996	16	-.5	.08	16	-.4	.04	-0.10 [-0.14, -0.06]	1.26
Rodriguez-Villar et al., 2004	22	27.8	2.9	22	27.9	2.9	-0.10 [-1.81, 1.61]	0.68
Chiu et al., 2014	32	-.1	.57	31	-.1	.56	0.00 [-0.28, 0.28]	1.24
Haji-GhaziTehrani et al., 2022	24	-1.5	.88	24	-1.5	1.27	0.00 [-0.62, 0.62]	1.14
Merovci et al., 2024	10	0	.63	8	0	1.03	0.00 [-0.77, 0.77]	1.08
Numao et al., 2013	11	-.2	.33	11	-.2	.33	0.00 [-0.28, 0.28]	1.24
Raygan et al., 2016	28	-.9	.7	28	-.9	.8	0.00 [-0.39, 0.39]	1.21
Thomsen et al., 2022	34	-2	.6	33	-2	.6	0.00 [-0.29, 0.29]	1.23
Thomson et al., 2010	19	-2.3	1.6	21	-2.3	2.1	0.00 [-1.17, 1.17]	0.90
Weber et al., 2022	28	-2	.6	31	-2	.6	0.00 [-0.31, 0.31]	1.23
White et al., 2007	9	-1.1	.96	10	-1.1	1.05	0.00 [-0.91, 0.91]	1.02
Yancy Jr et al., 2004	45	0	1.05	34	-.1	1.05	0.10 [-0.37, 0.57]	1.19
deLuis et al., 2015	49	-2.1	1.38	45	-2.2	2.08	0.10 [-0.61, 0.81]	1.10
Meckling et al., 2004	15	-2.1	1.26	16	-2.2	.72	0.10 [-0.62, 0.82]	1.10
Liu et al., 2013	25	-2.2	.67	24	-2.4	.4	0.20 [-0.11, 0.51]	1.23
Tay et al., 2018	33	-2.1	.33	28	-2.3	.36	0.20 [0.03, 0.37]	1.25
Guldbrand et al., 2012	30	-.8	1.34	31	-1	1.14	0.20 [-0.42, 0.82]	1.13
Parr et al., 2016	29	-1.5	.7	32	-1.7	.8	0.20 [-0.18, 0.58]	1.21
Retterstøl et al., 2018	15	-.4	.29	15	-.6	.32	0.20 [-0.02, 0.42]	1.25
Bush et al., 2018	12	-.2	.54	17	-.4	.52	0.20 [-0.19, 0.59]	1.21
Veum et al., 2017	20	-3.5	.66	18	-3.7	.75	0.20 [-0.25, 0.65]	1.19
Tay et al., 2015	41	-3.2	.33	37	-3.5	.33	0.30 [0.15, 0.45]	1.26
Rasmussen et al., 2007	12	.8	1.3	15	.3	1.4	0.50 [-0.53, 1.53]	0.96
deLuis et al., 2015	61	-2.2	2.24	56	-2.8	.82	0.60 [-0.02, 1.22]	1.14
Le et al., 2016	81	-2.2	1.21	82	-2.8	1.08	0.60 [0.25, 0.95]	1.22
**Overall**							-0.72 [-0.93, -0.51]	

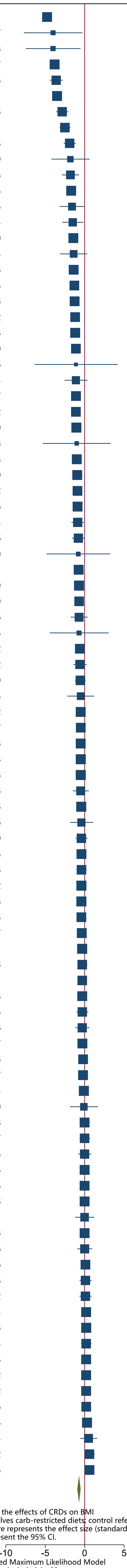

Heterogeneity: $\tau^2 = 0.89$, $I^2 = 99.01\%$, $H^2 = 100.57$

Test of $\theta_i = \theta_j$: Q(92) = 3211.56, p = 0.00

Test of $\theta = 0$: z = -6.79, p = 0.00

Forest plot depicting the effects of CRDs on BMI
Note. Treatment involves carb-restricted diets; control refers to higher-carbohydrate diet
each study, the square represents the effect size (standardized mean difference) and the associated bars represent the 95% CI.

Random-effects REML model
Sorted by: _meta_es

Restricted Randomized Maximum Likelihood Model
The number of studies included: k=93

Study	Treatment N	Treatment Mean	Treatment SD	Control N	Control Mean	Control SD	Mean diff. with 95% CI	Weight (%)
Michalczyk et al., 2020	18	-4.22	.77	20	.76	.87	-4.98 [-5.50, -4.46]	1.92
Lundanes et al., 2025	30	-9	.82	25	-5.4	.89	-3.60 [-4.05, -3.15]	1.94
Goss et al., 2020	19	-4.1	1.94	15	-.8	1.06	-3.30 [-4.39, -2.21]	1.68
Valsdottir et al., 2020	14	-6.3	2.67	15	-3.1	.86	-3.20 [-4.62, -1.78]	1.51
Swenson et al., 2007	19	-29.2	9.6	13	-26.2	7.3	-3.00 [-9.17, 3.17]	0.28
Brehm et al., 2005	20	-6.19	1.35	20	-3.24	1.92	-2.95 [-3.98, -1.92]	1.71
Röhling et al., 2020	65	-4.5	.43	28	-1.6	.64	-2.90 [-3.12, -2.68]	1.98
Wycherley et al., 2014	23	-12.6	9.11	20	-10.2	7.16	-2.40 [-7.35, 2.55]	0.40
Cohen et al., 2018	25	-5.2	3.19	20	-2.9	5.12	-2.30 [-4.74, 0.14]	1.02
Abete et al., 2009	19	-5.8	.73	19	-3.5	.83	-2.30 [-2.80, -1.80]	1.92
Claessens et al., 2009	32	-1.96	2.94	16	.24	2.8	-2.20 [-3.94, -0.46]	1.35
Golay et al., 1996	22	-9	5.22	21	-7	1.83	-2.00 [-4.36, 0.36]	1.06
Brinkworth et al., 2009a	33	-11.3	8.62	36	-9.4	7.2	-1.90 [-5.64, 1.84]	0.62
Ahmad 2020	25	-1.4	1.5	25	.5	1.5	-1.90 [-2.73, -1.07]	1.80
Gram-Kampmann et al., 2022	44	-2.9	2.84	20	-1	2.59	-1.90 [-3.36, -0.44]	1.49
Lambadiari et al., 2024	16	40.51	12.8	16	42.3	12.84	-1.79 [-10.67, 7.09]	0.15
He et al., 2022	44	-3	3.32	44	-1.3	3.98	-1.70 [-3.23, -0.17]	1.45
Genoni et al., 2016	22	-2.14	.28	17	-.46	.46	-1.68 [-1.91, -1.45]	1.98
McMillan-Price et al., 2006	31	-4.3	2.78	27	-2.8	2.6	-1.50 [-2.89, -0.11]	1.53
Meckling et al., 2007	10	-5.2	1.8	8	-3.7	2.9	-1.50 [-3.68, 0.68]	1.13
Dellis et al., 2020	35	-6.57	.39	35	-5.12	.57	-1.45 [-1.68, -1.22]	1.98
McAuley et al., 2005	31	-4.4	1.01	30	-3.1	1.13	-1.30 [-1.84, -0.76]	1.91
Otten et al., 2016	25	-5.2	1.09	16	-3.9	2	-1.30 [-2.25, -0.35]	1.75
Brinkworth et al., 2004a	19	-1.4	2.35	19	-.1	2.27	-1.30 [-2.77, 0.17]	1.48
McManus et al., 2001	25	-4.2	5.1	10	-3	6.3	-1.20 [-5.20, 2.80]	0.56
Noakes et al., 2005	52	-5.7	4.33	48	-4.5	3.46	-1.20 [-2.74, 0.34]	1.45
Li et al., 2024	22	-3.18	.48	23	-2.03	.47	-1.15 [-1.43, -0.87]	1.98
Luong et al., 2024	11	35.1	9.8	11	36.2	9.5	-1.10 [-9.17, 6.97]	0.17
Krebs et al., 2012	152	-2.8	2.28	158	-1.7	2.01	-1.10 [-1.58, -0.62]	1.93
McAuley et al., 2005	30	-4.4	1.01	31	-3.4	1.4	-1.00 [-1.61, -0.39]	1.89
Brinkworth et al., 2009b	32	-6.1	2.06	28	-5.1	1.59	-1.00 [-1.94, -0.06]	1.75
Frisch et al., 2009	85	-4.2	4.9	80	-3.3	4.4	-0.90 [-2.32, 0.52]	1.51
Martins et al., 2025	26	-4.2	2.06	24	-3.4	1.8	-0.80 [-1.88, 0.28]	1.69
Heggen et al., 2016	64	-.4	2.7	57	.4	2.3	-0.80 [-1.70, 0.10]	1.77
Hyde et al., 2021	12	-1	.83	12	-.2	1.66	-0.80 [-1.85, 0.25]	1.70
Martens et al., 2015	15	-.4	.79	17	.4	1.04	-0.80 [-1.45, -0.15]	1.87
Brinkworth et al., 2004b	21	3.5	6.94	22	4.3	2.3	-0.80 [-3.86, 2.26]	0.80
deLuis et al., 2007	47	-3	1.59	43	-2.3	2.03	-0.70 [-1.45, 0.05]	1.83
Parr et al., 2016	29	-4.9	2.3	32	-4.3	1.7	-0.60 [-1.61, 0.41]	1.72
Kleiner et al., 2006	9	-3	2.37	7	-2.4	2.33	-0.60 [-2.92, 1.72]	1.07
Tay et al., 2014b	46	-10.2	5.7	47	-9.6	5.2	-0.60 [-2.82, 1.62]	1.12
Foster et al., 2010	153	-8.65	.4	154	-8.16	.47	-0.49 [-0.59, -0.39]	2.00
Ruth et al., 2013	18	-5.2	4	15	-4.8	3.1	-0.40 [-2.88, 2.08]	1.01
Thomsen et al., 2022	34	-4.3	1.1	32	-3.9	2	-0.40 [-1.17, 0.37]	1.82
Numao et al., 2013	11	-.4	.66	11	-.2	.73	-0.20 [-0.78, 0.38]	1.90
Bradley et al., 2009	12	-3.9	1.33	12	-3.8	.93	-0.10 [-1.02, 0.82]	1.76
Crabtree et al., 2021	13	-4.3	1.66	12	-4.2	1.73	-0.10 [-1.43, 1.23]	1.56
Haufe et al., 2012	41	-4.5	1.66	49	-4.4	2.39	-0.10 [-0.97, 0.77]	1.78
Walker et al., 1996	16	-.87	1.6	16	-.83	1.48	-0.04 [-1.11, 1.03]	1.69
Karl et al., 2015	22	-4.6	.56	24	-4.7	.61	0.10 [-0.24, 0.44]	1.96
Karl et al., 2015	21	-4.9	.56	24	-5.1	.69	0.20 [-0.17, 0.57]	1.96
Meksawan et al., 2004	10	12.6	2.85	10	12.4	3.48	0.20 [-2.59, 2.99]	0.89
Tay et al., 2018	33	-4.3	.97	28	-4.6	.99	0.30 [-0.19, 0.79]	1.92
Bush et al., 2018	12	-.7	1.07	17	-1	1.02	0.30 [-0.47, 1.07]	1.83
Rasmussen et al., 2007	12	1	4.8	15	.6	3.4	0.40 [-2.69, 3.49]	0.79
Liu et al., 2013	25	-2.5	.8	24	-3	.88	0.50 [0.03, 0.97]	1.93
Veum et al., 2017	20	-10.1	1.02	18	-10.6	1.71	0.50 [-0.38, 1.38]	1.78
Retterstøl et al., 2018	15	-.4	.65	15	-1	1.1	0.60 [-0.05, 1.25]	1.87
White et al., 2007	9	-2.6	1.85	10	-3.2	2.52	0.60 [-1.41, 2.61]	1.21
Tay et al., 2015	41	-7.9	.94	37	-8.6	.94	0.70 [0.28, 1.12]	1.95
deLuis et al., 2015	49	-3.6	1.43	45	-4.3	4.96	0.70 [-0.75, 2.15]	1.50
McMillan-Price et al., 2006	28	-3.7	2.65	30	-4.5	2.74	0.80 [-0.59, 2.19]	1.53
Kitabchi et al., 2013	12	-6.6	2.41	12	-7.5	2.62	0.90 [-1.11, 2.91]	1.21
deLuis et al., 2015	61	-3.5	1.3	56	-4.8	1.44	1.30 [0.80, 1.80]	1.92
Meckling et al., 2004	15	-4.1	2.47	16	-5.4	1.77	1.30 [-0.21, 2.81]	1.47
de Luis et al., 2015	168	4.4	1.81	163	2.3	2.37	2.10 [1.65, 2.55]	1.94
**Overall**							-0.84 [-1.19, -0.49]	

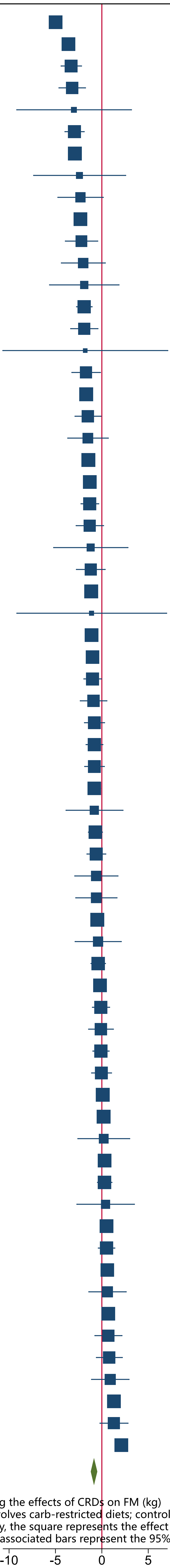

Heterogeneity: $\tau^2 = 1.62$, $I^2 = 95.59\%$, $H^2 = 22.67$

Test of $\theta_i = \theta_j$: $Q(65) = 1415.26$, $p = 0.00$

Test of $\theta = 0$: $z = -4.68$, $p = 0.00$

Forest plot depicting the effects of CRDs on FM (kg)
Note. Treatment involves carb-restricted diets; control refers to higher-carbohydrate diets. For each study, the square represents the effect size (standardized mean difference) and the associated bars represent the 95% CI.

Restricted Randomized Maximum Likelihood Model
The number of studies included: k=66

Random-effects REML model
Sorted by: _meta_es

Study	Treatment N	Treatment Mean	Treatment SD	Control N	Control Mean	Control SD	Mean diff. with 95% CI	Weight (%)
D'Abbondanza et al., 2020	28	40	7	28	44	9	-4.00 [-8.22, 0.22]	0.45
Michalczyk et al., 2020	18	-3.66	.74	20	-.27	1.25	-3.39 [-4.05, -2.73]	2.43
D'Abbondanza et al., 2020	42	47	5	42	50	5	-3.00 [-5.14, -0.86]	1.18
Guevara-Cruz 2024	11	-3.2	.86	11	-.2	.78	-3.00 [-3.69, -2.31]	2.41
Valsdottir et al., 2020	14	-2.5	1.02	15	.2	1.61	-2.70 [-3.69, -1.71]	2.14
Buscemi et al., 2009	10	-3.3	2.39	10	-1	1.26	-2.30 [-3.97, -0.63]	1.52
Meckling et al., 2007	10	-4.6	2.43	8	-2.5	2.32	-2.10 [-4.31, 0.11]	1.14
Claessens et al., 2009	32	-1.86	2.6	16	-.14	1.88	-1.72 [-3.15, -0.29]	1.72
Lundanes et al., 2025	30	-3.6	.43	25	-1.9	.48	-1.70 [-1.94, -1.46]	2.69
Chen et al., 2020	43	-1.17	.77	42	.38	.31	-1.55 [-1.80, -1.30]	2.69
Bazzano et al., 2014	59	-1.2	.41	60	.3	.41	-1.50 [-1.65, -1.35]	2.72
Hu et al., 2013	75	-1.2	.41	73	.3	.41	-1.50 [-1.63, -1.37]	2.72
Ahmad 2020	25	-1	1	25	.4	1.5	-1.40 [-2.11, -0.69]	2.39
Gardner et al., 2007	77	-2.9	4.8	76	-1.5	4	-1.40 [-2.80, 0.00]	1.75
Genoni et al., 2016	22	-1.48	.36	17	-.14	.51	-1.34 [-1.61, -1.07]	2.68
Wycherley et al., 2014	23	-7.5	5.75	20	-6.2	4.92	-1.30 [-4.52, 1.92]	0.69
Chen et al., 2022	36	-.8	1.24	35	.4	1.23	-1.20 [-1.77, -0.63]	2.50
Kleiner et al., 2006	9	-1.5	1.34	7	-.4	1.18	-1.10 [-2.36, 0.16]	1.88
Skytte et al., 2019	14	-.5	1.5	14	.6	1.5	-1.10 [-2.21, 0.01]	2.02
Hansen et al., 2023	110	-1.3	.22	55	-.27	.36	-1.03 [-1.12, -0.94]	2.73
Li et al., 2024	22	-2.92	.62	23	-1.92	.6	-1.00 [-1.36, -0.64]	2.64
Parr et al., 2016	29	-3.8	2.5	32	-2.9	1.8	-0.90 [-1.99, 0.19]	2.04
Wal et al., 2007	42	-1.22	1.21	44	-.47	1.5	-0.75 [-1.33, -0.17]	2.50
Krebs et al., 2012	152	-1.3	1.18	158	-.6	1.61	-0.70 [-1.02, -0.38]	2.66
Perissiou et al., 2020	33	-2	2	31	-1.3	1.4	-0.70 [-1.55, 0.15]	2.27
Thomsen et al., 2022	34	-2.3	1.1	32	-1.8	1.4	-0.50 [-1.11, 0.11]	2.48
Lean et al., 1997	40	-2.9	.33	41	-2.5	.33	-0.40 [-0.54, -0.26]	2.72
Jenkins et al., 2014	20	-4.2	.41	19	-3.9	.47	-0.30 [-0.58, -0.02]	2.68
Gram-Kampmann et al., 2022	44	-1	1.74	20	-.7	2.39	-0.30 [-1.34, 0.74]	2.09
Numao et al., 2013	11	-.4	.56	11	-.1	.67	-0.30 [-0.82, 0.22]	2.54
Wachsmuth et al., 2022	18	-1.7	1.4	18	-1.4	1.4	-0.30 [-1.21, 0.61]	2.21
Luong et al., 2024	11	37.2	8.4	11	37.5	7.9	-0.30 [-7.11, 6.51]	0.19
Kikuchi 2023	21	-2.8	1.19	21	-2.6	1.78	-0.20 [-1.12, 0.72]	2.21
Gardner et al., 2018	304	-2.15	.2	305	-1.97	.21	-0.18 [-0.21, -0.15]	2.74
Krauss et al., 2006	47	-3.6	.2	49	-3.5	.3	-0.10 [-0.20, 0.00]	2.73
Otten et al., 2016	25	-2.7	1.48	16	-2.6	1.79	-0.10 [-1.11, 0.91]	2.12
McManus et al., 2001	25	-3	4	10	-3	5	0.00 [-3.15, 3.15]	0.71
Meksawan et al., 2004	10	20.5	6.96	10	20.5	7.9	0.00 [-6.53, 6.53]	0.21
Bush et al., 2018	12	-.6	1.14	17	-.7	.97	0.10 [-0.67, 0.87]	2.34
Thomson et al., 2010	19	-2.4	1.6	21	-2.5	3.2	0.10 [-1.49, 1.69]	1.58
Bradley et al., 2009	12	-1.6	1.14	12	-1.8	1.18	0.20 [-0.73, 1.13]	2.19
Hyde et al., 2021	12	-.6	1.3	12	-.8	1.45	0.20 [-0.90, 1.30]	2.03
Chiu et al., 2014	32	-.2	1.7	31	-.6	1.67	0.40 [-0.43, 1.23]	2.28
Liu et al., 2013	25	-1.2	.73	24	-1.7	.48	0.50 [0.15, 0.85]	2.65
Ruth et al., 2013	18	-2.5	2.9	15	-3	1.6	0.50 [-1.15, 2.15]	1.54
Veum et al., 2017	20	-6	.87	18	-6.6	1.07	0.60 [-0.02, 1.22]	2.47
Zainordin et al., 2021	14	-.2	.51	16	-1.4	.62	1.20 [0.79, 1.61]	2.61
Meckling et al., 2004	15	-2.5	1.15	16	-3.7	1.62	1.20 [0.20, 2.20]	2.13
**Overall**							-0.78 [-1.09, -0.47]	

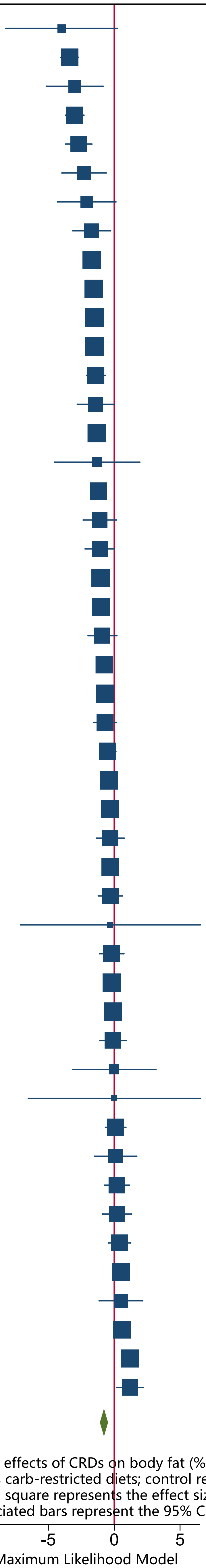

Heterogeneity: $\tau^2 = 0.90$, $I^2 = 98.36\%$, $H^2 = 60.95$

Test of $\theta_i = \theta_j$: $Q(47) = 1445.05$, $p = 0.00$

Test of $\theta = 0$: $z = -4.94$, $p = 0.00$

Forest plot depicting the effects of CRDs on body fat (%)
Note. Treatment involves carb-restricted diets; control refers to higher-carbohydrate diets. For each study, the square represents the effect size (standardized mean difference) and the associated bars represent the 95% CI.

Restricted Randomized Maximum Likelihood Model
The number of studies included: k=48

Random-effects REML model
Sorted by: _meta_es

Study	Treatment N	Treatment Mean	Treatment SD	Control N	Control Mean	Control SD	Mean diff. with 95% CI	Weight (%)
Hyde et al., 2021	12	-1.3	.001	12	1.6	1.73	-2.90 [-3.88, -1.92]	2.66
Gram-Kampmann et al., 2022	44	-2.1	1.33	20	.5	.45	-2.60 [-3.20, -2.00]	3.29
Luong et al., 2024	11	56.5	10.1	11	57.9	10.2	-1.40 [-9.88, 7.08]	0.11
Ruth et al., 2013	18	-1.6	1.3	15	-.3	2.3	-1.30 [-2.55, -0.05]	2.23
Bradley et al., 2009	12	-2.8	1.4	12	-1.7	.1	-1.10 [-1.89, -0.31]	2.97
Otten et al., 2016	25	-1.6	.5	16	-.6	.001	-1.00 [-1.25, -0.75]	3.74
Meckling et al., 2004	15	-1.9	.39	16	-1	.4	-0.90 [-1.18, -0.62]	3.71
Cohen et al., 2018	25	-.9	.5	20	-.1	1.34	-0.80 [-1.37, -0.23]	3.34
Kitabchi et al., 2013	12	-2.7	.69	12	-2	.69	-0.70 [-1.25, -0.15]	3.37
Brinkworth et al., 2004b	21	1	.92	22	1.7	.001	-0.70 [-1.08, -0.32]	3.60
Foster et al., 2010	153	-3.53	.22	154	-3.18	.22	-0.35 [-0.40, -0.30]	3.84
Martins et al., 2025	26	-1.1	.1	24	-.9	.2	-0.20 [-0.29, -0.11]	3.83
Thomson et al., 2010	19	-.8	.9	21	-.6	.8	-0.20 [-0.73, 0.33]	3.40
Goss et al., 2020	19	-1.5	.001	15	-1.3	.3	-0.20 [-0.33, -0.07]	3.81
Brehm et al., 2005	20	-2.08	.001	20	-1.94	.67	-0.14 [-0.43, 0.15]	3.69
Michalczyk et al., 2020a	18	-.58	.66	20	-.44	.43	-0.14 [-0.49, 0.21]	3.63
Liu et al., 2013	25	-2.6	.001	24	-2.5	.49	-0.10 [-0.29, 0.09]	3.78
Veum et al., 2017	20	-1.1	.4	18	-1	.3	-0.10 [-0.33, 0.13]	3.75
McMillan-Price et al., 2006	28	-.4	1.06	30	-.3	1.1	-0.10 [-0.66, 0.46]	3.36
McMillan-Price et al., 2006	31	-.6	1.11	27	-.5	1.04	-0.10 [-0.66, 0.46]	3.36
Walker et al., 1996	16	-.15	.92	16	-.1	.48	-0.05 [-0.56, 0.46]	3.43
Li et al., 2024	22	-.4	.2	23	-.36	.19	-0.04 [-0.15, 0.07]	3.82
He et al., 2022	44	-.5	1.33	44	-.5	1.99	0.00 [-0.71, 0.71]	3.12
Meckling et al., 2007	10	.9	.6	8	.8	.3	0.10 [-0.36, 0.56]	3.50
Noakes et al., 2005	52	-1.5	2.16	48	-1.8	2.08	0.30 [-0.53, 1.13]	2.91
Meksawan et al., 2004	10	52.3	15.18	10	51.9	15.18	0.40 [-12.91, 13.71]	0.05
Wachsmuth et al., 2022	18	.2	1.1	18	-.2	.2	0.40 [-0.12, 0.92]	3.42
Bush et al., 2018	12	.4	.1	17	-.1	.2	0.50 [0.38, 0.62]	3.81
Heggen et al., 2016	64	.4	.3	57	-.2	.2	0.60 [0.51, 0.69]	3.83
Valsdottir et al., 2020	14	-.9	.4	15	-1.8	.1	0.90 [0.69, 1.11]	3.77
Parr et al., 2016	29	.7	1.7	32	-.4	1.7	1.10 [0.25, 1.95]	2.87
**Overall**							-0.30 [-0.59, -0.01]	

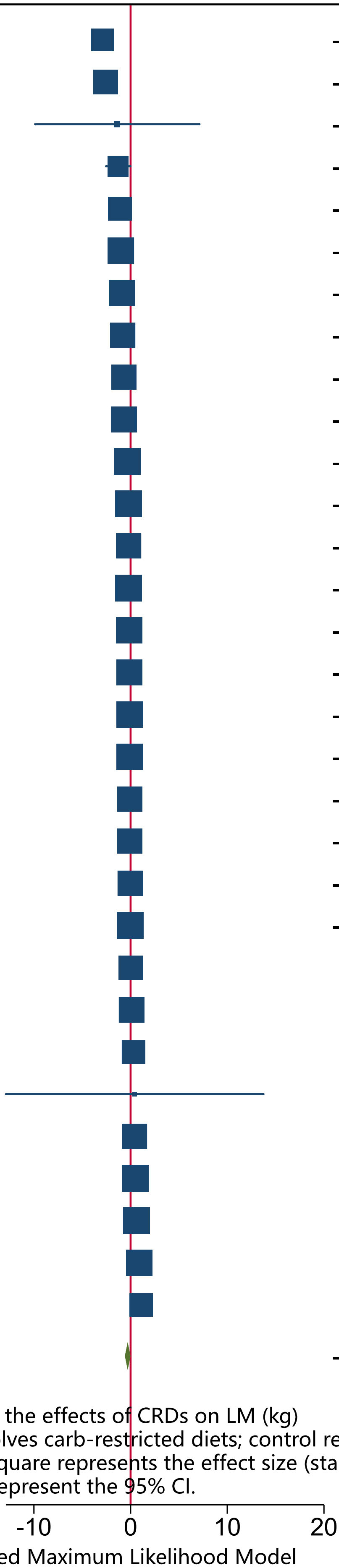

Heterogeneity: $\tau^2 = 0.56$, $I^2 = 98.33\%$, $H^2 = 59.78$

Test of $\theta_i = \theta_j$: Q(30) = 732.07, p = 0.00

Test of $\theta = 0$: z = -2.05, p = 0.04

Forest plot depicting the effects of CRDs on LM (kg)
Note. Treatment involves carb-restricted diets; control refers to higher-carbohydrate diets. For each study, the square represents the effect size (standardized mean difference) and the associated bars represent the 95% CI.

Restricted Randomized Maximum Likelihood Model
The number of studies included: k=31

Random-effects REML model
Sorted by: _meta_es

Study	Treatment N	Treatment Mean	Treatment SD	Control N	Control Mean	Control SD	Mean diff. with 95% CI	Weight (%)
D'Abbondanza et al., 2020	28	74	10	28	77	14	-3.00 [-9.37, 3.37]	0.06
D'Abbondanza et al., 2020	42	56	9	42	59	8	-3.00 [-6.64, 0.64]	0.19
Swenson et al., 2007	19	-9.2	7.6	13	-7	3.1	-2.20 [-6.58, 2.18]	0.13
deLuis et al., 2007	47	-3.1	2.69	43	-1.8	3	-1.30 [-2.48, -0.12]	1.56
Ahmad 2020	25	-.9	1.5	25	.2	1	-1.10 [-1.81, -0.39]	3.34
Brinkworth et al., 2009a	33	-3.2	2.67	36	-2.3	1.2	-0.90 [-1.86, 0.06]	2.15
Wycherley et al., 2014	23	-3.3	1.92	20	-2.4	1.34	-0.90 [-1.90, 0.10]	2.01
Röhling et al., 2020	65	-1	.23	28	-.2	.33	-0.80 [-0.92, -0.68]	8.98
Lundanes et al., 2025	30	-2.9	.61	25	-2.1	.66	-0.80 [-1.14, -0.46]	6.67
Brinkworth et al., 2004a	19	-2.3	2.36	19	-1.6	2.44	-0.70 [-2.23, 0.83]	0.99
Brinkworth et al., 2009b	32	-2.2	2.52	28	-1.6	2.36	-0.60 [-1.84, 0.64]	1.42
Karl et al., 2015	22	-1.2	.38	24	-.6	.36	-0.60 [-0.81, -0.39]	8.07
Genoni et al., 2016	22	-1.38	.38	17	-.79	.36	-0.59 [-0.83, -0.35]	7.84
Martins et al., 2025	26	-1.9	1.02	24	-1.4	1.47	-0.50 [-1.20, 0.20]	3.40
McAuley et al., 2005	31	-2	1.1	30	-1.6	1.31	-0.40 [-1.01, 0.21]	4.02
Tay et al., 2018	33	-2.2	.31	28	-2	.33	-0.20 [-0.36, -0.04]	8.61
Rasmussen et al., 2007	12	.2	1	15	.4	1.4	-0.20 [-1.14, 0.74]	2.22
Frisch et al., 2009	85	-1.5	2.5	80	-1.3	3.6	-0.20 [-1.14, 0.74]	2.23
McAuley et al., 2005	30	-2	1.1	31	-1.8	1.26	-0.20 [-0.79, 0.39]	4.11
Tay et al., 2015	41	-1.8	.28	37	-1.6	.31	-0.20 [-0.33, -0.07]	8.87
Veum et al., 2017	20	-1.8	1.55	18	-1.6	1.51	-0.20 [-1.18, 0.78]	2.11
Karl et al., 2015	21	-1.7	.31	24	-1.6	.31	-0.10 [-0.28, 0.08]	8.41
Claessens et al., 2009	32	.88	2.09	16	.96	1.52	-0.08 [-1.23, 1.07]	1.61
Numao et al., 2013	11	-.4	.8	11	-.4	.73	0.00 [-0.64, 0.64]	3.78
Tay et al., 2014b	46	-1.7	2	47	-1.9	1.7	0.20 [-0.55, 0.95]	3.06
Thomsen et al., 2022	34	-1.4	1.6	33	-1.8	1.4	0.40 [-0.32, 1.12]	3.25
Martens et al., 2015	15	.5	2.36	17	-.6	2.26	1.10 [-0.50, 2.70]	0.91
**Overall**							-0.41 [-0.57, -0.25]	

Heterogeneity: $\tau^2 = 0.07$, $I^2 = 70.72\%$, $H^2 = 3.42$

Test of $\theta_i = \theta_j$: Q(26) = 105.08, p = 0.00

Test of $\theta = 0$: z = -5.01, p = 0.00

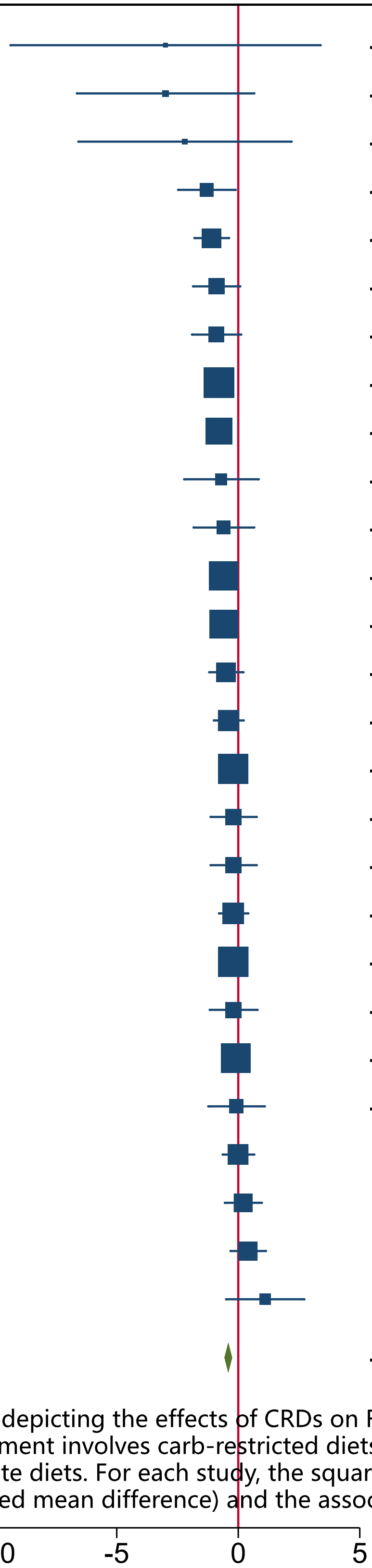

Forest plot depicting the effects of CRDs on FFM (kg)
Note. Treatment involves carb-restricted diets; control refers to higher-carbohydrate diets. For each study, the square represents the effect size (standardized mean difference) and the associated bars represent the 95% CI.

Restricted Randomized Maximum Likelihood Model
The number of studies included: k=27

Random-effects REML model
Sorted by: _meta_es

Study	Treatment N	Treatment Mean	Treatment SD	Control N	Control Mean	Control SD	Mean diff. with 95% CI	Weight (%)
Michalczyk et al., 2020b	46	-13.7	3.17	45	.74	2.08	-14.44 [-15.54, -13.34]	1.33
D'Abbondanza et al., 2020	28	128	12	28	138	12	-10.00 [-16.29, -3.71]	0.58
Goday et al., 2016	45	-14	1.9	40	-5.4	2	-8.60 [-9.43, -7.77]	1.35
Li et al., 2022	24	-9.29	3.62	29	-.77	3.16	-8.52 [-10.35, -6.69]	1.24
D'Abbondanza et al., 2020	42	121	18	42	129	19	-8.00 [-15.92, -0.08]	0.43
Gram-Kampmann et al., 2022	44	-7.7	3.2	20	-.8	3.12	-6.90 [-8.58, -5.22]	1.26
Cunha et al., 2020	20	-8	3.96	19	-3.05	2.91	-4.95 [-7.14, -2.76]	1.18
Abete et al., 2009	19	-11.3	1.33	19	-6.4	1.86	-4.90 [-5.93, -3.87]	1.34
Sun et al., 2019	15	-4.02	2.36	15	.8	1.47	-4.82 [-6.23, -3.41]	1.30
Cai et al., 2021	22	-5.25	2.44	22	-.45	1.78	-4.80 [-6.06, -3.54]	1.31
Dorans et al., 2022	75	-5.2	.87	75	-.5	.54	-4.70 [-4.93, -4.47]	1.39
Wal et al., 2007	42	-4.24	2.49	44	.15	1.7	-4.39 [-5.29, -3.49]	1.35
Guevara-Cruz 2024	11	-5.3	2.77	11	-1	1.88	-4.30 [-6.28, -2.32]	1.22
McManus et al., 2001	25	-9	7.5	10	-4.8	11.3	-4.20 [-10.58, 2.18]	0.57
Jönsson et al., 2009	13	94	9	13	98	11	-4.00 [-11.73, 3.73]	0.44
Chen et al., 2020	43	-5.69	1.39	42	-1.86	.76	-3.83 [-4.31, -3.35]	1.38
Sun et al., 2023	76	-5.5	.38	74	-1.7	.38	-3.80 [-3.92, -3.68]	1.39
Sun et al., 2023	76	-6.9	.38	72	-3.3	.38	-3.60 [-3.72, -3.48]	1.39
McAuley et al., 2005	30	-8.3	1.97	31	-4.8	2.36	-3.50 [-4.59, -2.41]	1.33
Wekesa et al., 2016	24	82.3	2.94	24	85.8	2.45	-3.50 [-5.03, -1.97]	1.28
Ahmad 2020	25	-2	4	25	1.4	5	-3.40 [-5.91, -0.89]	1.13
Dellis et al., 2020	35	-8.69	.48	35	-5.48	.5	-3.21 [-3.44, -2.98]	1.39
Chen et al., 2022	36	-5.3	3.59	35	-2.2	1.65	-3.10 [-4.41, -1.79]	1.31
Lambadiari et al., 2024	16	115.56	15.52	16	118.53	15.71	-2.97 [-13.79, 7.85]	0.27
Hansen et al., 2023	110	-5.1	.71	55	-2.3	.94	-2.80 [-3.06, -2.54]	1.38
Mousavi et al., 2023	35	-5.34	1.72	35	-2.75	1.77	-2.59 [-3.41, -1.77]	1.36
Röhling et al., 2020	65	-4.8	.69	28	-2.3	1.02	-2.50 [-2.86, -2.14]	1.38
Retterstøl et al., 2018	15	-.5	1	15	1.9	1.46	-2.40 [-3.30, -1.50]	1.35
Turton et al., 2023	16	100.9	15.3	16	103.3	15.8	-2.40 [-13.18, 8.38]	0.27
Claessens et al., 2009	32	-1.97	3.9	16	.41	3.68	-2.38 [-4.68, -0.08]	1.17
Hyde et al., 2021	12	-1.9	1.6	12	.4	1.66	-2.30 [-3.60, -1.00]	1.31
Frisch et al., 2009	85	-6.9	6.1	80	-4.7	8.9	-2.20 [-4.52, 0.12]	1.16
Wolever et al., 2008	53	4.5	16.17	55	6.6	11.3	-2.10 [-7.35, 3.15]	0.70
Buscemi et al., 2009	10	-8	4.24	10	-6	1.26	-2.00 [-4.74, 0.74]	1.09
Chiu et al., 2014	32	-2	5.66	31	0	5.57	-2.00 [-4.77, 0.77]	1.09
McMillan-Price et al., 2006	31	-6.3	3.34	27	-4.3	3.64	-2.00 [-3.80, -0.20]	1.24
Zainordin et al., 2021	14	-4	3.89	16	-2	.92	-2.00 [-3.96, -0.04]	1.22
YancyWSJr et al., 2010	57	-11.07	1.42	65	-9.08	.97	-1.99 [-2.42, -1.56]	1.38
Genoni et al., 2016	22	-3.35	.43	17	-1.55	.56	-1.80 [-2.11, -1.49]	1.38
Bazzano et al., 2014	59	-6.7	.92	60	-5	.92	-1.70 [-2.03, -1.37]	1.38
Hu et al., 2013	75	-6.7	.92	73	-5	.92	-1.70 [-2.00, -1.40]	1.38
Kikuchi 2023	21	-8.5	2.81	21	-6.8	3.41	-1.70 [-3.59, 0.19]	1.23
Haji-GhaziTehrani et al., 2022	24	-4.8	4.51	24	-3.3	2.99	-1.50 [-3.66, 0.66]	1.19
Tay et al., 2014b	46	-10.6	7.1	47	-9.1	6.4	-1.50 [-4.25, 1.25]	1.09
Zinn et al., 2017	14	-4.8	2.73	12	-3.3	2.09	-1.50 [-3.39, 0.39]	1.23
Elhayany et al., 2010	61	-10.4	2.05	55	-9.1	2.05	-1.30 [-2.05, -0.55]	1.36
Golay et al., 1996	22	-11	2.81	21	-10	2.75	-1.00 [-2.66, 0.66]	1.26
Liu et al., 2013	25	-7.8	1.95	24	-6.8	1.53	-1.00 [-1.98, -0.02]	1.34
deLuis et al., 2007	47	-4.2	2.99	43	-3.4	2.31	-0.80 [-1.91, 0.31]	1.33
McCullough et al., 2022	8	-2.6	3.45	8	-1.8	4.46	-0.80 [-4.71, 3.11]	0.90
Lean et al., 1997	40	-6.5	.82	41	-5.7	.77	-0.80 [-1.15, -0.45]	1.38
Tay et al., 2015	41	-9.8	1.07	37	-9.1	1.07	-0.70 [-1.18, -0.22]	1.38
Pinsawas et al., 2024	26	-4.2	2.42	22	-3.5	2.38	-0.70 [-2.06, 0.66]	1.30
Tay et al., 2018	33	-7.9	1.1	28	-7.2	1.15	-0.70 [-1.27, -0.13]	1.37
Skytte et al., 2019	14	-1	1.87	14	-.3	2.24	-0.70 [-2.23, 0.83]	1.28
Jenkins et al., 2014	20	-6.1	.41	19	-5.4	.59	-0.70 [-1.02, -0.38]	1.38
Gardner et al., 2018	304	-4.41	.46	305	-3.74	.46	-0.67 [-0.74, -0.60]	1.39
Haufe et al., 2011	52	-6.2	4.33	50	-5.6	4.95	-0.60 [-2.40, 1.20]	1.24
McAuley et al., 2005	31	-8.3	1.97	30	-7.7	2.83	-0.60 [-1.82, 0.62]	1.32
Heggen et al., 2016	64	-2.9	4.2	57	-2.4	9.2	-0.50 [-3.00, 2.00]	1.13
Li et al., 2024	22	-4.37	.8	23	-4.02	.77	-0.35 [-0.81, 0.11]	1.38
Parr et al., 2016	29	-5.5	3.3	32	-5.2	3	-0.30 [-1.88, 1.28]	1.27
Guldbrand et al., 2012	30	-2	3.26	31	-2	4.16	0.00 [-1.88, 1.88]	1.23
Otten et al., 2016	25	-12	2.51	16	-12	1.8	0.00 [-1.42, 1.42]	1.29
Rodriguez-Villar et al., 2004	22	98	8	22	98	8	0.00 [-4.73, 4.73]	0.77
Krebs et al., 2012	152	-3	2.8	158	-3.1	2.77	0.10 [-0.52, 0.72]	1.37
Harvey et al., 2019	14	-2.81	.54	12	-2.95	1.34	0.14 [-0.62, 0.90]	1.36
Boers et al., 2014	18	-3.1	2	16	-3.3	2.4	0.20 [-1.28, 1.68]	1.29
deLuis et al., 2015	49	-5.3	5.54	45	-5.6	2.95	0.30 [-1.52, 2.12]	1.24
Walker et al., 1996	16	-1.4	.28	16	-1.7	.36	0.30 [0.08, 0.52]	1.39
Archer et al 2003	32	-2.2	3.1	31	-2.6	2.81	0.40 [-1.06, 1.86]	1.29
Oliveira et al., 2023	60	-2.3	.97	61	-2.7	.97	0.40 [0.05, 0.75]	1.38
Bradley et al., 2009	12	-4.6	2.26	12	-5.1	1.82	0.50 [-1.14, 2.14]	1.27
Wolever et al., 2008	53	4.5	16.17	48	4	15.39	0.50 [-5.67, 6.67]	0.59
McMillan-Price et al., 2006	28	-5	3.7	30	-5.6	3.83	0.60 [-1.34, 2.54]	1.22
Thomson et al., 2010	19	-5.3	4.4	21	-6.1	7.4	0.80 [-3.03, 4.63]	0.91
Wan et al., 2017	101	-.3	.08	101	-1.1	.1	0.80 [0.78, 0.82]	1.39
He et al., 2022	44	-3.3	7.96	44	-4.2	6.63	0.90 [-2.16, 3.96]	1.04
deLuis et al., 2015	61	-7.7	2.11	56	-8.7	3.1	1.00 [0.05, 1.95]	1.34
Thomsen et al., 2022	34	-32	23	33	-33	26	1.00 [-10.75, 12.75]	0.23
Bush et al., 2018	12	.2	2.53	17	-1.8	1.84	2.00 [0.41, 3.59]	1.27
Veum et al., 2017	20	-11	1.65	18	-13	2.08	2.00 [0.81, 3.19]	1.32
Haufe et al., 2012	41	-6	3.12	49	-9	2.51	3.00 [1.84, 4.16]	1.32
de Luis et al., 2015	168	-6.2	7.13	163	-11.6	2.16	5.40 [4.26, 6.54]	1.33
**Overall**							-1.86 [-2.48, -1.24]	

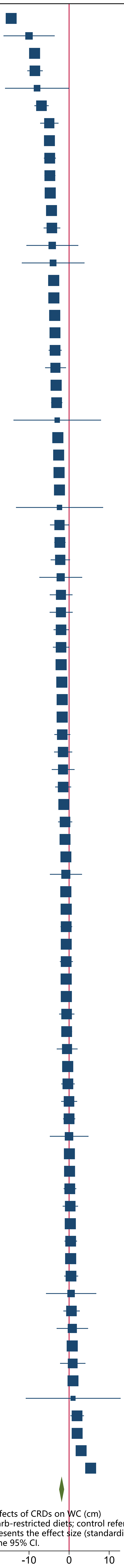

Heterogeneity: $\tau^2 = 7.29$, $I^2 = 99.66\%$, $H^2 = 298.07$

Test of $\theta_i = \theta_j$: Q(83) = 16811.39, p = 0.00

Test of $\theta = 0$: z = -5.85, p = 0.00

Random-effects REML model
Sorted by: _meta_es

Forest plot depicting the effects of CRDs on WC (cm)
Note. Treatment involves carb-restricted diets; control refers to higher-carbohydrate diets. For each study, the square represents the effect size (standardized mean difference) and the associated bars represent the 95% CI.

Restricted Randomized Maximum Likelihood Model
The number of studies included: k=84

Study	Treatment N	Treatment Mean	Treatment SD	Control N	Control Mean	Control SD	Mean diff. with 95% CI	Weight (%)
Buscemi et al., 2009	10	-8	1.79	10	-3	.89	-5.00 [-6.24, -3.76]	5.73
Zainordin et al., 2021	14	-5	1.85	16	0	1.53	-5.00 [-6.21, -3.79]	5.77
Wal et al., 2007	42	-3.43	2.24	44	.08	1.27	-3.51 [-4.28, -2.74]	6.20
Chen et al., 2020	43	-6.05	.95	42	-2.89	.86	-3.16 [-3.55, -2.77]	6.44
Chen et al., 2022	36	-6	2.98	35	-3.6	1.45	-2.40 [-3.50, -1.30]	5.89
Sun et al., 2019	15	-2.51	1.93	15	-.13	1.52	-2.38 [-3.62, -1.14]	5.73
Gram-Kampmann et al., 2022	44	-3.5	1.77	20	-1.2	8.58	-2.30 [-4.93, 0.33]	4.02
Harvey et al., 2019	14	-5.4	1.5	12	-3.56	.73	-1.84 [-2.77, -0.91]	6.05
Claessens et al., 2009	32	-.26	4.36	16	1.36	3.352	-1.62 [-4.06, 0.82]	4.25
Mousavi et al., 2023	35	-2.58	1.66	35	-1.11	1.66	-1.47 [-2.25, -0.69]	6.19
He et al., 2022	44	-2.8	5.97	44	-1.5	4.64	-1.30 [-3.53, 0.93]	4.50
Cai et al., 2021	22	-1.7	2.39	22	-1.18	1.69	-0.52 [-1.74, 0.70]	5.75
Lean et al., 1997	40	-5.7	.66	41	-5.2	.71	-0.50 [-0.80, -0.20]	6.48
Golay et al., 1996	22	-9	5.22	21	-9	1.33	0.00 [-2.30, 2.30]	4.41
Hyde et al., 2021	12	-1.2	1.04	12	-1.3	1.58	0.10 [-0.97, 1.17]	5.92
Li et al., 2024	22	-3.97	.69	23	-4.23	.68	0.26 [-0.14, 0.66]	6.44
Liu et al., 2013	25	-3.1	.71	24	-3.8	.64	0.70 [0.32, 1.08]	6.44
Walker et al., 1996	16	-.9	3.2	16	-2.1	4.8	1.20 [-1.63, 4.03]	3.79
**Overall**							-1.65 [-2.50, -0.79]	

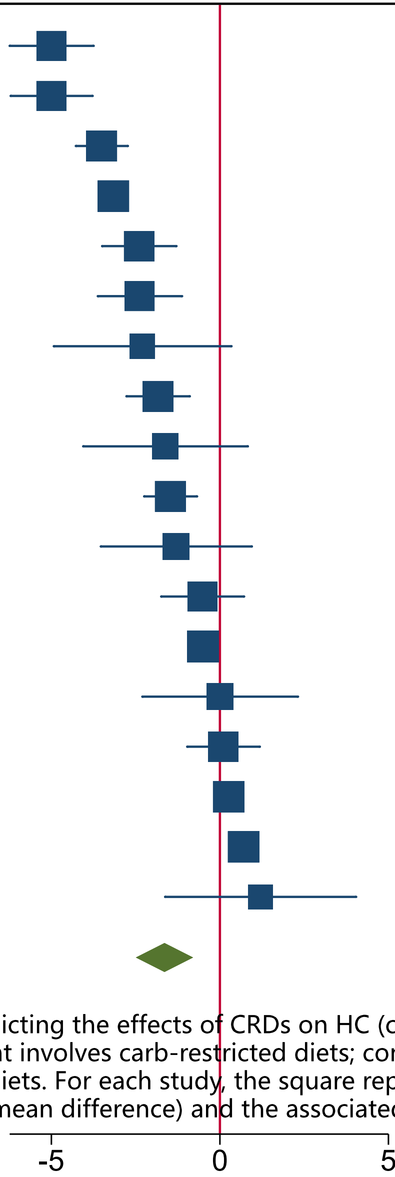

Heterogeneity: $\tau^2 = 2.88$, $I^2 = 95.71\%$, $H^2 = 23.30$

Test of $\theta_i = \theta_j$: $Q(17) = 394.44$, $p = 0.00$

Test of $\theta = 0$: $z = -3.79$, $p = 0.00$

Forest plot depicting the effects of CRDs on HC (cm)
Note. Treatment involves carb-restricted diets; control refers to higher-carbohydrate diets. For each study, the square represents the effect size (standardized mean difference) and the associated bars represent the 95% CI.

Restricted Randomized Maximum Likelihood Model
The number of studies included: k=18

Random-effects REML model
Sorted by: _meta_es

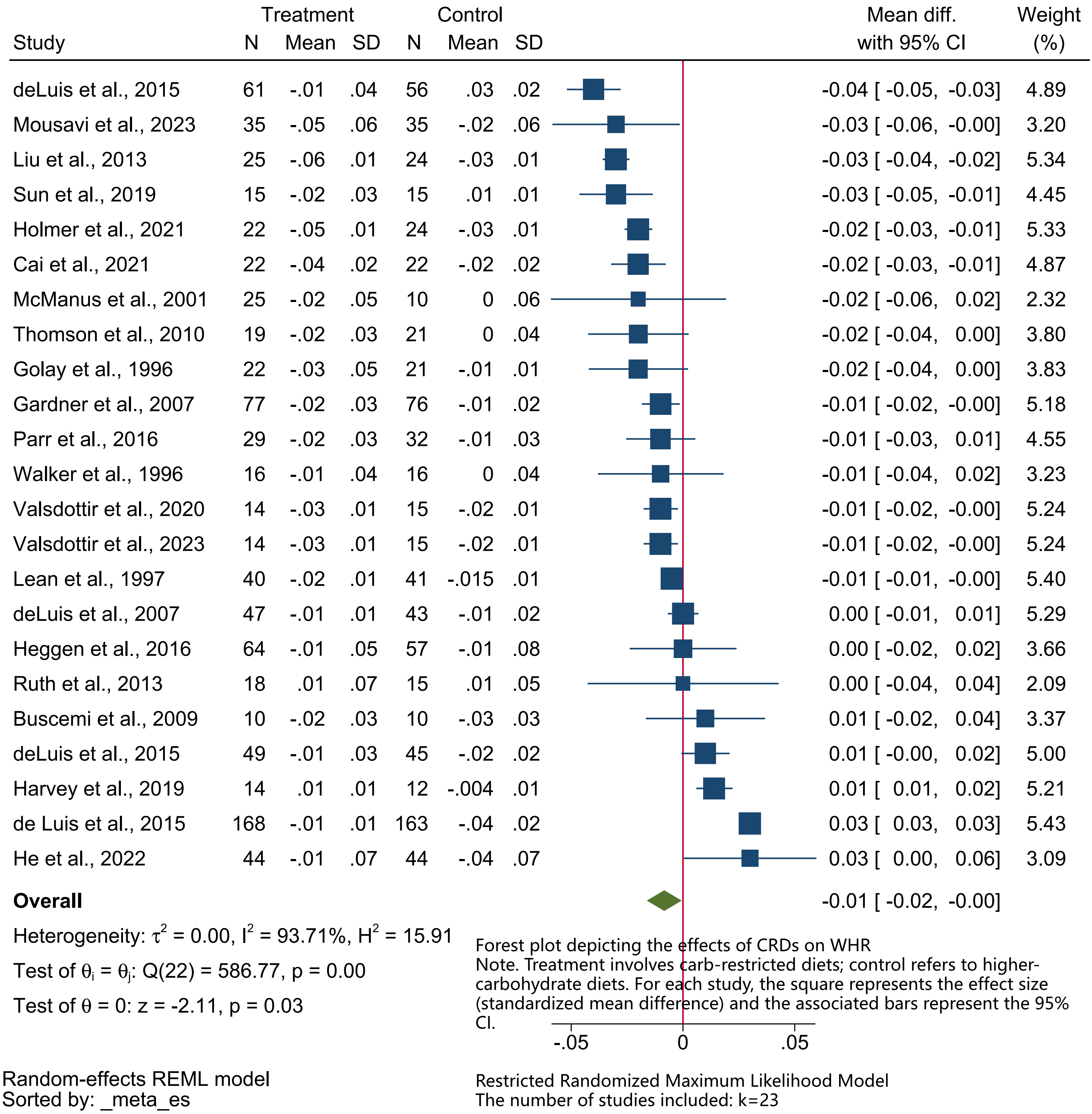

Forest plot depicting the effects of CRDs on WHR
Note. Treatment involves carb-restricted diets; control refers to higher-carbohydrate diets. For each study, the square represents the effect size (standardized mean difference) and the associated bars represent the 95% CI.

Restricted Randomized Maximum Likelihood Model
The number of studies included: k=23

Study	Treatment N	Treatment Mean	Treatment SD	Control N	Control Mean	Control SD	Mean diff. with 95% CI	Weight (%)
McCullough et al., 2022	8	-.96	.03	8	-.17	.13	-0.79 [-0.88, -0.70]	16.65
Goss et al., 2020	19	-.5	.3	15	-.1	.001	-0.40 [-0.55, -0.25]	15.86
Valsdottir et al., 2020	14	-.3	.2	15	-.1	.001	-0.20 [-0.30, -0.10]	16.55
Zainordin et al., 2021	14	-.2	.06	16	-.06	.06	-0.14 [-0.18, -0.10]	17.03
Otten et al., 2016	25	-.37	.09	16	-.28	.01	-0.09 [-0.13, -0.05]	17.02
Cohen et al., 2018	25	-.18	.07	20	-.12	.15	-0.06 [-0.13, 0.01]	16.88
**Overall**							-0.28 [-0.50, -0.05]	

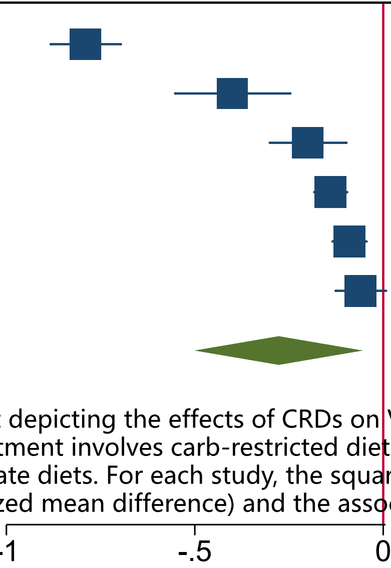

Heterogeneity: $\tau^2 = 0.08$, $I^2 = 98.48\%$, $H^2 = 65.92$

Test of $\theta_i = \theta_j$: Q(5) = 206.73, p = 0.00

Test of $\theta = 0$: z = -2.44, p = 0.01

Random-effects REML model
Sorted by: _meta_es

Forest plot depicting the effects of CRDs on VAT (kg)
Note. Treatment involves carb-restricted diets; control refers to higher-carbohydrate diets. For each study, the square represents the effect size (standardized mean difference) and the associated bars represent the 95% CI.

Restricted Randomized Maximum Likelihood Model
The number of studies included: k=6

Effects of carbohydrate-restricted diets and macronutrient replacements on cardiovascular health and body composition in adults: A meta-analysis of randomized trials; Feng, Shuo

Supplementary Figures - Funnel plots of CRDs on Cardiovascular Health and Body Composition Outcomes

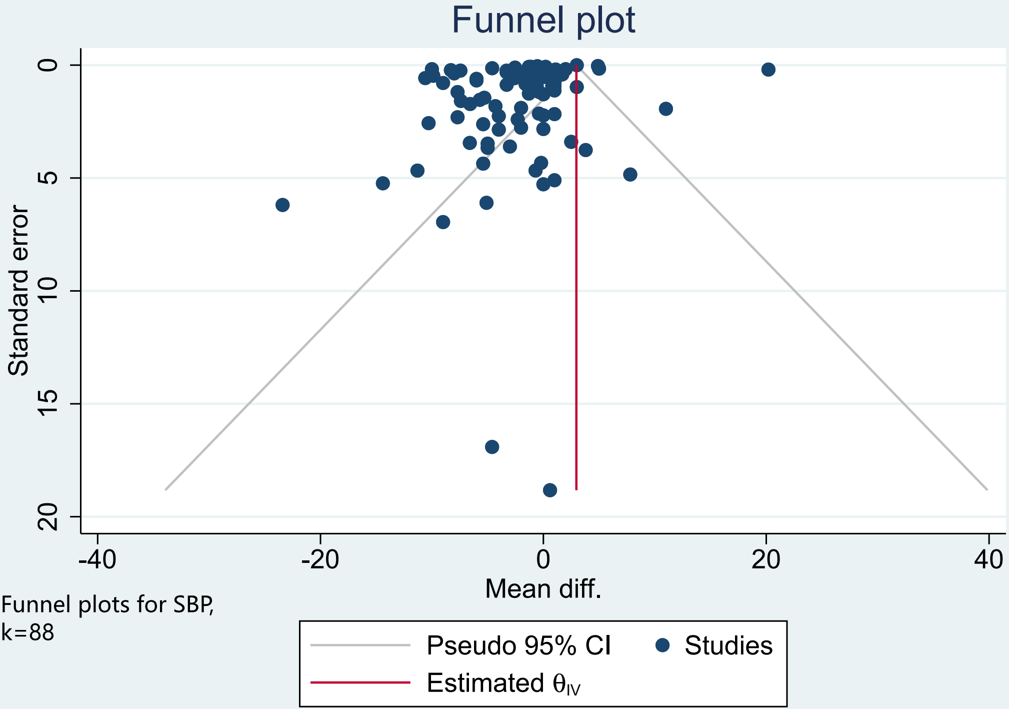

Funnel plots for SBP,
k=88

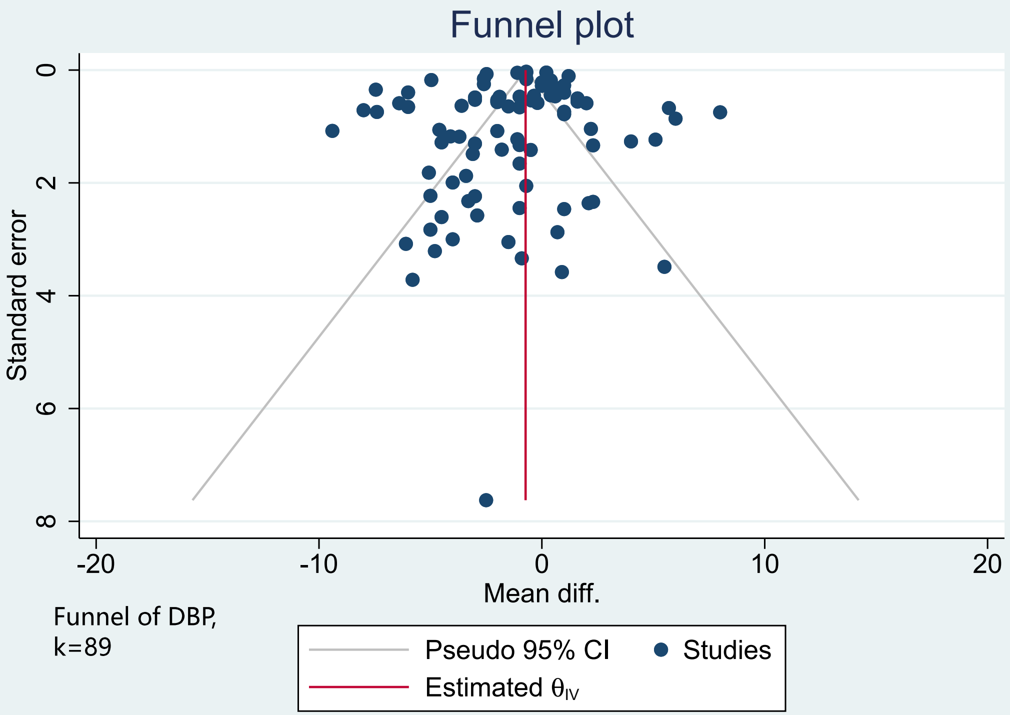

Funnel of DBP,
k=89

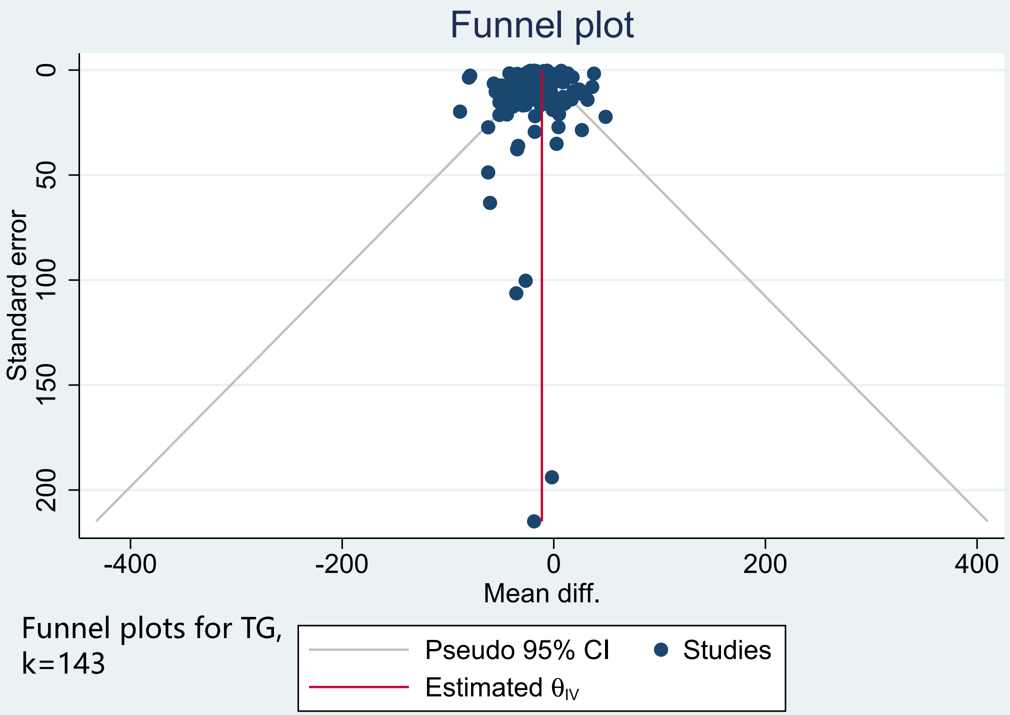

Funnel plots for TG,
k=143

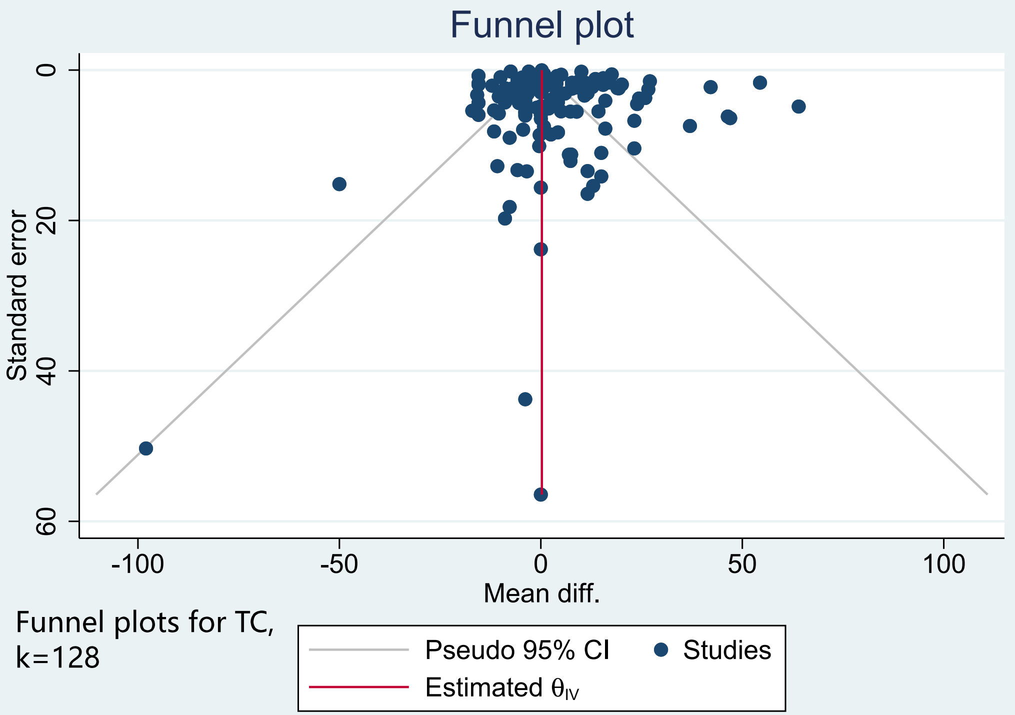

Funnel plots for TC, k=128

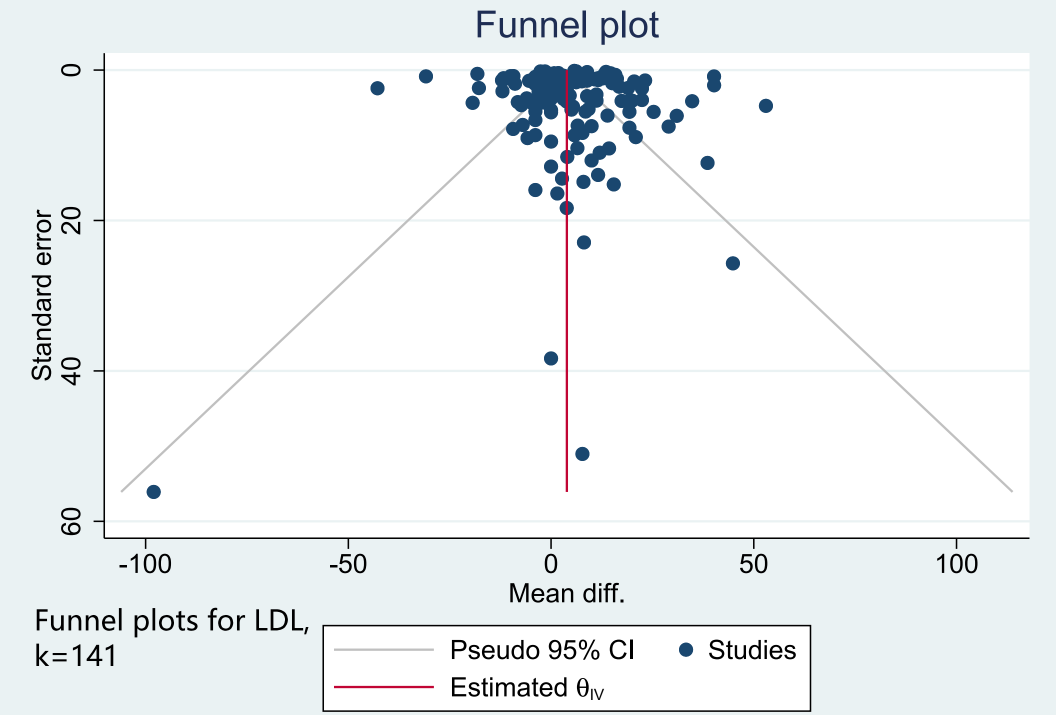

Funnel plots for LDL, k=141

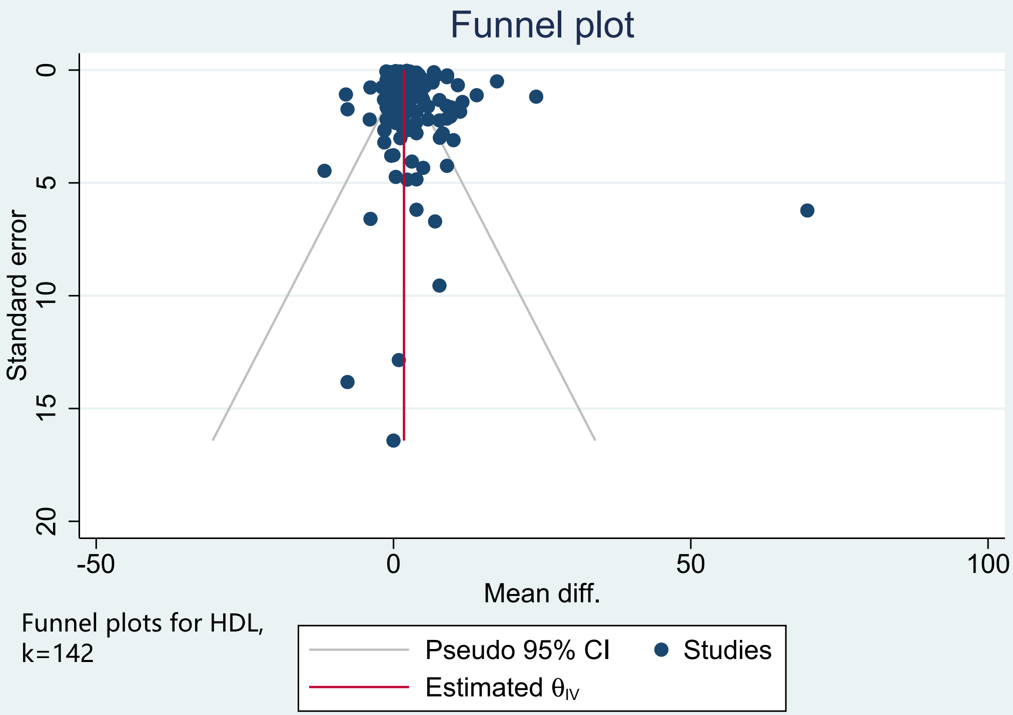

Funnel plots for HDL, k=142

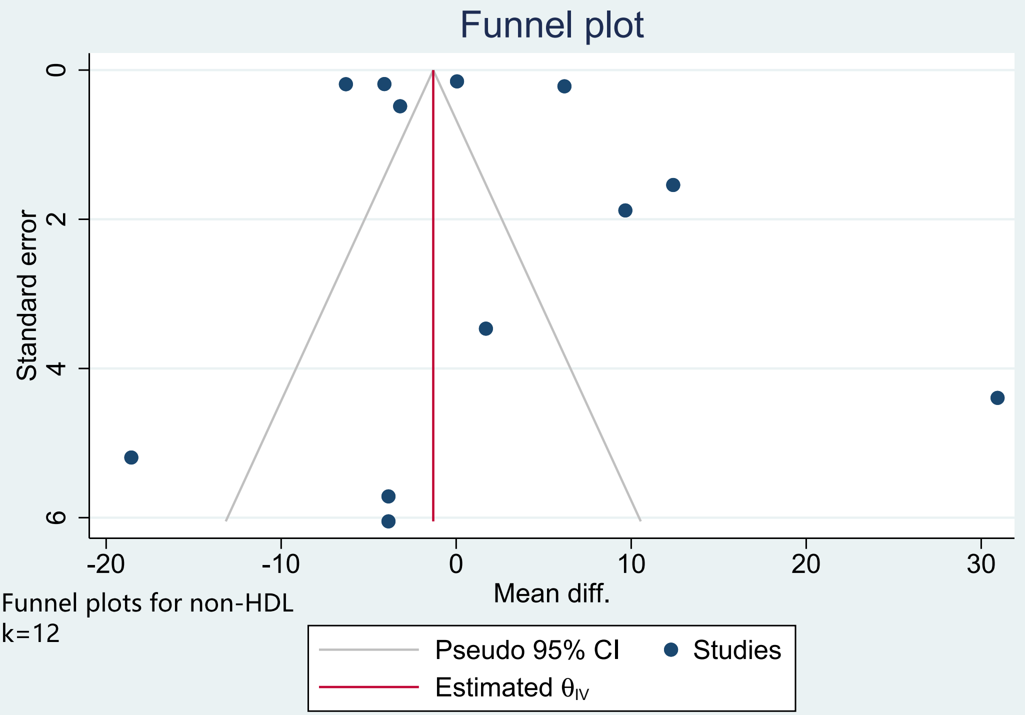

Funnel plots for non-HDL
k=12

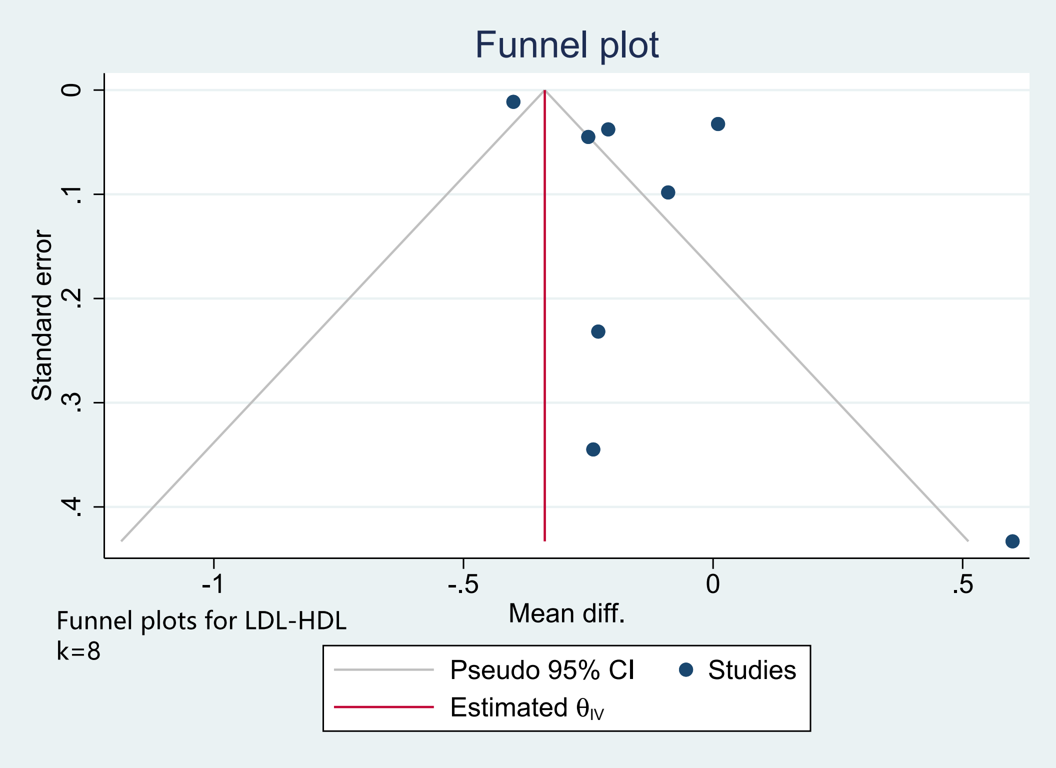
Funnel plot
Standard error
0
.1
.2
.3
.4
-1
-.5
0
.5
Mean diff.
Funnel plots for LDL-HDL
k=8
Pseudo 95% CI
Studies
Estimated θ_{IV}

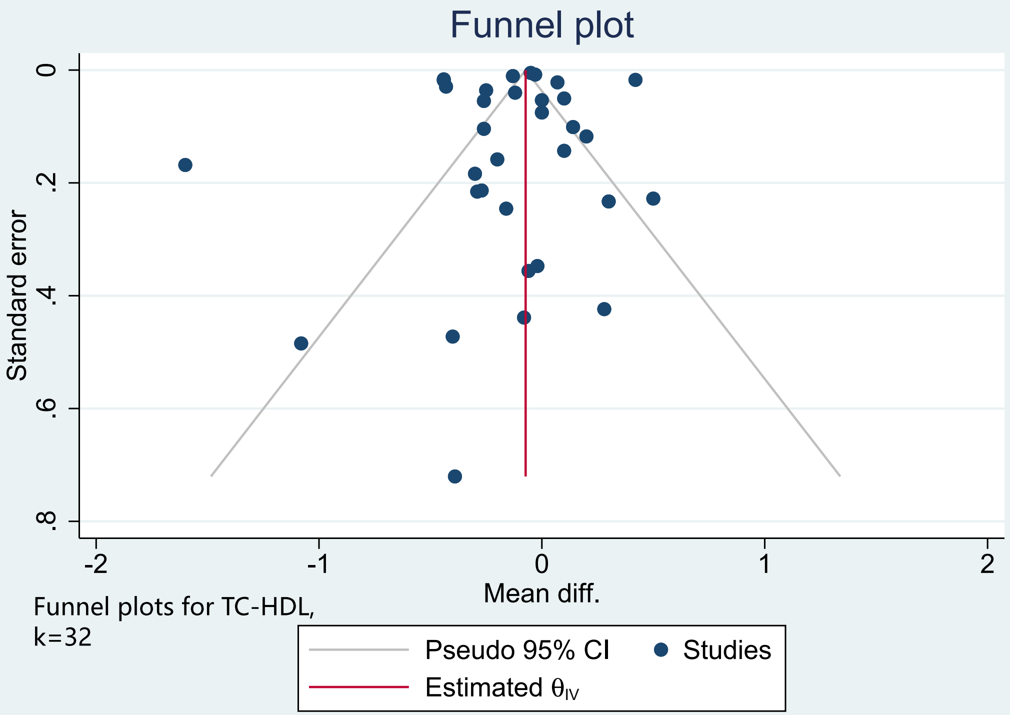

Funnel plots for TC-HDL,
k=32

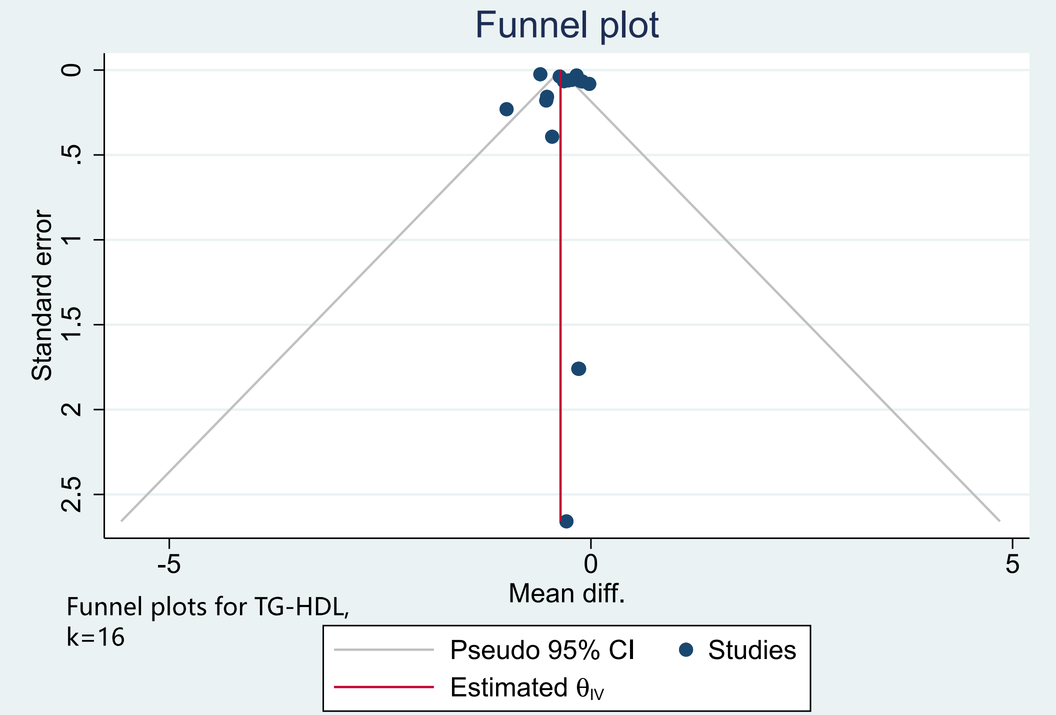

Funnel plots for TG-HDL, k=16

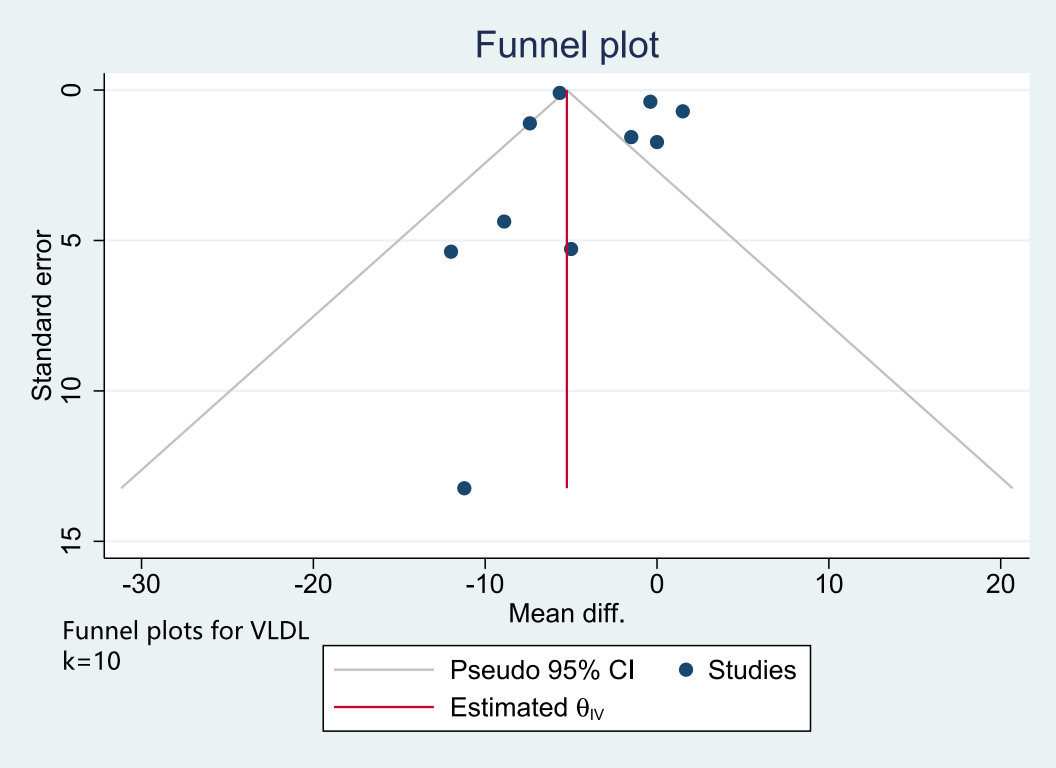

Funnel plots for VLDL
k=10

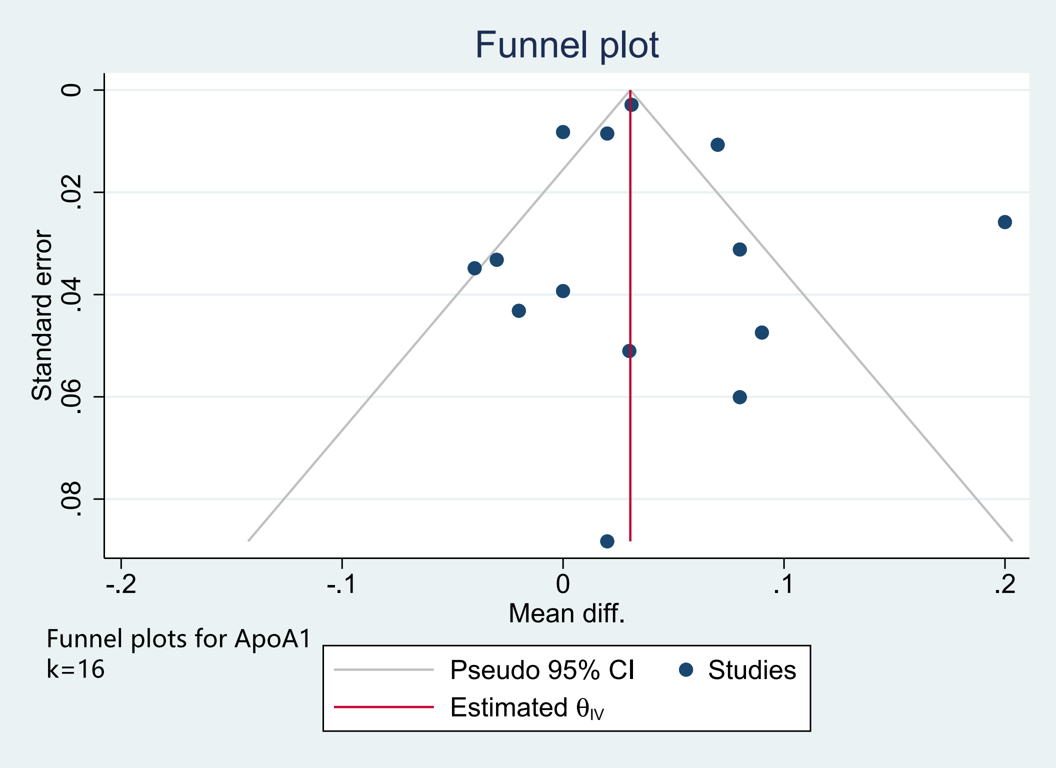

Funnel plots for ApoA1
k=16

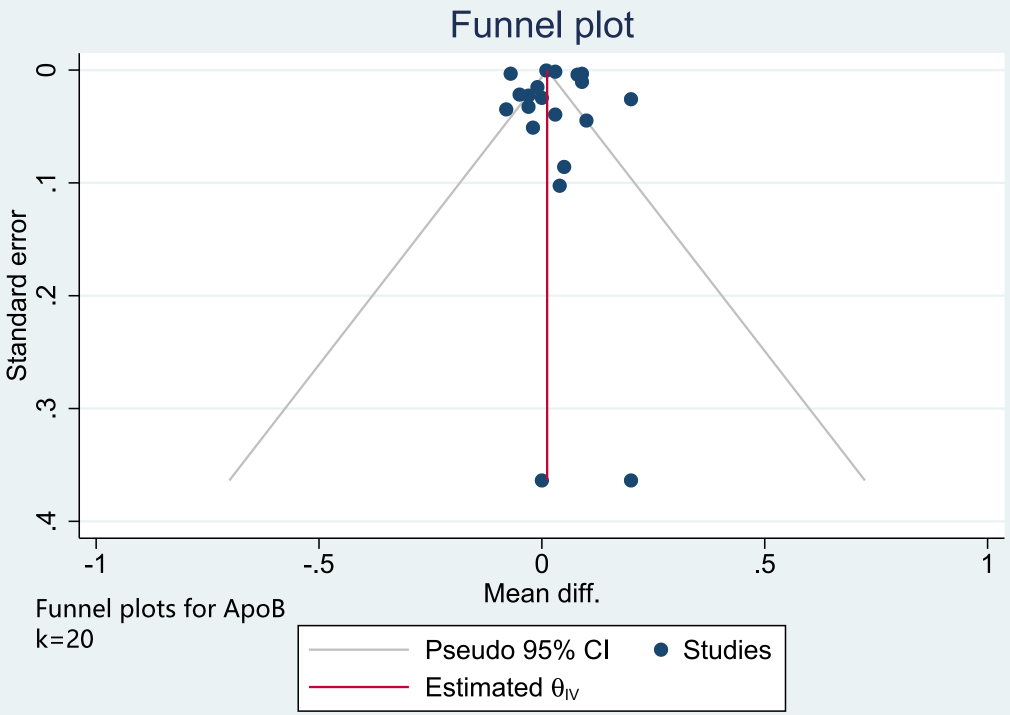

Funnel plots for ApoB
k=20

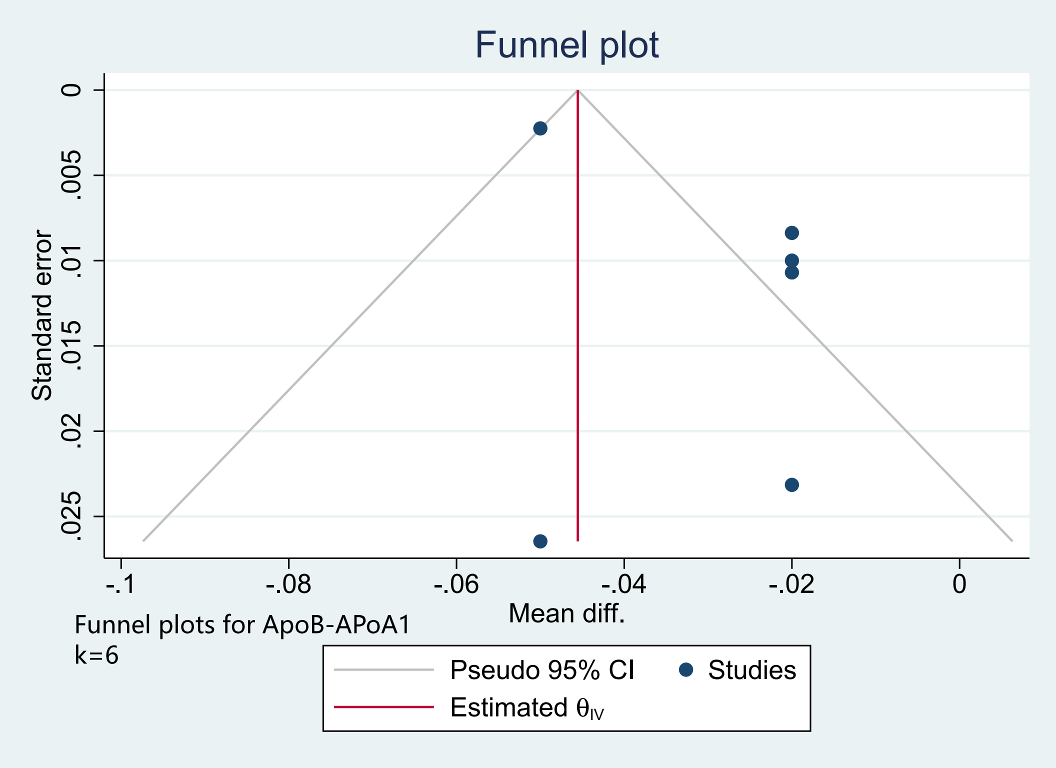

Funnel plot
Standard error
0
.005
.01
.015
.02
.025
-.1
-.08
-.06
-.04
-.02
0
Mean diff.
Funnel plots for ApoB-APoA1
k=6
Pseudo 95% CI
Studies
Estimated θIV

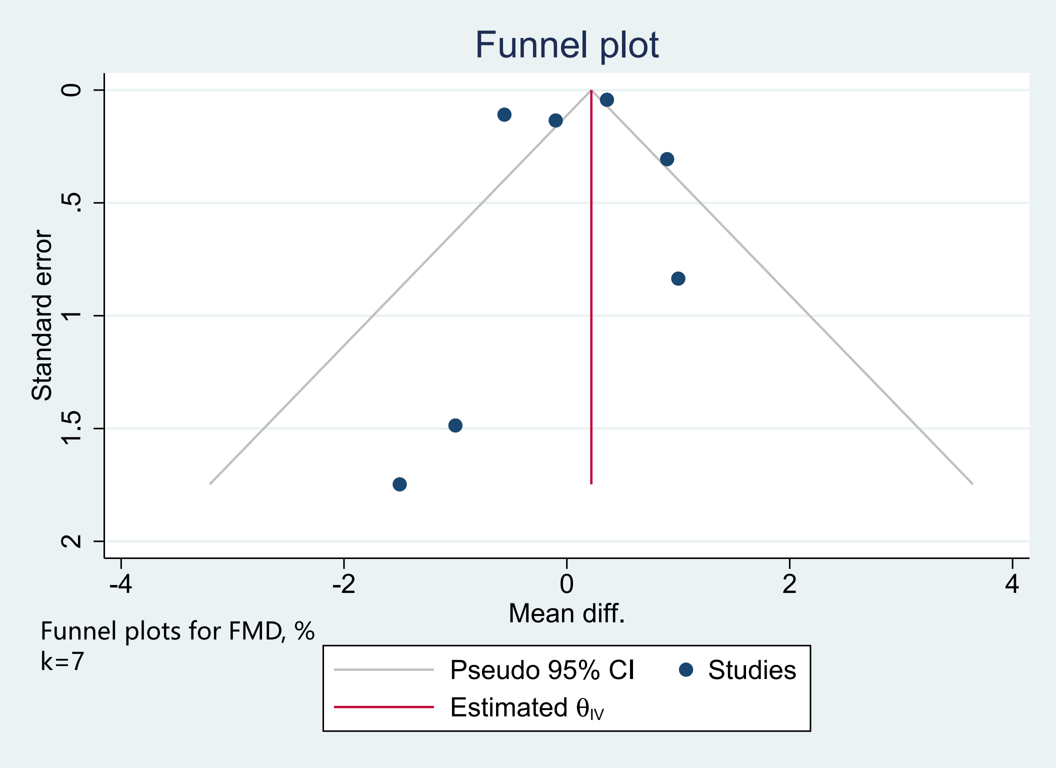

Funnel plot
Standard error
0
.5
1
1.5
2
-4
-2
0
2
4
Mean diff.
Funnel plots for FMD, %
k=7
Pseudo 95% CI
Studies
Estimated θIV

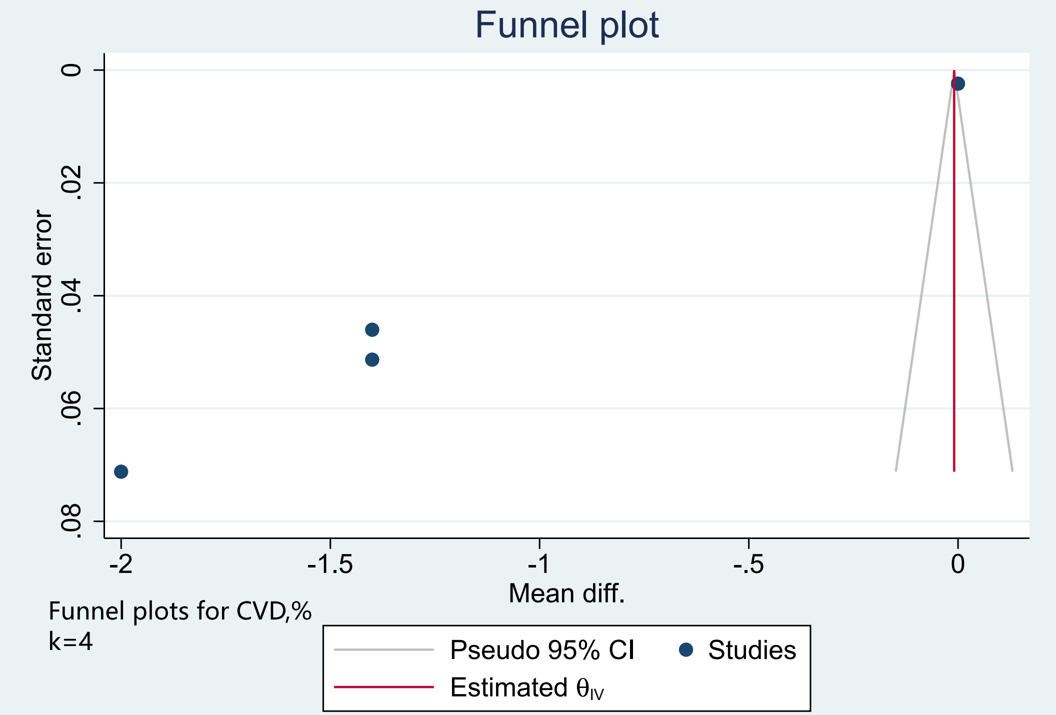

Funnel plots for CVD,%
k=4

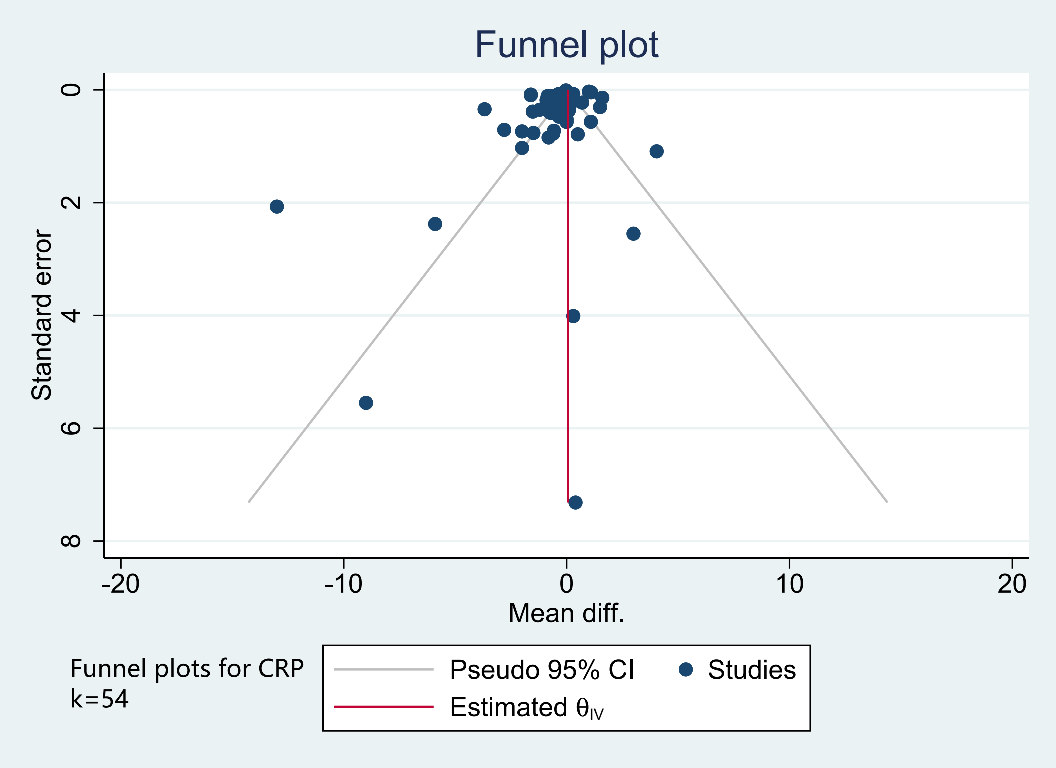
Funnel plot
Standard error
0
2
4
6
8
-20
-10
0
10
20
Mean diff.
Funnel plots for CRP
k=54
Pseudo 95% CI
Studies
Estimated θIV

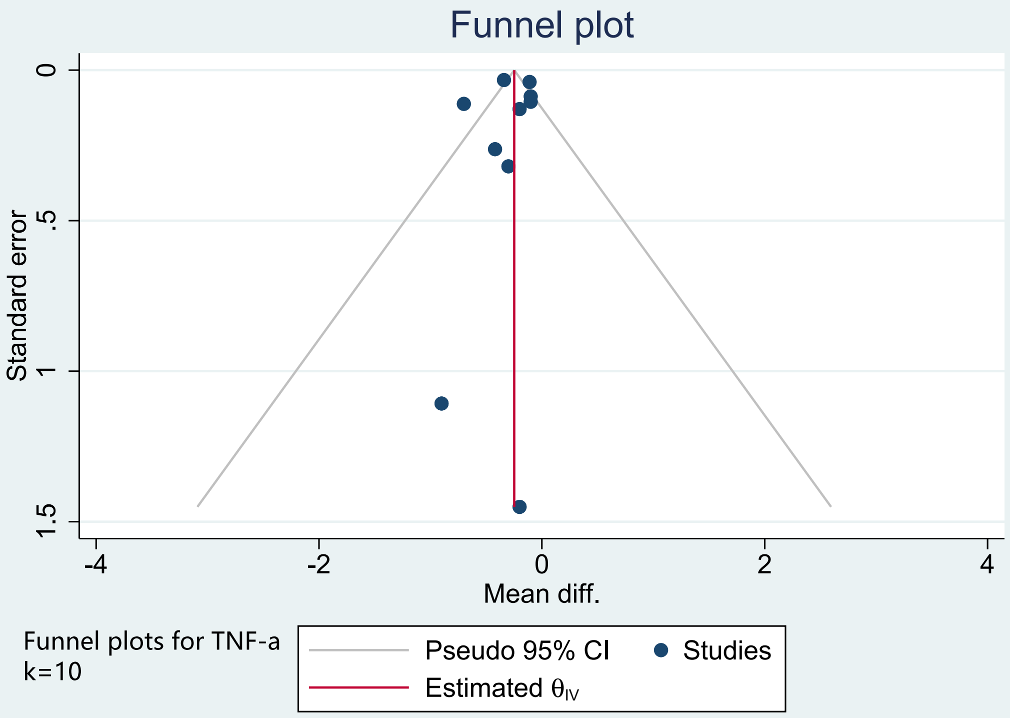

Funnel plots for TNF-a
k=10

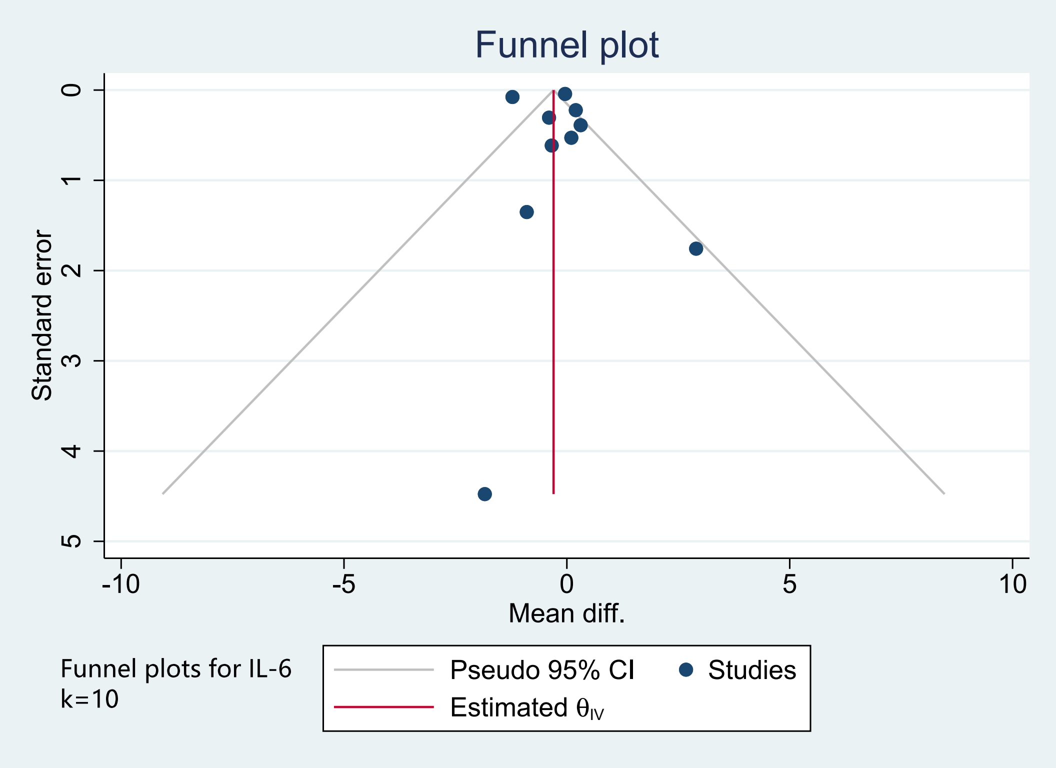

Funnel plot
Standard error
0
1
2
3
4
5
-10
-5
0
5
10
Mean diff.
Funnel plots for IL-6
k=10
Pseudo 95% CI
Studies
Estimated θ_{IV}

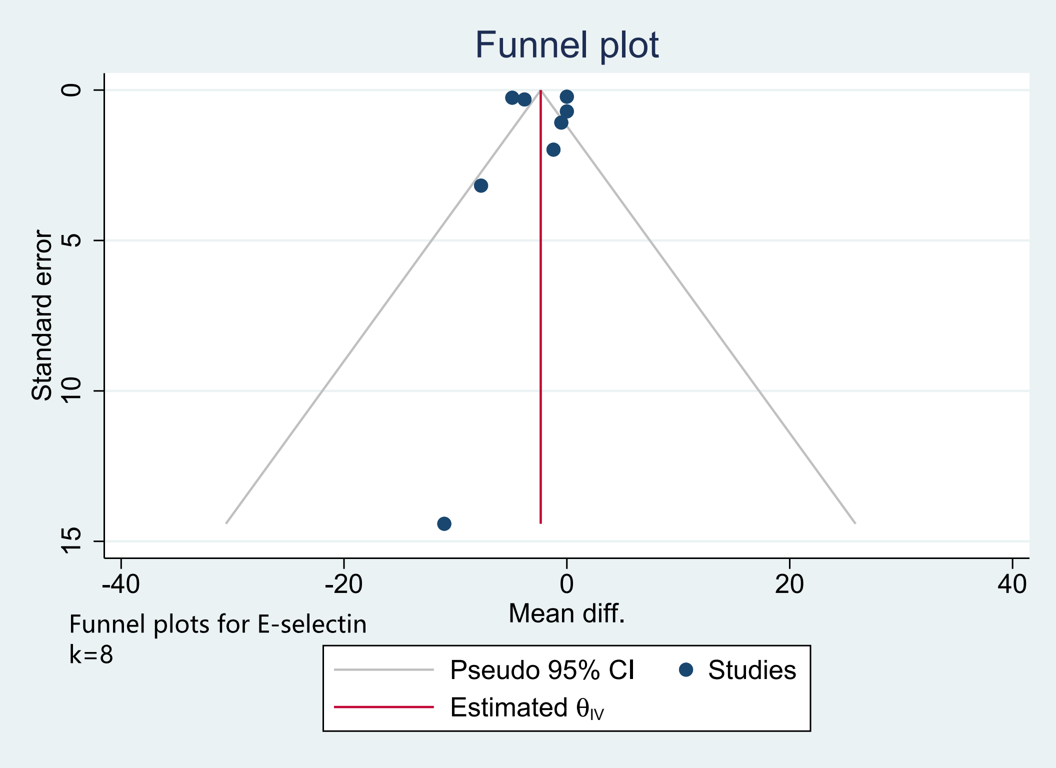
Funnel plot
Standard error
0
5
10
15
-40
-20
0
20
40
Mean diff.
Funnel plots for E-selectin
k=8
Pseudo 95% CI
Studies
Estimated θ_{IV}

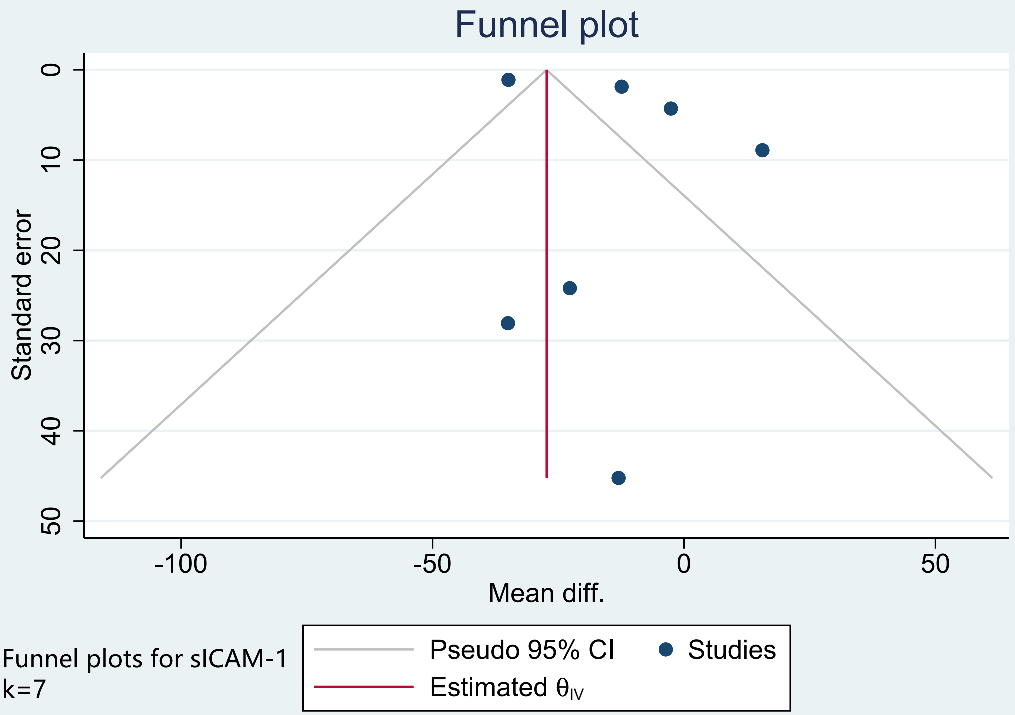

Funnel plots for sICAM-1
k=7

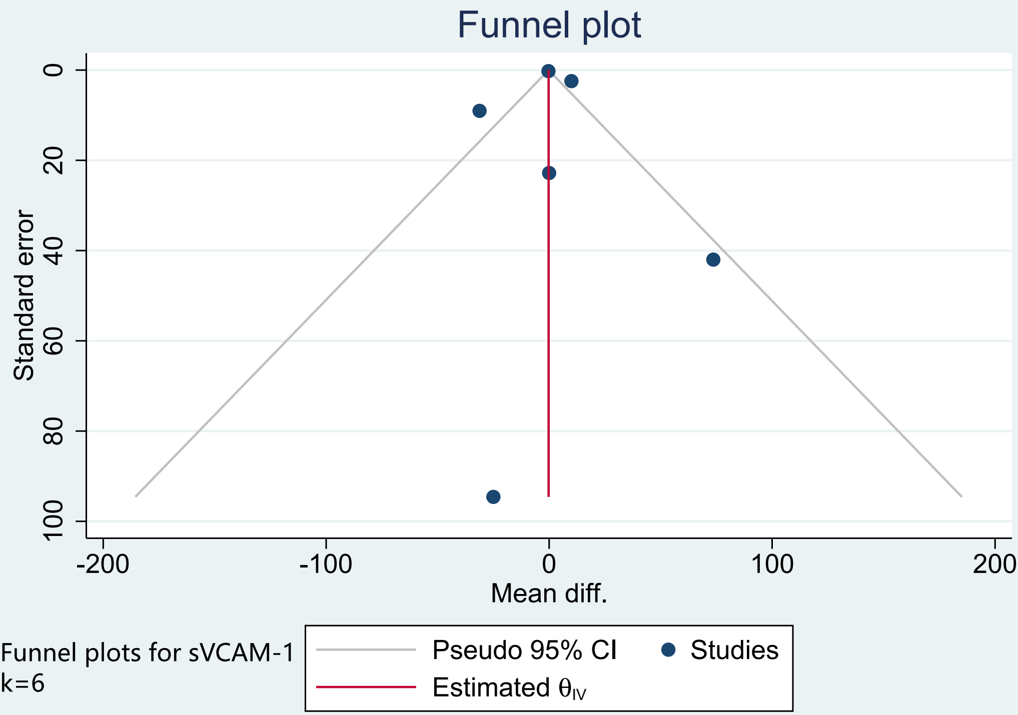

Funnel plots for sVCAM-1
k=6

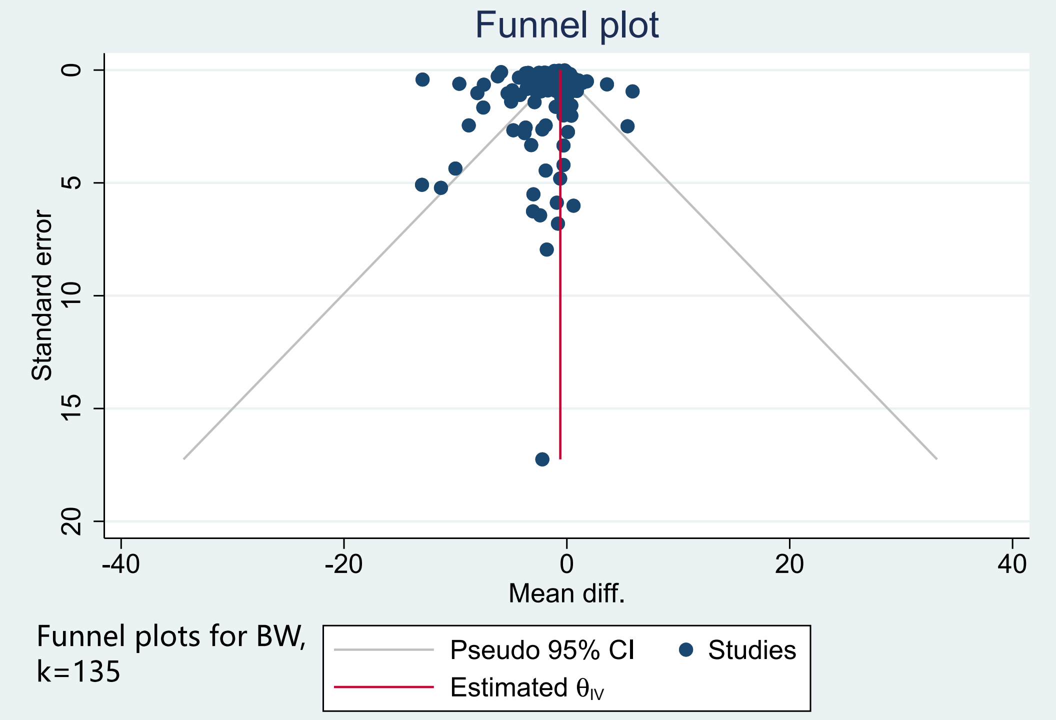

Funnel plots for BW, k=135

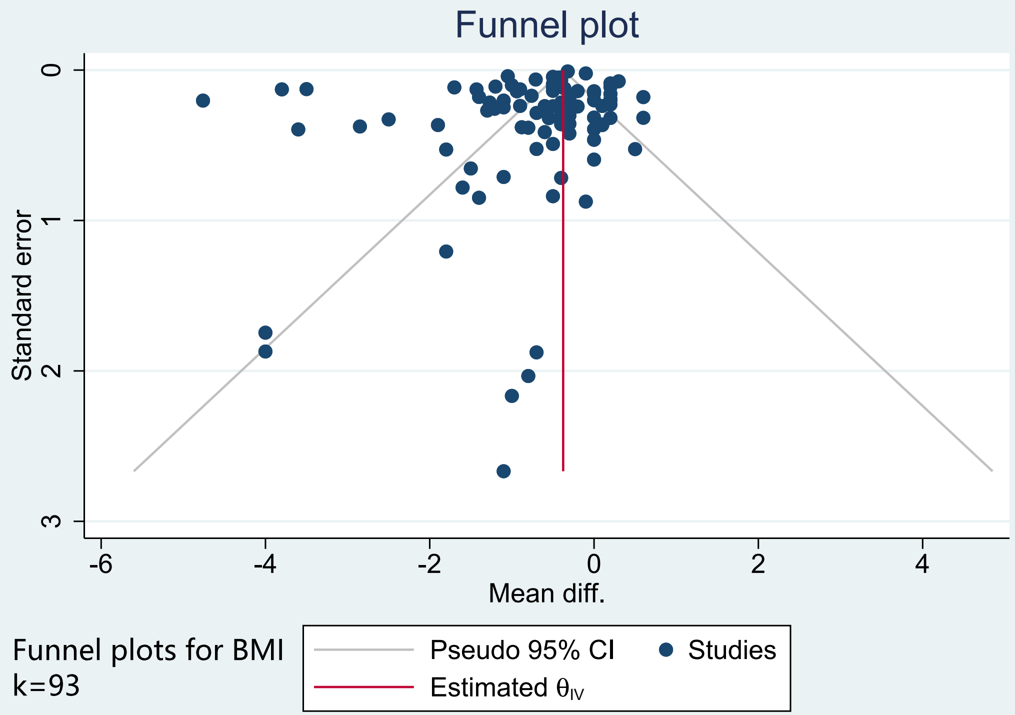

Funnel plots for BMI
k=93

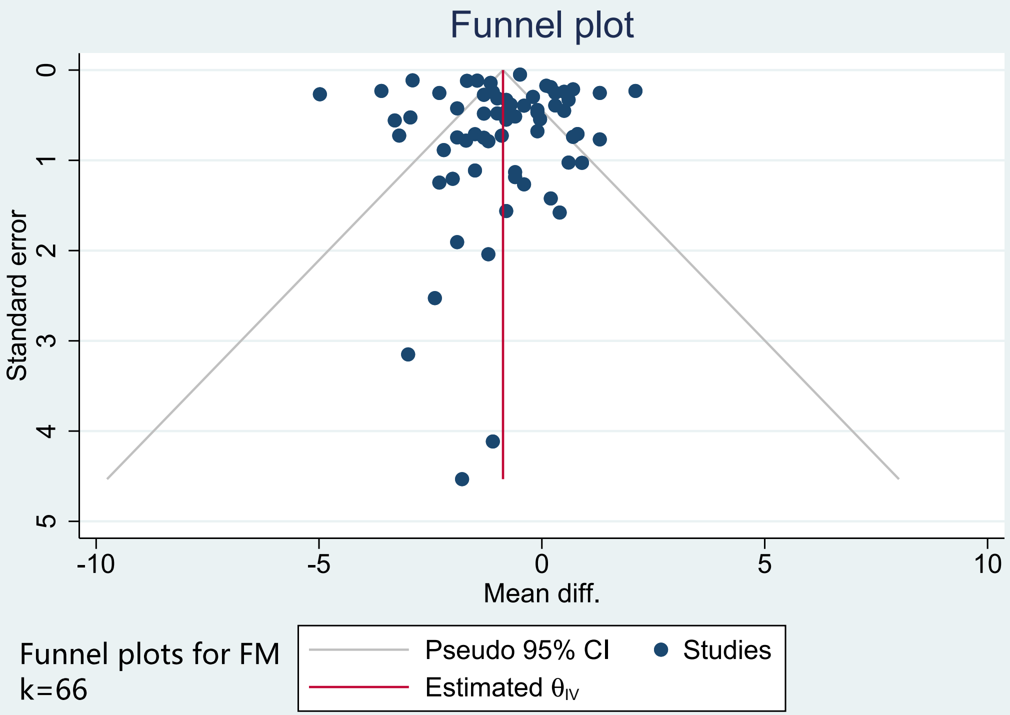

Funnel plots for FM
k=66

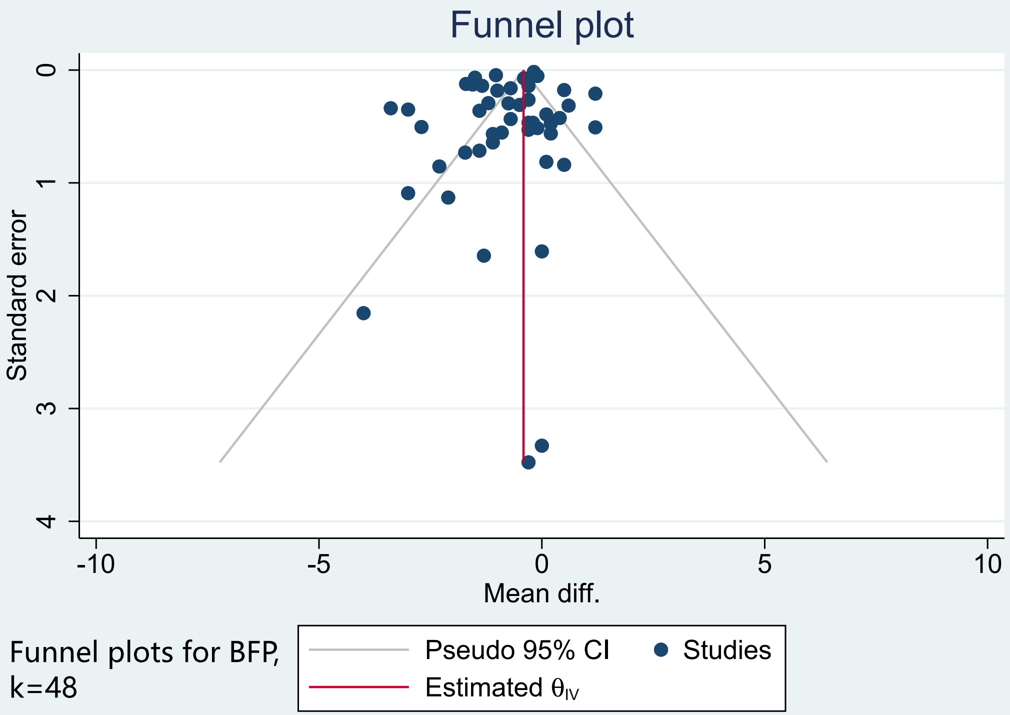

Funnel plots for BFP,
k=48

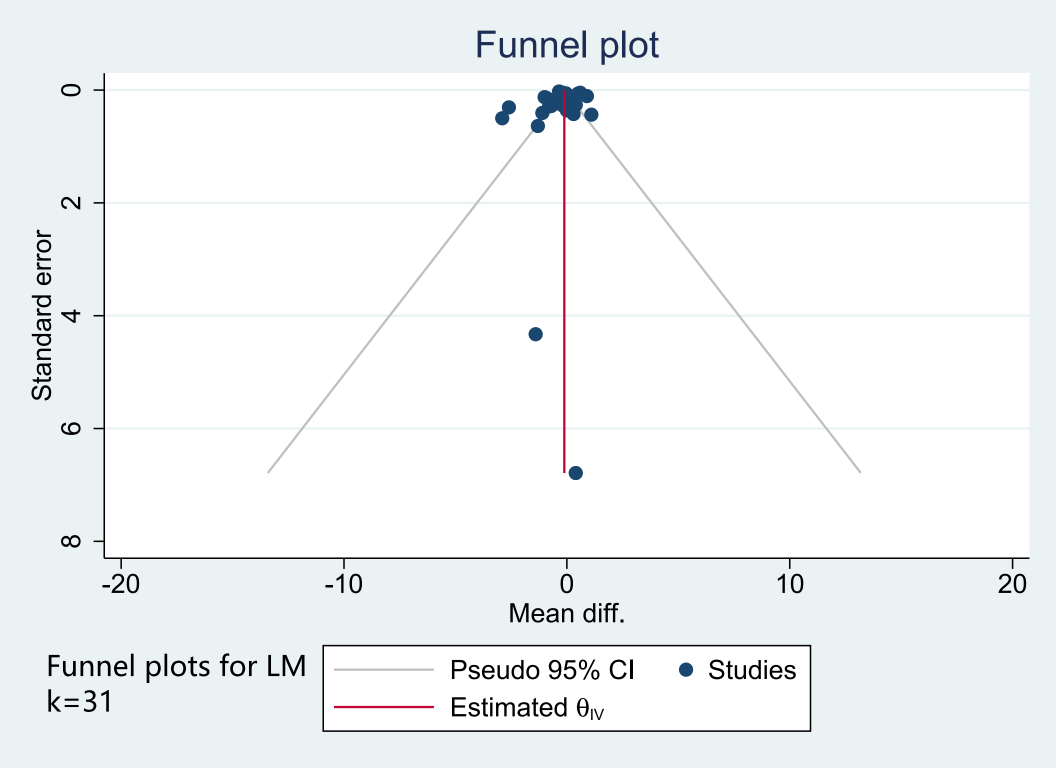

Funnel plot
Standard error
0
2
4
6
8
-20
-10
0
10
20
Mean diff.
Funnel plots for LM
k=31
Pseudo 95% CI
Studies
Estimated θIV

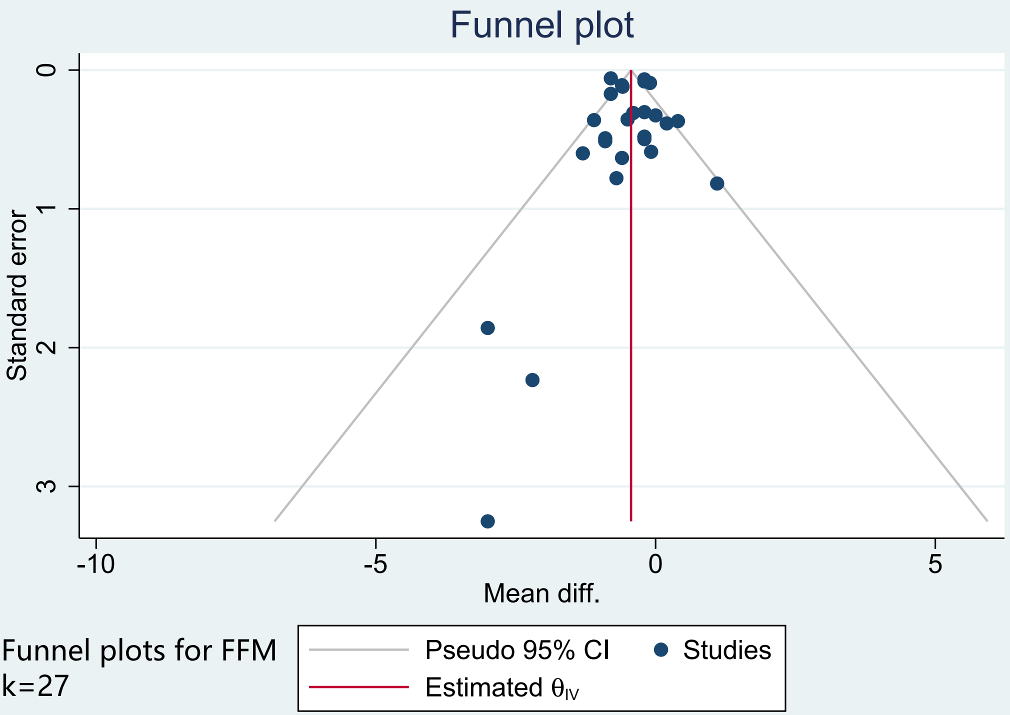

Funnel plots for FFM
k=27

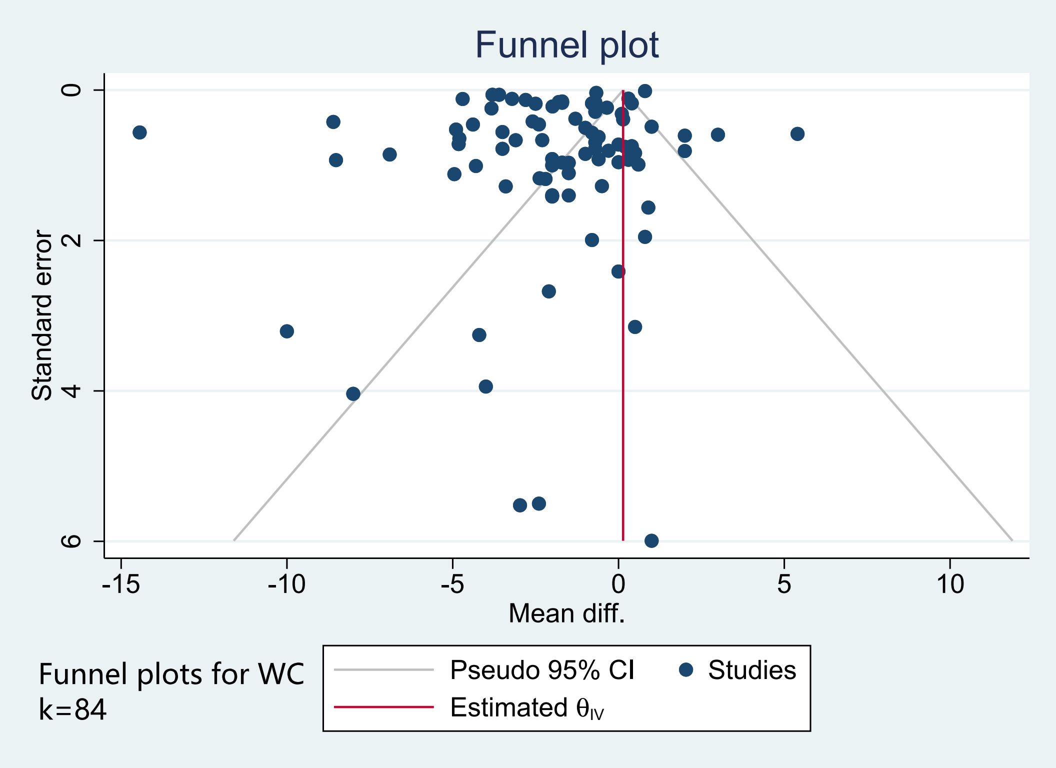
Funnel plot
Standard error
0
2
4
6
-15
-10
-5
0
5
10
Mean diff.
Funnel plots for WC
k=84
Pseudo 95% CI
Studies
Estimated θIV

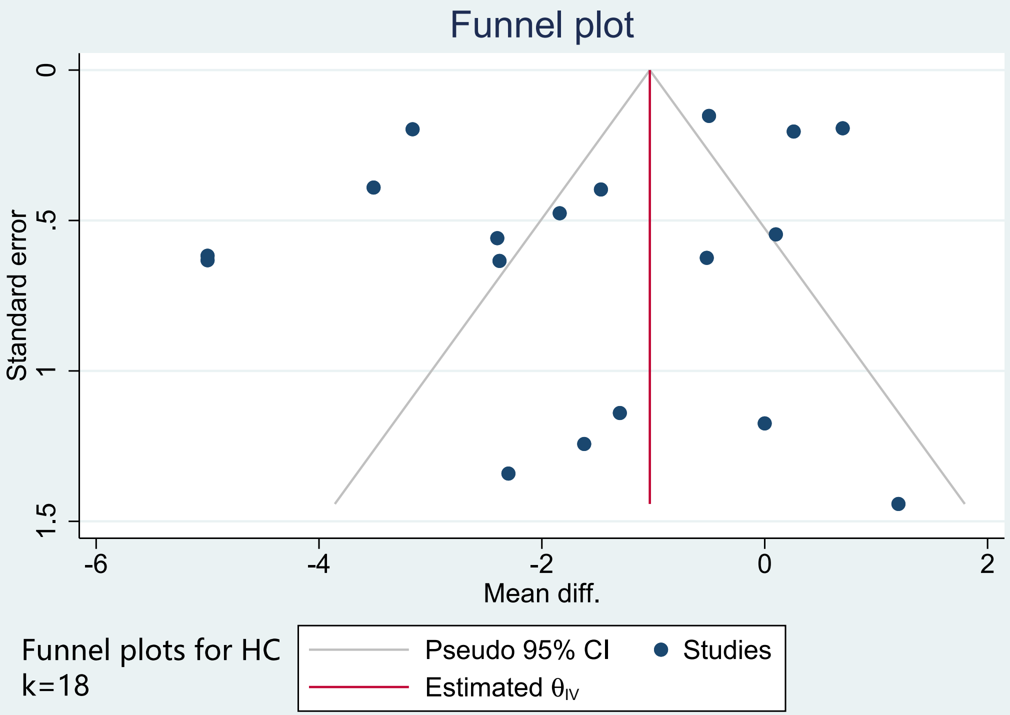

Funnel plots for HC
k=18

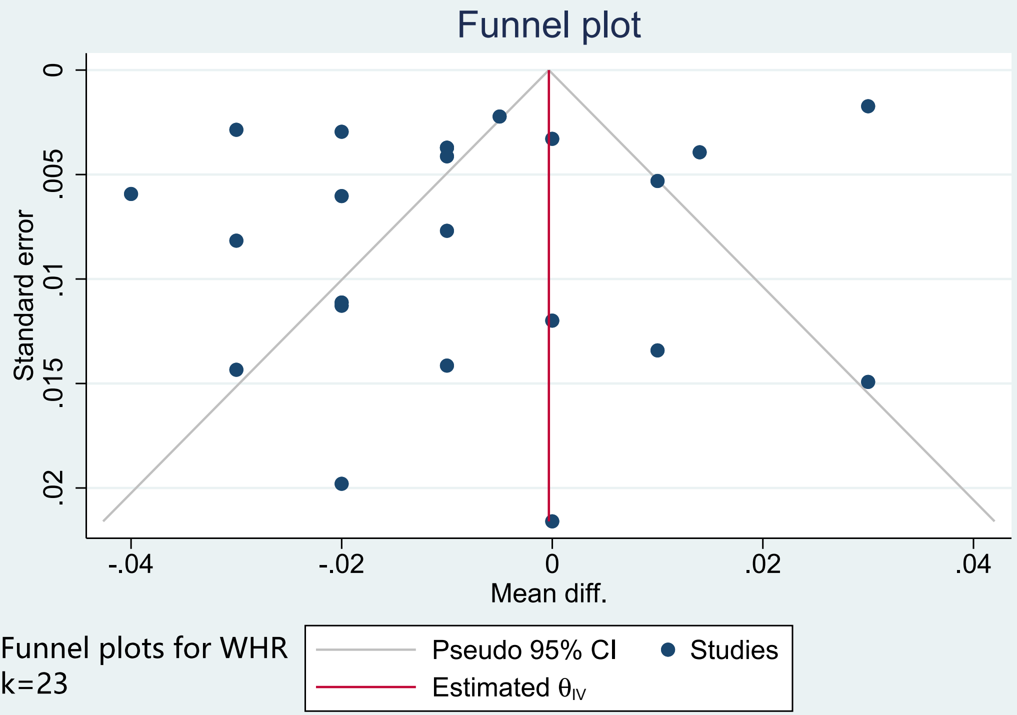

Funnel plots for WHR
k=23

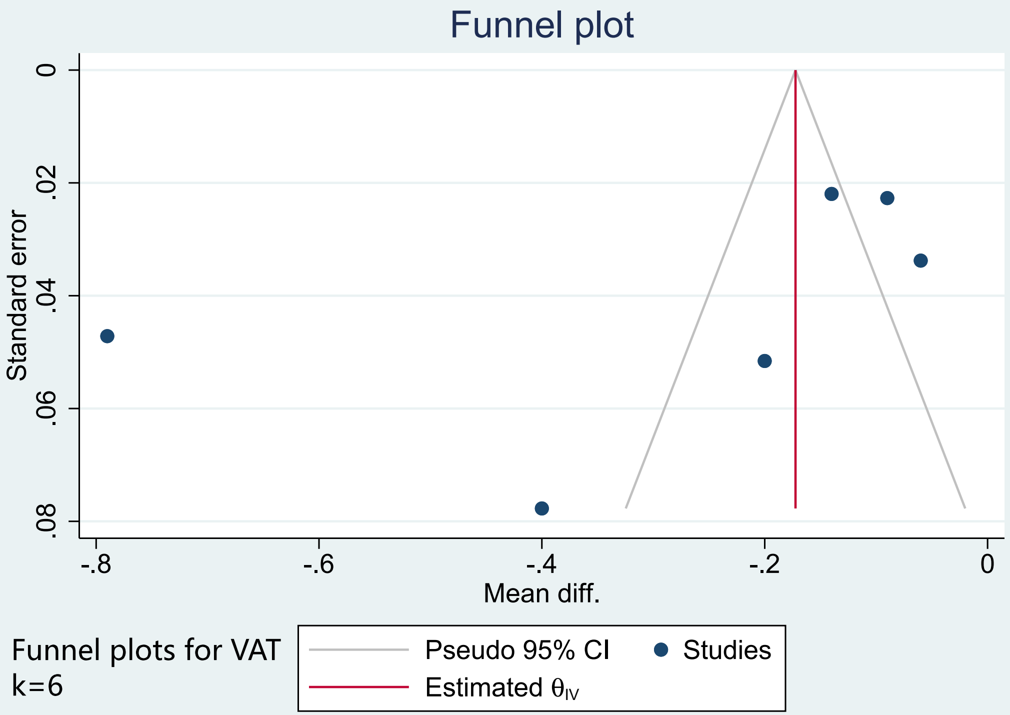

Funnel plots for VAT
k=6
